# Stereodirecting Effect of Esters at the 4-Position of Galacto- and Glucopyranosyl Donors: Effect of 4-*C*-Methylation on Side-Chain Conformation and Donor Reactivity, and Influence of Concentration and Stoichiometry on Distal Group Participation

**DOI:** 10.1021/acs.joc.3c01496

**Published:** 2023-09-07

**Authors:** Chennaiah Ande, David Crich

**Affiliations:** † Department of Pharmaceutical and Biomedical Sciences, University of Georgia, 250 West Green Street, Athens, Georgia 30602, United States; ‡ Department of Chemistry, University of Georgia, 302 East Campus Road, Athens, Georgia 30602, United States; § Complex Carbohydrate Research Center, University of Georgia, 315 Riverbend Road, Athens, Georgia 30602, United States

## Abstract

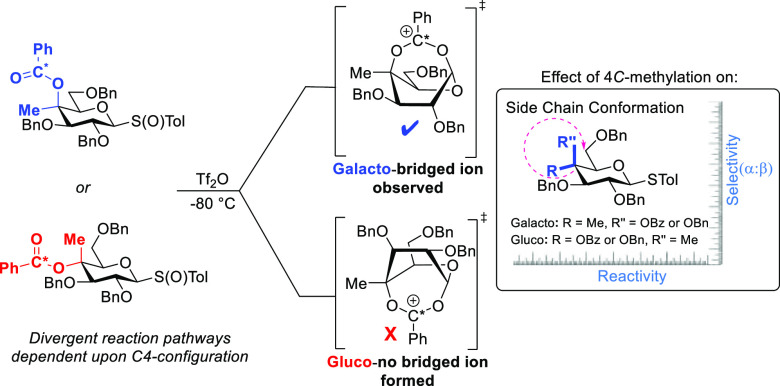

When generated in a mass spectrometer bridged bicyclic 1,3-dioxenium ions derived from 4-*O*-acylgalactopyranosyl, donors can be observed by infrared spectroscopy at cryogenic temperatures, but they are not seen in the solution phase in contrast to the fused bicyclic 1,3-dioxalenium ions of neighboring group participation. The inclusion of a 4-*C*-methyl group into a 4-*O*-benzoyl galactopyranosyl donor enables nuclear magnetic resonance observation of the bicyclic ion arising from participation by the distal ester, with the methyl group influence attributed to ester ground state conformation destabilization. We show that a 4-*C*-methyl group also influences the side-chain conformation, enforcing a *gauche,trans* conformation in gluco and galactopyranosides. Competition experiments reveal that the 4-*C*-methyl group has only a minor influence on the rate of reaction of 4-*O*-benzoyl or 4-*O*-benzyl-galacto and glucopyranosyl donors and, consequently, that participation by the distal ester does not result in kinetic acceleration (anchimeric assistance). We demonstrate that the stereoselectivity of the 4-*O*-benzoyl-4-*C*-methyl galactopyranosyl donor depends on reaction concentration and additive (diphenyl sulfoxide) stoichiometry and hence that participation by the distal ester is a borderline phenomenon in competition with standard glycosylation mechanisms. An analysis of a recent paper affirming participation by a remote pivalate ester is presented with alternative explanations for the observed phenomena.

## Introduction

**Scheme 1:**
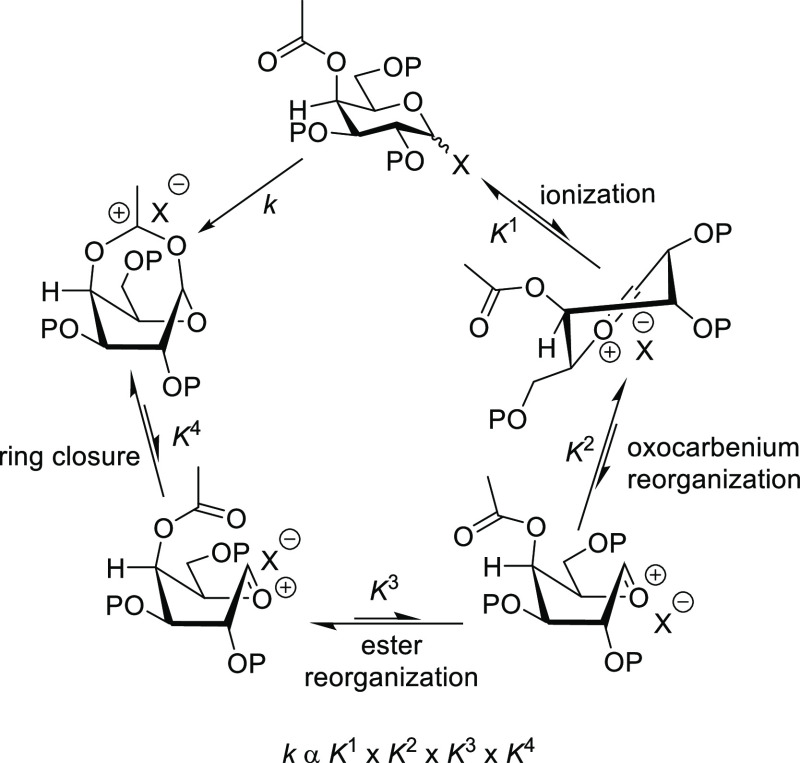
Unfavorable Equilibria in the Formation of the Bridged Dioxacarbenium Ions Required for DGP from 4-*O*-Acylgalactopyranosyl Donors

The development of practical, reproducible, and highly stereoselective glycosylation reactions is of critical importance to the glycosciences.^1−3^ In this respect, the concept of stereodirecting participation from distal esters (distal group participation, DGP) has recently received much attention with particular emphasis on the formation of α-galacto and α-fucopyranosides with DGP by esters at the 4-position.^4−9^ The DGP concept, a seemingly straightforward extrapolation of the well-established concept of neighboring group participation (NGP) involving the intermediacy of bridged bicyclic ions as intermediates, was introduced as long ago as 1972.^10,11^ The concept has received support from studies of the influence on selectivity of electron-donating and/or withdrawing groups on the directing ester, with Seeberger, Pagel, and co-workers most recently showing 4-*O*-pivalate esters to be more α-directing than 4-*O*-acetates.^5,12−14^ We, on the other hand, have argued against DGP in the form of bridged bicyclic ions on multiple grounds:^4,15^ (i) the experimentally demonstrated reduced thermodynamic stability of six- and seven-membered cyclic 1,3-dioxcarbenium ions compared to their five-membered counterparts,^16−18^ (ii) the established negative correlation of ring size with ring closure in simple non-carbohydrate models of 1,3-dioxacarbenium ion formation,^19^ (iii) the unstable ester conformation^4,20−22^ required for DGP, (iv) the failure of a variety of probes to trap the intermediate ions under typical glycosylation conditions,^23,24^ (v) the absence of spectroscopic evidence in the condensed phase for bridged bicyclic 1,3-dioxacarbenium ions as compared to fused bicyclic 1,3-dioxalenium ions, (vi) the failure of most models to take into account the stabilization of glycosyl oxocarbenium ions in the form of covalent intermediates by combination with counterions in the solution, (vii) the concatenation of unfavorable steps required, which renders the process kinetically unfavorable (summarized in Scheme 1), and (viii) the availability of other typically overlooked explanations for the experimental phenomena.

**Figure 1:**
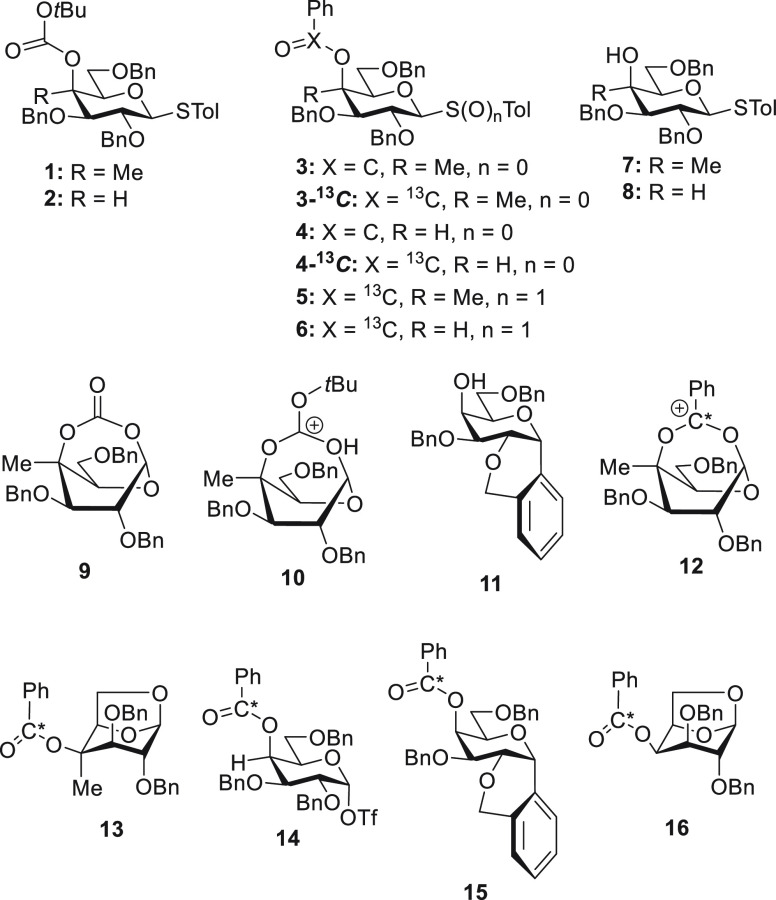
Donors **1**–**6**, alcohols **7** and **8**, the bridged ions **10** and **12**, galactosyl triflate **14**, and the decomposition products **9**, **11**, **13**, **15**, and **16**.

Previously, focusing on the barrier to DGP imposed by the unfavorable ester conformation required, we prepared a series of galactopyranosyl donors **1**–**6** from the corresponding alcohols **7** and **8** (Figure 1) and studied their reactions and decomposition products.^25^ Notably, activation of the 4-methyl-4-*O*-Boc derivative **1** with diphenyl sulfoxide and trifluoromethanesulfonic anhydride in dichloromethane at −80 °C followed by warming to room temperature gave a 56% yield of the bridged bicyclic carbonate **9**, indicative of *tert*-butyl group loss from the corresponding bicyclic ion **10**. In contrast, donor **2**, lacking the 4-*C*-methyl group, gave a complex reaction mixture in which no bridged bicyclic carbonate could be located and from which the only isolated product was the intramolecular Friedel–Crafts compound **11**. Activation of the 4-*C*-methyl-4-*O*-(^13^C_1_-benzoate) donor **5** with triflic anhydride in deuteriodichloromethane at −80 °C gave nuclear magnetic resonance (NMR) spectra interpreted as predominantly composed of the bridged bicyclic dioxacarbenium ion **12**, thereby providing the first direct spectroscopic evidence for DGP in solution. Gradual warming of the NMR probe revealed ion **12** to be stable up to −40 °C above which decomposition to the 1,6-anhydro derivative **13**, isolated in 41% yield, was observed. Analogous to the results with the 4-*O*-Boc derivatives, repetition of the experiment with the desmethyl analogue **6** did not provide any evidence for the corresponding bridged bicyclic ion but rather revealed the formation of the galactosyl triflate **14** on activation at −80 °C with decomposition to **15** and **16**, isolated in 27 and 29% yield, on warming above −20 °C.^25^ Thus, we found no evidence for DGP by esters of secondary alcohols at the 4-position of typical galactopyranosyl donors but demonstrated that the formation of bridged intermediates is possible from esters of tertiary alcohols at the same position. We interpret the change in behavior on going from secondary to tertiary esters as a consequence of increased conformational mobility in the tertiary systems and so in terms of removal of one of the unfavorable equilibria (*K*^3^) depicted in Scheme 1 thereby facilitating cyclization in manner akin to the well-known “*gem*-dimethyl” effect on cyclization reactions in general.^26^

We now turn our attention to other possible roles played by the methyl group in donors such as **1** and **5** beyond the facilitation of cyclization to bridged intermediates. Jensen and Bols demonstrated previously that 4-*C*-methylation of methyl α-D-galacto-and glucopyranoside leads to a minor reduction in the rate of acid-catalyzed hydrolysis compared to the parent glycosides, and presciently suggested that this may be due to a change in the conformation of the C5–C6 bond (side-chain conformation) imposed by the presence of the methyl group.^27^ Indeed, such a change in side-chain conformation is apparent from the observed variations in the ^1^H NMR coupling constants between H5 and the two diastereotopic H6’s in **1**–**8**, and it is now well-established that side-chain conformation modulates the reactivity and selectivity of glycosyl donors.^15,28,29^ In donor **5**, with the demonstrated propensity to form a bridged intermediate, there arises the further possibility of enhanced reaction rate through anchimeric assistance. To more fully understand the reactivity of galacto- and glucopyranosyl donors substituted with esters at the 4-position, and the manner in which it is influenced by 4-*C*-methylation, we have studied and report on the manner in which 4-*C*-methylation influences side-chain conformation in galacto- and glucopyranosides and, through the aegis of competition reactions, describe the effect of 4-*C*-methylation on glycosylation reaction rate. We also report on the role of concentration and stoichiometry in DGP discovered in the course of our experiments. Finally, we address the work of Seeberger, Pagel, and co-workers on the superiority of pivalate esters in DGP^5^ and argue against the use of gas phase experiments in the absence of counterions as predictors of solution phase reactivity in glycosylation reactions.

## Results and Discussion

### Influence of 4-*C*-Methylation on Side Chain Conformation

**Figure 2:**
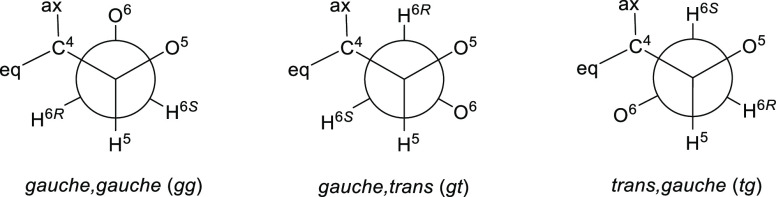
Staggered conformations of hexose side chains and their relationship to axial (ax) and equatorial (eq) substituents at C4.

The side-chain conformation of carbohydrates is usually considered in terms of an equilibrium mixture of three staggered conformers dubbed the *gauche,gauche* (*gg*), *gauche,trans* (*gt*), and *trans,gauche* (*tg*) conformers (Figure 2), wherein the first descriptor refers to the relationship of the C5–O5 and C6–O6 bonds and the second one to that of the C5–C4 and C6–O6 bonds.^30−32^

**Scheme 2:**
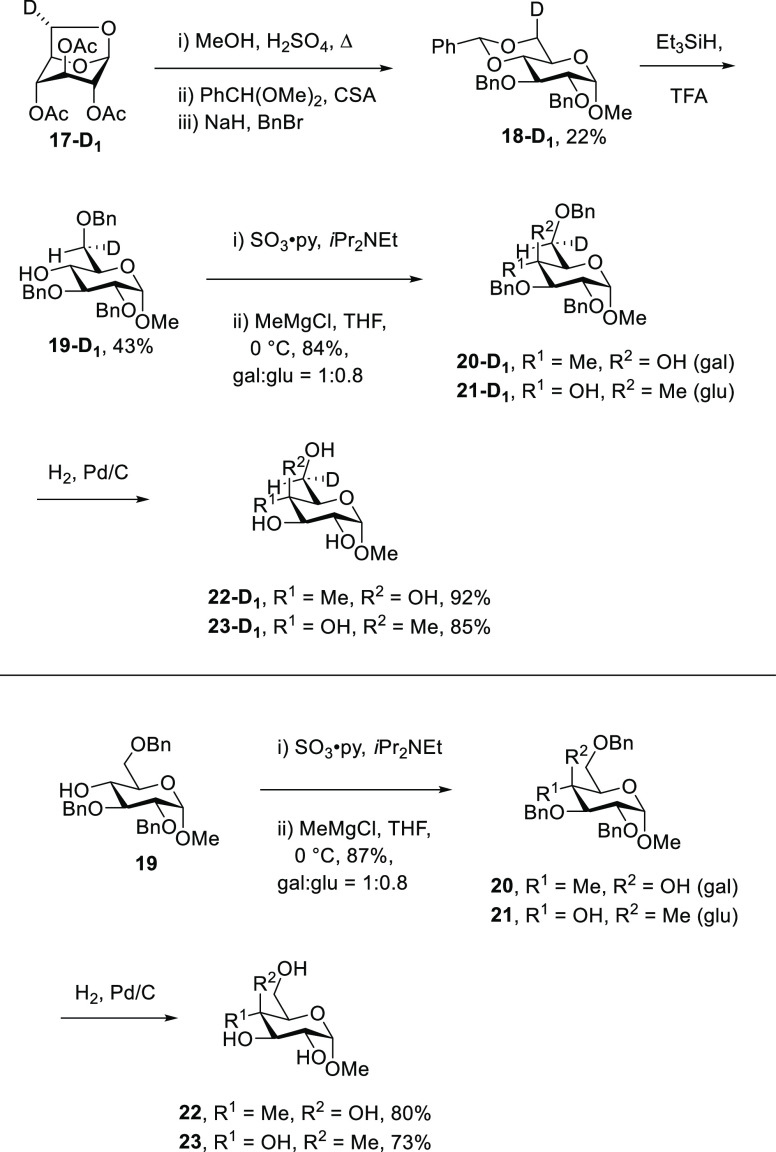
Synthesis of **19**–**23** and Their 6*S*-Deuterio Isotopomers

Analysis of the relative populations of the *gg*, *gt*, and *tg* conformers in the side chain of a given compound requires unambiguous assignment of the diastereotopic H6pro-*R* (H^6*R*^) and H6pro-*S* (H^6*S*^) protons, which is best achieved by stereospecific monodeuteriation.^33−38^ Thus, tri-*O*-acetyl-6*S*-deuterio-1,6-anhydro-d-glucose **17-D_1_**(38) was converted in 22% overall yield to methyl 2,3-di-*O*-benzyl-4,6-*O*-benzylidene-6*S*-deuterio-α-D-glucopyranoside **18-D_1_** by standard means. Reductive cleavage of the benzylidene acetal^39^ with triethylsilane and TFA^40^ then gave 43% of **19-D_1_** that was subjected to Parekh–Doering oxidation followed by treatment with methylmagnesium chloride resulting in a 1:0.8 mixture of the 4-*C*-methyl galacto- and gluco-derivatives **20-D_1_** and **21-D_1_** in 84% overall yield. Routine hydrogenolysis then afforded **22-D_1_** and **23-D_1_** in 92 and 85% isolated yield, respectively (Scheme 2). Unlabeled **22** and **23** were prepared analogously from unlabeled **19**(41,42) via **20** and **21** (Scheme 2).

**Figure 3:**
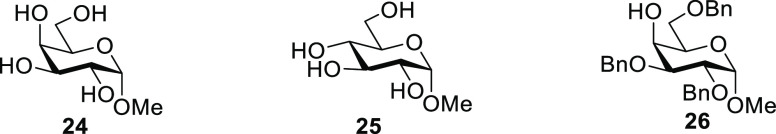
Literature compounds **24**, **25**, and **26**.

^1^H NMR spectra for compounds **22**, **23**, and their monodeuterio analogues were recorded at 500 in MHz in D_2_O and those of the partially benzylated derivatives **19**, **20**, and **22** in either CDCl_3_ or C_6_D_6_ for reasons of resolution, with assignments of the diastereotopic side-chain resonances made with the aid of the isotopomers, leading to the data presented in Table 1. Protected galacto- and glucopyranosides retain the same conformations of their respective side chains in CDCl_3_ and C_6_D_6_.^38,43^ The data for methyl α-D-galactopyranoside **24** and methyl α-D-glucopyranoside **25** (Table 1) are taken from the literature,^35^ while those for methyl 2,3,6-tri-*O*-benzyl-α-D-galactopyranoside **26**(44) and methyl 2,3,6-tri-*O*-benzyl-α-D-glucopyranoside **19**(45) (Figure 3) are taken from the literature with assignment of the pro*R* and pro*S* side chain hydrogens made by analogy with the corresponding rigorously assigned phenyl β-thioglycosides.^38^ With all assignments made and coupling constants determined, a standard population analysis was conducted,^46−48^ whereby eqs 1–3 were solved for the mole fractions of the three staggered conformations (*f_gg-tg_*) for all compounds by inputting the experimental coupling constants (^3^*J*_5,6*R*_ and ^3^*J*_5,6*S*_) and the limiting coupling constants (^3^*J*_*R*,*gg-tg*_ and ^3^*J*_*S*,*gg-tg*_) for each of the three staggered conformers determined with conformationally locked standards.^43^ In this analysis, negative populations of any given conformer are considered to arise from imperfections in the limiting coupling constants applied and the errors in the measurement of the experimental coupling constants (∼0.4 Hz) and are considered negligible if ≤−5%:^43^
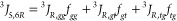
1
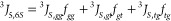
2
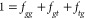
3

**Table 1:** Diagnostic Chemical Shifts, Coupling Constants, and Derived Side-Chain Conformations

**compound**	**solvent**	**configuration**	**C4 substitution**	**other protection**	δ_H5_	δ_H6*R*, H6S_	^3^*J*_5,6*R,*_ ^3^*J*_5,6*S*_	side-chain conformation		
								*gg*	*gt*	*tg*
**22**	D_2_O	galacto	Me, OH		3.64	3.62, 3.84	8.7, 2.2	24.8	78.1	–2.9
**24**	D_2_O	galacto	H, OH		3.92	3.70, 3.70	7.8, 6.0	3.7	50.7	45.6
**23**	D_2_O	gluco	Me, OH		3.66	3.57, 3.88	9.2, 2.1	20.7	83.6	–4.3
**25**	D_2_O	gluco	H, OH		3.69	3.76, 3.89	5.4, 2.3	56.0	44.0	0.0
**20**	C_6_D_6_	galacto	Me, OH	2,3,6-tri-*O*-Bn	3.92	3.88, 3.95	5.9, 2.6	49.0	47.8	3.2
**26**	CDCl_3_	galacto	H, OH	2,3,6-tri-*O*-Bn	3.90	3.68, 3.74	5.8, 5.7	25.6	31.8	42.6
**21**	CDCl_3_	gluco	Me, OH	2,3,6-tri-*O*-Bn	3.90	3.58, 3.72	7.0, 5.1	18.6	46.9	34.5
**19**	CDCl_3_	gluco	H, OH	2,3,6-tri-*O*-Bn	nd^a^	nd^a^	5.3, 4.1	43.1	34.5	22.4
**7**	CDCl_3_	galacto	Me, OH	2,3,6-tri-*O*-Bn	3.44	3.93, 3.82	5.5, 2.9	50.5	42.3	7.2
**8**	CDCl_3_	galacto	H, OH	2,3,6-tri-*O*-Bn	3.57	3.81, 3.76	5.7, 5.7	26.6	30.8	42.6
**3**	CDCl_3_	galacto	Me, OBz	2,3,6-tri-*O*-Bn	3.76	4.26, 3.89	7.5, 2.5	34.2	64.5	1.3
**4**	CDCl_3_	galacto	H, OBz	2,3,6-tri-*O*-Bn	3.87	3.69, 3.58	6.9, 5.9	13.3	42.0	44.7
**29**	CDCl_3_	galacto	Me, OBn	2,3,6-tri-*O*-Bn	3.53	3.79, 3.94	6.2, 3.6	38.2	46.0	15.8
**28**	C_6_D_6_	gluco	Me, OBz	2,3,6-tri-*O*-Bn	nd^a^	nd^a^	6.0, 2.9	45.7	47.3	7.0
**30**	CDCl_3_	gluco	Me, OBn	2,3,6-tri-*O*-Bn	3.74	3.66, 3.94	7.6, 2.1	36.3	67.4	–3.7

a Not determined due to insufficient resolution.

The installation of a methyl group at the 4-position of methyl α-D-galactopyranoside **24** gives compound **22** in which ^3^*J*_H5,6*R*_ is reduced by ∼1 Hz on incorporation of the methyl group, while ^3^*J*_H5,6*S*_ decreases from 6.0 to 2.2 Hz indicative of a significant change of the mean side-chain conformation on methylation. As is apparent from inspection of the population analysis of the three staggered conformers, this change involves depopulation of the *tg* conformation with correspondingly increased populations of the *gg* and *gt* conformers to the extent that the *gt* conformer dominates following C-methylation. Turning to methyl 2,3,6-tri-*O*-benzyl-α-D-galactopyranoside **26** and its 4-*C*-methyl derivative **20**, the inclusion of the methyl group again destabilizes and depopulates the *tg* conformer leading to correspondingly increased populations of the *gg* and *gt* conformers. In both **20** and **26**, the *gg* and *gt* conformations are approximately equally populated, which we attribute to an intramolecular hydrogen bond stabilizing the otherwise relatively disfavored *gg* conformation in the aprotic solvent.^38^ In the gluco-series, the inclusion of a 4-*C*-methyl group results in a reduction in population of the *gg* conformation and correspondingly increased populations of the *gt* and *tg* conformation, whether in D_2_O solution for the unprotected **23** and **25** or in CDCl_3_ solution for the 2,3,6-tri-*O*-benzylated compounds **19** and **21**. In the absence of protection at the 2, 3, and 6 positions in D_2_O the *gt* conformation is greatly favored (84%) in the 4-*C*-methyl gluco compound **23** just as it is in the unprotected galacto isomer **22**. With all but the 4-hydroxy group protected and in aprotic solution, the addition of the 4-*C*-methyl group in the gluco series results in an approximately 3:2:1 mix of the *gt*, *tg*, and *gg* conformers with the abnormally high population of the *tg* conformer in this case being due to the presence of an intramolecular hydrogen bond, consistent with previous results.^38^ Overall, it is apparent that a 4-*C*-methyl group in the galacto series (Figure 2, ax = OH, eq = Me) destabilizes the *tg* conformation because of a syn-pentane-type, or 1,3-interaction, with O^6^. In the gluco series, the addition of the methyl group (Figure 2, ax = Me, eq = OH) results in an analogous destabilization of the *gg* conformation. In the absence of intramolecular hydrogen bonding, the presence of the 4-*C*-methyl group gives rise to side-chain populations in both the galacto and gluco series that both co-incidentally strongly favor the *gt* conformation.

**Scheme 3:**
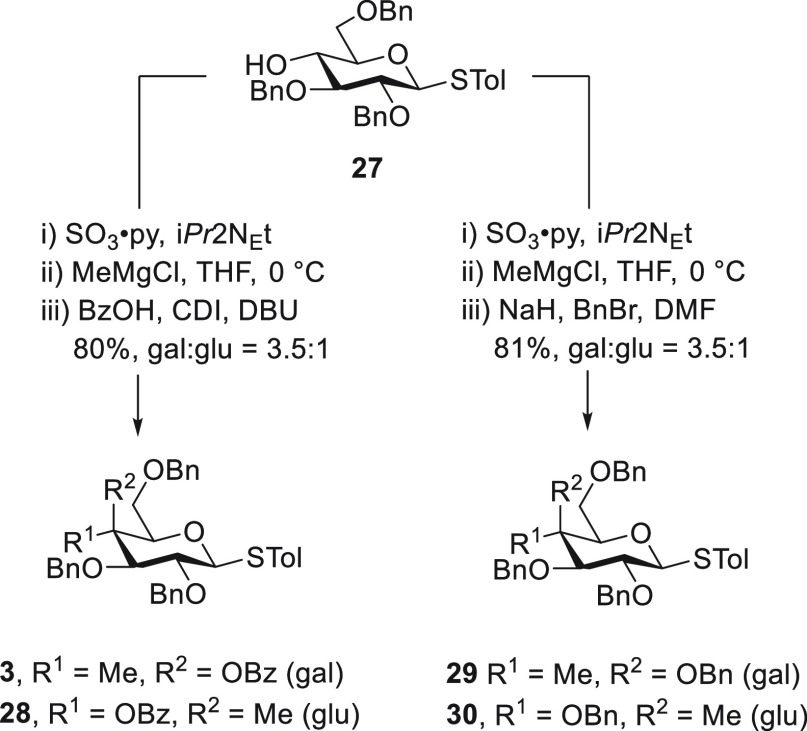
Synthesis of **3** and **28**–**30**

We next turned to the influence of protecting groups, benzoate esters or benzyl ethers, at the 4-position in conjunction with that of 4-*C*-methylation on side-chain conformation. To this end, we employed the *p*-tolyl β-D-thioglycospyranosides, with all compounds either obtained as described previously^25^ or as set out in Scheme 3 starting from *p*-tolyl 2,3,6-tri-*O*-benzyl-β-D-glucothiopyranoside **27**.^45^ Vázquez and coworkers have established that neither anomeric configuration nor the nature of the aglycon greatly influences glycoside side-chain conformation.^49−52^ Therefore, in this series of compounds, we simply assigned the pro*R* and pro*S* hydrogen resonances on the basis of their relative chemical shifts by analogy^30^ with the methyl α-pyranosides described above and with closely related and rigorously assigned phenyl β-D-thioglycopyranosides described previously.^38^

Comparison of the side-chain conformations of galactosyl thioglycosides **3** and **4** reveals that the inclusion of a 4-*C*-methyl group has a similar effect on the 4-*O*-benzoate esters as on the corresponding alcohols (**20** and **26**) to the extent that it strongly disfavors the *tg* conformation. However, in the absence of intramolecular hydrogen bonding stabilizing the *gg* conformation, the result is an approximately 1:2 mixture of the *gg* and *gt* conformers that approaches the population distribution seen for the tetraol **22** in D_2_O solution. The corresponding 4-*O*-benzyl ether **29** populates the *gg* conformer to a similar extent as the 4-*O*-benzoate **3** but has a somewhat enhanced population (15.8%) of the *tg* conformer that is achieved at the expense of a reduction in population of the *gt* conformer. In the glucosyl series, 4-*O*-benzoate **28** populates an approximately 1:1 mixture of the *gg* and *gt* conformers with only a minor contribution from the *tg* conformer. The corresponding 4-*O*-benzyl ether **30** is an approximately 1:2 mixture of the *gg* and *gt* conformations with no contribution from the *tg* conformation.

### Influence of 4-*C*-Methylation on Glycosylation

To investigate the influence of 4-*C*-methylation on glycosylation, both with and without possible anchimeric assistance from a benzoate ester at the 4-position, we designed a series of competition experiments in which equimolar mixtures of two glycosyl donors compete for a deficiency of glycosyl acceptor under similar conditions to those used in our investigation into the effect of methylation on DGP, namely, with activation by the diphenyl sulfoxide/triflic anhydride combination^53^ in dichloromethane at −78 °C. We used 1,2;3,4-di-*O*-isopropylidene galactopyranose **31** as acceptor, and buffered the reactions with 2,4,6-tri-*tert*-butylpyrimidine (TTBP). Because of the anticipated complexity of the competition reaction mixtures, we first conducted each of the individual glycosylation reactions and isolated and characterized the major products for use as authentic standards (Table 2).

**Table 2:**
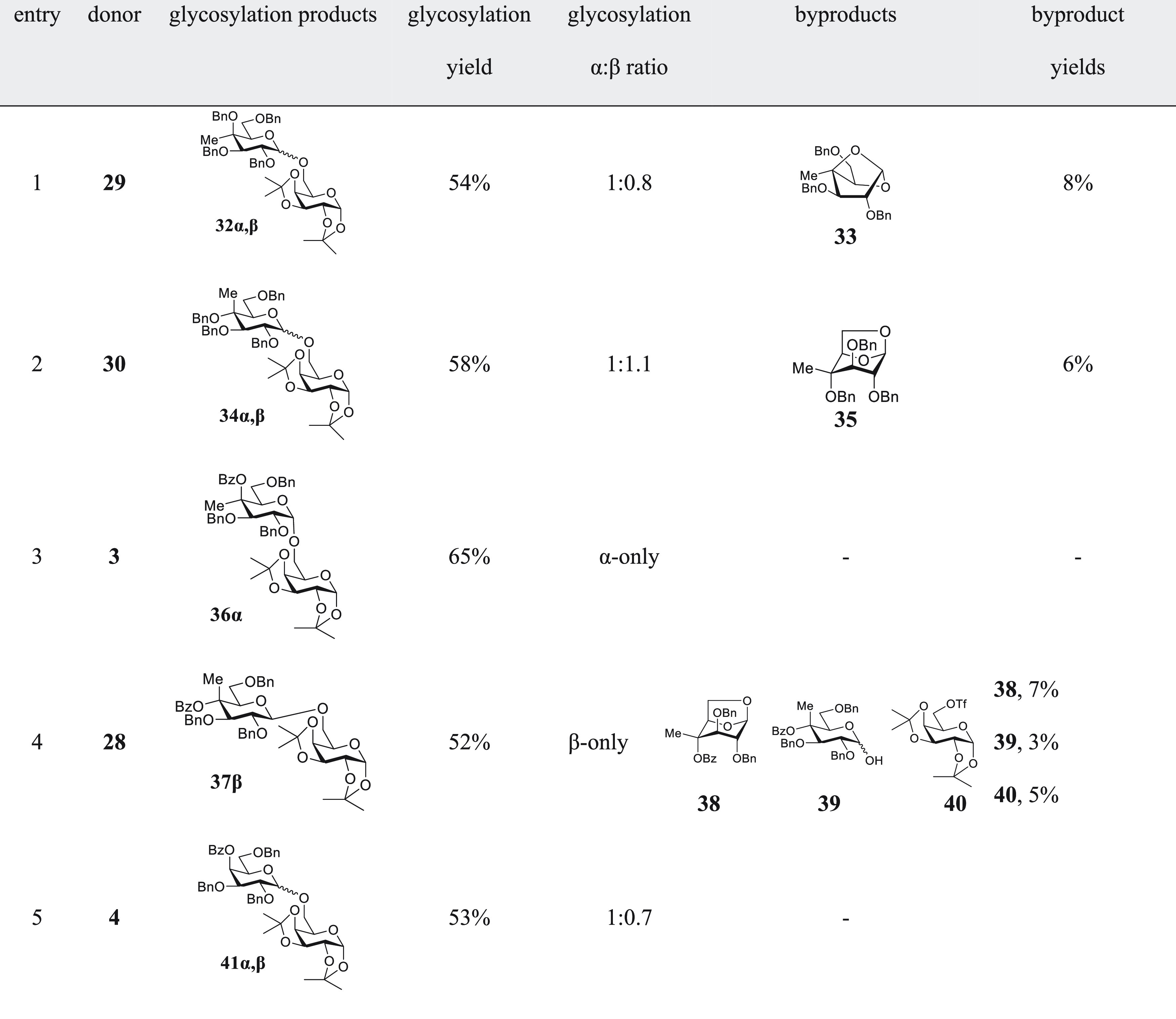
Influence of 4-C-Methylation on Glycosylation of 1,2;3,4-di-*O*-Isopropylidene Galactopyranose (**31**)

Comparison of Table 2 entries 1 and 3 reveals the influence of the 4-*O*-protecting group on selectivity, with the benzyl ether **29** showing essentially no selectivity and the benzoate ester **3** displaying complete α-selectivity. Comparison of entries 3 and 5 in Table 2 confirms the influence of the 4-*C*-methyl group in the benzoate series on selectivity with the desmethyl donor **4** showing only minimal selectivity for the α-glycoside in contrast with the complete α-selectivity seen with **31** in the presence of the methyl group. Comparison of entries 1 and 2 in Table 2 reveals the relatively minor effect of configuration at C4 on selectivity in the 4-*C*-methyl 4-*O*-benzyl series with both the galacto **29** and gluco **30** donors affording the coupled products with minimal selectivity. In comparing **29** with **30**, however, it is noteworthy that the major byproduct from the gluco donor **30** (Table 2, entry 2) was the 1,6-anhydro derivative **35**, whereas the major byproduct from the galacto donor **29** (Table 2, entry 1) was the more unusual 1,4-anhydro derivative **33**.^54^ Finally, comparison of the two 4-*C*-methyl-4-*O*-benzoyl donors **3** and **28** (Table 2, entries 3 and 4) reveals the far greater influence of configuration for donors carrying an ester at the 4-position than those with a 4-*O*-benzyl ether, as **3** and **28** both display complete but opposite selectivity. In addition, isolated from the reaction of **28** were the minor byproduct **38**, a 1,6-anhydro derivative, which is comparable to derivative **35** obtained in minor amounts from the reaction of the benzyl ether **30**, the hemiacetal **39**, and the triflated acceptor **40**.

**Scheme 4:**
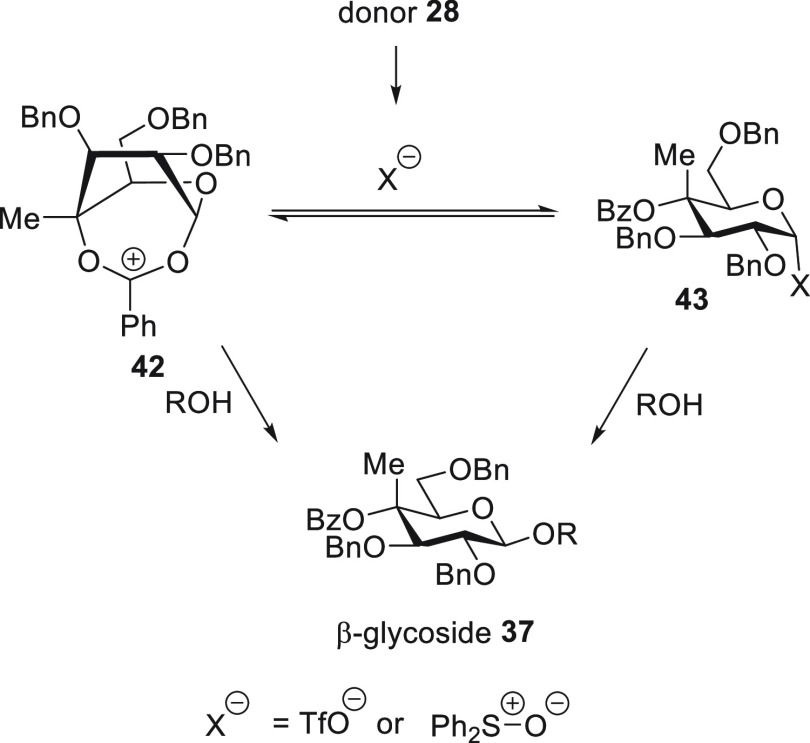
Possible Reaction Pathways for the Formation of β-Glucoside **37** from Donor **28**

The high β-selectivity observed in the formation of **37** from the gluco-donor **28** (Table 2, entry 4) could be interpreted in terms of DGP by the benzoate group through an intermediate bridged ion **42**, or simply by S_N_2 displacement of the covalent donor **43** (Scheme 4). In the case the selectivity arises through S_N_2-like displacement from the covalent donor **43**, the difference in selectivity between the 4-*O*-benzoyl and 4-*O*-benzyl donors **28** and **30**, respectively (Table 2, entries 4 and 2), would be due to the stronger electron-withdrawing ability of the benzoate ester and the consequent stabilization of the covalent donor.^55,56^ DGP from esters at the 4-position of mannosyl donors via bridged ions related to **42** has been advocated in the literature,^57^ but in previous work we have failed to find any evidence in support of it.^23^

**Figure 4:**
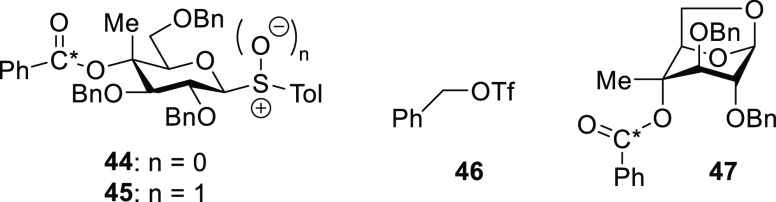
Structures of isotopically enriched donors **44** and **45**, benzyl triflate **46**, and of 1,6-anhydro sugar **47**.

To probe the possibility of DGP with donor **28**, we synthesized the isotopomer 99% enriched in ^13^C at the carbonyl C of the benzoate ester **44** (Supporting Information) and oxidized **44** to the glycosyl sulfoxide **45** (Figure 4) for ease of activation at −80 °C (Supporting Information). Sulfoxide **45** was activated at −80 °C in CD_2_Cl_2_ solution in the presence of TTBP by the addition of Tf_2_O, and the ^1^H and ^13^C spectra were quickly recorded. The reaction mixture was then allowed to warm in 10 °C increments in the probe of the NMR spectrometer with spectra recorded at every stage (Supporting Information). Complete activation was observed at −80 °C and gave rise to relatively complex spectra containing several apparent activated species and resonances attributed to benzyl triflate **46**^58^ along with minor amounts of ^13^C enriched 1,6-anhydro sugar **47** (Figure 4).^58^ On warming, the proportions of **46** and **47** in the reaction mixture gradually increased until −20 to −10 °C when the signal attributed to **46** was lost. Workup of the reaction mixture after it reached room temperature allowed isolation of the ^13^C enriched 1,6-anhydro sugar **47** in 31% yield. Most importantly, and in stark contrast to the galactopyranosyl series and the ready observation of ion **12**,^25^ at no stage of this NMR experiment was any evidence observed for the formation of a bridged bicyclic ion **42**. We conclude that DGP through a cyclic ion such as **42** is highly unlikely for donors with equatorial esters at the 4-position and consequently that the high β-selectivity in the coupling of donor **28** with diisopropylidenegalactopyranose **31** (Table 2, entry 4) is the result of stabilization of an activated covalent intermediate **43** by the electron-withdrawing effect of the remote ester, with subsequent S_N_2-like displacement.

Turning to the competition experiments, equimolar mixtures of two donors and acceptor **31** were activated in dichloromethane at −78 °C in the presence of TTBP: After stirring at −78 °C for 20 h, the reaction mixtures were quenched and analyzed by reverse phase-HPLC with detection by ultraviolet and electrospray ionization (ESI)-mass spectrometry. In view of the complexity of the reaction mixtures, no attempt was made to isolate or characterize the saccharides or the various byproducts formed, whose presence was nevertheless confirmed by mass spectrometry, but attention was focused on the relative proportions of the unreacted donors, leading overall to the results set out in Table 3.

**Table 3:**
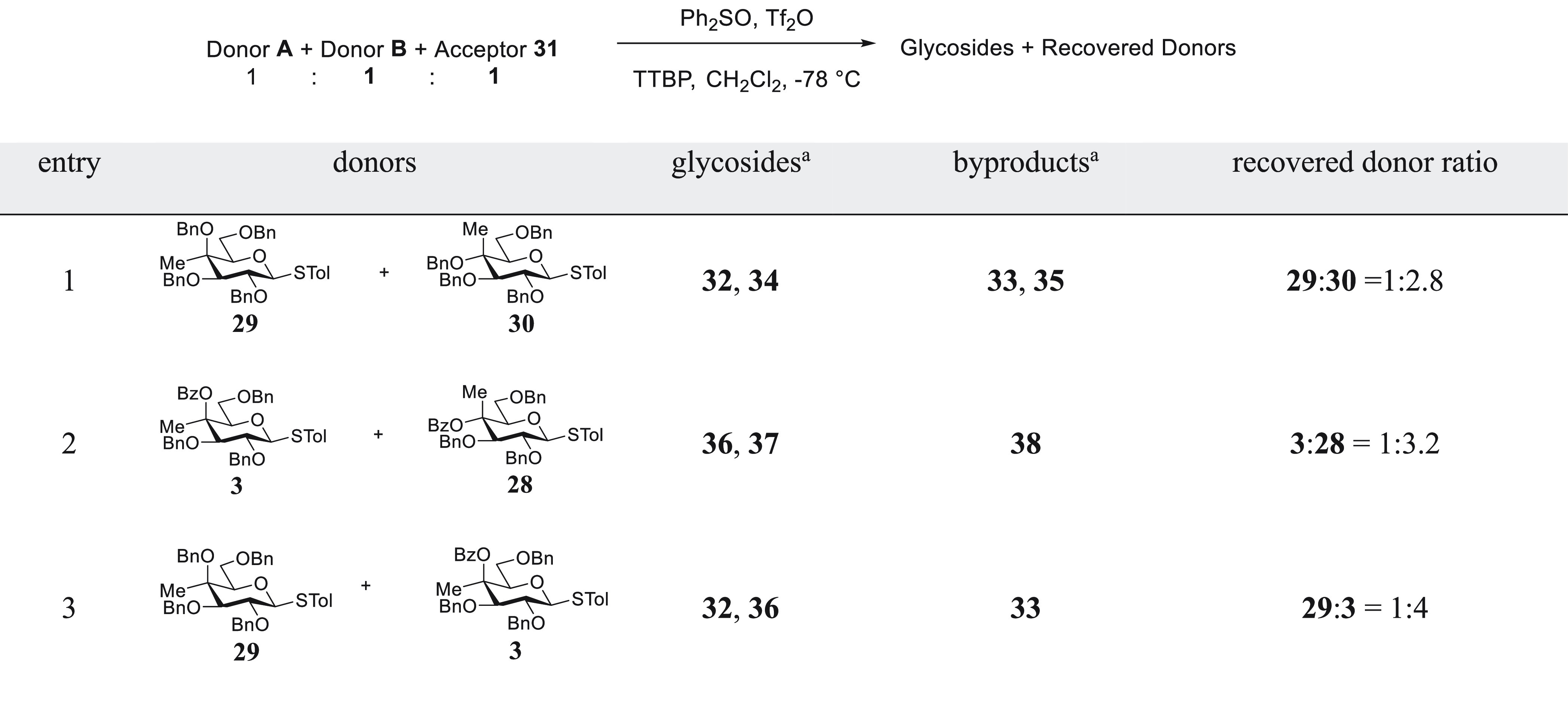
Competition Reactions

a Identified by ESI mass spectrometry in the reaction mixture.

The competition reaction between the two benzylated 4-*C*-methyl donors **29** and **30** (Table 3, entry 1), or between the two benzoylated donors **3** and **28** (Table 3, entry 2), shows that in a given pair of isomers the galacto-isomer is moderately more reactive than its gluco-counterpart, such that the inclusion of the methyl group does not alter the very well-established relative anomeric reactivity patterns of galacto and gluco isomers.^59−61^ The very similar ratios of recovered donors in Table 3, entries 1 and 2 also rule out the possibility of any significant anchimeric assistance by the 4-*O*-benzoate ester in donor **3** in spite of its predisposition toward participation demonstrated by the formation of the bridged ion **12** in NMR experiments conducted in the absence of acceptor alcohol. This absence of anchimeric assistance by the remote ester is consistent with multiple investigations into the possibility of kinetic acceleration of NGP by esters at the 2-position of 1,2-*cis*-glycosyl donors.^4^ Likewise, anchimeric assistance in the subsequent reaction of an activated 1,2-*trans* species, such as an α-glycosyl triflate or glycosyl oxysulfonium ion, can be excluded on the basis of these results. The third competition reaction, between the 4-*O*-benzyl and 4-*O*-benzoyl 4-*C*-methyl galactopyranosyl donors, **29** and **3**, respectively, demonstrates that the ether-protected system **29** is moderately more reactive than its esterified congener **3** (Table 3, entry 3). Thus, the presence of the 4-*C*-methyl group and the associated change in conformation of the ester in **3** and the side-chain conformation in both **3** and **29**, with respect to the desmethyl analogues, does not perturb the classical reactivity sequence according to which esters are more disarming than ethers in glycosylation reactions.^56^

### Influence of Concentration and Stoichiometry on Glycosylation Selectivity

Perhaps the most striking result in Table 2 is the fully α-selective coupling of the 4-*C*-methyl-4-*O*-benzoyl galactopyranosyl donor **3** with diacetone galactopyranose **31** (Table 2, entry 3), which stands in contrast to the 1.3:1 α/β ratio we reported previously under similar conditions.^25^ We traced this apparent discrepancy with our earlier work to the differences in concentration and stoichiometry between the previous experiment^25^ and the one reported in Table 2. Thus, the original experiments^25^ were conducted with a 0.1 M solution of donor **3** and 80 mole % of acceptor **31**, whereas the experiments reported in Table 2 used a 0.05 M solution of **3** and a full equivalent of acceptor. In addition, the earlier experiment also employed 200 mole percent of diphenyl sulfoxide and 150 mole % of triflic anhydride, whereas the experiments reported in Table 2 used 100 mole % of diphenyl sulfoxide and 100 mole % of triflic anhydride. In each case, sufficient base (TTBP) was present to buffer the triflic acid formed in the course of the reaction. All of the changes from the published work were instituted in preparation for the competition experiments reported in Table 3, which require that the reactions not go to more than approximately 50% overall completion. To determine whether the change in selectivity resulted from the change in concentration or the change in diphenyl sulfoxide stoichiometry, we first repeated the coupling of **3** with diacetone galactopyranose **31** under the previous conditions, obtaining essentially the same results, and then conducted two further couplings independently varying overall concentration and diphenyl sulfoxide stoichiometry leading to the results presented in Table 4.

**Table 4:**
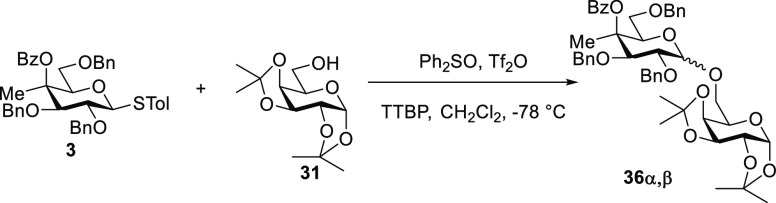
Influence of Concentration and Diphenyl Sulfoxide Equivalence on the Coupling of Donor **3** with Diacetone Galactopyranose **31**

entry	[donor] (M)	[acceptor] (M)	[Ph_2_SO] (M)	[Tf_2_O] (M)	[TTBP] (M)	**36**, %yield	**36**, α/β ratio
1^a^	0.05	0.05	0.05	0.05	0.075	65%	α only
2^b^	0.10	0.083	0.20	0.15	0.20	45%	α/β = 1.2:1
3	0.10	0.083	0.10	0.15	0.20	42%	α/β = 1:1
4	0.05	0.05	0.10	0.05	0.075	51%	α/β = 3.2:1

a Reproduced from Table 2, entry 3 for ease of comparison.

b Conditions reported previously.^25^

**Scheme 5:**
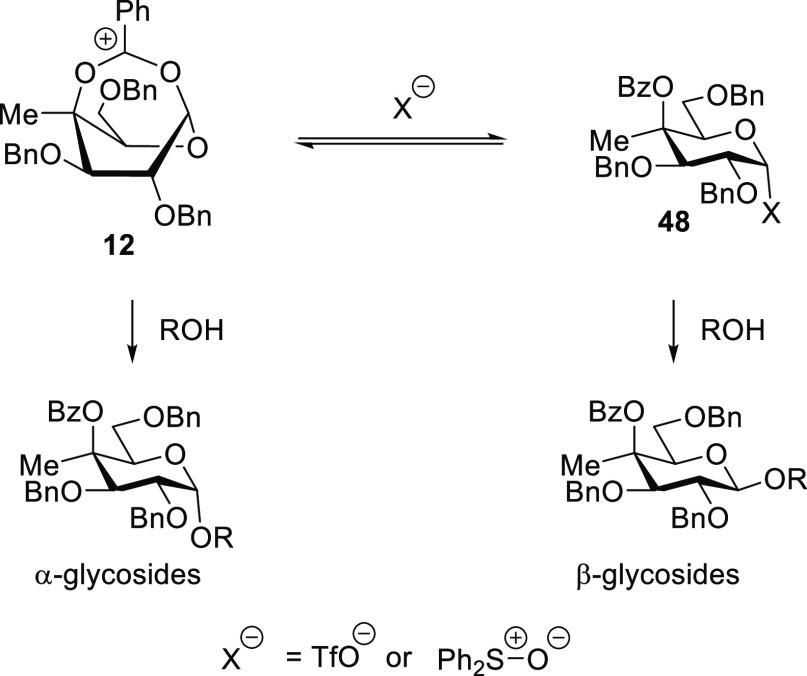
Concentration and Additive-Dependent Nature of DGP Following Activation of Donor **3**

From the data presented in Table 4, it is evident that both the overall reaction concentration and the presence of excess diphenyl sulfoxide in the reaction mixture impact the selectivity of coupling of donor **3** to diacetone galactopyranose **31**. Thus, from a comparison of Table 4, entries 1 and 4, it is clear that at the lower concentration of 0.05 M donor and acceptor, the presence of excess diphenyl sulfoxide reduces the α/β-selectivity significantly. At the higher concentration of both donor and acceptor, the α/β ratio is reduced further again (Table 4, entries 2 and 3), but it is the reaction containing free diphenyl sulfoxide that is marginally more α-selective. We interpret these results as demonstrating the borderline nature of DGP by the benzoate ester in **3**, having previously drawn attention to what we consider to be the borderline nature of even NGP mechanisms in glycosylation.^15^ These observations can be understood in terms of the equilibrium between the bridged ion **12** resulting from DGP, ultimately leading to the α-product, and a more typical covalent donor, either a glycosyl sulfoxide or a glycosyl oxysulfonium ion **48** that is the precursor of the β-product (Scheme 5). Both glycosyl triflates^62^ and glycosyl oxysulfonium ions^63^ have been characterized multiple times,^64^ with the oxysulfonium ions being considered to be the more stable of the two.^63^

### Influence of a 4-O-Pivalate Ester on Galactopyranosylation

**Figure 5:**
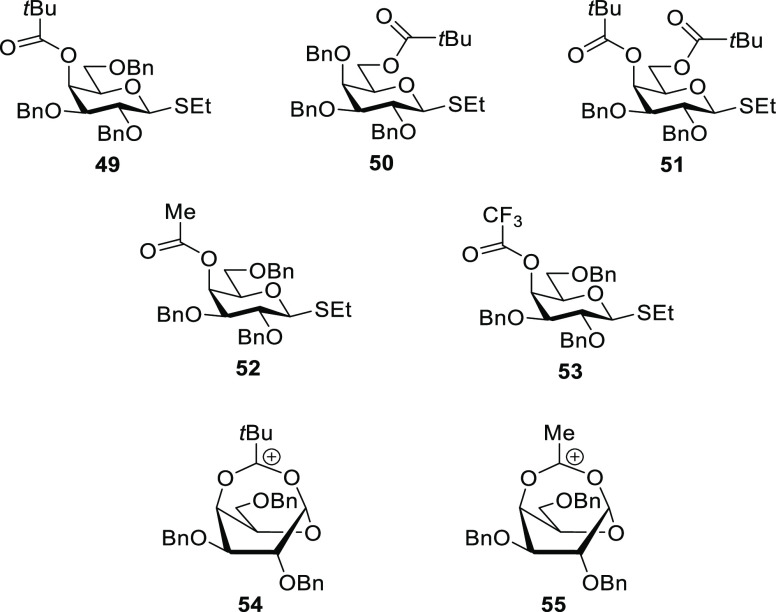
Donors and intermediates considered by Seeberger, Pagel, and co-workers.

Finally, we address the origin of the stereodirecting influence of pivalate esters at the 4- and 6-positions of galactosyl donors described recently by Seeberger, Pagel, and co-workers.^5^ Briefly, the authors of this paper reported that the 4-*O*-pivalate protected donor **49** gave excellent α-selectivity in glycosylation reactions conducted with activation by *N*-iodosuccinimide and triflic acid in dichloromethane at −20 °C and that this selectivity was superior to that seen with the corresponding 6-*O*-pivalate **50**, the 4,6-di-*O*-pivalate **51**, the 4-*O*-acetate **52**, and the 4-*O*-trifluoroacetate **53** (Figure 5). On the basis of cryogenic gas phase infrared measurements on isolated ions generated in a mass spectrometer with support by density functional theory investigations also carried out in the absence of solvent and counterion, the authors concluded that the optimal α-selectivity seen with **49** was the result of DGP via a bridged ion **54**. They further concluded that **54** was more stabilized with respect to the corresponding simple oxocarbenium ion than the corresponding bridged ion **55** arising from the 4-*O*-acetate because of the greater electron-donating ability of the *tert*-butyl group in **54**. No evidence for DGP was found with the trifluoroacetate **53** although it gave reasonable α-selectivity under the glycosylation conditions employed, certainly better than that seen with the acetate **52**. Alternative explanations for the increased α-directing effect of electron-rich esters at the 4-position of galactosyl donors^4,15^ were not considered.

We have previously shown that the presence of a either 4-*O*- or a 6-*O*-pivalate ester does not change the mix of *gg*, *gt*, and *tg* side-chain conformers of galactopyranosyl donors carrying benzyl ethers on the remaining hydroxyl groups with respect to the corresponding acetates.^38^ The presence of a 4-*O*-trifluoroacetate ester, however, has a significant influence in that the population of the *tg* conformer increases to 75% from the 55% seen in both the 4-*O*-pivalate and acetate esters, with corresponding reductions in the populations of the *gg* and *gt* conformers. The strongly electron-withdrawing effect of the 4-*O*-trifluoroacetate ester is therefore reinforced by the change in side-chain conformation it confers, which logically leads to stabilization of covalent intermediates such as the β-galactosyl triflate and the strong α-selectivity seen by Seeberger, Pagel, and co-workers with donor **53**. As we had not previously investigated the combined influence of pivalate groups at the 4- and 6-positions on the side-chain conformation of a galactopyranosyl donor, we prepared donor **51** by standard means from the corresponding diol.^5^ The ^3^*J*_H5,H6a_ and ^3^*J*_H5,H6b_ coupling constants of **51** that report on its side-chain conformation were found to be 7.3 and 6.0 Hz, respectively, and fully consistent with those of the corresponding 2,3,6-tri-*O*-benzyl-4-*O*-pivaloyl and 2,3,4-tri-*O*-benzyl-6-*O*-pivaloyl donors,^38^ and we conclude that there is no interaction between the 4- and 6-*O*-pivalate esters in **51**.

**Figure 6:**
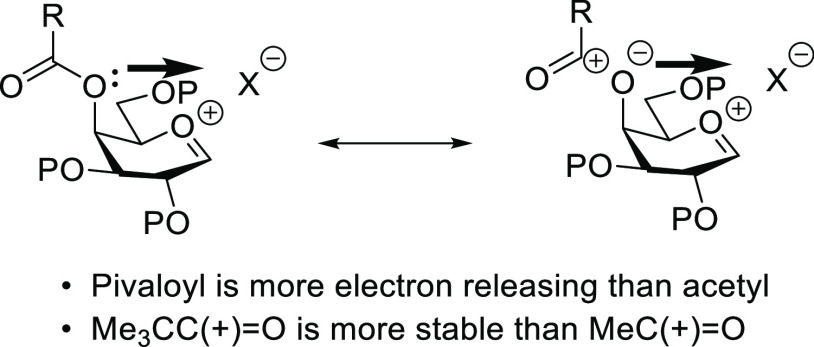
Non-DGP model for α-directing influence of electron-rich esters at galactose O4.

As we have shown with donor **6**, in the solution phase there is no evidence for the formation of a cyclic dioxocarbenium ion bridging the 1- and 6-positions, rather the anticipated covalent glycosyl triflate is observed:^25^ This observation is bolstered by multiple failed experiments designed to trap such an intermediate.^23,25^ A bridged ion such as **12** only becomes observable in the solution phase once the ground state ester conformation is destabilized by the presence of a well-placed substituent, when trapping experiments for related ions are also successful.^25^ Boons and coworkers previously found the pivalate **49** to give an α/β-selectivity of 16:1 on activation with *N*-iodosuccinimide and trimethylsilyl triflate in 1:1 toluene/dioxane, whereas the corresponding 4-*O*-benzoate gave a selectivity of 17:1 suggesting that the pivalate ester is at best marginally more advantageous than a benzoate ester.^12^ There is no reason to believe that the ground state conformation of a pivalate ester is any different from that of acetate or benzoate esters and consequently no reason to believe that pivalate esters have any special propensity toward DGP. We conclude that the experimental observations of Seeberger, Pagel, and co-workers on the very high α-selectivity seen with donor **49**, while certainly significant from a preparative standpoint, likely do not arise from DGP in the form of the bridged ion **54**. We stress that experiments demonstrating the formation of such bridged ions in the absence of the counterion either in the gas phase or in silico have little or no relevance to the solution phase, where the correct reference point is the covalent donor and not a naked oxocarbenium ion.^4,15^ We propose, consistent with our previous suggestions on the influence of electron density in the modulation of selectivity by 4-*O*-carboxylate esters in galactopyranosylation,^4,15^ that the advantageous effect of the pivalate ester as compared to the corresponding acetate can be attributed to the increased electron density on O4 of the galactosyl donor, as a result of electron donation by the *tert*-butyl group, and the consequent increased electrostatic stabilization of (partial) positive charge at the anomeric locus and a consequent shift of the general reaction mechanism toward looser and more α-selective ion pairs. This hypothesis is best appreciated by consideration of the minor ester no-bond resonance form in Figure 6, is consistent with the increased stability of the pivaloyl cation over the acetyl cation,^65,66^ and models for the enhanced reactivity of galactopyranosyl over glucopyranosyl systems that invoke stabilization of positive charge at the anomeric center by electrostatic interaction with the electron density at O4 in galactose and related systems.^67−70^

## Conclusions

Installation of a single methyl group at C4 of galacto- and glucopyranosyl donors changes the relative populations of the three staggered conformers of the side chain by destabilizing the *t*g conformation in the galacto- series and of the *gg* conformation in the glucopyranosyl series. This leads to the preferential adoption of the *gt* conformation in both 4-*C*-methyl galacto- and glucopyranosyl donors. Competition experiments between galacto- and glucopyranosyl donors bearing a 4-*C*-methyl group reveal the galacto isomer to be moderately more reactive than their gluco counterparts and that ether-protected 4-*C*-methyl donors are more reactive than ester-protected ones consistent with conventional reactivity patterns. No evidence was found in support of anchimeric assistance by a 4-O-benzoate ester even in the presence of a 4-*C*-methyl group. Overall, we conclude that the presence of a 4-*C*-methyl group does not significantly impact the reactivity of a galacto or a glucopyranosyl donor carrying either a benzyl ether or a benzoate ester at the 4-position. The stereochemical outcome of 4-*O*-benzoyl-4-*C*-methyl galactopyranosylation is dependent on both concentration and stoichiometry of the reagent leading to the conclusion that, even in this most favorable of cases, DGP at best is a borderline phenomenon.

## Data Availability

The data underlying this study are available in the published article and its Supporting Information.

## Supporting Information Available

Full experimental details and copies of NMR spectra (PDF)
