# Supplementary material for: Stereodirecting Effect of Esters at the 4-Position of Galacto- and Glucopyranosyl Donors: Effect of 4-C-Methylation on Side-Chain Conformation and Donor Reactivity, and Influence of Concentration and Stoichiometry on Distal Group Participation
Source: J Org Chem. 2023 Sep 7;88(19):13883–93. doi: 10.1021/acs.joc.3c01496 (PMC10563135; doi:10.1021/acs.joc.3c01496)

Supporting Information

for

Stereodirecting Effect of Esters at the 4-Position of Galacto- and Glucopyranosyl Donors. Effect of 4-*C*-Methylation on Side Chain Conformation and Donor Reactivity, and Influence of Concentration and Stoichiometry on Distal Group Participation

Chennaiah Ande[a] and David Crich[a,b,c,*]

a) Department of Pharmaceutical and Biomedical Sciences, University of Georgia, 250 West Green Street, Athens, GA 30602, USA

b) Department of Chemistry, University of Georgia, 302 East Campus Road, Athens, GA 30602, USA

c) Complex Carbohydrate Research Center, University of Georgia, 315 Riverbend Road, Athens, GA 30602, USA

David.Crich@uga.edu

Table S1. Contents

General Experimental	S3
Experimental Procedures and Characterization Data	S4-S31
References	S31
Copies of ^{1}H, ^{13}C, 2D NMR Spectra and UHPLC-MS Chromatograms	S32-S160

General Experimental

All reagents were purchased from commercial sources and used without further purification unless noted. All reactions were carried out under a positive pressure of argon or nitrogen. Solvents used for column chromatography were analytical grade and were purchased from commercial suppliers. Thin-layer chromatography was carried out with 250 µm glass backed silica (XHL) plates. Detection of compounds was achieved by UV absorption (254 nm) and by charring with a 20:80 v/v solution of sulfuric acid in ethanol or with a ceric ammonium molybdate solution. All organic solutions were concentrated at 30-45 ºC on a rotary evaporator. Purification of crude residues was performed by flash column chromatography using a COMBIFLASH® NextGen system, unless otherwise stated. Specific rotations were recorded on a Rudolph Research Analytical AUTOPOL® III automatic polarimeter in $CHCl_3$, MeOH or H_2O, at 589 nm and 23 ± 1 ºC with a path length of 10 cm. Nuclear magnetic resonance (NMR) spectra of all compounds were obtained in $CDCl_3$ (δ 7.27 and 77.1 ppm, respectively), D_2O (δ 4.79 ppm), or CD_3CN (δ 1.94 and 1.3 ppm, respectively), C_6D_6 (δ 7.16 and 128.1 ppm, respectively) at 500, 600 or 900 MHz. The chemical shifts (δ) are calculated with respect to residual solvent peak and are given in ppm. Multiplicities are abbreviated as follows: s (singlet), d (doublet), t (triplet), q (quartet), dd (doublet of doublet), bs (broad singlet) and m (multiplet). Peak assignments were based on two-dimensional NMR (COSY and HSQC) experiments, and the configurational or conformational assignments were determined with the aid of selective 1D NOESY and HMBC NMR experiments. High-resolution electrospray ionization (ESI) mass spectrometry spectra were recorded using a Thermo Scientific Orbitrap mass analyzer. UHPLC traces of crude reaction of competitive glycosylations were obtained using a ThermoFisher Vanquish UHPLC with PDA detector and an Acclaim 120 ^{18}C 4.6 x 50 mm column.

Experimental Procedures and Characterization Data

Synthesis of methyl 2,3-di-*O*-benzyl-4,6-*O*-benzylidene-6-(*S*)-deuterio-*α*-D-glucopyranoside (18-D_1)

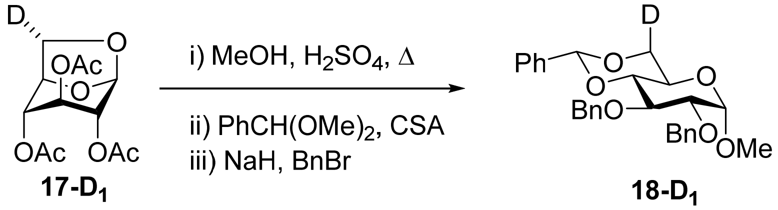

17-D_1 **18-D_1**

To a solution of 1,6-anhydro-2,3,4-tri-*O*-acetyl-6-(*S*)-deuterio-*β*-D-glucopyranose[1] **17-D_1** (0.33 g, 1.14 mmol) in MeOH (26 mL) at rt was added 2 drops of con. H_2SO_4. The solution was refluxed for 24 h, then the reaction was quenched by addition of triethylamine (5 mL). The solvent was evaporated under reduced pressure and the crude product was filtered through a short plug of silica gel, concentrated in *vacuo* and the obtained residue was subjected directly to the benzylidene acetal protection conditions. The acquired residue (0.15 g, 0.78 mmol) was dissolved in CH_3CN (1.5 mL) was treated with benzaldehyde dimethylacetal (129 μL, 0.86 mmol) followed by camphorsulfonic acid (0.046 g, 0.19 mmol) at rt. The reaction mixture was stirred for 2.5 h, then neutralized with triethylamine (0.5 mL), and concentrated under reduced pressure to afford the crude residue which was used without further purification. The crude residue (0.78 mmol) was dissolved in anhydrous DMF (1.5 mL) and cooled to 0 °C before 60% NaH in mineral oil (0.075 g, 3.13 mmol) was added followed by benzyl bromide (280 µL, 2.35 mmol). The reaction mixture was stirred at rt overnight, then cooled to 0 °C before quenching with water (1 mL). The reaction mixture was partitioned between ethyl acetate and water, the organic layer was separated, washed with brine, dried over anhydrous Na_2SO_4, and concentrated to dryness. The crude product was purified by column chromatography (2:8 ethyl acetate/hexanes) and given **18-D_1** (117 mg, 22% over three steps) as a colorless syrup.

R_f = 0.43 (2:8 EtOAc/hexanes)

$[\alpha]_D^{23}$ –24.3 (*c* 0.4, $CHCl_3$)

^{1}H NMR (500 MHz, $CDCl_3$): δ 7.52 – 7.50 (m, 2H, Ar-H), 7.43 – 7.27 (m, 13H, Ar-H), 5.58 (s, 1H, benzylidene C-H), 4.95 (d, *J* = 11.3 Hz, 1H, OCH_2Ar), 4.90 – 4.85 (m, 2H, OCH_2Ar), 4.73 (d, *J* = 12.2 Hz, 1H, OCH_2Ar), 4.63 (d, *J* = 3.9, 1H, H-1), 4.27 (d, *J* = 5.0 Hz, 1H, H-6), 4.08 (t, *J* = 9.3 Hz, 1H, H-3), 3.85 (dd, *J* = 9.6, 5.0 Hz, 1H, H-5), 3.63 (t, *J* = 9.4 Hz, 1H, H-4), 3.59 (dd, *J* = 9.4, 3.8 Hz, 1H, H-2), 3.43 (s, 3H, $-OCH_3$).

$^{13}C\{^1H\}$ NMR (126 MHz, $CDCl_3$): δ 138.9 (Ar), 138.3 (Ar), 137.5 (Ar), 129.0 (Ar), 128.6 (Ar), 128.4 (Ar), 128.3 (Ar), 128.2 (Ar), 128.1 (Ar), 128.0 (Ar), 127.7 (Ar), 126.1 (Ar), 101.4 (benzylidene-*C*), 99.4 (C-1), 82.2 (C-4), 79.3 (C-3), 78.7 (C-2), 75.4 (OCH_2Ar), 73.9 (OCH_2Ar), 68.8 (t, J_{C-D} = 21.7 Hz, C-6), 62.4 (C-5), 55.4 (-OCH_3).

HRMS–ESI (*m/z*): $[M + Na]^+$ calcd for $C_{28}H_{29}DNaO_6$, 486.1997; found, 486.1987.

Methyl 2,3,6-tri-*O*-benzyl-6-(*S*)-deuterio-*α*-D-glucopyranoside (19-D_1)

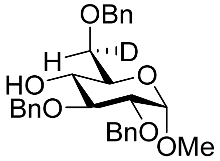

To a solution of compound **18-D_1** (0.117 g, 0.252 mmol) in CH_2Cl_2 (2.5 mL) at 0 °C was added Et_3SiH (0.20 mL, 1.26 mmol) followed by TFA (97 μL, 1.26 mmol) in a dropwise manner. The reaction stirred at 0 °C for 45 min and quenched with saturated aqueous $NaHCO_3$ solution (1 mL). The mixture was extracted with CH_2Cl_2 (2 X 10 mL); all combined organic phases were washed with brine, dried over Na_2SO_4, filtered, and evaporated under reduced pressure to give a crude residue. The crude residue was purified by column chromatography (1:1 EtOAc/hexanes) and to afford compound **19-D_1** (50 mg, 43%) as a colorless oil.

R_f = 0.6 (1:1 EtOAc/hexanes)

$[\alpha]_D^{23}$ –71.8 (*c* 0.3, $CHCl_3$)

^1H NMR (500 MHz, $CDCl_3$): δ 7.42 – 7.19 (m, 15H, Ar-H), 5.00 (d, *J* = 11.5 Hz, 1H, OCH_2Ar), 4.77 – 4.75 (m, 2H, OCH_2Ar), 4.70 – 4.62 (m, 2H, OCH_2Ar, H-1), 4.56 – 4.56 (m, 2H, OCH_2Ar), 3.78 (t, *J* = 9.2 Hz, 1H, H-3), 3.70 (dd, *J* = 9.7, 4.7 Hz, 1H, H-5), 3.65 (d, *J* = 4.6 Hz, 1H, H-6), 3.59 (t, *J* = 9.3 Hz, 1H, H-4), 3.53 (dd, *J* = 9.6, 3.5 Hz, 1H, H-2), 3.38 (s, 3H, -OCH_3), 2.32 (s, 1H, -OH).

$^{13}C\{^1H\}$ NMR (126 MHz, $CDCl_3$): δ 139.0 (Ar), 138.21 (Ar), 138.16 (Ar), 128.7 (Ar), 128.6 (Ar), 128.5 (Ar), 128.3 (Ar), 128.13 (Ar), 128.08 (Ar), 128.0 (Ar), 127.8 (Ar), 127.7 (Ar), 98.3 (C-1), 81.6 (C-3), 79.7 (C-2), 75.6 (OCH_2Ar), 73.7 (OCH_2Ar), 73.3 (CH_2Ph), 70.9 (C-4), 70.0 (C-5), 69.3 (t, J_{C-D} = 21.1 Hz, C-6), 55.4 (-OCH_3).

HRMS–ESI (*m/z*): $[M + Na]^+$ calcd for $C_{28}H_{31}DNaO_6$, 488.2154; found, 488.2154.

Synthesis of compounds 20-D_1 and 21-D_1

Compound **19-D_1** (0.037 g, 0.079 mmol) was dissolved in anhydrous solution of DMSO:CH_2Cl_2 2:1 (1 mL). The mixture was cooled to 0 °C and added diisopropylethylamine (69 μL, 0.39 mmol), sulfur trioxide pyridine complex (0.051 g, 0.31 mmol). The reaction mixture was allowed to gradually warm up to the rt and stirred until completion of the starting material **19-D_1** (detected by LCMS and TLC) (EtOAc/hexanes: 3:7). The reaction mixture was concentrated under reduced pressure and the

residue was diluted with Et_2O (10 mL) and water (10 mL). The aqueous layer was extracted with Et_2O twice and the combined organic layer was washed with saturated aqueous $NaHCO_3$ solution (10 mL), brine (10 mL), dried over Na_2SO_4, filtered, and concentrated to give the intermediate ketone as which was used in the next step without purification. MeMgCl (3 M in THF) (80 μL, 0.23 mmol) was added to a stirred solution of crude ketone in THF (0.7 mL) at 0 °C. After 1 h the reaction was quenched with 0.5 mL aqueous saturated NH_4Cl solution, diluted with Et_2O (5 mL), washed with aqueous NH_4Cl (5 mL) and brine (5 mL), dried with Na_2SO_4, and concentrated. The crude product was purified by flash column chromatography (3:7 EtOAc/hexanes) to give alcohols **20-D$_1$** (18 mg) and **21-D$_1$** (14 mg) (overall 84% over two steps) as a colorless syrups.

Methyl 2,3,6-tri-*O*-benzyl-4-*C*-methyl-6-(*S*)-deuterio-*α*-D-galactopyranoside (**20-D$_1$**)

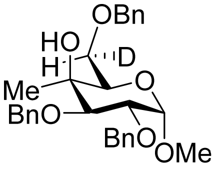

R$_f$ = 0.5 (3:7 EtOAc/hexanes)
[α]$_D^{23}$ +8.4 (*c* 0.1, $CHCl_3$)

^{1}H NMR (900 MHz, $CDCl_3$): δ 7.30 – 7.20 (m, 15H, Ar-H), 4.95 (d, *J* = 11.0 Hz, 1H, OCH_2Ar), 4.70 (d, *J* = 12.0 Hz, 1H, OCH_2Ar), 4.64 (d, *J* = 3.6 Hz, 1H, H-1), 4.58 – 4.56 (m, 2H, OCH_2Ar), 4.49 (s, 2H, OCH_2Ar), 3.83 (dd, *J* = 9.6, 3.7 Hz, 1H, H-2), 3.65 (d, *J* = 5.7 Hz, 1H, H-5), 3.62 (d, *J* = 5.7 Hz, 1H, H-6), 3.50 (d, *J* = 9.6 Hz, 1H, H-3), 3.34 (s, 3H, $-OCH_3$), 2.64 (s, 1H, OH), 1.11 (s, 3H, $-CH_3$).
^{13}C{^{1}H} NMR (226 MHz, $CDCl_3$): δ 138.5 (Ar), 138.1 (Ar), 128.58 (Ar), 128.55 (Ar), 128.5 (Ar), 128.4 (Ar), 128.3 (Ar), 128.0 (Ar), 127.9 (Ar), 127.85 (Ar), 127.80 (Ar), 98.4 (C-1), 80.7 (C-3), 78.1 (C-2), 76.4 (C-4), 74.3 (OCH_2Ar), 73.8 (OCH_2Ar), 73.4 (OCH_2Ar), 72.5 (C-5), 69.2 (t, J_{C-D} = 21.1 Hz, C-6), 55.5 ($-OCH_3$), 22.4 ($-CH_3$).
^{1}H NMR (600 MHz, C_6D_6): δ 7.37 – 7.21 (m, 6H, Ar-H), 7.16 – 7.05 (m, 9H, Ar-H), 4.99 (d, J = 11.1 Hz, 1H, OCH_2Ar), 4.81 (d, *J* = 3.5 Hz, 1H, H-1), 4.50 – 4.45 (m, 2H, OCH_2Ar), 4.45 – 4.40 (m, 2H, OCH_2Ar), 4.38 (d, *J* = 12.1 Hz, 1H, OCH_2Ar), 4.05 (dd, *J* = 9.6, 3.5 Hz, 1H, H-2), 3.92 (d, *J* = 6.2 Hz, 1H, H-5), 3.87 (d, *J* = 6.2 Hz, 1H, H-6), 3.72 (d, *J* = 9.6 Hz, 1H, H-3), 3.27 (s, 3H, $-OCH_3$), 1.10 (s, 3H, $-CH_3$).
^{13}C{^{1}H} NMR (151 MHz, C_6D_6): δ 139.4 (Ar), 139.2 (Ar), 139.1 (Ar), 98.3 (C-1), 80.8 (C-3), 79.1 (C-2), 76.2 (C-4), 74.0 (OCH_2Ar), 73.55 (C-5), 73.47 (OCH_2Ar), 72.6 (OCH_2Ar), 69.7 (t, J_{C-D} = 21.1 Hz, C-6), 55.0 ($-OCH_3$), 22.8 ($-CH_3$).
HRMS–ESI (*m/z*): $[M + Na]^+$ calcd for $C_{29}H_{33}DNaO_6$, 502.2310; found, 502.2296.

Methyl 2,3,6-tri-*O*-benzyl-4-*C*-methyl-6-(*S*)-deuterio-*α*-D-glucopyranoside (21-D$_1$)

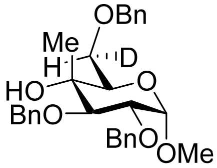

R$_f$ = 0.52 (3:7 EtOAc/hexanes)

[α]$_D^{23}$ +58.6 (*c* 0.1, $CHCl_3$)

^{1}H NMR (900 MHz, $CDCl_3$): δ 7.38 – 7.27 (m, 15H, Ar-H), 4.95 (d, *J* = 11.6 Hz, 1H, OCH_2Ar), 4.81 – 4.75 (m, 2H, OCH_2Ar), 4.62 (d, *J* = 12.1 Hz, 1H, OCH_2Ar), 4.59 (d, *J* = 3.8 Hz, 1H, H-1), 4.58 – 4.55 (m, 1H, OCH_2Ar), 4.52 (d, *J* = 12.0 Hz, 1H, OCH_2Ar), 3.88 (d, *J* = 6.9 Hz, 1H, H-5), 3.80 (d, *J* = 10.0 Hz, 1H, H-3), 3.55 (d, *J* = 7.0 Hz, 1H, H-6), 3.43 – 3.38 (m, 4H, $-OCH_3$, H-2), 2.49 (s, 1H, -OH), 1.17 (s, 3H, $-CH_3$).

^{13}C{^{1}H} NMR (226 MHz, $CDCl_3$): δ 139.3 (Ar), 138.4 (Ar), 138.0 (Ar), 128.58 (Ar), 128.55 (Ar), 128.5 (Ar), 128.2 (Ar), 128.0 (Ar), 127.92 (Ar), 127.87 (Ar), 127.7 (Ar), 98.2 (C-1), 83.7 (C-3), 78.7 (C-2), 75.8 (OCH_2Ar), 74.4 (C-4), 73.7 (OCH_2Ar), 73.5 (OCH_2Ar), 71.0 (C-5), 68.8 (t, $J_{C\text{-}D}$ = 21.1 Hz, C-6), 55.2 ($-OCH_3$), 16.0 ($-CH_3$).

HRMS–ESI (*m/z*): $[M + Na]^+$ calcd for $C_{29}H_{33}DNaO_6$, 502.2310; found, 502.2295.

General procedure for hydrogenolysis: To a solution of the corresponding methyl glycoside (1 equiv.) in EtOH (0.01 M) was added Pd/C (10% Pd, 1 equiv. by mass) followed by 1 N aqueous HCl (2 equiv.), the suspension was degassed and stirred vigorously under 1 atm of H_2 (balloon) for 14 h. The palladium on carbon was filtered through a syringe filter (0.22 μm) and the filtrate concentrated at reduced pressure give the deprotected substrates.

Methyl 4-*C*-methyl-6-(*S*)-deuterio-*α*-D-galactopyranoside (22-D$_1$)

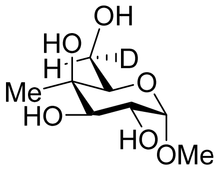

Prepared from compound **20-D$_1$** (5 mg, 0.01 mmol) with following quantities of reagents according to the general procedure for hydrogenolysis.

Pd/C (5 mg), 1 N aqueous HCl (20 μL, 0.02 mmol), EtOH (1 mL).
Obtained **22-D$_1$** (2 mg, 92%) as a colorless syrup.

[α]$_D^{23}$ +69.4 (*c* 0.2, MeOH)

^{1}H NMR (900 MHz, D_2O): δ 4.77 (d, *J* = 4.1 Hz, 1H, H-1), 3.69 (dd, *J* = 10.1, 4.0 Hz, 1H, H-2), 3.64 (d, *J* = 8.7 Hz, 1H, H-5), 3.62 (d, *J* = 8.7 Hz, 1H, H-6), 3.43 (d, *J* = 10.1 Hz, 1H, H-3), 3.34 (s, 3H, OCH_3), 1.16 (s, 3H, CH_3).
^{13}C{^{1}H} NMR (226 MHz, D_2O): δ 99.1 (C-1), 74.0 (C-5), 73.2 (C-4), 72.8 (C-3), 68.9 (C-2), 59.7 (t, $J_{C\text{-}D}$ = 21.4 Hz, C-6), 54.9 (-OCH_3), 19.7 (CH_3).
HRMS–ESI (*m/z*): $[M + Na]^+$ calcd for $C_8H_{15}DNaO_6$, 232.0901; found, 232.0902.

Methyl 4-*C*-methyl-6-(*S*)-deuterio-*α*-D-glucopyranoside (23-D$_1$)

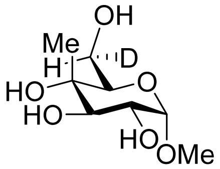

Prepared from compound **20-D$_1$** (3 mg, 0.006 mmol) with following quantities of reagents according to the general procedure for hydrogenolysis.
Pd/C (3 mg), 1 N aqueous HCl (13 μL, 0.012 mmol), EtOH (0.6 mL).
Obtained **23-D$_1$** (1.1 mg, 85%) as a colorless syrup.

$[\alpha]_D^{23}$ +42.1 (*c* 0.1, MeOH)

^{1}H NMR (900 MHz, D_2O): δ 4.74 (d, *J* = 4.1 Hz, 1H, H-1), 3.61 (d, *J* = 9.2 Hz, 1H, H-5), 3.59 – 3.56 (m, 2H, H-3, H-6), 3.51 (dd, *J* = 10.2, 4.1 Hz, 1H, H-2), 3.35 (s, 3H, OCH_3), 0.99 (s, 3H, CH_3).
^{13}C{^{1}H} NMR (226 MHz, D_2O): δ 98.9 (C-1), 75.3 (C-3), 74.4 (C-5), 72.9 (C-4), 70.1 (C-2), 59.3 (t, $J_{C\text{-}D}$ = 22.1 Hz, C-6), 54.8 (-OCH_3), 12.9 (CH_3).
HRMS–ESI (*m/z*): $[M + Na]^+$ calcd for $C_8H_{15}DNaO_6$, 232.0901; found, 232.0904.

Synthesis of compounds 20 and 21

Compound **19** (0.45 g, 0.97 mmol) was dissolved in anhydrous solution of DMSO:CH_2Cl_2 2:1 (8 mL). The mixture was cooled to 0 °C and added diisopropylethylamine (0.8 mL, 4.84 mmol), sulfur trioxide pyridine complex (0.617 g, 3.87 mmol). The reaction mixture was allowed to gradually warm up to the rt and stirred until completion of the starting material **19** (detected by LCMS and TLC) (EtOAc:hexane: 3:7, R_f =0.6). The reaction mixture was concentrated under reduced pressure and the residue diluted with Et_2O (30 mL) and water (30 mL). The aqueous layer was extracted with Et_2O twice and combined organic layer was washed with aqueous saturated $NaHCO_3$ (30 mL), brine (30 mL), dried over Na_2SO_4, filtered, and concentrated to give the intermediate ketone as which was used in the next step without purification. MeMgCl (3 M in THF) (1 mL, 2.90 mmol) was added to a stirred solution of ketone (0.97 mmol) in THF (8 mL) at 0 °C. After 1 h the reaction was quenched with 2 mL of aqueous saturated NH_4Cl solution, diluted with Et_2O (30 mL), washed with aqueous saturated NH_4Cl (30 mL) and brine (30 mL), dried with Na_2SO_4, and concentrated. The

crude product was purified by column chromatography (3:7 ethyl acetate/hexanes) and to give alcohols **20** (225 mg) and **21** (180 mg) (overall 87% over two steps) as a colorless syrups.

Methyl 2,3,6-tri-*O*-benzyl-4-*C*-methyl-*α*-D-galactopyranoside (**20**)

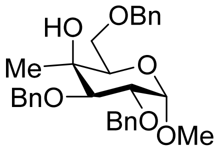

***R*$_f$** = 0.5 (3:7 EtOAc/hexanes)

$[\alpha]_D^{23}$ +45.0 (*c* 0.7, $CHCl_3$)

^{1}H NMR (500 MHz, $CDCl_3$): δ 7.38 – 7.25 (m, 15H, Ar-H), 5.02 (d, *J* = 11.0 Hz, 1H, OCH_2Ar), 4.77 (d, *J* = 12.0 Hz, 1H, OCH_2Ar), 4.71 (d, *J* = 3.7 Hz, 1H, H-1), 4.67 – 4.65 (m, 2H, OCH_2Ar), 4.56 (s, 2H, OCH_2Ar), 3.91 (dd, *J* = 9.6, 3.7 Hz, 1H, H-2), 3.82 (t, *J* = 7.4 Hz, 1H, H-6a), 3.71 (d, *J* = 8.8 Hz, 2H, H-5, H-6b), 3.58 (d, *J* = 9.6 Hz, 1H, H-3), 3.41 (s, 3H, $-OCH_3$), 2.71 (s, 1H, -OH), 1.18 (s, 3H, -CH_3).

^{13}C{^{1}H} NMR (126 MHz, $CDCl_3$): δ 138.4 (Ar), 138.0 (Ar), 128.54 (Ar), 128.50 (Ar), 128.47 (Ar), 128.4 (Ar), 128.2 (Ar), 128.0 (Ar), 127.9 (Ar), 127.8 (Ar), 127.7 (Ar), 98.3 (C-1), 80.7 (C-3), 78.1 (C-2), 76.3 (C-4), 74.2 (OCH_2Ar), 73.7 (OCH_2Ar), 73.3 (OCH_2Ar), 72.5 (C-5), 69.5 (C-6), 55.4 ($-OCH_3$), 22.3 ($-CH_3$).

^{1}H NMR (600 MHz, C_6D_6): δ 7.32 – 7.23 (m, 6H, Ar-H), 7.16 – 7.02 (m, 9H, Ar-H), 4.99 (d, *J* = 11.1 Hz, 1H, OCH_2Ar), 4.81 (d, *J* = 3.5 Hz, 1H, H-1), 4.49 (d, *J* = 11.0 Hz, 1H, OCH_2Ar), 4.47 – 4.41 (m, 3H, OCH_2Ar), 4.38 (d, *J* = 12.1 Hz, 1H, OCH_2Ar), 4.05 (dd, *J* = 9.6, 3.5 Hz, 1H, H-2), 3.95 (dd, *J* = 9.9, 2.6 Hz, 1H, H-6a), 3.93 – 3.91 (m, 1H, H-5), 3.88 (dd, *J* = 9.9, 5.9 Hz, 1H, H-6b), 3.71 (d, *J* = 9.6 Hz, 1H, H-3), 3.27 (s, 3H, $-OCH_3$), 2.45 (s, 1H, -OH), 1.10 (s, 3H, $-CH_3$).

^{13}C{^{1}H} NMR (151 MHz, C_6D_6): δ 139.4 (Ar), 139.2 (Ar), 139.1 (Ar), 98.3 (C-1), 80.8 (C-3), 79.1 (C-2), 76.3 (C-4), 74.1 (OCH_2Ar), 73.6 (C-5), 73.5 (OCH_2Ar), 72.6 (OCH_2Ar), 70.2 (C-6), 55.1 ($-OCH_3$), 22.8 ($-CH_3$).

HRMS–ESI (*m/z*): $[M + Na]^+$ calcd for $C_{29}H_{34}DNaO_6$, 501.2247; found, 501.2250.

Methyl 2,3,6-tri-*O*-benzyl-4-*C*-methyl-*α*-D-glucopyranoside (**21**)

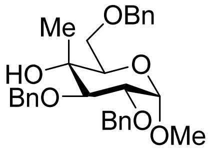

***R*$_f$** = 0.52 (3:7 EtOAc/hexanes)

[α]$_D^{23}$ +61.5 (*c* 0.1, $CHCl_3$)

^{1}H NMR (500 MHz, $CDCl_3$): δ 7.41 – 7.22 (m, 15H, Ar-H), 4.95 (d, *J* = 11.6 Hz, 1H, OCH_2Ar), 4.79 – 4.77 (m, 2H, OCH_2Ar), 4.63 (d, *J* = 12.1 Hz, 1H, OCH_2Ar), 4.60 (d, *J* = 3.8 Hz, 1H, H-1), 4.59 – 4.49 (m, 2H, OCH_2Ar), 3.90 (dd, *J* = 7.0, 5.1 Hz, 1H, H-5), 3.81 (d, *J* = 10.1 Hz, 1H, H-3), 3.72 (dd, *J* = 9.9, 5.1 Hz, 1H, H-6a), 3.58 (dd, *J* = 9.9, 7.0 Hz, 1H, H-6b), 3.44 (dd, *J* = 3.8, 10.1 Hz, 1H, H-2), 3.41 (s, 3H, $-OCH_3$), 2.42 (s, 1H, -OH), 1.18 (s, 3H, $-CH_3$).

^{13}C{^{1}H} NMR (126 MHz, $CDCl_3$): δ 139.3 (Ar), 138.4 (Ar), 137.9 (Ar), 128.54 (Ar), 128.52 (Ar), 128.51 (Ar), 128.1 (Ar), 127.92 (Ar), 127.87 (Ar), 127.8 (Ar), 127.7 (Ar), 98.1 (C-1), 83.6 (C-3), 78.7 (C-2), 75.8 (OCH_2Ar), 74.3 (C-4), 73.7 (OCH_2Ar), 73.4 (OCH_2Ar), 71.0 (C-5), 69.1 (C-6), 55.2 ($-OCH_3$), 15.9 ($-CH_3$).

HRMS–ESI (*m/z*): $[M + Na]^+$ calcd for $C_{29}H_{34}NaO_6$, 501.2247; found, 501.2250.

Methyl 4-*C*-methyl-*α*-D-galactopyranoside (22)

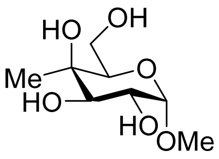

Prepared from compound **20** (8 mg, 0.02 mmol) with following quantities of reagents according to the general procedure for hydrogenolysis.

Pd/C (8 mg), 1 N aqueous HCl (17 μL, 0.02 mmol), EtOH (1.7 mL).
Obtained **22** (2.8 mg, 80%) as a colorless syrup.

[α]$_D^{23}$ +110.0 (*c* 0.3, MeOH)

^{1}H NMR (900 MHz, D_2O): δ 4.77 (d, *J* = 4.0 Hz, 1H, H-1), 3.84 (d, *J* = 8.7 Hz, 1H, H-6a), 3.69 (dd, *J* = 10.1, 4.1 Hz, 1H, H-2), 3.65 – 3.62 (m, 2H, H-5, H-6b), 3.43 (d, *J* = 10.0 Hz, 1H, H-3), 3.33 (s, 3H, $-OCH_3$), 1.15 (s, 3H, CH_3).

^{13}C{^{1}H} NMR (226 MHz, D_2O): δ 99.1 (C-1), 74.0 (C-5), 73.2 (C-4), 72.8 (C-3), 68.9 (C-2), 60.0 (C-6), 54.9 ($-OCH_3$), 19.7 (CH_3).

HRMS–ESI (*m/z*): $[M + Na]^+$ calcd for $C_8H_{16}NaO_6$, 231.0831; found, 231.0835.

Methyl 4-*C*-methyl-*α*-D-glucopyranoside (**23**)

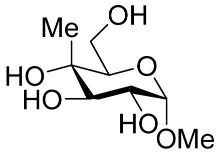

Prepared from compound **21** (5 mg, 0.01 mmol) with following quantities of reagents according to the general procedure for hydrogenolysis.

Pd/C (5 mg), 1 N aqueous HCl (10 μL, 0.01 mmol), EtOH (1 mL).
Obtained **23** (1.6 mg, 73%) as a colorless syrup.

$[\alpha]_D^{23}$ +117.2 (*c* 0.2, MeOH)

^{1}H NMR (900 MHz, D_2O): δ 4.79 (d, *J* = 4.1 Hz, 1H, H-1), 3.88 (dd, *J* = 11.6, 2.1 Hz, 1H, H-6a), 3.66 (dd, *J* = 9.2, 2.1 Hz, 1H, H-5), 3.65 – 3.62 (m, 2H, H-3, H-6b), 3.56 (dd, *J* = 10.3, 4.1 Hz, 1H, H-2), 3.39 (s, 3H, $-OCH_3$), 1.04 (s, 3H, CH_3).
^{13}C{^{1}H} NMR (226 MHz, D_2O): δ 98.9 (C-1), 75.3 (C-3), 74.4 (C-5), 72.9 (C-4), 70.1 (C-2), 59.6 (C-6), 54.8 ($-OCH_3$), 12.9 (CH_3).
HRMS–ESI (*m/z*): $[M + Na]^+$ calcd for $C_8H_{16}NaO_6$, 231.0831; found, 231.0832.

***p*-Methylphenyl 2,3,6-tri-*O*-benzyl-1-thio-*β*-D-glucopyranoside** (**27**)

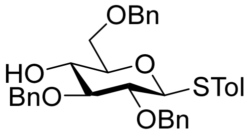

It was synthesized according to the literature procedure[2] from commercially available1,2,3,4,6-penta-*O*-acetyl-*β*-D-glucopyranose.

^{1}H NMR (500 MHz, $CDCl_3$): δ 7.53 – 7.48 (m, 2H, Ar-H), 7.47 – 7.43 (m, 2H, Ar-H), 7.42 – 7.28 (m, 13H, Ar-H), 7.12 – 7.03 (m, 2H, Ar-H), 4.95 – 4.93 (m, 2H, OCH_2Ar), 4.85 – 4.75 (m, 2H, OCH_2Ar), 4.66 (d, *J* = 9.6 Hz, 1H, H-1), 4.63 – 4.54 (m, 2H, OCH_2Ar), 3.81 (dd, *J* = 10.5, 4.0 Hz, 1H, H-6a), 3.78 (dd, *J* = 10.5, 5.2 Hz, 1H, H-6b), 3.67 (t, *J* = 9.2 Hz, 1H, H-4), 3.56 (t, *J* = 8.8 Hz, 1H, H-3), 3.52 – 3.45 (m, 2H, H-2, H-5), 2.59 (bs, 1H, -OH), 2.34 (s, 3H, CH_3).
^{13}C{^{1}H} NMR (126 MHz, $CDCl_3$): δ 138.6 (Ar), 138.2 (Ar), 138.1 (Ar), 137.8 (Ar), 132.7 (Ar), 129.9 (Ar), 129.8 (Ar), 128.7 (Ar), 128.6 (Ar), 128.57 (Ar), 128.53 (Ar), 128.4 (Ar), 128.3 (Ar), 128.03 (Ar), 128.0 (Ar), 127.98 (Ar), 127.8 (Ar), 127.79 (Ar), 88.0 (C-1), 86.3 (C-3), 80.6 (C-2), 78.2 (C-5), 75.6 (OCH_2Ar), 75.4 (OCH_2Ar), 73.7 (OCH_2Ar), 71.8 (C-4), 70.5 (C-6), 21.2 (CH_3).
HRMS–ESI (*m/z*): $[M + Na]^+$ calcd for $C_{34}H_{36}NaO_5S$, 579.2175; found, 579.2169.

Synthesis of compounds 3 and 28:

Compound **27** (0.70 g, 1.26 mmol) was dissolved in anhydrous solution of DMSO: CH_2Cl_2 2:1 (11 mL). The mixture was cooled to 0 °C and added diisopropylethylamine (1.1 mL, 6.30 mmol) sulfur trioxide pyridine complex (0.802 g, 5.04 mmol). The reaction mixture was allowed to gradually warm up to the rt and stirred until completion of the starting material **27** (detected by LCMS and TLC) (EtOAc:hexanes: 4:6, R_f =0.62). The reaction mixture was concentrated under reduced pressure and the residue diluted with Et_2O (40 mL) and water (40 mL). The aqueous layer was extracted with Et_2O twice and the combined organic layer was washed with aqueous saturated $NaHCO_3$ (40 mL), brine (40 mL), dried over Na_2SO_4, filtered, and concentrated to give the intermediate ketone as which was used in the next step without purification. MeMgCl (3 M in THF) (1.3 mL, 3.78 mmol) was added to a stirred solution of ketone (1.26 mmol) in THF (11 mL) at 0 °C. After 1 h the reaction was quenched with 4 mL aqueous saturated NH_4Cl solution, diluted with Et_2O (40 mL), washed with aqueous NH_4Cl (40 mL) and brine (40 mL), dried with Na_2SO_4, and concentrated to give the intermediate tertiary alcohol, which was used in the next step without purification. Benzoic acid (0.77 g, 6.30 mmol) and 1,1′- carbonyldiimidazole (0.97 g, 6.30 mmol) were dissolved in anhydrous THF (3 mL). The reaction mixture was stirred at rt for 1 hr. A solution of crude alcohol in THF (2 mL) was added in a dropwise manner, followed by addition of 1,8- diazabicyclo[5.4.0]undec-7-ene (DBU) (0.18 mL, 1.20 mmol) was done. The reaction was stirred for 48 h at 35 °C. After 48 h addition of DBU (0.18 mL, 1.20 mmol) was done which was repeated after 48 h. It was neutralized with aqueous saturated $NaHCO_3$ solution (5 mL), diluted with EtOAc (10 mL). Organic layer was separated out, it was collected whereas aqueous layer was extracted two times with EtOAc (2 × 10 ml). Organic layers were collected, dried over Na_2SO_4, filtered, and concentrated under reduced pressure to afford crude product, which was purified by column chromatography (1:9 EtOAc/hexanes) to give compounds **3** (527 mg) and **28** (151 mg) (overall 80% over three steps) as a white solids.

***p*-Methylphenyl 4-*O*-benzoyl-2,3,6-tri-*O*-benzyl-4-*C*-methyl-1-thio-*β*-D-galactopyranoside** (**3**)

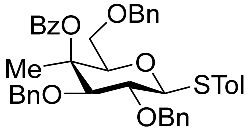

***R*$_f$** = 0.31 (1:9 EtOAc/hexanes)

^{1}H NMR (500 MHz, CDCl$_3$): δ 7.92 – 7.84 (m, 2H, Ar-H), 7.48 – 7.15 (m, 20H, Ar-H), 6.82 – 6.80 (m, 2H, Ar-H), 4.88 (d, *J* = 11.4 Hz, 1H, OCH_2Ar), 4.83 (d, *J* = 10.2 Hz, 1H, OCH_2Ar), 4.71 – 4.60 (m, 3H, OCH_2Ar, H-1), 4.51 (d, *J* = 11.6 Hz, 1H, OCH_2Ar), 4.45 (d, *J* = 11.6 Hz, 1H, OCH_2Ar), 4.06

(dd, J = 11.1, 2.4 Hz, 1H, H-6a), 3.81 (dd, J = 9.5, 8.9 Hz, 1H, H-2), 3.68 (dd, J = 11.1, 7.5 Hz, 1H, H-6b), 3.56 (dd, J = 7.5, 2.4 Hz, 1H, H-5), 3.33 (d, J = 8.9 Hz, 1H, H-3), 2.15 (s, 3H, ($ArCH_3$), 1.67 (s, 3H, CH_3).

$^{13}C\{^1H\}$ NMR (126 MHz, $CDCl_3$): δ 165.6 (C=O), 138.5 (Ar), 138.3 (Ar), 138.1 (Ar), 137.4 (Ar), 134.7 (Ar), 132.8 (Ar), 132.2 (Ar), 131.6 (Ar), 130.7 (Ar), 130.2 (Ar), 130.0 (Ar), 129.8 (Ar), 129.0 (Ar), 128.53 (Ar), 128.50 (Ar), 128.48 (Ar), 128.44 (Ar), 128.43 (Ar), 128.0 (Ar), 127.9 (Ar), 127.72 (Ar), 127.68 (Ar), 127.65 (Ar), 88.2 (C-1), 87.3 (C-3), 83.8 (C-4), 83.6 (C-5), 78.7 (C-2), 76.3 (OCH_2Ar), 75.5 (OCH_2Ar), 73.7 (OCH_2Ar), 70.5 (C-6), 21.2 ($ArCH_3$), 18.1 (CH_3).

HRMS–ESI (*m/z*): $[M + Na]^+$ calcd for $C_{42}H_{42}NaO_6S$, 697.2594; found, 697.2565.

***p*-Methylphenyl 4-*O*-benzoyl-2,3,6-tri-*O*-benzyl-4-*C*-methyl-1-thio-*β*-D-glucopyranoside (28)**

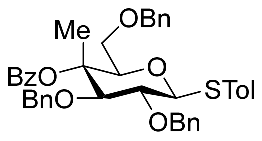

R_f = 0.41 (1:9 EtOAc/hexanes)

$[\alpha]_D^{23}$ –7.2 (*c* 0.9, $CHCl_3$)

^1H NMR (500 MHz, $CDCl_3$): δ 7.87 – 7.79 (m, 2H, Ar-H), 7.49 – 7.38 (m, 3H, Ar-H), 7.32 – 7.06 (m, 17H, Ar-H), 6.99 – 6.91 (m, 2H, Ar-H), 4.87 – 4.79 (m, 3H, H-3, H-5, OCH_2Ar), 4.76 (d, J = 10.0 Hz, 1H, H-1), 4.63 (m, 2H, OCH_2Ar), 4.50 (d, J = 11.0 Hz, 1H, OCH_2Ar), 4.42 – 4.34 (m, 2H, OCH_2Ar), 3.67 – 3.58 (m, 2H, H-6a, H-6b), 3.38 (dd, J = 10.0, 8.7 Hz, 1H, H-2), 2.22 (s, 3H, ($ArCH_3$), 1.41 (s, 3H, CH_3).

$^{13}C\{^1H\}$ NMR (126 MHz, $CDCl_3$): δ 165.7 (C=O), 138.37 (Ar), 138.35 (Ar), 138.2 (Ar), 137.8 (Ar), 134.7 (Ar), 133.2 (Ar), 132.5 (Ar), 130.71 (Ar), 130.69 (Ar), 130.0 (Ar), 129.83 (Ar), 129.81 (Ar), 129.0 (Ar), 128.48 (Ar), 128.45 (Ar), 128.3 (Ar), 128.1 (Ar), 127.91 (Ar), 127.86 (Ar), 127.7 (Ar), 127.5 (Ar), 88.2 (C-1), 84.5 (C-4), 82.7 (C-3), 81.1 (C-2), 77.4 (C-5), 75.5 (OCH_2Ar), 75.4 (OCH_2Ar), 73.4 (OCH_2Ar), 68.3 (C-6), 21.2 ($ArCH_3$), 15.3 (CH_3).

HRMS–ESI (*m/z*): $[M + Na]^+$ calcd for $C_{42}H_{42}NaO_6S$, 697.2594; found, 697.2578.

Synthesis of compounds 29 and 30

Compound **27** (0.67 g, 1.20 mmol) was dissolved in anhydrous solution of DMSO:CH_2Cl_2 2:1 (10 mL). The mixture was cooled to 0 °C and added diisopropylethylamine (1 mL, 6.01 mmol) sulfur trioxide pyridine complex (0.766 g, 4.81 mmol). The reaction mixture was allowed to gradually warm up to the rt and stirred until completion of the starting material **27** (detected by LCMS and TLC) (EtOAc:hexanes: 4:6, R_f =0.62). The reaction mixture was concentrated under reduced pressure and the residue diluted with Et_2O (30 mL) and water (30 mL). The aqueous layer was extracted

with Et_2O twice and the combined organic layer was washed with aqueous saturated $NaHCO_3$ (30 mL), brine (30 mL), dried over Na_2SO_4, filtered, and concentrated to give the intermediate ketone as which was used in the next step without purification. MeMgCl (3 M in THF) (1.2 mL, 3.61 mmol) was added to a stirred solution of ketone (1.20 mmol) in THF (10 mL) at 0 °C. After 1 h the reaction was quenched with 3 mL aqueous saturated NH_4Cl solution, diluted with Et_2O (30 mL), washed with aqueous saturated NH_4Cl (30 mL) and brine (30 mL), dried with Na_2SO_4, and concentrated to give the intermediate tertiary alcohol, which was used in the next step without purification. The crude residue was dissolved in anhydrous DMF (12 mL) and cooled to 0 °C before 60% NaH in mineral oil (0.058 g, 2.4 mmol) was added followed by benzyl bromide (285 µL, 2.4 mmol). The reaction mixture was stirred at rt overnight, then cooled to 0 °C before quenching with water (1 mL). The reaction mixture was partitioned between EtOAc and H_2O, the organic layer was separated, washed with brine, dried over anhydrous Na_2SO_4, and concentrated to dryness. The crude product was purified by column chromatography (1.5:8.5 EtOAc/hexanes) and to give compounds **29** (502 mg) and **30** (143 mg) (overall 81% over three steps) as a colorless syrups.

***p*-Methylphenyl 2,3,4,6-tetra-*O*-benzyl-4-*C*-methyl-1-thio-*β*-D-galactopyranoside** (**29**)

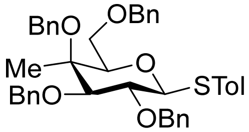

R_f = 0.4 (1.5:8.5 EtOAc/hexanes)
$[\alpha]_D^{23}$ +4.5 (*c* 0.4, $CHCl_3$)

^{1}H NMR (500 MHz, $CDCl_3$): δ 7.52 – 7.50 (m, 2H, Ar-H), 7.38 – 7.28 (m, 20H, Ar-H), 6.92 (d, *J* = 7.7 Hz, 2H, Ar-H), 5.00 (d, *J* = 11.3 Hz, 1H, OCH_2Ar), 4.95 (d, *J* = 11.9 Hz, 1H, OCH_2Ar), 4.84 (d, *J* = 10.1 Hz, 1H, OCH_2Ar), 4.72 – 4.58 (m, 5H, H-1, OCH_2Ar), 4.50 (d, *J* = 11.7 Hz, 1H, OCH_2Ar), 3.98 – 3.90 (m, 2H, H-2, H-6a), 3.79 (dd, *J* = 10.8, 6.2 Hz, 1H, H-6b), 3.53 (dd, *J* = 6.2, 3.6 Hz, 1H, H-5), 3.38 (d, *J* = 9.4 Hz, 1H, H-3), 2.28 (s, 3H, $ArCH_3$), 1.34 (s, 3H, -CH_3).
^{13}C{^{1}H} NMR (126 MHz, $CDCl_3$): δ 140.3 (Ar), 138.7 (Ar), 138.5 (Ar), 138.3 (Ar), 137.0 (Ar), 131.9 (Ar), 130.7 (Ar), 129.7 (Ar), 128.54 (Ar), 128.52 (Ar), 128.49 (Ar), 128.47 (Ar), 128.2 (Ar), 127.9 (Ar), 127.71 (Ar), 127.69 (Ar), 127.4 (Ar), 127.3 (Ar), 127.0 (Ar), 88.6 (C-3), 88.1 (C-1), 83.7 (C-5), 78.6 (C-2), 77.5 (C-4), 76.7 (OCH_2Ar), 75.5 (OCH_2Ar), 73.6 (OCH_2Ar), 69.9 (C-6), 66.9 (OCH_2Ar), 21.2 ($ArCH_3$), 16.9 (-CH_3).
HRMS–ESI (*m/z*): $[M + Na]^+$ calcd for $C_{42}H_{44}NaO_5S$, 683.2801; found, 683.2794.

***p*-Methylphenyl 2,3,4,6-tetra-*O*-benzyl-4-*C*-methyl-1-thio-*β*-D-glucopyranoside (30)**

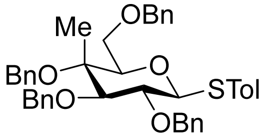

***R*$_f$** = 0.5 (1.5:8.5 EtOAc/hexanes)

[α]$_D^{23}$ +1.0 (*c* 0.4, $CHCl_3$)

^{1}H NMR (500 MHz, $CDCl_3$): δ 7.56 – 7.52 (m, 2H, Ar-H), 7.42 – 7.38 (m, 2H, Ar-H), 7.35 – 7.23 (m, 16H, Ar-H), 7.22 – 7.19 (m, 2H, Ar-H), 7.04 (d, *J* = 7.8 Hz, 2H, Ar-H), 4.97 – 4.92 (m, 2H, OCH_2Ar), 4.77 (d, *J* = 11.2 Hz, 1H, OCH_2Ar), 4.71 – 4.69 (m, 2H, H-1, OCH_2Ar), 4.65 (bs, 2H, OCH_2Ar), 4.59 (d, *J* = 11.9 Hz, 1H, OCH_2Ar), 4.52 (d, *J* = 11.9 Hz, 1H, OCH_2Ar), 3.94 (dd, *J* = 10.6, 2.1 Hz, 1H, H-6a), 3.82 (d, *J* = 8.9 Hz, 1H, H-3), 3.74 (dd, *J* = 7.6, 2.1 Hz, 1H, H-5), 3.66 (dd, *J* = 10.6, 7.6 Hz, 1H, H-6b), 3.45 (dd, *J* = 9.9, 8.9 Hz, 1H, H-2), 2.31 (s, 3H, $ArCH_3$), 1.35 (s, 3H, -CH_3).

^{13}C{^{1}H} NMR (126 MHz, $CDCl_3$): δ 139.0 (Ar), 138.8 (Ar), 138.6 (Ar), 138.2 (Ar), 137.7 (Ar), 132.3 (Ar), 130.3 (Ar), 129.8 (Ar), 128.54 (Ar), 128.50 (Ar), 128.48 (Ar), 128.46 (Ar), 128.3 (Ar), 128.0 (Ar), 127.8 (Ar), 127.7 (Ar), 127.5 (Ar), 127.4 (Ar), 127.2 (Ar), 88.2 (C-1), 87.3 (C-3), 81.7 (C-5), 81.0 (C-2), 77.9 (C-4), 75.6 (OCH_2Ar), 75.1 (OCH_2Ar), 73.5 (OCH_2Ar), 69.0 (C-6), 65.7 (OCH_2Ar), 21.2 (-$ArCH_3$), 13.3 (-CH_3).

HRMS–ESI (*m/z*): $[M + Na]^+$ calcd for $C_{42}H_{44}NaO_5S$, 683.2801; found, 683.2803.

General procedure for the competitive glycosylations

A mixture of 0.05 M solution of the glycosyl donor **A** (1.0 equiv.), glycosyl donor **B** (1.0 equiv.), diphenyl sulfoxide (1.0 equiv.), TTBP (1.5 equiv.), and 4 Å molecular sieves (200 mg; 2 g/mmol of the donor) in dichloromethane was stirred for 1 h at rt under argon then cooled to −78 °C and treated with Tf_2O (1.0 equiv.). The resulting mixture was stirred for 1 h before a solution of the acceptor (1.0 equiv.) in dichloromethane (0.02 M) was slowly added. The resulting mixture was stirred at −78 °C for 20 h. The reaction was quenched at −78 °C by adding Et_3N (0.5 mL). The temperature was allowed to warm to room temperature. The mixture was taken up in dichloromethane, filtered through a syringe filter (0.22 µm), washed with CH_2Cl_2. The organic phase was washed with aqueous saturated $NaHCO_3$, dried over Na_2SO_4, filtered, and concentrated to give crude product mixture. Conversion ratio was determined by total recovered donor mass and ultra-performance liquid chromatography (UPLC) of the crude product mixture.

General procedure for the glycosylations

A mixture of 0.05 M solution of the donor (1.0 equiv.), diphenyl sulfoxide (1.0 equiv.), TTBP (1.5 equiv.), and 4 Å molecular sieves (200 mg; 2 g/mmol of the donor) in dichloromethane was stirred

for 1 h at room temperature under argon then cooled to −78 °C and treated with Tf_2O (1.0 equiv.). The resulting mixture was stirred for 1 h before a solution of the acceptor (1.0 equiv.) in dichloromethane (0.02 M) was slowly added. The resulting mixture was stirred at −78 °C for 20 h. The reaction was quenched at −78 °C by adding Et_3N (0.5 mL). The temperature was allowed to warm to room temperature. The mixture was taken up in dichloromethane, filtered through a syringe filter (0.22 μm), washed with CH_2Cl_2. The organic phase was washed with aqueous saturated $NaHCO_3$, dried over Na_2SO_4, filtered, and concentrated to give crude product mixture. Purification of crude product by column chromatography afforded the corresponding glycopyranosides. The anomeric ratio of the products was determined by the integration of the ^{1}H NMR spectrum of the crude product mixture.

Synthesis of compounds 32α, 32β and 33

Compounds **32α**, **32β** (combined yield 34 mg, 54%; α/β = 1.0:0.8) and **33** (2.8 mg, 8%) were obtained from the reaction of donor **29** (51 mg, 0.077 mmol) and acceptor **31** (20 mg, 0.077 mmol) after column chromatography (1:9 EtOAc/hexanes) following the general procedure for glycosylation.

6-*O*-(2,3,4,6-tetra-*O*-benzyl-4-*C*-methyl-*α*-D-galactopyranosyl)-1,2:3,4-di-*O*-isopropylidene-*α*-D-galactopyranose (32α)

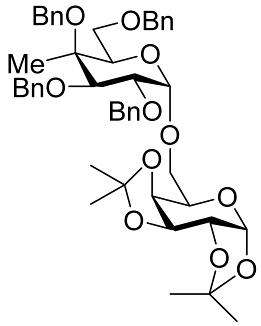

R_f = 0.20 (1:9 EtOAc/hexanes)

$[\alpha]_D^{23}$ +17.7 (*c* 0.4, $CHCl_3$)

Colorless syrup; **^{1}H NMR (500 MHz, $CDCl_3$):** δ 7.59 – 7.57 (m, 4H, Ar-H), 7.37 (m, 4H, Ar-H), 7.26 (m, 10H, Ar-H), 7.14 (m, 2H, Ar-H), 5.47 (d, *J* = 5.0 Hz, 1H, H-1'), 5.03 – 5.01 (m, 2H, OCH_2Ar, H-1), 4.83 (d, *J* = 12.0 Hz, 1H, OCH_2Ar), 4.67 (d, *J* = 11.9 Hz, 1H, OCH_2Ar), 4.59 – 4.50 (m, 5H, OCH_2Ar, H-3'), 4.40 (d, *J* = 12.0 Hz, 1H, OCH_2Ar), 4.28 – 4.22 (m, 2H, H-2', H-4'), 4.03 (td, *J* = 6.7, 2.1 Hz, 1H, H-5'), 3.95 (dd, *J* = 10.0, 3.6 Hz, 1H, H-2), 3.87 – 3.77 (m, 3H,H-5, H-6a, H-6'a), 3.73 (dd, *J* = 10.7, 6.9 Hz, 1H, H-6'b), 3.68 (d, *J* = 10.0 Hz, 1H, H-3), 3.53(dd, *J* = 10.8, 6.1 Hz, 1H, H-6b), 1.51 (s, 3H, CH_3), 1.49 (s, 3H, CH_3), 1.36 (s, 3H, CH_3), 1.27 (s, 3H, CH_3), 1.23 (s, 3H, CH_3).

$^{13}C\{^1H\}$ NMR (126 MHz, $CDCl_3$): δ 145.8 (Ar), 140.4 (Ar), 139.3 (Ar), 138.72 (Ar), 138.67 (Ar), 133.3 (Ar), 131.2 (Ar), 129.5 (Ar), 129.4 (Ar), 128.5 (Ar), 128.45 (Ar), 128.44 (Ar), 128.2 (Ar), 127.9 (Ar), 127.8 (Ar), 127.73 (Ar), 127.65 (Ar), 127.57 (Ar), 127.55 (Ar), 127.5 (Ar), 127.4 (Ar), 127.0 (Ar), 125.0 (Ar), 109.4 ($(CH_3)_2C$), 108.6 ($(CH_3)_2C$), 96.8 (C-1), 96.5 (C-1'), 82.9 (C-3), 78.3 (C-4), 77.5 (C-2), 76.6 (OCH_2Ar), 75.3 (C-5), 73.3 (OCH_2Ar), 72.3 (OCH_2Ar), 71.1 (C-3'), 70.9 (C-2'), 70.8 (C-4'), 69.8 (C-6), 67.1 (OCH_2Ar), 66.1 (C-6'), 65.8 (C-5'), 26.4 (CH_3), 26.2 (CH_3), 25.1 (CH_3), 24.8 (CH_3), 17.1 (CH_3).

HRMS–ESI (*m/z*): $[M + Na]^+$ calcd for $C_{47}H_{56}NaO_{11}$, 819.3714; found, 819.3718.

6-*O*-(2,3,4,6-tetra-*O*-benzyl-4-*C*-methyl-*β*-D-galactopyranosyl)-1,2:3,4-di-*O*-isopropylidene-*α*-D-galactopyranose (**32**β)

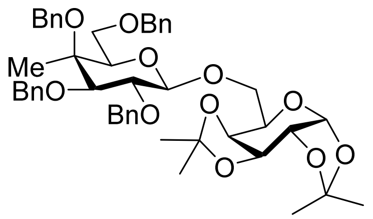

R$_f$ = 0.19 (1:9 EtOAc/hexanes)

$[\alpha]_D^{23}$ –11.5 (*c* 0.1, $CHCl_3$)

Colorless syrup; **^1H NMR (500 MHz, $CDCl_3$):** δ 7.42 – 7.28 (m, 12H, Ar-H), 7.24 – 7.15 (m, 8H, Ar-H), 5.57 (d, *J* = 5.1 Hz, 1H, H-1'), 5.05 (m, 2H, OCH_2Ar), 4.84 (d, *J* = 12.0 Hz, 1H, OCH_2Ar), 4.72 (d, *J* = 11.2 Hz, 1H, OCH_2Ar), 4.66 – 4.54 (m, 4H, OCH_2Ar, H-3'), 4.51 – 4.42 (m, 2H, OCH_2Ar, H-1), 4.30 (m, 1H, H-2'), 4.22 (m, 1H, H-4'), 4.17 (m, 1H, H-6a), 4.10 (d, *J* = 6.2 Hz, 1H, H-5), 3.90 (dd, *J* = 10.8, 2.2 Hz, 1H, H-6'a), 3.80 (t, *J* = 8.8 Hz, 1H, H-2), 3.77 – 3.71 (m, 1H, H-6b), 3.66 (dd, *J* = 11.7, 5.1 Hz, 1H, H-6'b), 3.43 (t, *J* = 4.9 Hz, 1H, H-5'), 3.25 (d, *J* = 10.0 Hz, 1H, H-3), 1.53 (s, 3H, CH_3), 1.49 (s, 3H, CH_3), 1.44 (s, 3H, CH_3), 1.30 (s, 3H, CH_3), 1.26 (s, 3H, CH_3).

$^{13}C\{^1H\}$ NMR (126 MHz, $CDCl_3$): δ 140.4 (Ar), 139.0 (Ar), 138.5 (Ar), 128.9 (Ar), 128.5 (Ar), 128.4 (Ar), 128.3 (Ar), 128.2 (Ar), 127.9 (Ar), 127.7 (Ar), 127.6 (Ar), 127.54 (Ar), 127.51 (Ar), 127.0 (Ar), 109.5 ($(CH_3)_2C$), 108.7 ($(CH_3)_2C$), 105.0 (C-1), 96.6 (C-1'), 86.2 (C-3), 79.73 (C-5'), 79.72 (C-2), 77.4 (C-4), 76.8 (OCH_2Ar), 74.6 (OCH_2Ar), 73.7 (OCH_2Ar), 71.7 (C-3), 71.0 (C-4'), 70.7 (C-2'), 70.0 (C-6), 69.9 (C-6'), 67.7 (OCH_2Ar), 67.2 (C-3), 26.20 (CH_3), 26.16 (CH_3), 25.2 (CH_3), 24.6 (CH_3), 17.2 (CH_3).

HRMS–ESI (*m/z*): $[M + Na]^+$ calcd for $C_{47}H_{56}NaO_{11}$, 819.3714; found, 819.3715.

1,4-Anhydro-2,3,6-tri-*O*-benzyl-4-*C*-methyl-*β*-D-galactopyranose (33)

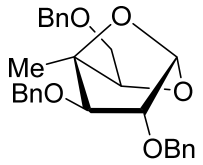

Rf = 0.40 (1.5:8.5 EtOAc/hexanes)

[α]$_D^{23}$ +43.8 (*c* 0.1, $CHCl_3$)

Colorless syrup; **^{1}H NMR (500 MHz, $CDCl_3$):** δ 7.28 – 7.20 (m, 15H, Ar-H), 5.32 (d, *J* = 2.4 Hz, 1H, H-1), 4.54 (d, *J* = 11.8 Hz, 1H, OCH_2Ar), 4.49 – 4.37 (m, 5H, OCH_2Ar), 3.77 (t, *J* = 2.0 Hz, 1H, H-2), 3.64 (dd, *J* = 7.5, 4.7 Hz, 1H, H-5), 3.40 (dd, *J* = 9.9, 7.5 Hz, 1H, H-6a), 3.35 – 3.30 (m, 2H, H-3, H-6b), 1.40 (s, 3H, CH_3).

^{13}C{^{1}H} NMR (126 MHz, $CDCl_3$): δ 138.0 (Ar), 137.9 (Ar), 137.7 (Ar), 128.7 (Ar), 128.6 (Ar), 128.2 (Ar), 128.0 (Ar), 127.9 (Ar), 127.8 (Ar), 98.0 (C-1), 88.4 (C-4), 88.1 (C-2), 84.6 (C-3), 77.0 (C-5), 73.6 (OCH_2Ar), 72.2 (OCH_2Ar), 71.3 (OCH_2Ar), 69.4 (C-6), 11.1 (CH_3).

HRMS–ESI (*m/z*): $[M + Na]^+$ calcd for $C_{28}H_{30}NaO_5$, 469.1985; found, 469.1979.

Synthesis of compounds 34α, 34β and 35

Compounds **34α**, **34β** (combined yield 36 mg, 58%; α/β = 1:1.1) and **35** (2 mg, 6%) were obtained from the reaction of donor **30** (51 mg, 0.077 mmol) and acceptor **31** (20 mg, 0.077 mmol) after column chromatography (1:9 EtOAc/hexanes) following the general procedure for glycosylation.

6-*O*-(2,3,4,6-tetra-*O*-benzyl-4-*C*-methyl-*α*-D-glucopyranosyl)-1,2:3,4-di-*O*-isopropylidene-*α*-D-galactopyranose (34α)

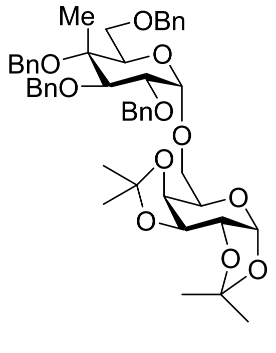

Rf = 0.20 (1:9 EtOAc/hexanes)

[α]$_D^{23}$ –19.0 (*c* 0.5, $CHCl_3$)

Colorless syrup; **^{1}H NMR (600 MHz, CD_3CN):** δ 7.37 – 7.23 (m, 20H, (Ar-H)), 5.49 (d, *J* = 5.0 Hz, 1H, H-1’), 5.03 (d, *J* = 3.8 Hz, 1H, H-1), 4.94 (d, *J* = 11.2 Hz, 1H, OCH_2Ar), 4.74 – 4.67 (m, 2H,

OCH$_2$Ar), 4.64 – 4.57 (m, 4H, OCH$_2$Ar, H-3'), 4.56 – 4.47 (m, 2H, OCH$_2$Ar), 4.35 (dd, *J* = 5.0, 2.5 Hz, 1H, H-2'), 4.27 (dd, *J* = 7.9, 2.0 Hz, 1H, H-4'), 4.13 – 4.10 (m, 1H, H-5), 4.08 – 4.04 (m, 1H, H-5'), 4.01 (d, *J* = 9.7 Hz, 1H, H-3), 3.80 (ddd, *J* = 10.5, 4.3, 2.7 Hz, 2H, H-6a, H-6'a), 3.66 (dd, *J* = 10.5, 5.7 Hz, 1H, H-6'b), 3.59 (dd, *J* = 9.7, 3.8 Hz, 1H, H-2), 3.52 (dd, *J* = 10.7, 8.0 Hz, 1H, H-6b), 1.50 (s, 3H, CH$_3$), 1.36 (s, 3H, CH$_3$), 1.30 (s, 3H, CH$_3$), 1.29 (s, 3H, CH$_3$), 1.21 (s, 3H, CH$_3$).

^{13}C{^{1}H} NMR (151 MHz, CD$_3$CN): δ 140.3 (Ar), 140.2 (Ar), 139.79 (Ar), 139.77 (Ar), 129.3 (Ar), 129.22 (Ar), 129.18 (Ar), 129.16 (Ar), 128.8 (Ar), 128.5 (Ar), 128.48 (Ar), 128.44 (Ar), 128.40 (Ar), 128.34 (Ar), 128.29 (Ar), 128.2 (Ar), 109.9 ((CH$_3$)$_2$C), 109.3 ((CH$_3$)$_2$C), 97.3 (C-1'), 96.5 (C-1), 81.9 (C-3), 80.6 (C-2), 78.8 (C-4), 75.5 (OCH$_2$Ar), 73.4 (OCH$_2$Ar), 72.5 (OCH$_2$Ar), 72.1 (C-5), 72.0 (C-4'), 71.6 (C-3'), 71.4 (C-2'), 69.5 (C-6), 67.0 (C-5'), 66.7 (C-6'), 66.1 (OCH$_2$Ar), 26.38 (CH$_3$), 26.36 (CH$_3$), 25.2 (CH$_3$), 24.8 (CH$_3$), 13.5 (CH$_3$).

HRMS–ESI (*m/z*): [M + Na]$^+$ calcd for $C_{47}H_{56}NaO_{11}$, 819.3714; found, 819.3709.

6-*O*-(2,3,4,6-tetra-*O*-benzyl-4-*C*-methyl-*β*-D-glucopyranosyl)-1,2:3,4-di-*O*-isopropylidene-*α*-D-galactopyranose (34β)

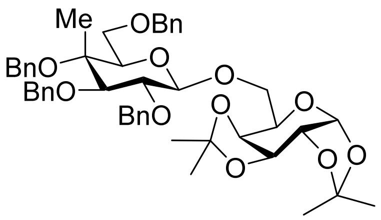

R$_f$ = 0.19 (1:9 EtOAc/hexanes)

[α]$_D^{23}$ +41.5 (*c* 0.1, CHCl$_3$)

Colorless syrup; **^{1}H NMR (500 MHz, CDCl$_3$):** δ 7.29 – 7.11 (m, 18H, Ar-H), 7.12 – 6.99 (m, 2H, Ar-H), 5.50 (d, *J* = 5.0 Hz, 1H, H-1'), 4.96 – 4.94 (m, 2H, OCH$_2$Ar), 4.62 (d, *J* = 11.4 Hz, 1H, OCH$_2$Ar), 4.60 – 4.52 (m, 4H, OCH$_2$Ar), 4.50 (dd, *J* = 7.9, 2.4 Hz, 1H, H-3'), 4.47 – 4.43 (m, 2H, H-1, OCH$_2$Ar), 4.24 (dd, *J* = 5.0, 2.4 Hz, 1H, H-2'), 4.17 (dd, *J* = 7.9, 1.9 Hz, 1H, H-4'), 4.13 – 4.08 (m, 1H, H-6'a), 4.05 (ddd, *J* = 7.5, 3.7, 1.9 Hz, 1H, H-5'), 3.83 (dd, *J* = 10.6, 1.5 Hz, 1H, H-6a), 3.76 (dd, *J* = 10.9, 7.5 Hz, 1H, H-6'b), 3.63 (d, *J* = 9.4 Hz, 1H, H-3), 3.58 (dd, *J* = 7.7, 1.5 Hz, 1H, H-5), 3.51 (dd, *J* = 10.5, 7.7 Hz, 1H, H-6b), 3.35 (dd, *J* = 9.4, 7.8 Hz, 1H, H-2), 1.44 (s, 3H, CH$_3$), 1.38 (s, 3H, CH$_3$), 1.25 (s, 3H, CH$_3$), 1.24 (s, 6H, CH$_3$).

^{13}C{^{1}H} NMR (126 MHz, CDCl$_3$): δ 139.2 (Ar), 139.1 (Ar), 138.9 (Ar), 138.5 (Ar), 128.7 (Ar), 128.5 (Ar), 128.43 (Ar), 128.40 (Ar), 128.3 (Ar), 127.8 (Ar), 127.7 (Ar), 127.6 (Ar), 127.5 (Ar), 127.4 (Ar), 127.2 (Ar), 109.5 ((CH$_3$)$_2$C), 108.7 ((CH$_3$)$_2$C), 105.1 (C-1), 96.5 (C-1'), 85.3 (C-3), 81.9 (C-2), 77.9 (C-4), 77.5 (C-5), 75.1 (OCH$_2$Ar), 74.7 (OCH$_2$Ar), 73.5 (OCH$_2$Ar), 71.6 (C-4'), 70.9 (C-2'), 70.7 (C-

3’), 70.3 (C-6’), 68.9 (C-6), 67.7 (C-5’), 65.9 (OCH_2Ar), 26.21 (CH_3), 26.15 (CH_3), 25.2 (CH_3), 24.6 (CH_3), 13.1 (CH_3).

HRMS–ESI (*m/z*): $[M + Na]^+$ calcd for $C_{47}H_{56}NaO_{11}$, 819.3714; found, 819.3691.

1,6-Anhydro-2,3,4-tri-*O*-benzyl-4-*C*-methyl-*β*-D-glucopyranose (35)

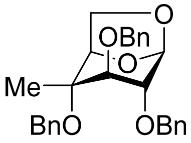

R_f = 0.33 (1.5:8.5 EtOAc/hexanes)

$[\alpha]_D^{23}$ +7.1 (*c* 0.1, $CHCl_3$)

Colorless syrup; **^{1}H NMR (500 MHz, $CDCl_3$):** δ 7.43 – 7.29 (m, 15H, Ar-H), 5.57 (s, 1H, H-1), 4.65 (d, *J* = 11.1 Hz, 1H, OCH_2Ar), 4.61 – 4.52 (m, 4H, OCH_2Ar), 4.50 (d, *J* = 5.8 Hz, 1H, H-5), 4.44 (d, *J* = 11.8 Hz, 1H, OCH_2Ar), 4.18 (d, *J* = 7.2 Hz, 1H, H-6a), 3.75 (dd, *J* = 7.3, 5.7 Hz, 1H, H-6b), 3.64 (d, *J* = 1.7 Hz, 1H, H-3), 3.45 (t, *J* = 1.8 Hz, 1H, H-2), 1.30 (s, 3H, CH_3).

$^{13}C\{^1H\}$ NMR (126 MHz, $CDCl_3$): δ 139.1 (Ar), 138.1 (Ar), 138.0 (Ar), 128.5 (Ar), 128.47 (Ar), 128.3 (Ar), 128.0 (Ar), 127.9 (Ar), 127.8 (Ar), 127.5 (Ar), 127.3 (Ar), 100.7 (C-1), 77.7 (C-3), 77.3 (C-4), 75.9 (C-5), 75.8 (C-2), 72.9 (OCH_2Ar), 72.0 (OCH_2Ar), 64.4 (OCH_2Ar), 64.2 (C-6), 17.8 (CH_3).

HRMS–ESI (*m/z*): $[M + Na]^+$ calcd for $C_{28}H_{30}NaO_5$, 469.1985; found, 469.1990.

Synthesis of compound (36α)

Compound **36α** (78 mg, 65%) was obtained from the reaction of donor **3** (100 mg, 0.148 mmol) and acceptor **31** (39 mg, 0.148 mmol) after column chromatography (2:8 EtOAc/hexanes) following the general procedure for glycosylation.

6-*O*-(4-*O*-benzoyl-2,3,6-tri-*O*-benzyl-4-*C*-methyl-*α*-D-galactopyranosyl)-1,2:3,4-di-*O*-isopropylidene-*α*-D-galactopyranose (36α)

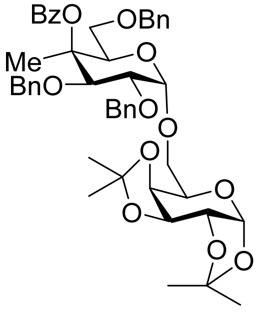

R_f = 0.28 (2:8 EtOAc/hexanes)

Colorless syrup; **^{1}H NMR (500 MHz, $CDCl_3$):** δ 7.94 – 7.90 (m, 2H, Ar-H), 7.52 – 7.50 (m, 1H, Ar-H), 7.38 – 7.23 (m, 17H, Ar-H), 5.57 (d, *J* = 5.0 Hz, 1H, H-1'), 5.16 (d, *J* = 3.7 Hz, 1H, H-1), 5.06 (d, *J* = 11.5 Hz, 1H, OCH_2Ar), 4.78 (dd, *J* = 11.7, 8.0 Hz, 2H, OCH_2Ar), 4.69 (d, *J* = 12.0 Hz, 1H, OCH_2Ar), 4.63 – 4.57 (m, 2H, OCH_2Ar, H-3'), 4.53 (d, *J* = 12.1 Hz, 1H, OCH_2Ar), 4.36 – 4.31 (m, 2H, H-2', H-4'), 4.13 (td, *J* = 6.7, 1.9 Hz, 1H, H-5'), 4.10 – 4.03 (m, 3H, H-2, H-5, H-6a), 3.97 (dd, *J* = 10.7, 6.8 Hz, 1H, H-6'a), 3.84 (dd, *J* = 10.7, 6.7 Hz, 1H, H-6'b), 3.78 (d, *J* = 9.6 Hz, 1H, H-3), 3.62 (dd, *J* = 10.9, 7.3 Hz, 1H, H-6b), 1.79 (s, 3H, CH_3), 1.57 (s, 3H, CH_3), 1.45 (s, 3H, CH_3), 1.35 (s, 3H, CH_3), 1.33 (s, 3H, CH_3).

$^{13}C\{^1H\}$ NMR (126 MHz, $CDCl_3$): δ 165.5 (C=O), 138.8 (Ar), 138.5 (Ar), 138.4 (Ar), 132.7 (Ar), 131.8 (Ar), 129.8 (Ar), 128.4 (Ar), 128.34 (Ar), 128.28 (Ar), 128.1 (Ar), 127.7 (Ar), 127.6 (Ar), 127.53 (Ar), 127.47 (Ar), 127.4 (Ar), 109.4 ($(CH_3)_2C$), 108.6 ($(CH_3)_2C$), 96.5 (C-1), 96.3 (C-1'), 84.9 (C-4), 81.7 (C-3), 77.4 (C-2), 76.5 (C-5), 76.1 (OCH_2Ar), 74.3 (C-5), 73.3 (OCH_2Ar), 72.1 (OCH_2Ar), 71.1 (C-4'), 70.9 (C-2'), 70.7 (C-3'), 69.9 (C-6), 66.0 (C-6'), 65.7 (C-5'), 26.3 (CH_3), 26.2 (CH_3), 25.1 (CH_3), 24.8 (CH_3), 18.5 (CH_3).

HRMS–ESI (*m/z*): $[M + Na]^+$ calcd for $C_{47}H_{54}NaO_{12}$, 833.3507; found, 833.3487.

Synthesis of compounds 37β, 38, 39 and 40:

Compounds **37**β (62 mg, 52%), **38** (5 mg, 7%), **39** (2.5 mg, 3%), **40** (3 mg, 5%) was obtained from the reaction of donor **28** (100 mg, 0.148 mmol) and acceptor **31** (39 mg, 0.148 mmol) after column chromatography (2:8 EtOAc/hexanes) following the general procedure for glycosylation.

6-*O*-(4-*O*-benzoyl-2,3,6-tri-*O*-benzyl-4-*C*-methyl-*β*-D-glucopyranosyl)-1,2:3,4-di-*O*-isopropylidene-*α*-D-galactopyranose (**37**β)

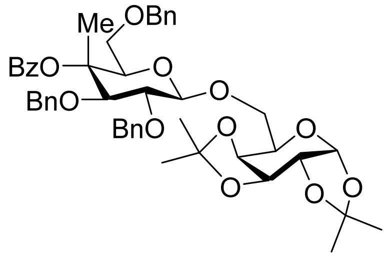

R_f = 0.23 (2:8 EtOAc/hexanes)

$[\alpha]_D^{23}$ +3.0 (*c* 0.1, $CHCl_3$)

Colorless syrup; **^{1}H NMR (500 MHz, $CDCl_3$):** δ 7.92 – 7.87 (m, 2H, Ar-H), 7.55 – 7.51 (m, 1H, Ar-H), 7.42 – 7.36 (m, 4H, Ar-H), 7.29 – 7.24 (m, 3H, Ar-H), 7.22 – 7.11 (m, 10H, Ar-H), 5.57 (d, *J* = 5.0 Hz, 1H, H-1'), 5.04 (d, *J* = 11.1 Hz, 1H, OCH_2Ar), 4.87 (dd, *J* = 7.1, 3.1 Hz, 1H, H-5), 4.81 (d, *J* = 9.2 Hz, 1H, H-3), 4.76 (d, *J* = 11.2 Hz, 1H, OCH_2Ar), 4.70 – 4.66 (m, 2H, OCH_2Ar, H-1), 4.58 – 4.54 (m, 2H, OCH_2Ar, H-3'), 4.53 – 4.44 (m, 2H, OCH_2Ar), 4.32 (dd, *J* = 5.0, 2.4 Hz, 1H, H-2'), 4.23

(dd, J = 7.9, 1.9 Hz, 1H, H-4'), 4.18 – 4.10 (m, 2H, H-5', H-6'a), 3.85 (dd, J = 10.6, 7.1 Hz, 1H, H-6'b), 3.71 (dd, J = 11.1, 3.1 Hz, 1H, H-6a), 3.63 (dd, J = 11.0, 7.0 Hz, 1H, H-6b), 3.46 (dd, J = 9.2, 7.9 Hz, 1H, H-2), 1.53 (s, 3H, CH_3), 1.50 (s, 3H, CH_3), 1.46 (s, 3H, CH_3), 1.32 (s, 3H, CH_3), 1.31 (s, 3H, CH_3).

$^{13}C\{^1H\}$ NMR (126 MHz, CDCl$_3$): δ 165.6 (C=O), 139.0 (Ar), 138.7 (Ar), 138.1 (Ar), 133.0 (Ar), 130.8 (Ar), 129.8 (Ar), 128.4 (Ar), 128.37 (Ar), 128.35 (Ar), 128.32 (Ar), 128.2 (Ar), 127.8 (Ar), 127.6 (Ar), 127.52 (Ar), 127.46 (Ar), 127.4 (Ar), 109.5 (($CH_3)_2C$), 108.7 (($CH_3)_2C$), 105.1 (C-1), 96.5 (C-1'), 84.7 (C-4), 82.1 (C-2), 80.2 (C-3), 75.3 (OCH_2Ar), 74.6 (OCH_2Ar), 73.3 (OCH_2Ar), 72.8 (C-5), 71.5 (C-4'), 70.9 (C-2'), 70.6 (C-3'), 70.1 (C-6'), 68.2 (C-5'), 67.5 (C-6), 26.2 (CH_3), 26.1 (CH_3), 25.1 (CH_3), 24.6 (CH_3), 15.3 (CH_3).

HRMS–ESI (*m/z*): $[M + Na]^+$ calcd for $C_{47}H_{54}NaO_{12}$, 833.3507; found, 833.3503.

1,6-Anhydro-4-*O*-benzoyl-2,3-di-*O*-benzyl-4-*C*-methyl-*β*-D-glucopyranose (**38**)

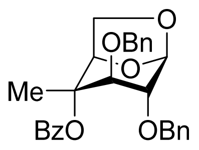

R_f = 0.31 (1:9 EtOAc/hexanes)

$[\alpha]_D^{23}$ –15.3 (*c* 0.1, CHCl$_3$)

Colorless syrup; **^1H NMR (500 MHz, CDCl$_3$):** δ 8.02 – 7.95 (m, 2H, Ar-H), 7.47 – 7.45 (m, 1H, Ar-H), 7.39 – 7.28 (m, 5H, Ar-H), 7.26 – 7.21 (m, 7H, Ar-H), 5.54 (d, J = 1.7 Hz, 1H, H-1), 5.09 (dd, J = 4.5, 1.5 Hz, 1H, H-5), 4.62 – 4.54 (m, 2H, OCH_2Ar), 4.48 – 4.46 (m, 2H, OCH_2Ar), 4.24 (dd, J = 7.4, 1.1 Hz, 1H, H-6a), 4.00 (d, J = 1.6 Hz, 1H, H-3), 3.75 (dd, J = 7.5, 5.9 Hz, 1H, H-6b), 3.44 (t, J = 1.6 Hz, 1H, H-2), 1.70 (s, 3H, CH_3).

$^{13}C\{^1H\}$ NMR (126 MHz, CDCl$_3$): δ 166.0 (C=O), 137.72 (Ar), 137.67 (Ar), 132.8 (Ar), 131.4 (Ar), 129.9 (Ar), 128.6 (Ar), 128.5 (Ar), 128.3 (Ar), 128.03 (Ar), 128.0 (Ar), 127.9 (Ar), 100.5 (C-1), 80.9 (C-4), 77.3 (C-3), 76.1 (C-5), 75.4 (C-2), 73.6 (OCH_2Ar), 72.2 (OCH_2Ar), 64.3 (C-6), 18.1 (CH_3).

HRMS–ESI (*m/z*): $[M + Na]^+$ calcd for $C_{28}H_{28}NaO_6$, 483.1778; found, 483.1770.

4-*O*-benzoyl-2,3,6-tri-*O*-benzyl-4-*C*-methyl-*α/β*-D-glucopyranose (**39**)

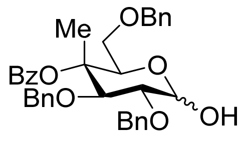

(α:β mixture of anomers ~1:1)

R_f = 0.26 (2:8 EtOAc/hexanes)

^1H NMR (500 MHz, CDCl$_3$): δ 7.91 – 7.88 (m, 3H, Ar-H), 7.58 – 7.49 (m, 2H, Ar-H), 7.47 – 7.43 (m, 1H, Ar-H), 7.40 – 7.35 (m, 4H, Ar-H), 7.35 – 7.28 (m, 10H, Ar-H), 7.21 – 7.12 (m, 20H, Ar-H), 5.35

(dd, J = 7.3, 2.9 Hz, 1H, H-5α), 5.24 (d, J = 3.9 Hz, 1H, H-1α), 5.06 (d, J = 9.4 Hz, 1H, H-3α), 4.93 – 4.90 (m, 3H, H-1β, H-5β, OCH_2Ar), 4.83 (d, J = 9.2 Hz, 1H, H-3β), 4.77 – 4.70 (m, 4H, OCH_2Ar), 4.66 (d, J = 11.7 Hz, 1H, OCH_2Ar), 4.57 – 4.55 (m, 2H, OCH_2Ar), 4.52 – 4.45 (m, 4H, OCH_2Ar), 3.67 – 3.54 (m, 5H, H-6α-a, H-6α-b, H-6β-a, H-6β-b, H-2α,) 3.41 (dd, J = 9.2, 7.8 Hz, 1H, H-2β), 1.50 (s, 3H, CH_3), 1.48 (s, 3H, CH_3).

$^{13}C\{^1H\}$ NMR (126 MHz, $CDCl_3$): δ 165.6 (C=O), 165.5 (C=O), 138.6 (Ar), 138.0 (Ar), 133.2 (Ar), 133.0 (Ar), 129.8 (Ar), 129.4 (Ar), 128.6 (Ar), 128.4 (Ar), 128.3 (Ar), 128.2 (Ar), 128.0 (Ar), 127.9 (Ar), 127.8 (Ar), 127.7 (Ar), 127.6 (Ar), 127.5 (Ar), 98.0 (C-1β), 91.2 (C-1α), 84.7 (C-4β), 84.6 (C-4α), 83.3 (C-2β), 80.2 (C-3β), 79.8 (C-2α), 78.1 (C-3α), 75.31 (OCH_2Ar), 75.26 (OCH_2Ar), 75.0 (OCH_2Ar), 73.5 (OCH_2Ar), 73.4 (OCH_2Ar), 73.2 (C-5β), 72.9 (C-5α), 68.0 (C-6β), 67.9 (C-6α), 15.3 (CH_3), 14.7 (CH_3).

HRMS–ESI (*m/z*): $[M + Na]^+$ calcd for $C_{35}H_{36}NaO_7$, 591.2353; found, 591.2338.

1,2:3,4-Di-*O*-isopropylidene-6-*O*-trifluoromethanesulfonyl-*α*-D-galactopyranose (**40**)

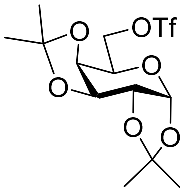

R_f = 0.53 (2:8 EtOAc/hexanes)

^1H NMR (500 MHz, $CDCl_3$): δ 5.53 (d, J = 4.9 Hz, 1H, H-1), 4.66 – 4.55 (m, 3H, H-3, H-6a, H-6b), 4.35 (dd, J = 5.0, 2.6 Hz, 1H, H-2), 4.23 (dd, J = 7.8, 2.0 Hz, 1H, H-4), 4.10 (ddd, J = 7.0, 4.6, 2.0 Hz, 1H, H-5), 1.52 (s, 3H, CH_3), 1.44 (s, 3H, CH_3), 1.33 (s, 3H, CH_3), 1.32 (s, 3H, CH_3).

$^{13}C\{^1H\}$ NMR (126 MHz, $CDCl_3$): δ 110.2 ($(CH_3)_2C$), 109.2 ($(CH_3)_2C$), 96.2 (C-1), 74.7 (C-6), 70.7 (C-3), 70.5 (C-4), 70.3 (C-2), 66.1 (C-5), 26.0 (CH_3), 25.9 (CH_3), 24.9 (CH_3), 24.5 (CH_3).

^{19}F NMR (470 MHz, $CDCl_3$): δ -74.3.

HRMS–ESI (*m/z*): $[M + Na]^+$ calcd for $C_{13}H_{19}NaO_8F_3S$, 415.0649; found, 415.0644.

Synthesis of compounds 41α and 41β

Compounds **41α** and **41β** (combined yield 32 mg, 53%; α/β = 1:0.7) were obtained from the reaction of donor **4** (50 mg, 0.075 mmol) and acceptor **31** (20 mg, 0.075 mmol) after column chromatography (2:8 EtOAc/hexanes) following the general procedure for glycosylation.

6-*O*-(4-*O*-benzoyl-2,3,6-tri-*O*-benzyl-*α*-D-galactopyranosyl)-1,2:3,4-di-*O*-isopropylidene-*α*-D-galactopyranose (**41**α)

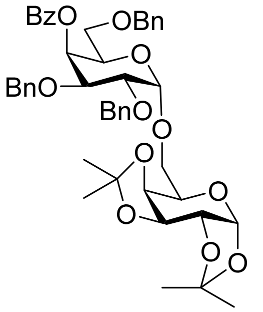

R_f = 0.42 (2:8 EtOAc/hexanes)

Colorless syrup; **^{1}H NMR (500 MHz, $CDCl_3$):** δ 8.01 – 7.99 (m, 2H, Ar-H), 7.55 (m, 1H, Ar-H), 7.43 – 7.40 (m, 2H, Ar-H), 7.32 – 7.18 (m, 15H, Ar-H), 5.88 (dd, *J* = 3.4, 1.3 Hz, 1H, H-4), 5.51 (d, *J* = 5.0 Hz, 1H, H-1'), 5.05 (d, *J* = 3.7 Hz, 1H, H-1), 4.85 (d, *J* = 11.2 Hz, 1H, OCH_2Ar), 4.76 (d, *J* = 11.9 Hz, 1H, OCH_2Ar), 4.69 (d, *J* = 11.9 Hz, 1H, OCH_2Ar), 4.58 – 4.55 (m, 2H, OCH_2Ar, H-3'), 4.49 (d, *J* = 11.9 Hz, 1H, OCH_2Ar), 4.41 (d, *J* = 11.9 Hz, 1H, OCH_2Ar), 4.34 – 4.28 (m, 3H, H-5, H-4', H-2'), 4.08 (dd, *J* = 10.0, 3.3 Hz, 1H, H-3), 4.04 (td, *J* = 6.7, 1.9 Hz, 1H, H-5'), 3.90 (dd, *J* = 10.0, 3.6 Hz, 1H, H-2), 3.83 (dd, *J* = 10.4, 6.3 Hz, 1H, H-6'a), 3.77 (dd, *J* = 10.4, 7.0 Hz, 1H, H-6'b), 3.56 – 3.48 (m, 2H, H-6a, H-6b), 1.52 (s, 3H, CH_3), 1.43 (s, 3H, CH_3), 1.32 (s, 3H, CH_3), 1.31 (s, 3H, CH_3).

^{13}C{^{1}H} NMR (126 MHz, $CDCl_3$): δ 165.9 (C=O), 138.7 (Ar), 138.5 (Ar), 137.9 (Ar), 133.1 (Ar), 130.3 (Ar), 130.0 (Ar), 128.48 (Ar), 128.46 (Ar), 128.38 (Ar), 128.31 (Ar), 128.02 (Ar), 127.97 (Ar), 127.92 (Ar), 127.73 (Ar), 127.67 (Ar), 127.5 (Ar), 109.4 ($(CH_3)_2C$), 108.7 ($(CH_3)_2C$), 98.2 (C-1), 96.5 (C-1'), 76.5 (C-3), 75.5 (C-2), 73.6 (OCH_2Ar), 73.2 (OCH_2Ar), 72.0 (OCH_2Ar), 71.1 (C-4'), 70.84 (C-2'), 70.78 (C-3'), 68.8 (C-4), 68.6 (C-6), 68.0 (C-5), 66.9 (C-5'), 66.3 (C-6'), 26.3 (CH_3), 26.2 (CH_3), 25.1 (CH_3), 24.8 (CH_3).

HRMS–ESI (*m/z*): $[M + Na]^+$ calcd for $C_{46}H_{52}NaO_{12}$, 819.3351; found, 819.3325.

6-*O*-(4-*O*-benzoyl-2,3,6-tri-*O*-benzyl-*β*-D-galactopyranosyl)-1,2:3,4-di-*O*-isopropylidene-*α*-D-galactopyranose (**41**β)

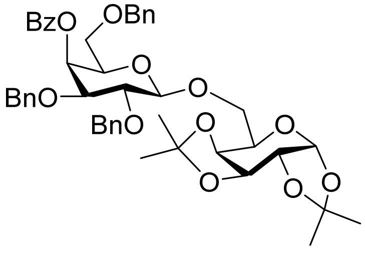

R_f = 0.37 (2:8 EtOAc/hexanes)

Colorless syrup; **^{1}H NMR (500 MHz, $CDCl_3$):** δ 8.11 – 8.03 (m, 2H, Ar-H), 7.59 – 7.53 (m, 1H, Ar-H), 7.45 – 7.38 (m, 4H, Ar-H), 7.29 – 7.23 (m, 13H, Ar-H), 5.80 (dd, *J* = 3.3, 1.1 Hz, 1H, H-4), 5.56 (d, *J* = 5.0 Hz, 1H, H-1'), 4.99 (d, *J* = 11.0 Hz, 1H, OCH_2Ar), 4.82 (d, *J* = 11.7 Hz, 1H, OCH_2Ar), 4.69 (d, *J* = 11.0 Hz, 1H, OCH_2Ar), 4.59 – 4.54 (m, 2H, OCH_2Ar, H-3'), 4.51 – 4.46 (m, 2H, H-1, OCH_2Ar), 4.42 (d, *J* = 11.8 Hz, 1H, OCH_2Ar), 4.31 (dd, *J* = 5.0, 2.4 Hz, 1H, H-2'), 4.23 (dd, *J* = 7.9, 1.6 Hz, 1H, H-3), 4.16 – 4.09 (m, 2H, H-6'a, H-5'), 3.83 – 3.77 (m, 2H, H-6'b, H-4'), 3.70 – 3.53 (m, 4H, H-2, H-5, H-6a, H-6b), 1.50 (s, 3H, CH_3), 1.43 (s, 3H, CH_3), 1.31 (s, 3H, CH_3), 1.30 (s, 3H, CH_3).

$^{13}C\{^1H\}$ NMR (126 MHz, $CDCl_3$): δ 165.9 (C=O), 138.9 (Ar), 138.1 (Ar), 137.7 (Ar), 133.1 (Ar), 130.1 (Ar), 130.0 (Ar), 128.49 (Ar), 128.47 (Ar), 128.4 (Ar), 128.3 (Ar), 128.2 (Ar), 128.1 (Ar), 128.0 (Ar), 127.8 (Ar), 127.6 (Ar), 127.4 (Ar), 109.5 ($(CH_3)_2C$), 108.7 ($(CH_3)_2C$), 104.8 (C-1), 96.5 (C-1'), 79.2 (C-2), 78.7 (C-3), 75.1 (OCH_2Ar), 73.8 (C-4'), 72.5 (OCH_2Ar), 72.1 (OCH_2Ar), 71.5 (C-5), 70.9 (C-3'), 70.6 (C-2'), 70.0 (C-6'), 68.4 (C-6), 67.6 (C-4), 67.4 (C-5'), 26.2 (CH_3), 26.1 (CH_3), 25.1 (CH_3), 24.5 (CH_3).

HRMS–ESI (*m/z*): $[M + Na]^+$ calcd for $C_{46}H_{52}NaO_{12}$, 819.3351; found, 819.3327.

Synthesis of compounds 3-*^{13}C* and 44

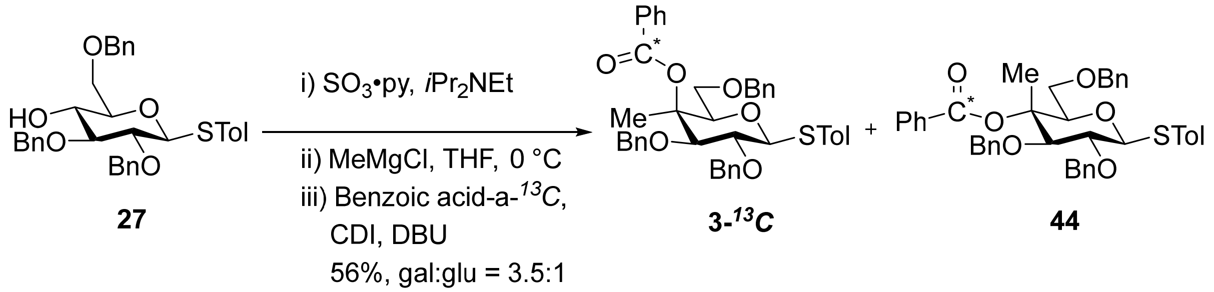

Compound **27** (0.35 g, 0.628 mmol) was dissolved in anhydrous solution of DMSO:CH_2Cl_2 2:1 (5 mL). The mixture was cooled to 0 °C and added diisopropylethylamine (0.55 mL, 3.14 mmol) sulfur trioxide pyridine complex (0.4 g, 2.51 mmol). The reaction mixture was allowed to gradually warm up to the rt and stirred until completion of the starting material **27** (detected by LCMS and TLC) (EtOAc:hexanes: 4:6, R_f =0.62). The reaction mixture was concentrated under reduced pressure and the residue diluted with Et_2O (30 mL) and water (30 mL). The aqueous layer was extracted with Et_2O twice and the combined organic layer was washed with aqueous saturated $NaHCO_3$ (40 mL), brine (40 mL), dried over Na_2SO_4, filtered, and concentrated to give the intermediate ketone as which was used in the next step without purification. MeMgCl (3 M in THF) (0.6 mL, 1.88 mmol) was added to a stirred solution of ketone (0.628 mmol) in THF (5 mL) at 0 °C. After 1 h the reaction was quenched with 4 mL aqueous saturated NH_4Cl solution, diluted with Et_2O (30 mL), washed with aqueous NH_4Cl (30 mL) and brine (30 mL), dried with Na_2SO_4, and concentrated to give the intermediate tertiary alcohol, which was used in the next step without purification. Benzoic acid-α-

^{13}C (0.39 g, 3.14 mmol) and 1,1′- carbonyldiimidazole (0.509 g, 3.14 mmol) were dissolved in anhydrous THF (2 mL). The reaction mixture was stirred at rt for 1 hr. A solution of crude alcohol (0.628 mmol) in THF (2 mL) was added in a dropwise manner, followed by addition of 1,8-diazabicyclo[5.4.0]undec-7-ene (DBU) (0.19 mL, 1.26 mmol) was done. The reaction was stirred for 48 h at 35 °C. After 48 h addition of DBU (0.19 mL, 1.26 mmol) was done which was repeated after 48 h. It was neutralized with aqueous saturated $NaHCO_3$ solution (5 mL), diluted with EtOAc (10 mL). Organic layer was separated out, it was collected whereas aqueous layer was extracted two times with EtOAc (2 × 10 ml). Organic layers were collected, dried over Na_2SO_4, filtered, and concentrated under reduced pressure to afford crude product, which was purified by column chromatography (1:9 EtOAc/hexanes) to give compounds **3-*^{13}C*** (187 mg) and **44** (53 mg) (overall 56% over three steps) as white solids.

p*-Methylphenyl 4-*O*-benzoyl-2,3,6-tri-*O*-benzyl-4-*C*-methyl-1-thio-*β*-D-galactopyranoside-*^{13}C (**3-*^{13}C***)

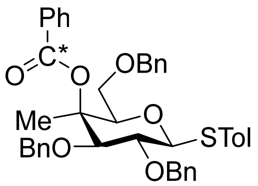

R_f = 0.31 (1:9 EtOAc/hexanes)

^{1}H NMR (500 MHz, $CDCl_3$): δ 7.99 – 7.95 (m, 2H, Ar-H), 7.62 – 7.55 (m, 1H, Ar-H), 7.52 – 7.47 (m, 2H, Ar-H), 7.45 – 7.41 (m, 2H, Ar-H), 7.41 – 7.37 (m, 2H, Ar-H), 7.36 – 7.25 (m, 13H, Ar-H), 6.93 – 6.85 (m, 2H, Ar-H), 4.97 (d, *J* = 11.4 Hz, 1H, OCH_2Ar), 4.93 (d, *J* = 10.2 Hz, 1H, OCH_2Ar), 4.79 (d, *J* = 11.4 Hz, 1H, OCH_2Ar), 4.75 (d, *J* = 9.7 Hz, 1H, H-1), 4.72 (d, *J* = 10.2 Hz, 1H, OCH_2Ar), 4.62 – 4.52 (m, 2H, OCH_2Ar), 4.15 (dd, *J* = 11.1, 2.3 Hz, 1H, H-6a), 3.91 (t, *J* = 9.3 Hz, 1H, H-2), 3.77 (dd, *J* = 11.1, 7.5 Hz, 1H, H-6b), 3.66 (dd, *J* = 7.5, 2.3 Hz, 1H, H-5), 3.43 (d, *J* = 8.9 Hz, 1H, H-3), 2.24 (s, 3H, CH_3), 1.76 (s, 3H, CH_3).

$^{13}C\{^1H\}$ NMR (126 MHz, $CDCl_3$): δ 165.6 (C=O), 138.4 (Ar), 138.3 (Ar), 138.1 (Ar), 137.4 (Ar), 132.8 (Ar), 132.2 (Ar), 131.6 (d, *J* = 75.3 Hz, 1C, Ar), 130.2 (Ar), 130.0 (*J* = 2.4 Hz, Ar), 129.7 (Ar), 128.5 (Ar), 128.4 (Ar), 128.0 (Ar), 127.9 (Ar), 127.7 (Ar), 127.6 (Ar), 88.2 (C-1), 87.2 (*J* = 2.1 Hz, 3C, C-3), 83.8 (*J* = 3.0 Hz, C-4), 83.6 (C-5), 78.6 (C-2), 76.3 (OCH_2Ar), 75.5 (OCH_2Ar), 73.7 (OCH_2Ar), 70.5 (C-6), 21.2 (CH_3), 18.1 (CH_3).

HRMS–ESI (*m/z*): $[M + Na]^+$ calcd for $C_{41}{}^{13}CH_{42}NaO_6S$, 698.2628; found, 698.2610.

***p*-Methylphenyl 4-*O*-benzoyl-2,3,6-tri-*O*-benzyl-4-*C*-methyl-1-thio-*β*-D-glucopyranoside-^{13}C (44)**

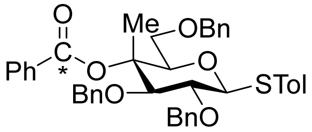

***R*$_f$** = 0.40 (1:9 EtOAc/hexanes)

$[\alpha]_D^{23}$ +11.4 (*c* 0.1, $CHCl_3$)

^{1}H NMR (500 MHz, CD_2Cl_2): δ 7.95 – 7.91 (m, 2H, Ar-H), 7.57 – 7.20 (m, 20H, Ar-H), 7.07 – 7.04 (m, 2H, Ar-H), 4.88 – 4.83 (m, 3H, H-3, H-5, OCH_2Ar), 4.81 (d, *J* = 9.9 Hz, 1H, H-1), 4.72 – 4.65 (m, 2H, OCH_2Ar), 4.55 (d, *J* = 11.0 Hz, 1H, OCH_2Ar), 4.44 (s, 2H, OCH_2Ar), 3.72 – 3.65 (m, 2H, H-6a, H-6b), 3.42 (dd, *J* = 9.9, 8.7 Hz, 1H, H-2), 2.29 (s, 3H, CH_3), 1.46 (s, 3H, CH_3).

$^{13}C\{^1H\}$ NMR (126 MHz, CD_2Cl_2): δ 165.6 (C=O), 138.5 (Ar), 138.4 (Ar), 138.3 (Ar), 137.8 (Ar), 133.2 (Ar), 132.2 (Ar), 130.7 (d, *J* = 74.2 Hz, 1C, Ar) 130.0 (Ar), 129.7 (Ar), 128.4 (d, *J* = 2.4 Hz, Ar) 128.3 (Ar), 128.2 (Ar), 128.0 (Ar), 127.7 (Ar), 127.6 (Ar), 127.4 (Ar), 88.0 (C-1), 84.4 (d, *J* = 2.9 Hz, 2C, C-4), 82.7 (C-3), 81.0 (C-2), 77.4 (C-5), 75.3 (OCH_2Ar), 75.2 (OCH_2Ar), 73.2 (OCH_2Ar), 68.3 (C-6), 20.8 (CH_3), 15.0 (d, *J* = 2.4 Hz, CH_3).

HRMS–ESI (*m/z*): $[M + Na]^+$ calcd for $C_{41}{}^{13}CH_{42}KO_6S$, 714.2367; found, 714.2335.

Synthesis of compounds 45

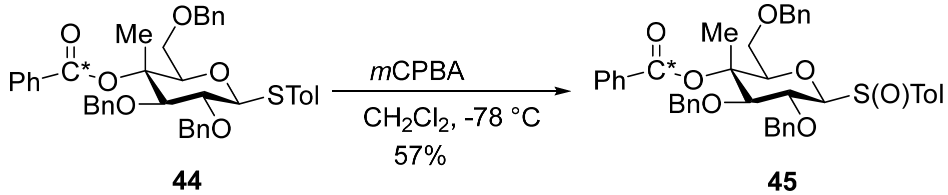

44 **45**

To a stirred solution of thioglycoside **44** (45 mg, 0.066 mmol) in anhydrous dichloromethane (0.5 mL), a solution of *m*CPBA (12 mg, 0.066 mmol, 77%) in anhydrous dichloromethane (0.5 mL) was added in a dropwise manner at -70 °C under argon. After 17 mins, the reaction mixture was neutralized with aqueous saturated $NaHCO_3$ solution (2 mL) and then warmed up to room temperature. The mixture was extracted with dichloromethane (2×10 mL); all combined organic phases were washed with brine, dried over Na_2SO_4, filtered, and evaporated under reduced pressure to give a crude residue. The crude product was purified by column chromatography (30% EtOAc/hexanes) to afford 1:0.3 ratio of two unidentified isomers (26 mg, 57%) as a colorless syrup. Only major isomer spectral data is provided here.

_p_-Methylphenyl4-_O_-benzoyl-2,3,6-tri-_O_-benzyl-4-_C_-methyl-1-thio-_β_-D-glucopyranosylsulfoxide-^{13}C (45)

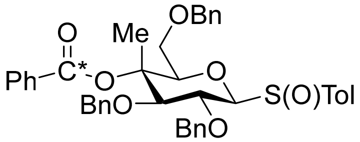

R_f = 0.45 (3:7 EtOAc/hexanes)

[α]$_D^{23}$ –73.1 (*c* 0.1, $CHCl_3$)

^{1}H NMR (500 MHz, CD_2Cl_2): δ 7.93 – 7.88 (m, 2H, Ar-H), 7.57 – 7.52 (m, 3H, Ar-H), 7.40 – 7.13 (m, 17H, Ar-H), 6.99 – 6.95 (m, 2H, Ar-H), 5.00 (d, *J* = 8.9 Hz, 1H, H-3), 4.92 (s, 2H, OCH_2Ar), 4.75 – 4.70 (m, 2H, OCH_2Ar, H-5), 4.61 (d, *J* = 11.1 Hz, 1H, OCH_2Ar), 4.20 (d, *J* = 9.9 Hz, 1H, H-1), 4.08 (t, *J* = 9.3 Hz, 1H, H-2), 4.00 – 3.90 (m, 2H, OCH_2Ar), 3.67 (dd, *J* = 11.8, 2.1 Hz, 1H, H-6a), 3.46 (dd, *J* = 11.8, 7.3 Hz, 1H, H-6b), 2.21 (s, 3H, CH_3), 1.50 (s, 3H, CH_3).

^{13}C{^{1}H} NMR (126 MHz, CD_2Cl_2): δ 165.7 (C=O), 141.6 (Ar), 138.3 (d, *J* = 2.92, Ar), 138.1 (Ar), 136.6 (Ar), 133.3 (Ar), 130.4 (d, *J* = 74.5 Hz, 1C, Ar), 129.73 (d, *J* = 2.4, Ar), 129.66 (Ar), 128.42 (Ar), 128.39 (Ar), 128.38 (Ar) 128.2 (Ar), 128.1 (Ar), 127.9 (Ar), 127.7 (Ar), 127.6 (Ar), 127.4 (Ar), 125.2 (Ar), 93.6 (C-1), 84.0 (d, *J* = 2.9, 2C, C-4), 82.3 (C-3), 78.8 (C-5), 76.7 (C-2), 75.6 (OCH_2Ar), 75.0 (OCH_2Ar), 73.2 (OCH_2Ar), 68.2 (C-6), 21.1 (CH_3), 14.8 (d, *J* = 3.24, CH_3).

HRMS–ESI (*m/z*): $[M + Na]^+$ calcd for $C_{41}{}^{13}CH_{42}NaO_7S$, 714.2570; found, 714.2571.

General procedure for variable temperature (VT) NMR experiment

A solution of **45** (1.0 equiv.) in CD_2Cl_2 (0.7 mL) containing TTBP (1.0 equiv.) was placed into an NMR tube and cooled to -80 °C in the NMR probe. The first ^{1}H and ^{13}C NMR spectra were obtained, then the sample was quickly removed from the probe and the addition of Tf_2O (2.0 equiv.) precooled at -78 °C was done quickly. The sample was returned to the NMR probe and ^{1}H and ^{13}C NMR spectra were recorded after 10 mins. The temperature was increased by 10 °C increments every 10 minutes and ^{1}H and ^{13}C NMR, spectra were acquired at each temperature.

1,6-Anhydro-4-_O_-benzoyl-2,3-di-_O_-benzyl-4-_C_-methyl-_β_-D-glucopyranose-^{13}C (47)

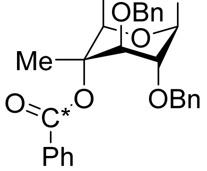

This compound was obtained from the decomposition of donor **45** (20 mg, 28.9 µmol) when variable temperature (VT) NMR studies were carried out following the general procedure for VT study. The reaction mixture was quenched with triethylamine (20.0 µL) at room temperature and diluted with dichloromethane (5 mL), washed with saturated aqueous $NaHCO_3$ (2 mL). The organic layer was

separated and dried over Na_2SO_4, filtered, and concentrated under reduced pressure. Crude product was purified by flash column chromatography (10% EtOAc/hexanes) to afford compound **47** (4 mg, 31%) as a colorless syrup.

R

R_f = 0.22 (1:9 EtOAc/hexanes)

$[\alpha]_D^{23}$ –46.4 (*c* 0.3, $CHCl_3$)

^{1}H NMR (500 MHz, $CDCl_3$): δ 8.02 – 7.95 (m, 2H, Ar-H), 7.50 – 7.44 (m, 1H, Ar-H), 7.34 – 7.15 (m, 12H, Ar-H), 5.54 (s, 1H, H-1), 5.10 (dd, *J* = 4.5, 1.5 Hz, 1H, H-5), 4.60 – 4.55 (m, 2H, OCH_2Ar), 4.46 (s, 2H, OCH_2Ar), 4.26 – 4.21 (m, 1H, H-6a), 4.00 (d, *J* = 1.7 Hz, 1H, H-3), 3.75 (dd, *J* = 7.4, 5.9 Hz, 1H, H-6b), 3.44 (t, *J* = 1.6 Hz, 1H, H-2), 1.70 (s, 3H, CH_3).

^{13}C{^{1}H} NMR (126 MHz, $CDCl_3$): δ 166.0 (C=O), 137.72 (Ar), 137.68 (Ar), 132.8 (Ar), 131.3 (d, *J* = 74.8 Hz, 1C, Ar), 129.9 (d, *J* = 2.5, Ar), 128.6 (Ar), 128.5 (Ar), 128.31 (Ar), 128.27 (Ar), 128.03 (Ar), 128.0, 127.9 (Ar), 127.6 (Ar), 100.5 (C-1), 80.9 (C-4), 77.3 (C-3), 76.1 (C-5), 75.4 (C-2), 73.6 (OCH_2Ar), 72.2 (OCH_2Ar), 64.3 (C-6), 18.1 (CH_3).

HRMS–ESI (*m/z*): $[M + Na]^+$ calcd for $C_{27}{}^{13}CH_{28}NaO_6$, 484.1811; found, 484.1801.

Table S2. Influence of concentration and diphenyl sulfoxide equivalence on the coupling of donor **3** with diacetone galactopyranose **31**.

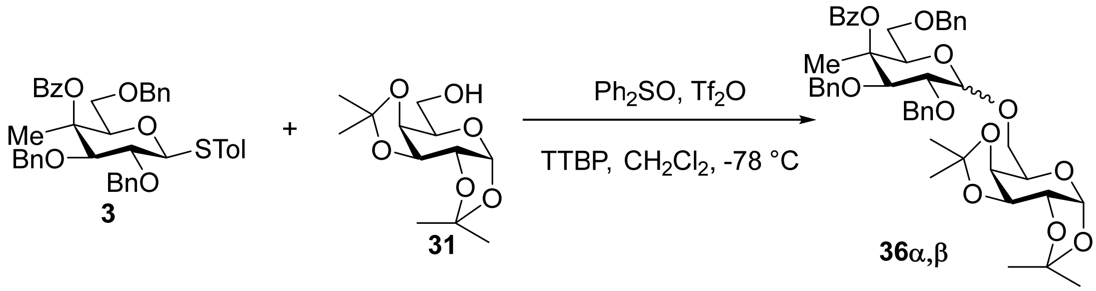

Entry	Donor **3**, (M)	Acceptor **31**, (M)	Ph_2SO, (M)	Tf_2O, (M)	TTBP, (M)	**36**, %yield, α:β ratio
1	0.05	0.05	0.05	0.05	0.075	65%, α-only
2	0.10	0.083	0.20	0.15	0.20	45%, α:β = 1.2:1
3	0.10	0.083	0.10	0.15	0.20	42%, α:β = 1:1
4	0.05	0.05	0.10	0.05	0.075	51%, α:β = 3.2:1

Entry 1: Reproduced results obtained from the reaction of donor **3** and acceptor **31** following the general procedure for glycosylation.

Entry 2: Compounds **36α** and **36β** (combined yield 42 mg, 45%; α/β = 1.2:1) were obtained from the reaction of donor **3** (78 mg, 0.115 mmol) and acceptor **31** (25 mg, 0.096 mmol) after column

chromatography (2:8 EtOAc/hexanes) following the general procedure for glycosylation conditions reported previously.[3]

Entry 3: Compounds **36**α and **36**β (combined yield 39 mg, 42%; α/β = 1:1) were obtained from the reaction of donor **3** (78 mg, 0.115 mmol) and acceptor **31** (25 mg, 0.096 mmol) after column chromatography (2:8 EtOAc/hexanes) following the general procedure for glycosylation conditions reported previously.[3]

Entry 4: Compounds **36**α and **36**β (combined yield 40 mg, 51%; α/β = 3.2:1) were obtained from the reaction of donor **3** (65 mg, 0.096 mmol) and acceptor **31** (25 mg, 0.096 mmol) after column chromatography (2:8 EtOAc/hexanes) following the general procedure for glycosylation conditions.

6-*O*-(4-*O*-benzoyl-2,3,6-tri-*O*-benzyl-4-*C*-methyl-*β*-D-galactopyranosyl)-1,2:3,4-di-*O*-isopropylidene-*α*-D-galactopyranose (36β)

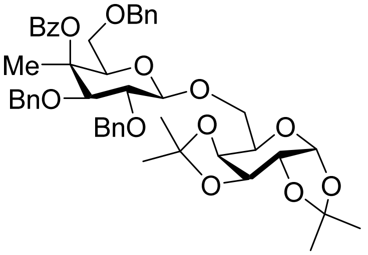

***R**_f_* = 0.26 (2:8 EtOAc/hexanes)

Colorless syrup; **^{1}H NMR (500 MHz, CDCl$_3$):** δ 7.94 – 7.89 (m, 2H, Ar-H), 7.65 – 7.62 (m, 1H, Ar-H), 7.46 – 7.43 (m, 2H, Ar-H), 7.40 – 7.25 (m, 15H, Ar-H), 5.57 (d, *J* = 5.0 Hz, 1H, H-1'), 5.04 (d, *J* = 11.4 Hz, 2H, OCH$_2$Ar), 4.82 – 4.70 (m, 2H, OCH$_2$Ar), 4.63 – 4.50 (m, 4H, H-1, H-3' OCH$_2$Ar), 4.30 (dd, *J* = 5.1, 2.4 Hz, 1H, H-2'), 4.20 (dd, *J* = 7.9, 1.9 Hz, 1H, H-5'), 4.18 – 4.08 (m, 3H, H-5, H-6a, H-6a'), 3.87 – 3.80 (m, 2H, H-2, H-6'b), 3.65 (dd, *J* = 11.1, 7.4 Hz, 1H, H-6b), 3.56 (dd, *J* = 7.4, 2.6 Hz, 1H, H-4'), 3.29 (d, *J* = 9.4 Hz, 1H, H-3), 1.69 (s, 3H, CH$_3$), 1.47 (s, 3H, CH$_3$), 1.43 (s, 3H, CH$_3$), 1.29 (s, 3H, CH$_3$), 1.28 (s, 3H, CH$_3$).

^{13}C{^{1}H} NMR (126 MHz, CDCl$_3$): δ 165.7 (C=O), 138.64 (Ar), 138.57 (Ar), 138.4 (Ar), 132.7 (Ar), 131.6 (Ar), 131.1 (Ar), 130.0 (Ar), 129.4 (Ar), 128.9 (Ar), 128.5 (Ar), 128.31 (Ar), 128.26 (Ar), 128.2 (Ar), 127.8 (Ar), 127.64 (Ar), 127.59 (Ar), 127.53 (Ar), 127.47 (Ar), 124.9 (Ar), 109.5 ((CH$_3$)$_2$C), 108.7 ((CH$_3$)$_2$C), 105.1 (C-1), 96.5 (C-1'), 84.9 (C-3), 83.7 (C-4), 79.4 (C-2), 79.1 (C-4'), 76.1 (C-2'), 74.4 (C-3'), 73.7 (C-6'), 71.5 (C-6), 70.9 (C-5'), 70.6 (OCH$_2$Ar), 70.4 (OCH$_2$Ar), 70.3 (OCH$_2$Ar), 67.7 (C-5), 26.12 (CH$_3$), 26.09 (CH$_3$), 25.1 (CH$_3$), 24.5 (CH$_3$), 18.0 (CH$_3$).

HRMS–ESI (*m/z*): [M + Na]$^+$ calcd for $C_{47}H_{54}NaO_{12}$, 833.3513; found, 833.3469.

Ethyl 2,3-bis-*O*-benzyl-4,6-bis(2,2-dimethylpropanoate)-1-thio-*β*-D-galactopyranoside (**51**)

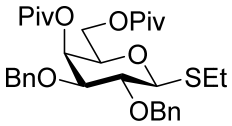

Compound **51** was synthesized according to literature precedent.[4]

R_f = 0.55 (1.5:8.5 EtOAc/hexanes)

^{1}H NMR (500 MHz, CD_3CN): δ 7.33 – 7.20 (m, 10H, Ar-H), 5.52 (dd, *J* = 3.3, 1.1 Hz, 1H, H-4), 4.74 – 4.65 (m, 3H, OCH_2Ar), 4.51 (d, *J* = 9.7 Hz, 1H, H-1), 4.45 (d, *J* = 11.0 Hz, 1H, OCH_2Ar), 4.08 – 3.94 (m, 2H, H-6a, H-6b), 3.89 (ddd, *J* = 7.3, 6.0, 1.1 Hz, 1H, H-5), 3.70 (dd, *J* = 9.2, 3.3 Hz, 1H, H-3), 3.47 (t, *J* = 9.5 Hz, 1H, H-2), 2.73 (dq, *J* = 12.8, 7.3 Hz,1H, -S-CH_2-CH_3), 2.62 (dq, *J* = 13.0, 7.6 Hz, 1H, -S-CH_2-CH_3), 1.24 (t, *J* = 7.4 Hz, 3H, -S-CH_2-CH_3), 1.16 (s, 9H, -OPiv), 1.15 (s, 9H, -OPiv).

^{13}C{^{1}H} NMR (126 MHz, CD_3CN): δ 177.6 (C=O, -OPiv), 177.2 (C=O, -OPiv), 138.7 (Ar), 138.4 (Ar), 128.3 (Ar), 128.2 (Ar), 128.1 (Ar), 127.6 (Ar), 84.3 (C-1), 81.0 (C-3), 77.5 (C-2), 75.0 (OCH_2Ar), 74.2 (C-5), 71.5 (OCH_2Ar), 66.6 (C-4), 61.9 (C-6), 38.8 (C_q, -OPiv), 38.4 (C_q, -OPiv), 26.6 (-OPiv), 26.4 (-OPiv), 23.8 (-S-CH_2-CH_3), 14.7 (-S-CH_2-CH_3).

HRMS–ESI (*m/z*): $[M + Na]^+$ calcd for $C_{32}H_{44}NaO_7S$, 595.2705; found, 595.2677.

References

1. Dharuman, S.; Amarasekara, H.; Crich, D. Interplay of Protecting Groups and Side Chain Conformation in Glycopyranosides. Modulation of the Influence of Remote Substituents on Glycosylation? *J. Org. Chem.* **2018**, *83*, 10334-10351.
2. Shie, C.-R.; Tzeng, Z.-H.; Kulkarni, S. S.; Uang, B.-J.; Hsu, C.-Y.; Hung, S.-C. $Cu(OTf)_2$ as an Efficient and Dual-Purpose Catalyst in the Regioselective Reductive Ring Opening of Benzylidene Acetals. *Angew. Chem. Int. Ed.* **2005**, *44*, 1665-1668
3. Upadhyaya, K.; Subedi, Y. P.; Crich, D. Direct Experimental Characterization of a Bridged Bicyclic Glycosyl Dioxacarbenium Ion by ^{1}H and ^{13}C NMR Spectroscopy: Importance of Conformation on Participation by Distal Esters. *Angew. Chem. Int. Ed.* **2021**, *60*, 25397-25403.
4. Greis, K.; Leuichnitz, S.; Kirschbaum, C.; Chang, C.-W.; Lin, M.-H.; Meijer, G.; von Helden, G.; Seeberger, P. H.; Pagel, K. The Influence of the Electron Density in Acyl Protecting Groups on the Selectivity of Galactose Formation. *J. Am. Chem. Soc.* **2022**, *144*, 20258-2026

NMR Spectra

^{1}H NMR (500 MHz, $CDCl_3$) spectrum of methyl 2,3-di-*O*-benzyl-4,6-*O*-benzylidene-6-(*S*)-deuterio-*α*-D-glucopyranoside (18-D_1)

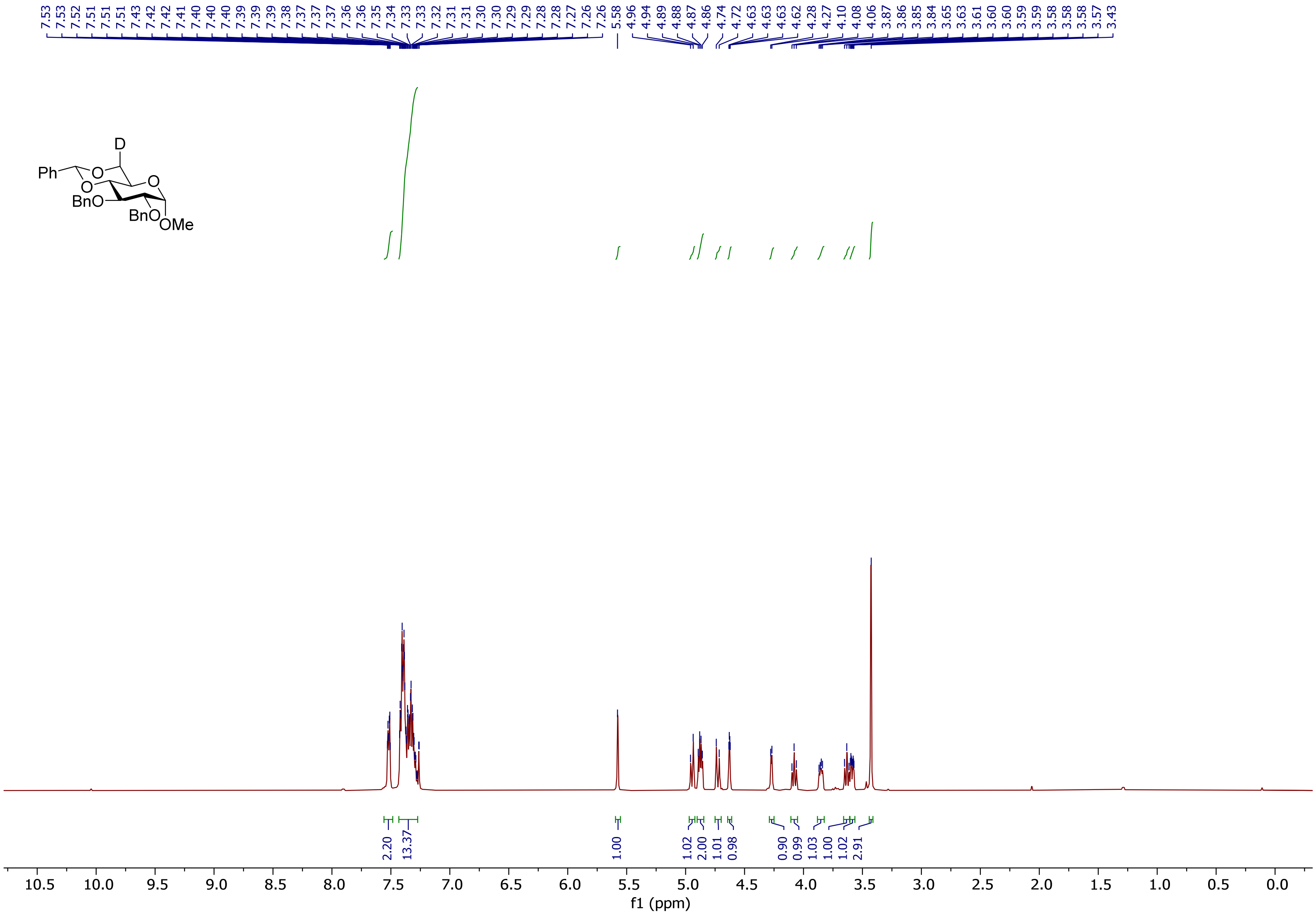

$^{13}C\{^1H\}$ NMR (126 MHz, $CDCl_3$) spectrum of methyl 2,3-di-*O*-benzyl-4,6-*O*-benzylidene-6-(*S*)-deuterio-*α*-D-glucopyranoside (**18-D₁**)

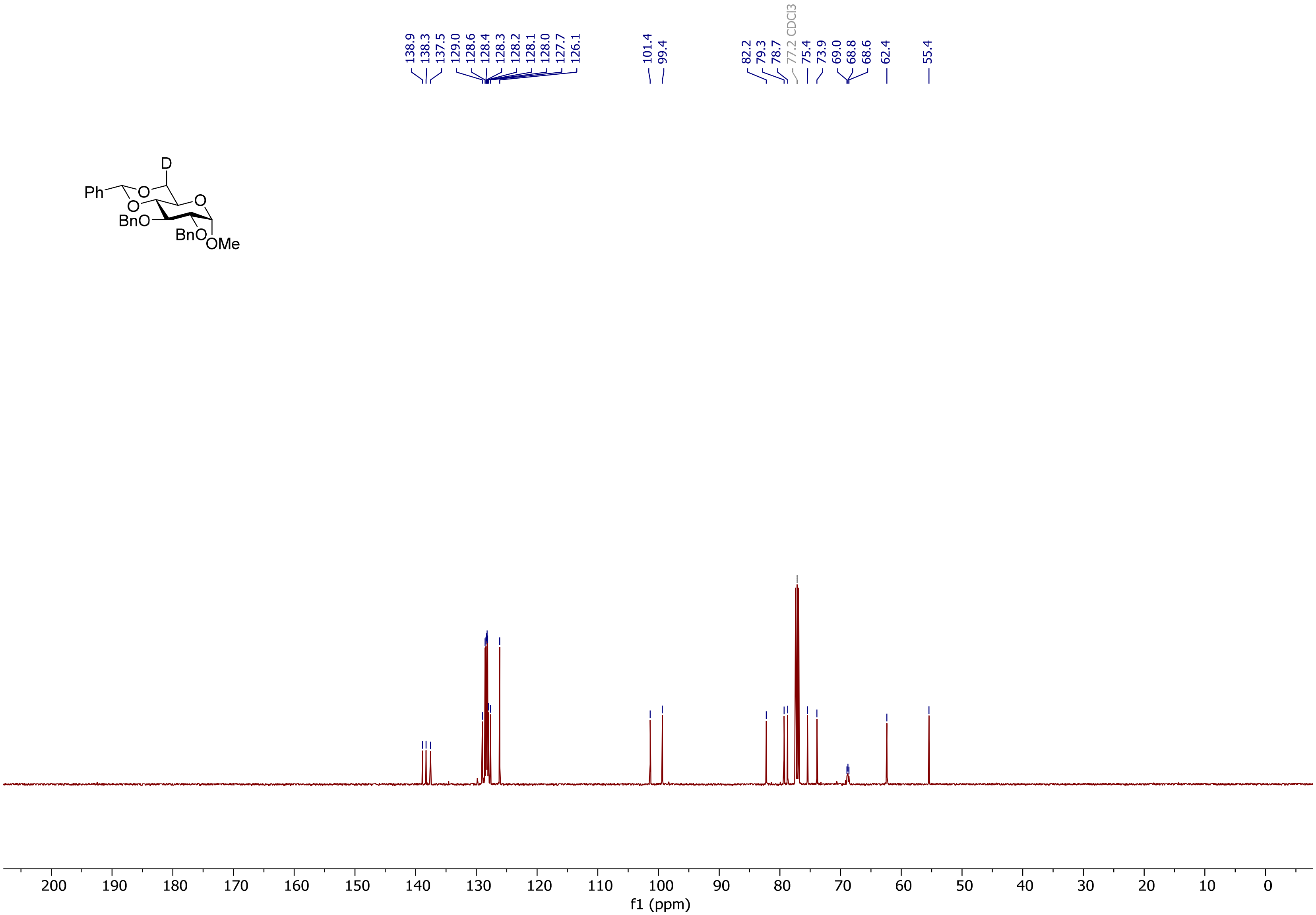

^{1}H NMR (500 MHz, CDCl$_3$) spectrum of Methyl 2,3,6-tri-*O*-benzyl-6-(*S*)-deuterio-*α*-D-glucopyranoside (19-D$_1$)

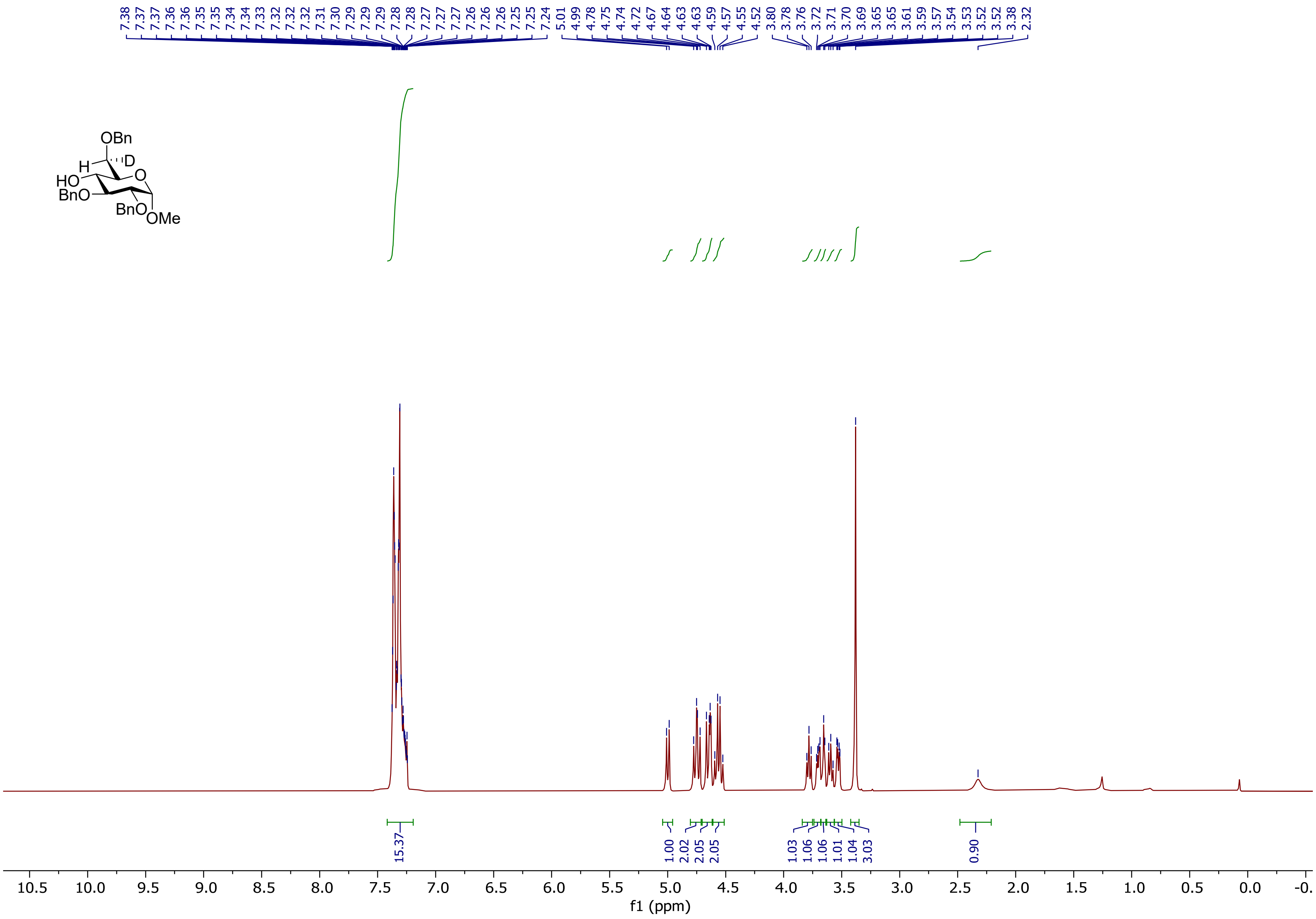

$^{13}C\{^{1}H\}$ NMR (126 MHz, $CDCl_3$) spectrum of methyl 2,3,6-tri-*O*-benzyl-6-(*S*)-deuterio-*α*-D-glucopyranoside (19-D$_1$)

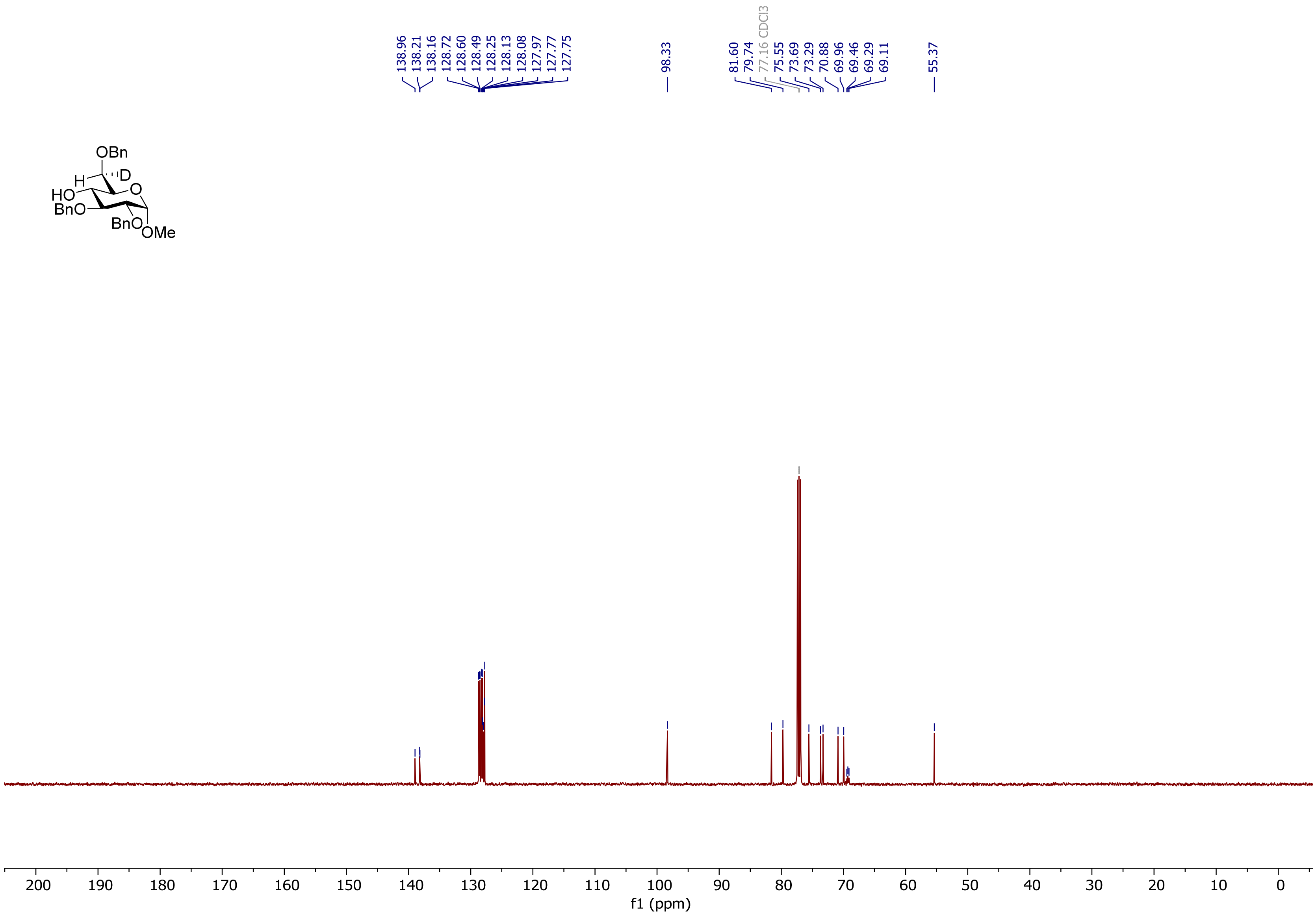

^{1}H NMR (900 MHz, $CDCl_3$) spectrum of methyl 2,3,6-tri-*O*-benzyl-4-*C*-methyl-6-(*S*)-deuterio-α-D-galactopyranoside (20-D$_1$)

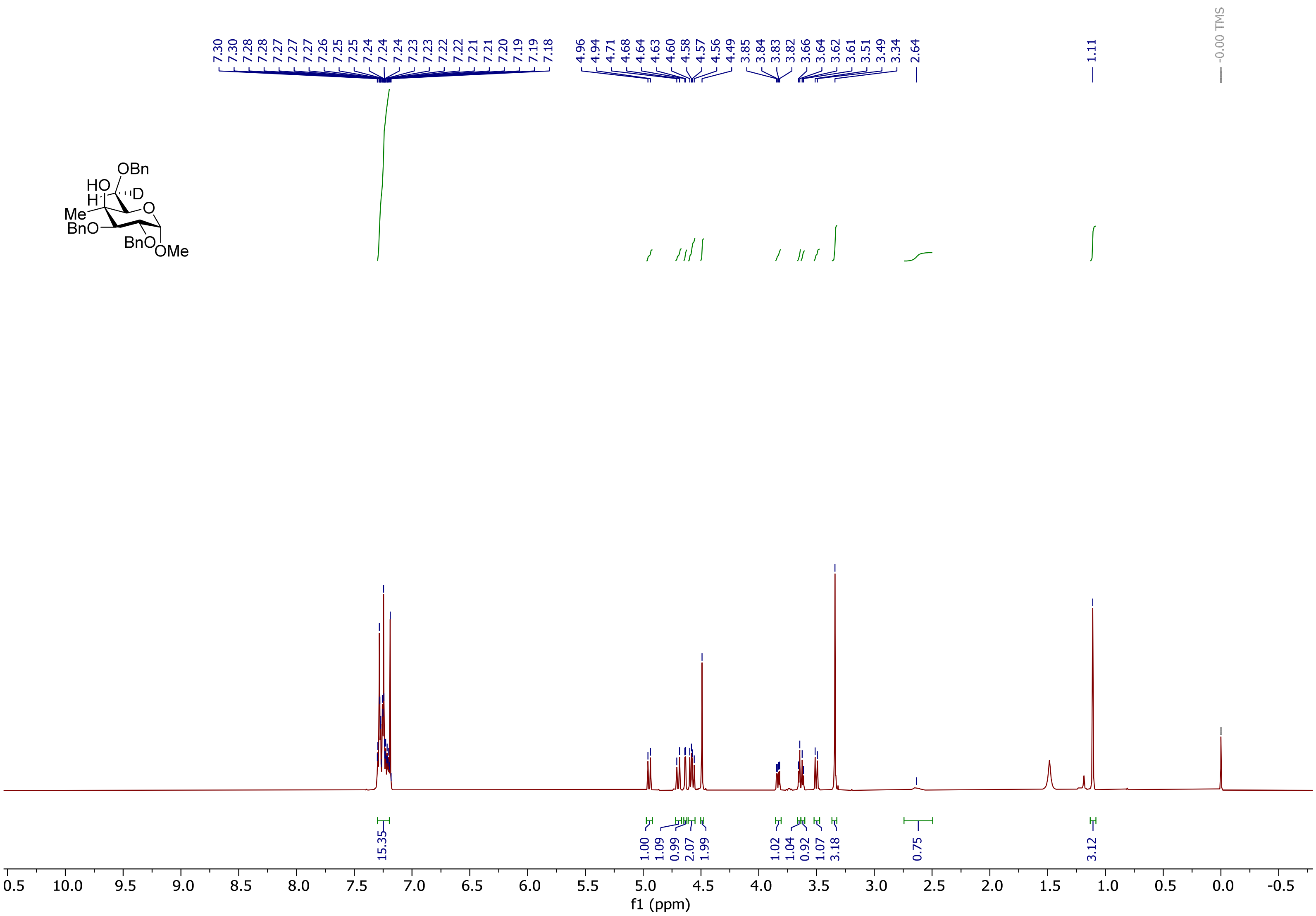

$^{13}C\{^1H\}$ NMR (226 MHz, $CDCl_3$) spectrum of methyl 2,3,6-tri-*O*-benzyl-4-*C*-methyl-6-(*S*)-deuterio-*α*-D-galactopyranoside (20-D$_1$)

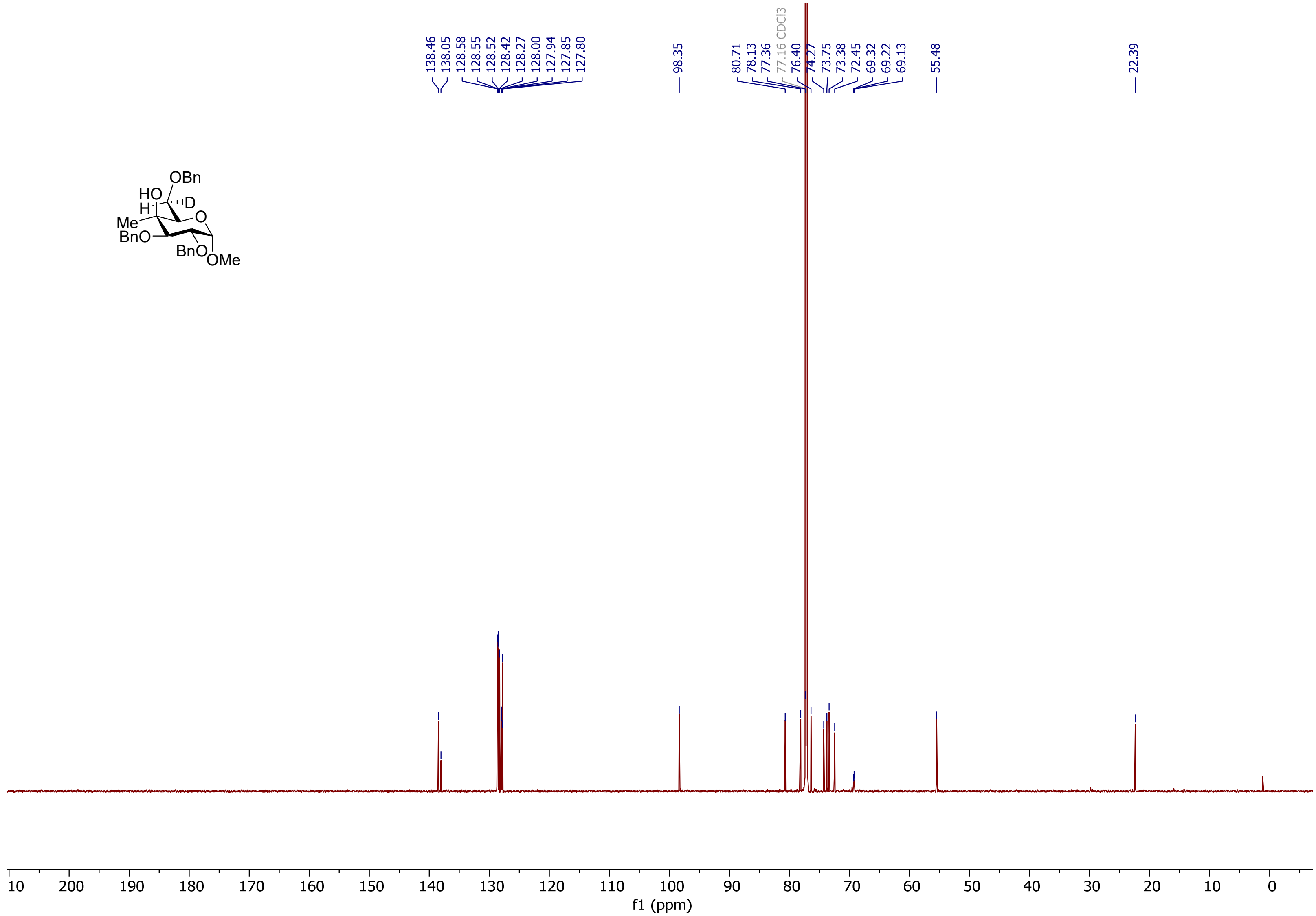

^{1}H NMR (600 MHz, C_6D_6) spectrum of methyl 2,3,6-tri-*O*-benzyl-4-*C*-methyl-6-(*S*)-deuterio-*α*-D-galactopyranoside (20-D$_1$)

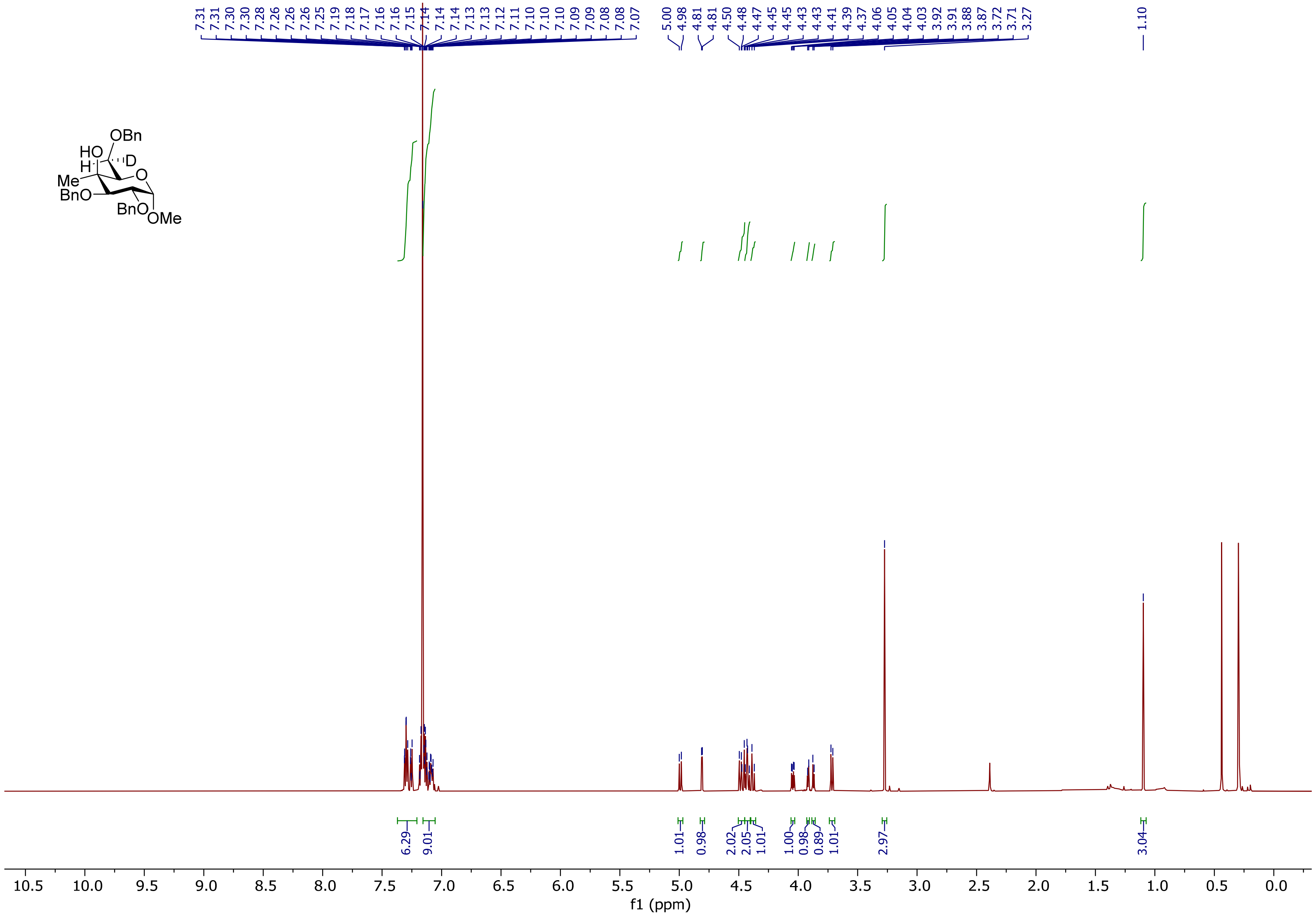

$^{13}C\{^{1}H\}$ NMR (151 MHz, C_6D_6) spectrum of methyl 2,3,6-tri-*O*-benzyl-4-*C*-methyl-6-(*S*)-deuterio-*α*-D-galactopyranoside (20-D_1)

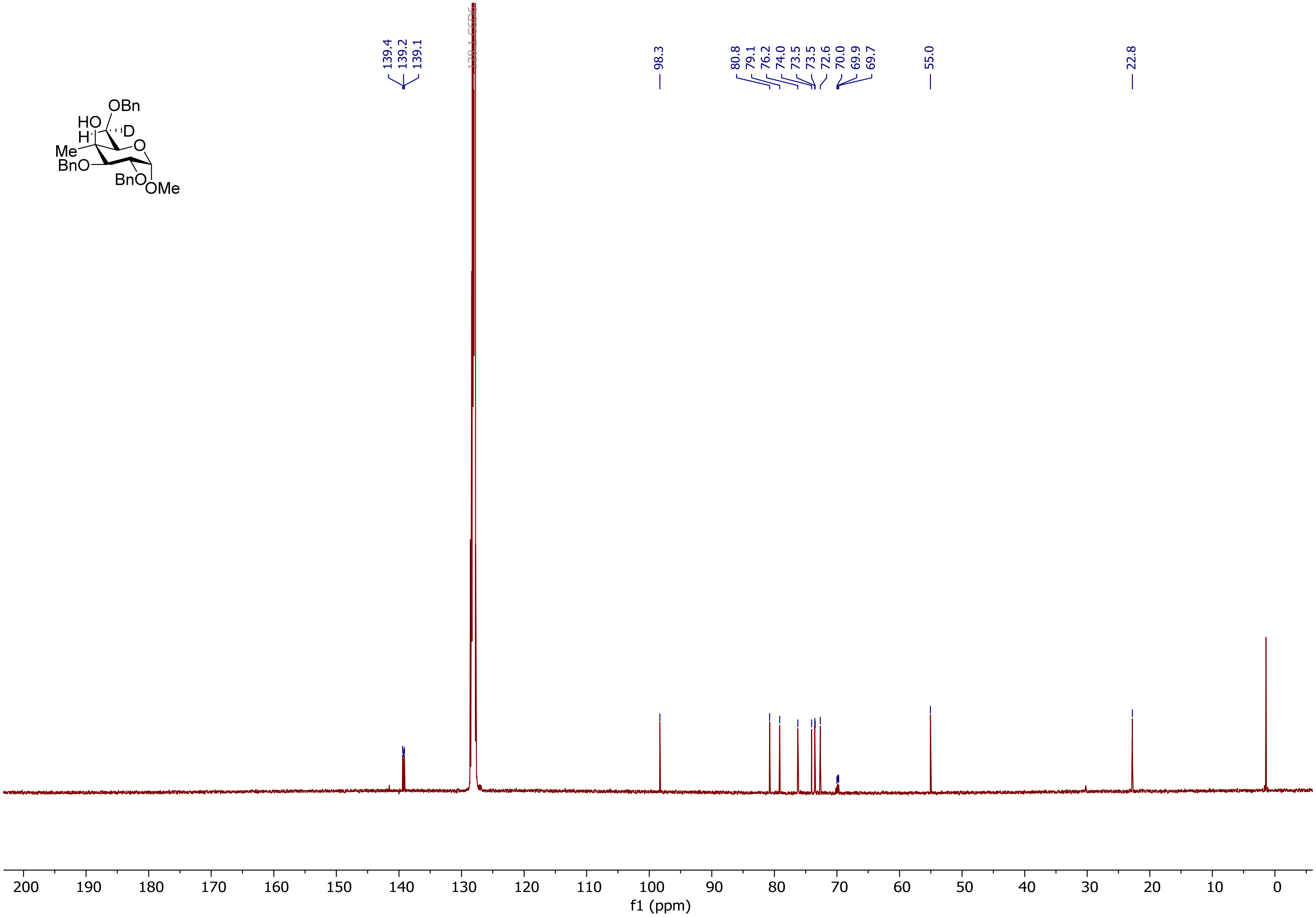

^{1}H NMR (900 MHz, $CDCl_3$) spectrum of methyl 2,3,6-tri-*O*-benzyl-4-*C*-methyl-6-(*S*)-deuterio-*α*-D-glucopyranoside (21-D$_1$)

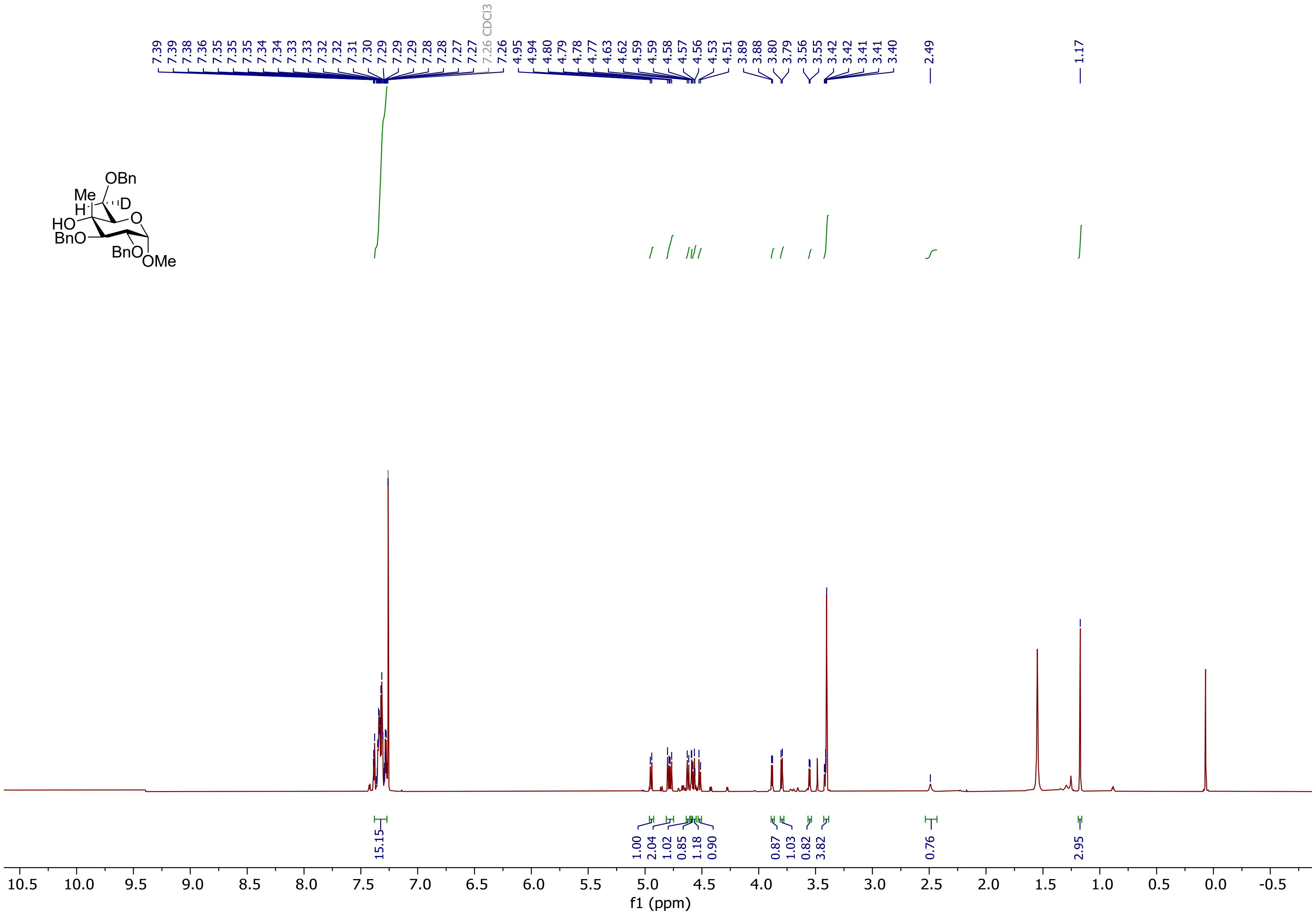

$^{13}C\{^1H\}$ NMR (226 MHz, $CDCl_3$) spectrum of methyl 2,3,6-tri-*O*-benzyl-4-*C*-methyl-6-(*S*)-deuterio-*α*-D-glucopyranoside (21-D_1)

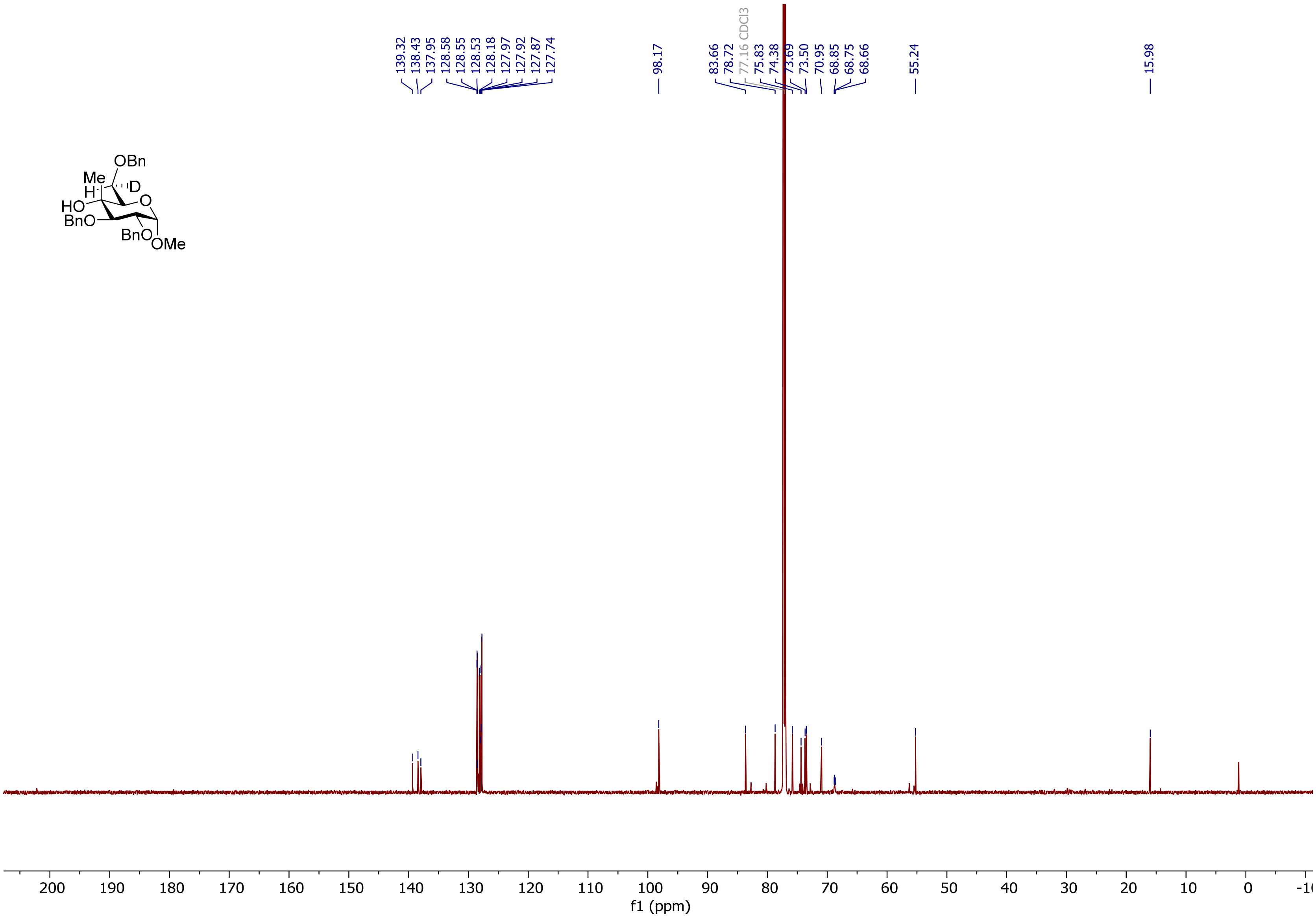

^{1}H NMR (900 MHz, D_2O) spectrum of methyl 4-*C*-methyl-6-(*S*)-deuterio-*α*-D-galactopyranoside (22-D_1)

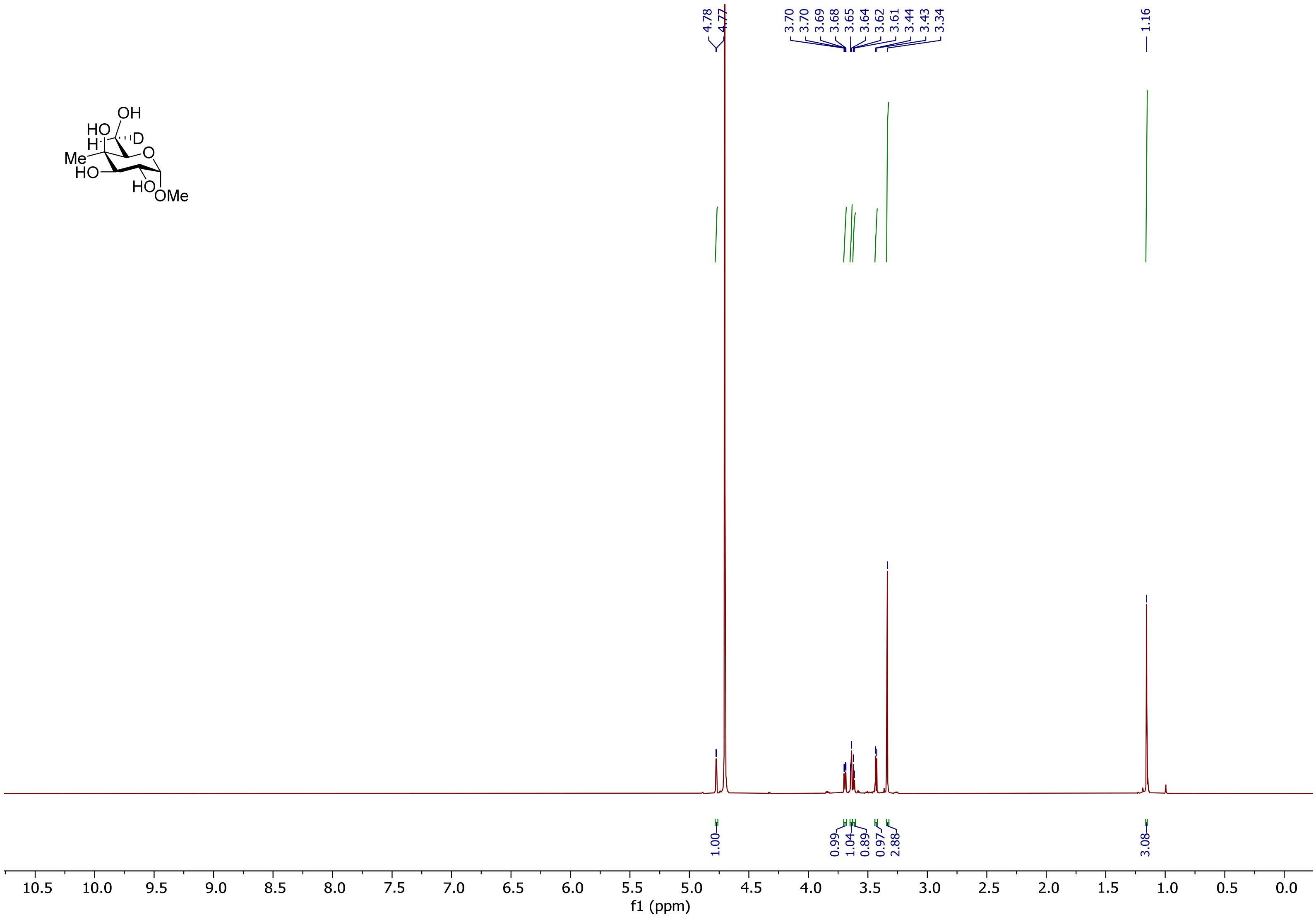

$^{13}C\{^1H\}$ NMR (226 MHz, D_2O) spectrum of methyl 4-*C*-methyl-6-(*S*)-deuterio-*α*-D-galactopyranoside (22-D$_1$)

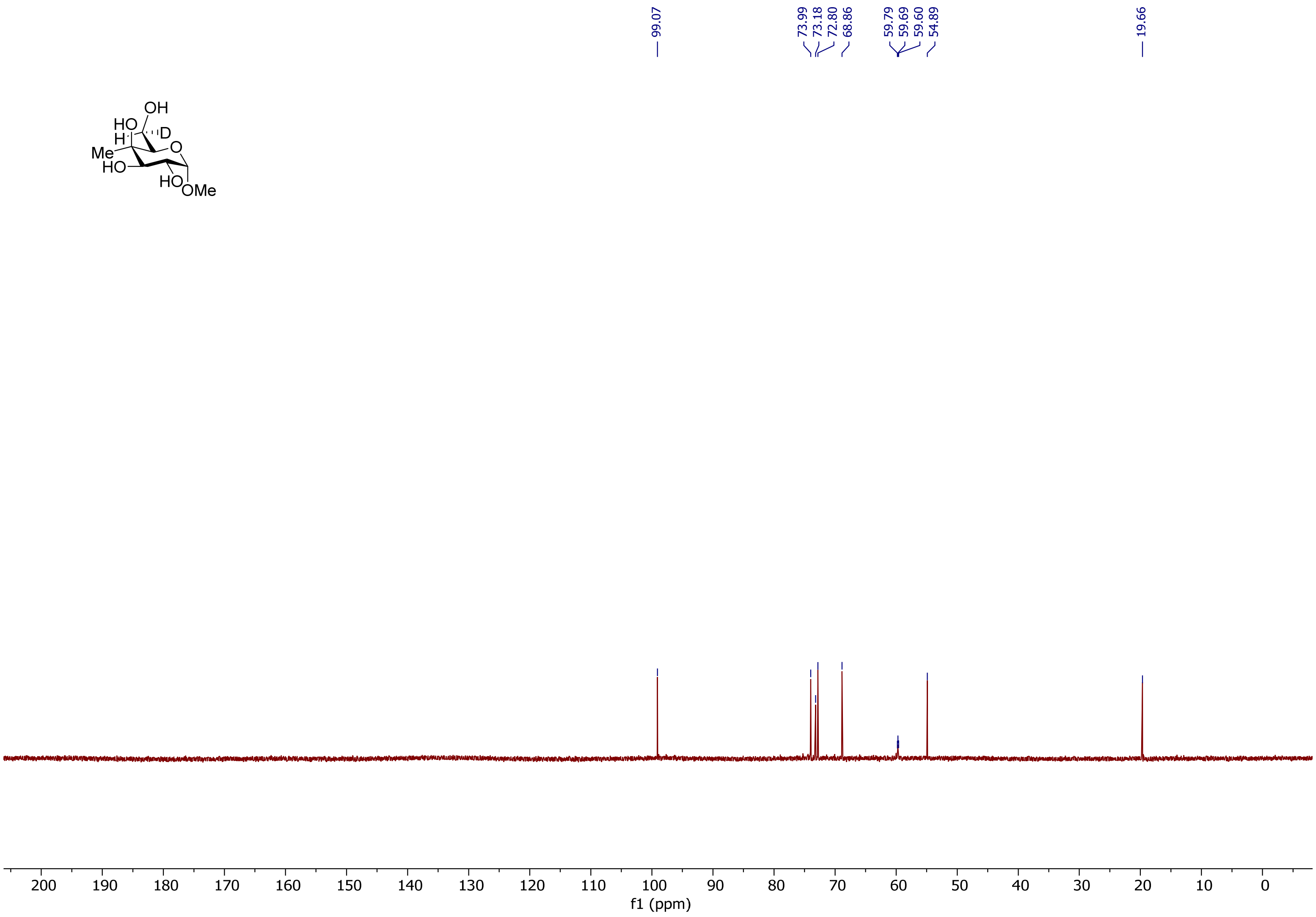

^{1}H NMR (900 MHz, D_2O) spectrum of methyl 4-*C*-methyl-6-(*S*)-deuterio-*α*-D-glucopyranoside (23-D_1)

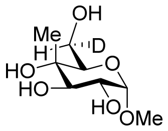

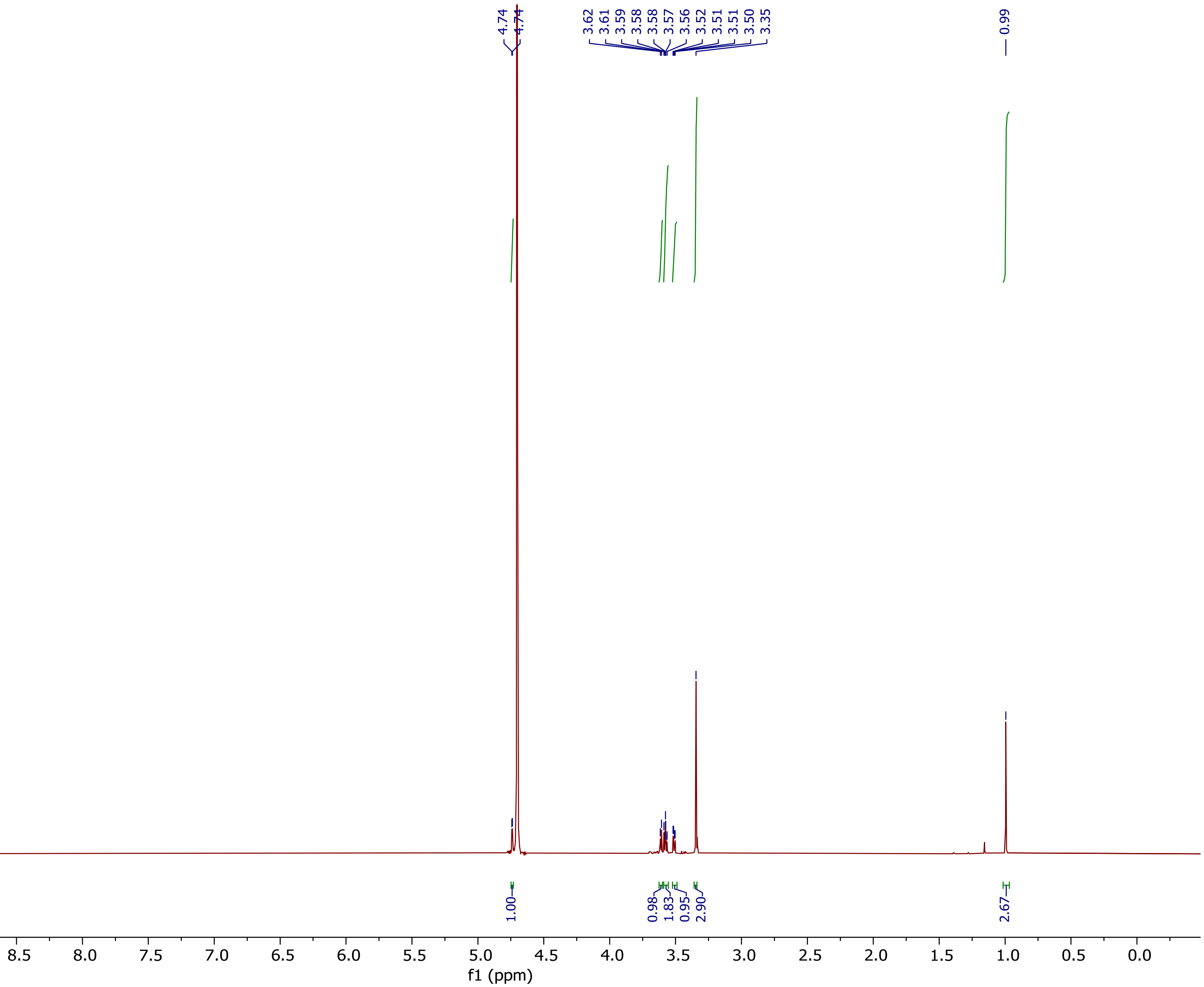

$^{13}C\{^1H\}$ NMR (226 MHz, D_2O) spectrum of methyl 4-*C*-methyl-6-(*S*)-deuterio-*α*-D-glucopyranoside (23-D_1)

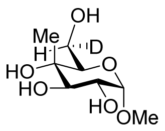

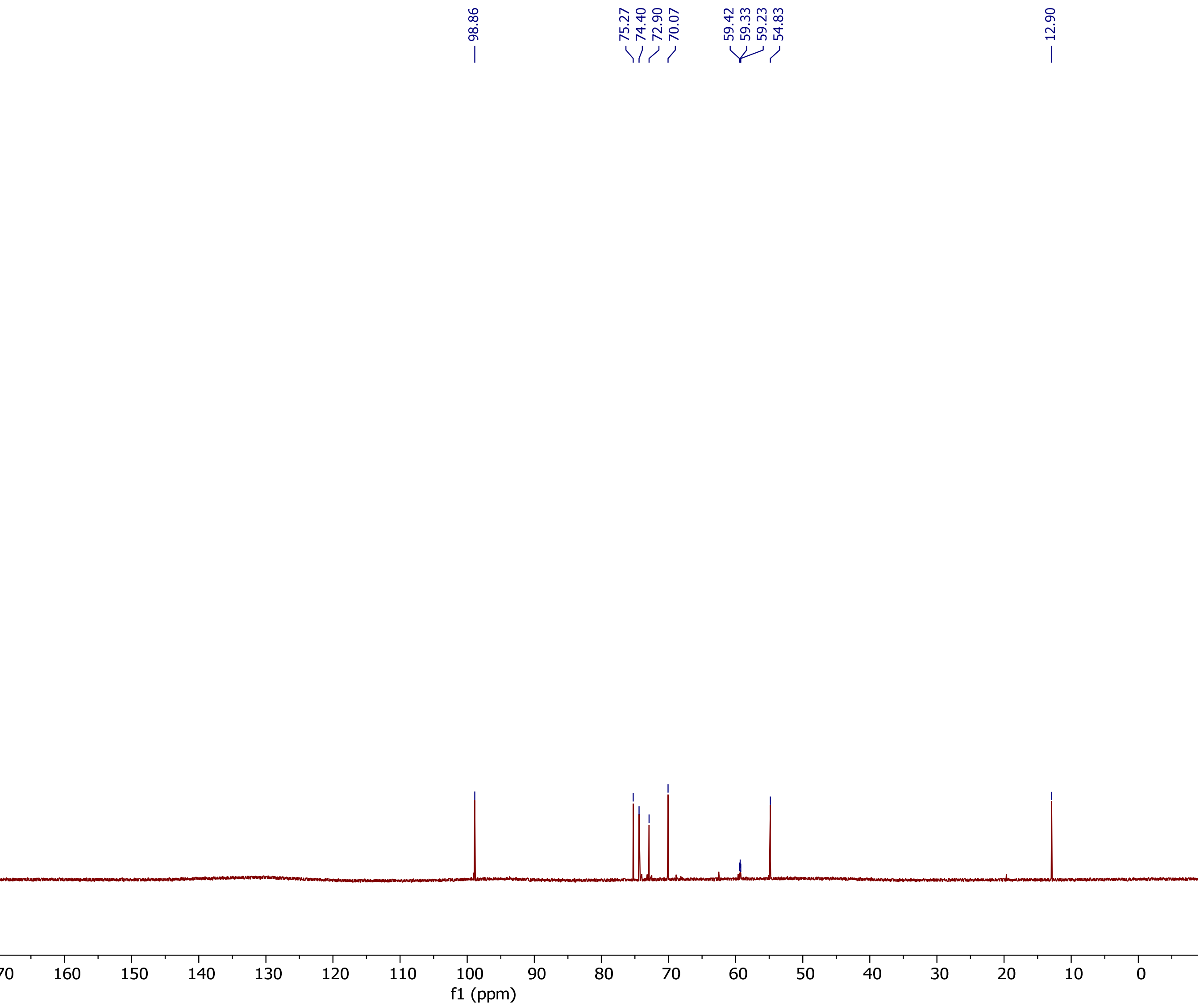

^1H NMR (500 MHz, $CDCl_3$) spectrum of methyl 2,3,6-tri-*O*-benzyl-4-*C*-methyl-*α*-D-galactopyranoside (20)

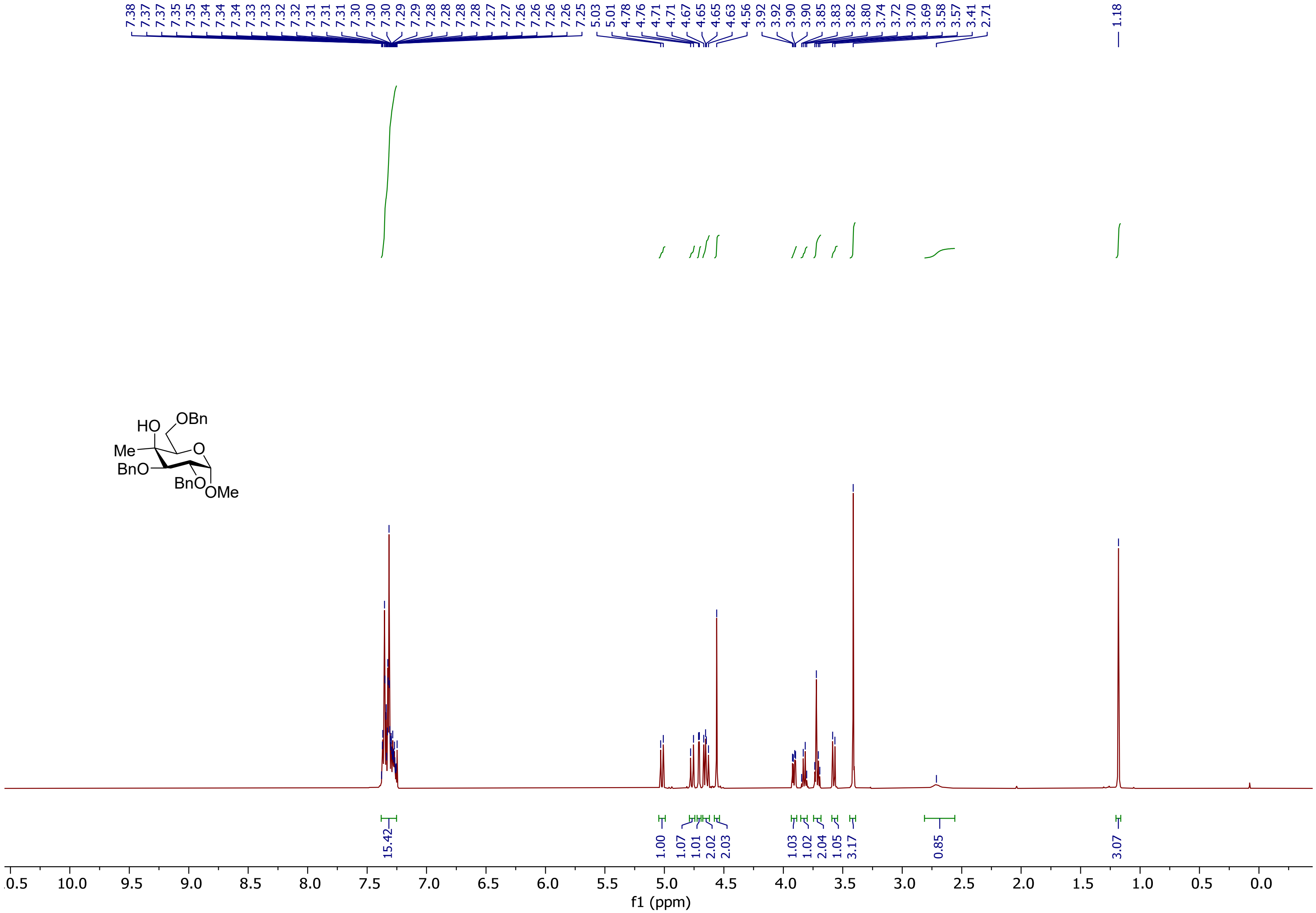

$^{13}C\{^1H\}$ NMR (126 MHz, $CDCl_3$) spectrum of methyl 2,3,6-tri-*O*-benzyl-4-*C*-methyl-*α*-D-galactopyranoside (**20**)

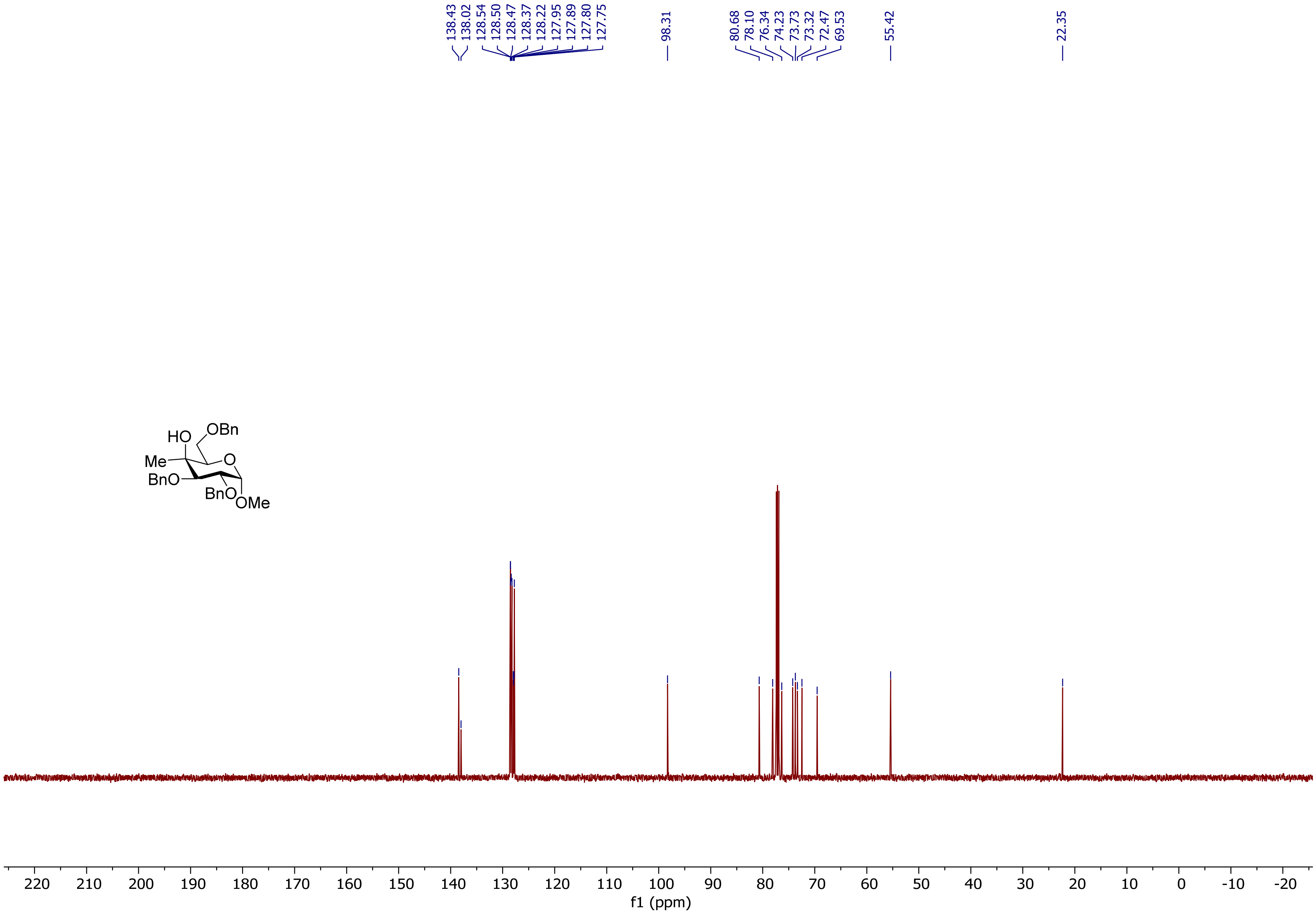

^{1}H NMR (600 MHz, C_6D_6) spectrum of methyl 2,3,6-tri-*O*-benzyl-4-*C*-methyl-*α*-D-galactopyranoside (20)

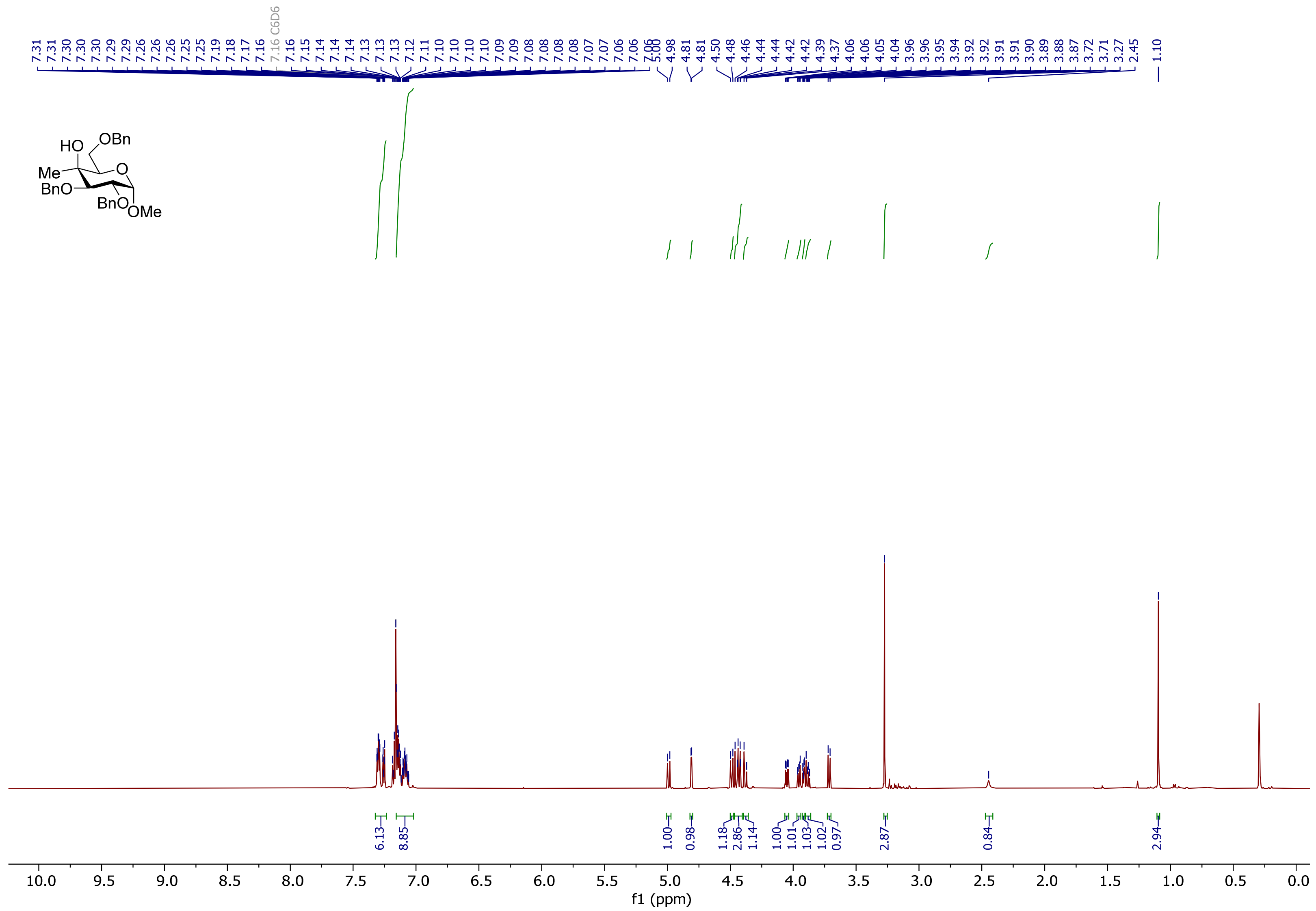

$^{13}C\{^{1}H\}$ NMR (151 MHz, C_6D_6) spectrum of methyl 2,3,6-tri-*O*-benzyl-4-*C*-methyl-α-D-galactopyranoside (20)

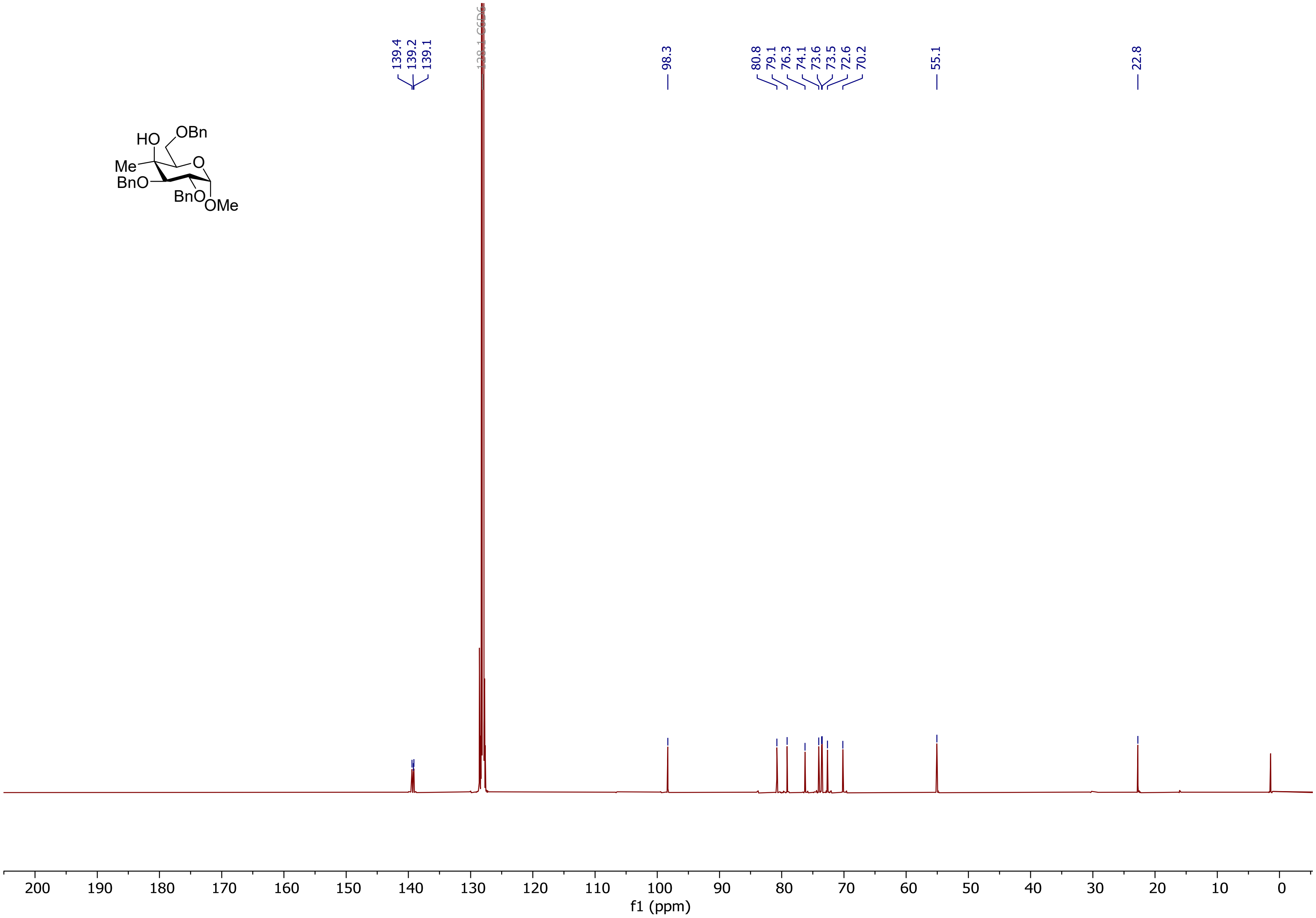

^{1}H NMR (500 MHz, CDCl$_3$) spectrum of methyl 2,3,6-tri-*O*-benzyl-4-*C*-methyl-*α*-D-glucopyranoside (21)

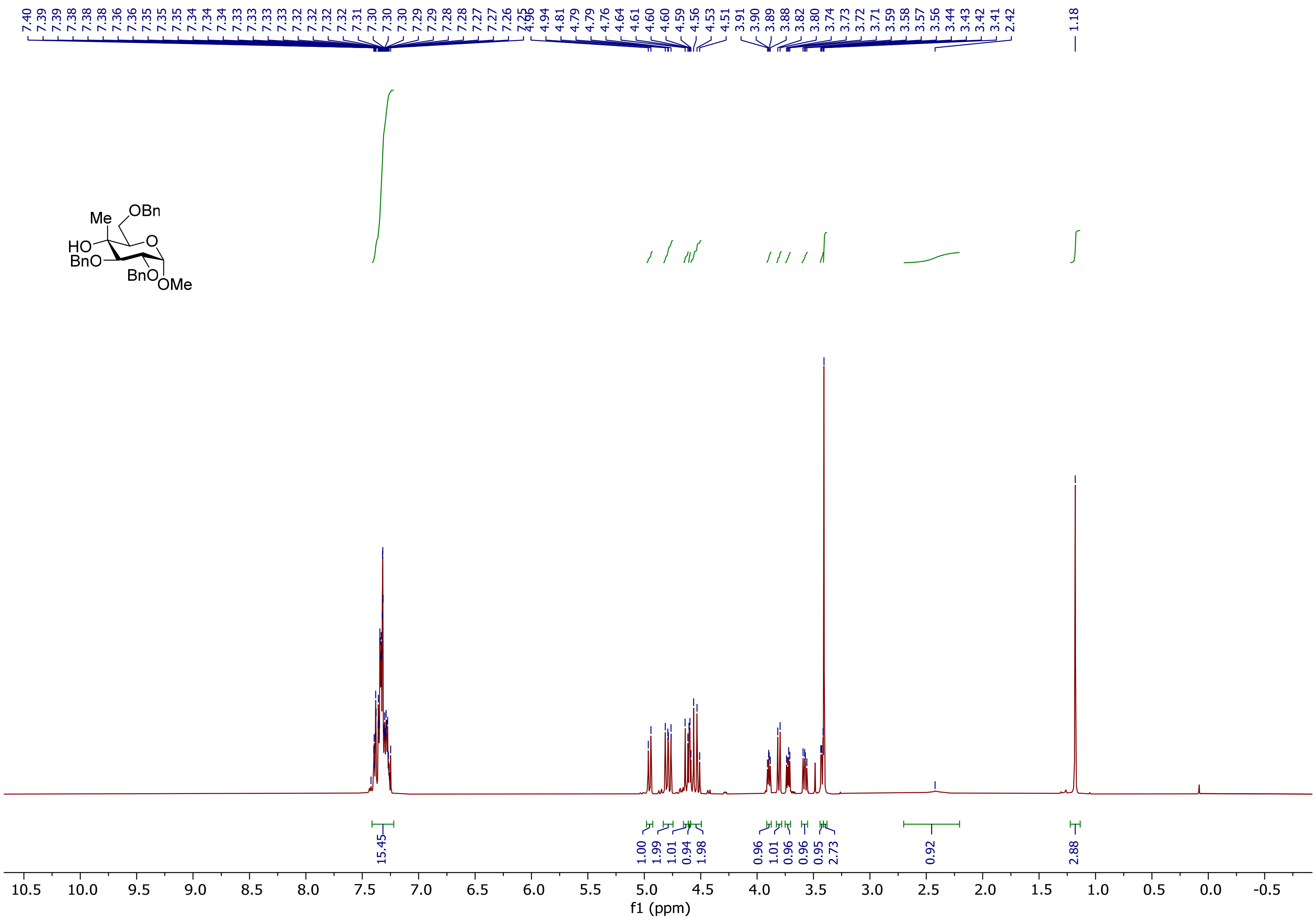

$^{13}C\{^{1}H\}$ NMR (126 MHz, $CDCl_3$) spectrum of methyl 2,3,6-tri-*O*-benzyl-4-*C*-methyl-*α*-D-glucopyranoside (21)

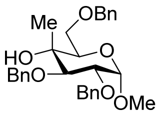

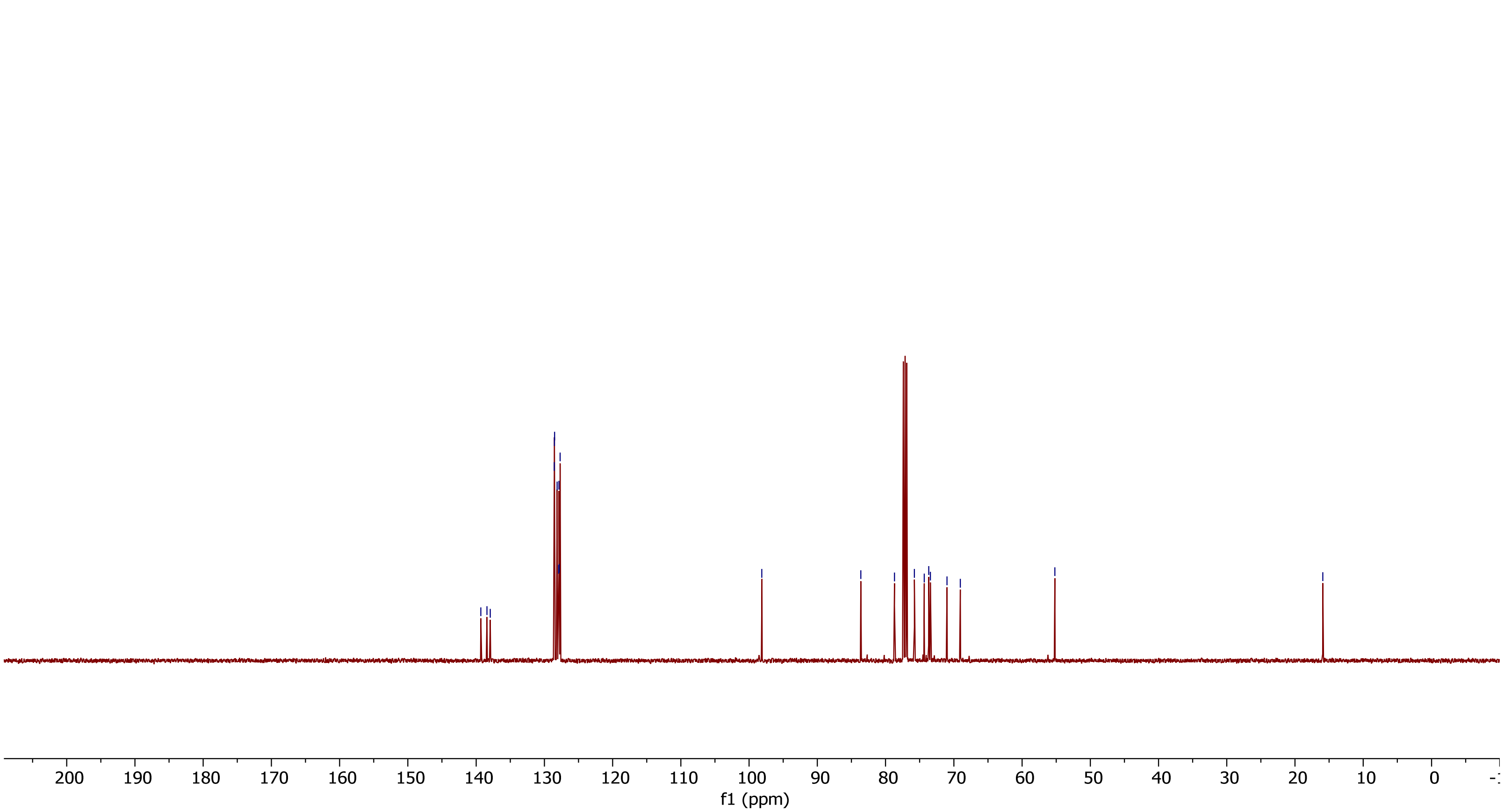

^{1}H NMR (900 MHz, D_2O) spectrum of methyl 4-*C*-methyl-*α*-D-galactopyranoside (22)

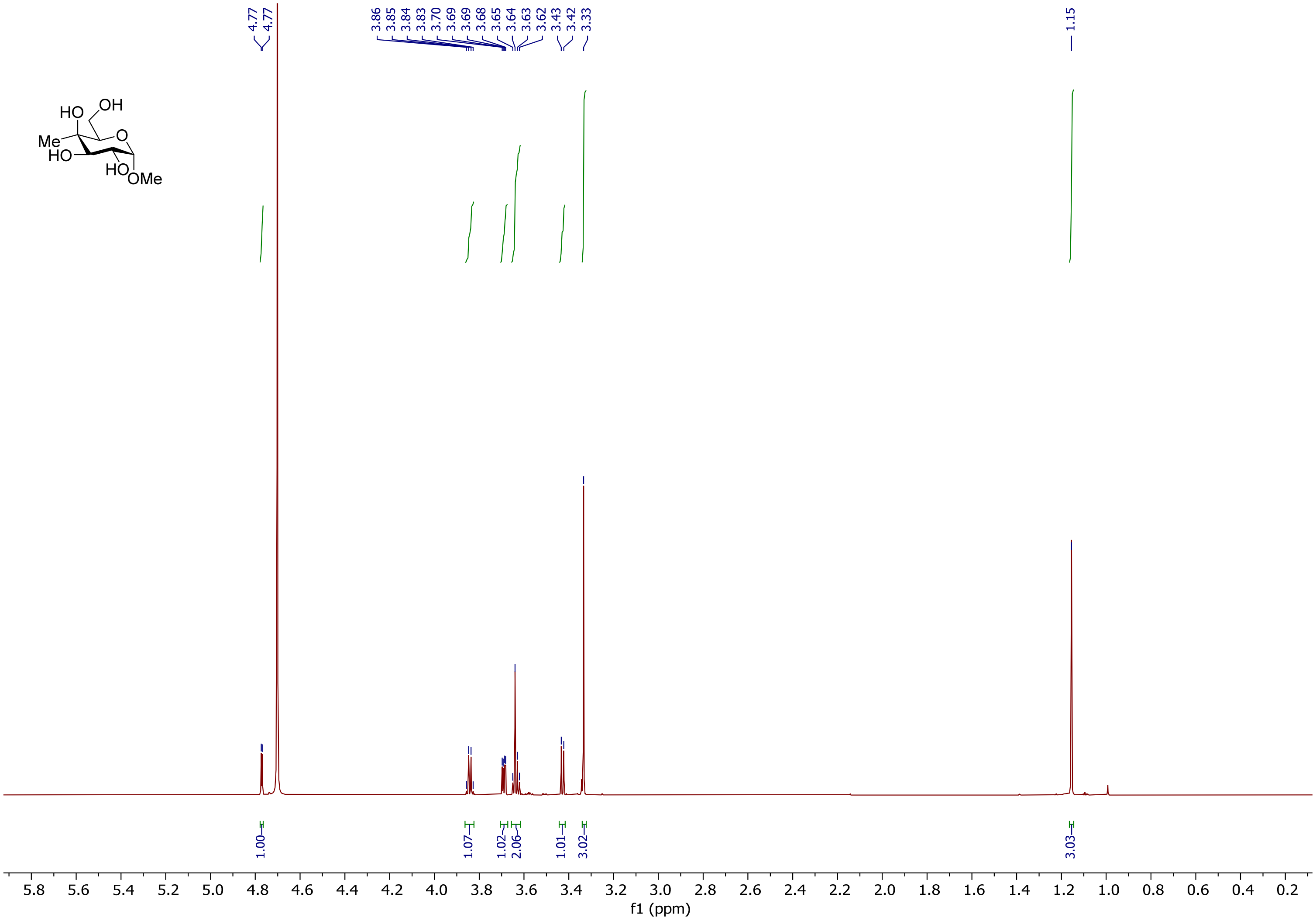

$^{13}C\{^{1}H\}$ NMR (226 MHz, D_2O) spectrum of methyl 4-*C*-methyl-*α*-D-galactopyranoside (22)

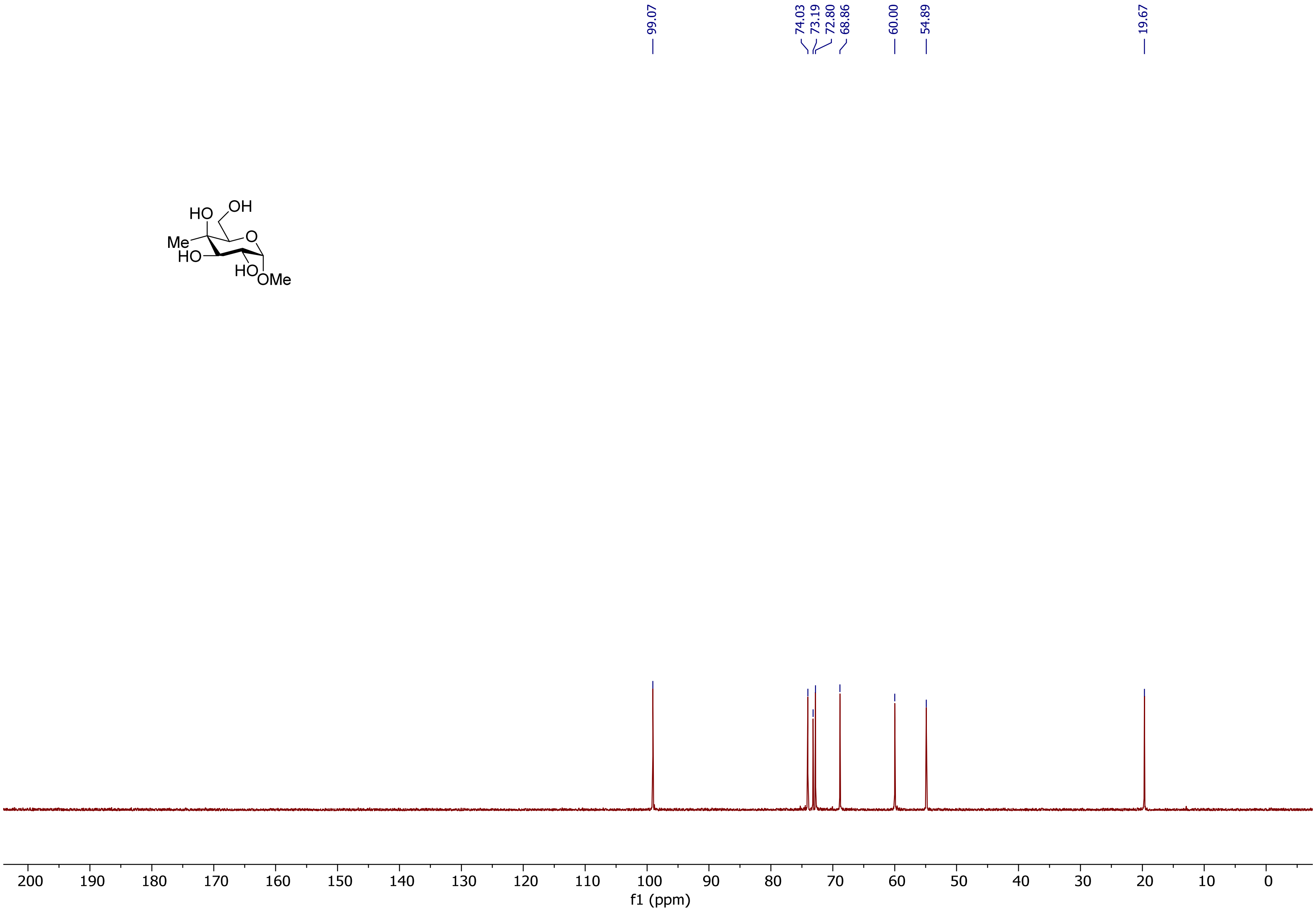

^{1}H NMR (900 MHz, D_2O) spectrum of methyl 4-*C*-methyl-*α*-D-glucopyranoside (23)

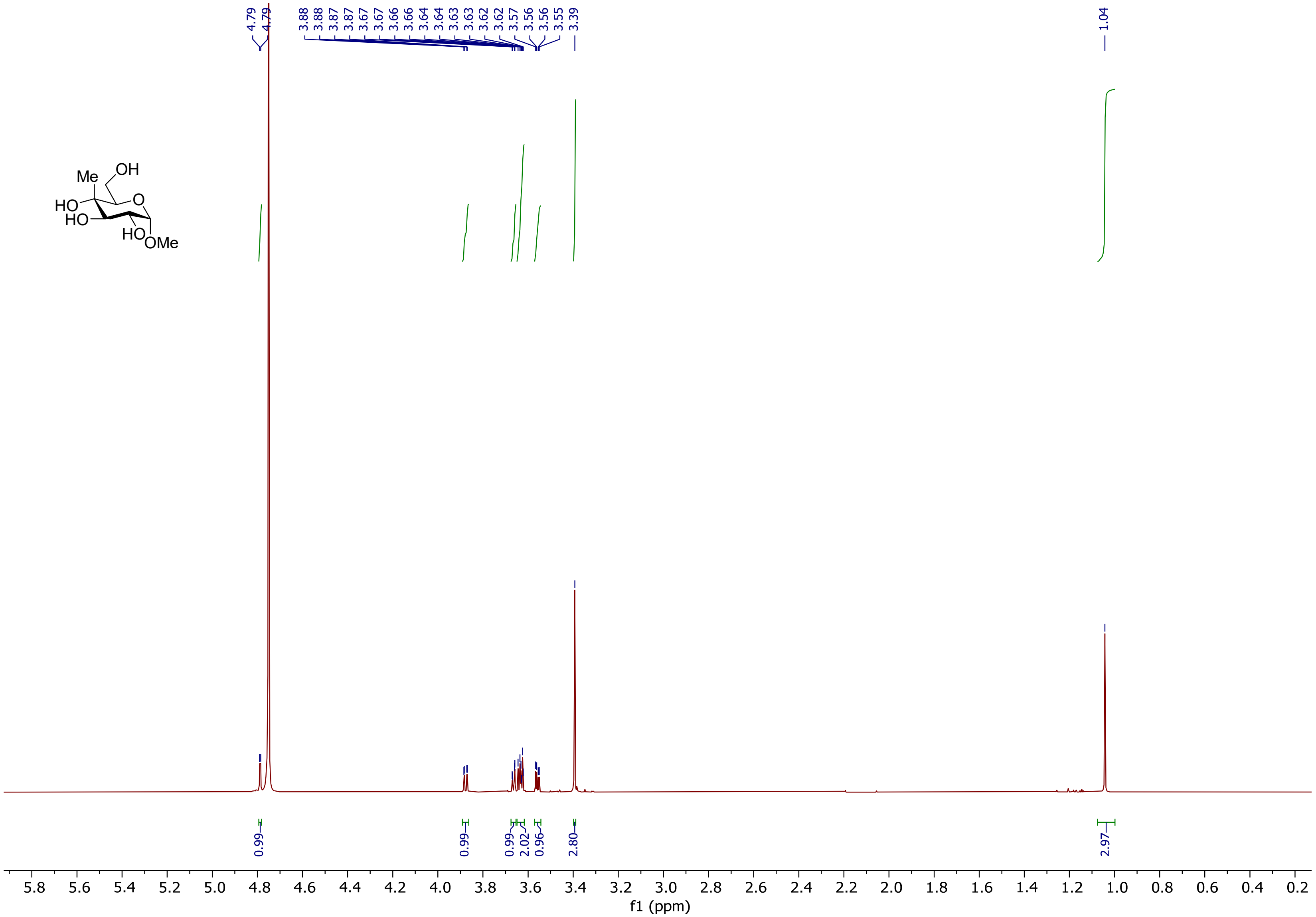

$^{13}C\{^{1}H\}$ NMR (226 MHz, D_2O) spectrum of methyl 4-*C*-methyl-*α*-D-glucopyranoside (23)

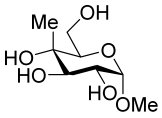

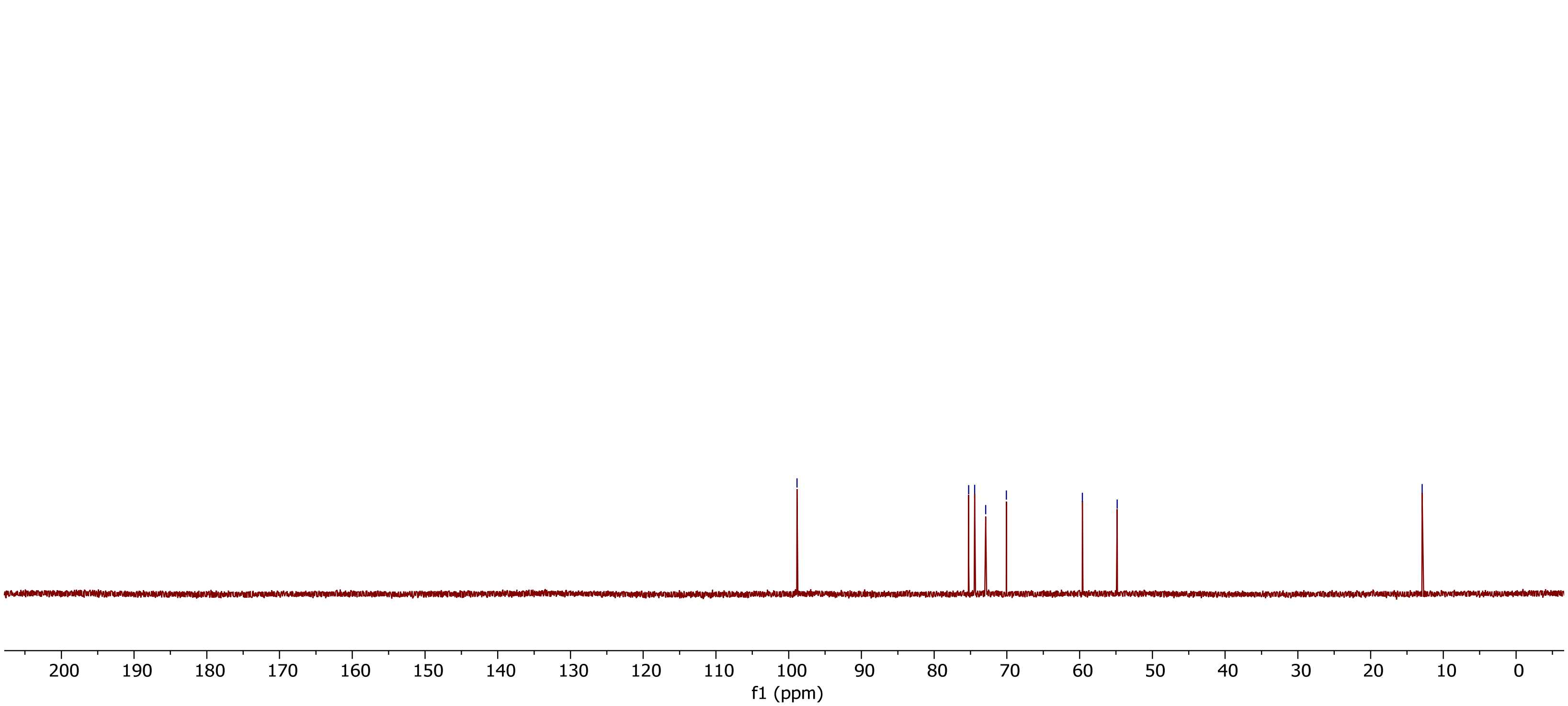

^{1}H NMR (500 MHz, $CDCl_3$) spectrum of *p*-methylphenyl 2,3,6-tri-*O*-benzyl-1-thio-*β*-D-glucopyranoside (27)

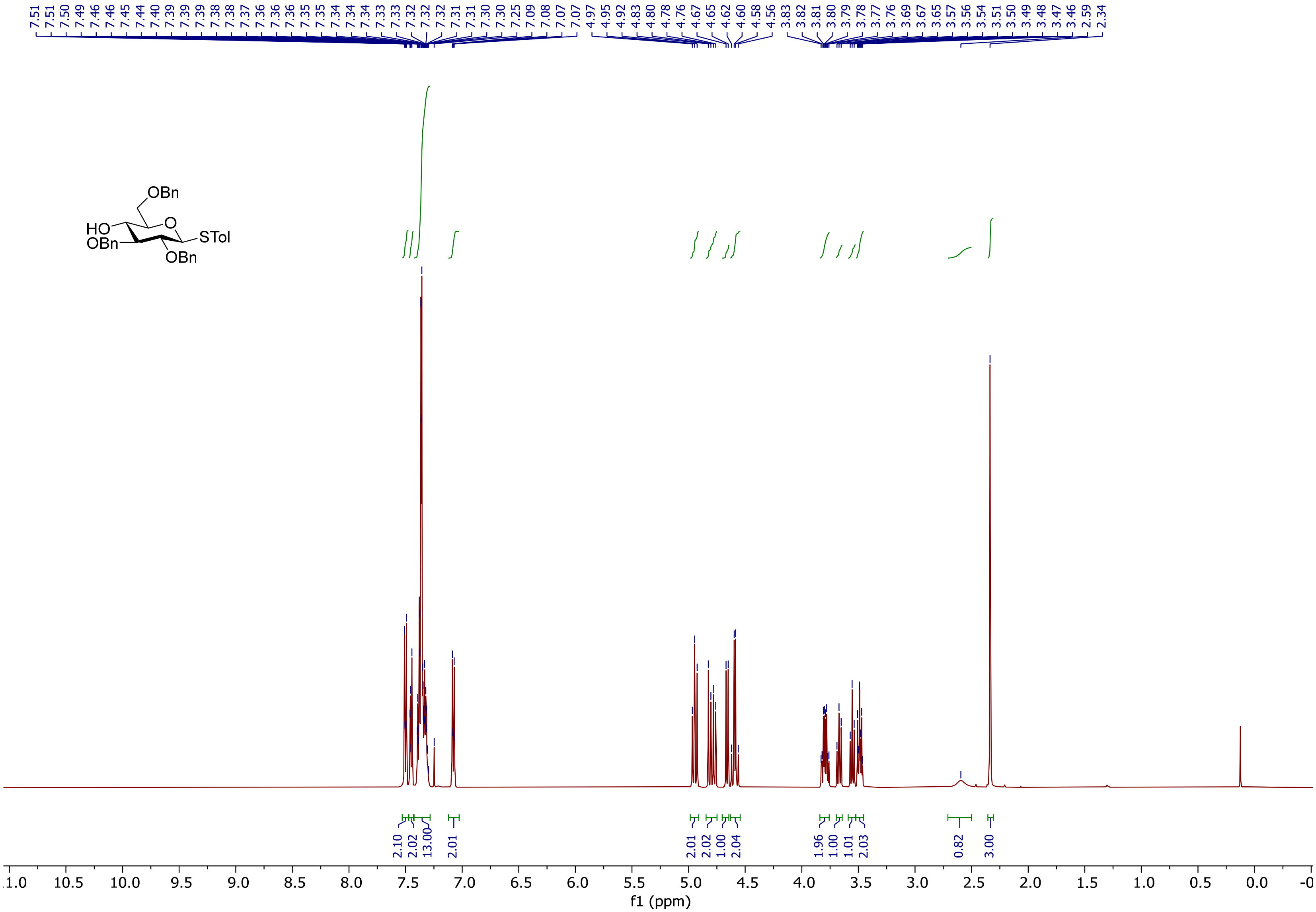

$^{13}C\{^1H\}$ NMR (126 MHz, $CDCl_3$) spectrum of *p*-methylphenyl 2,3,6-tri-*O*-benzyl-1-thio-*β*-D-glucopyranoside (27)

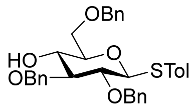

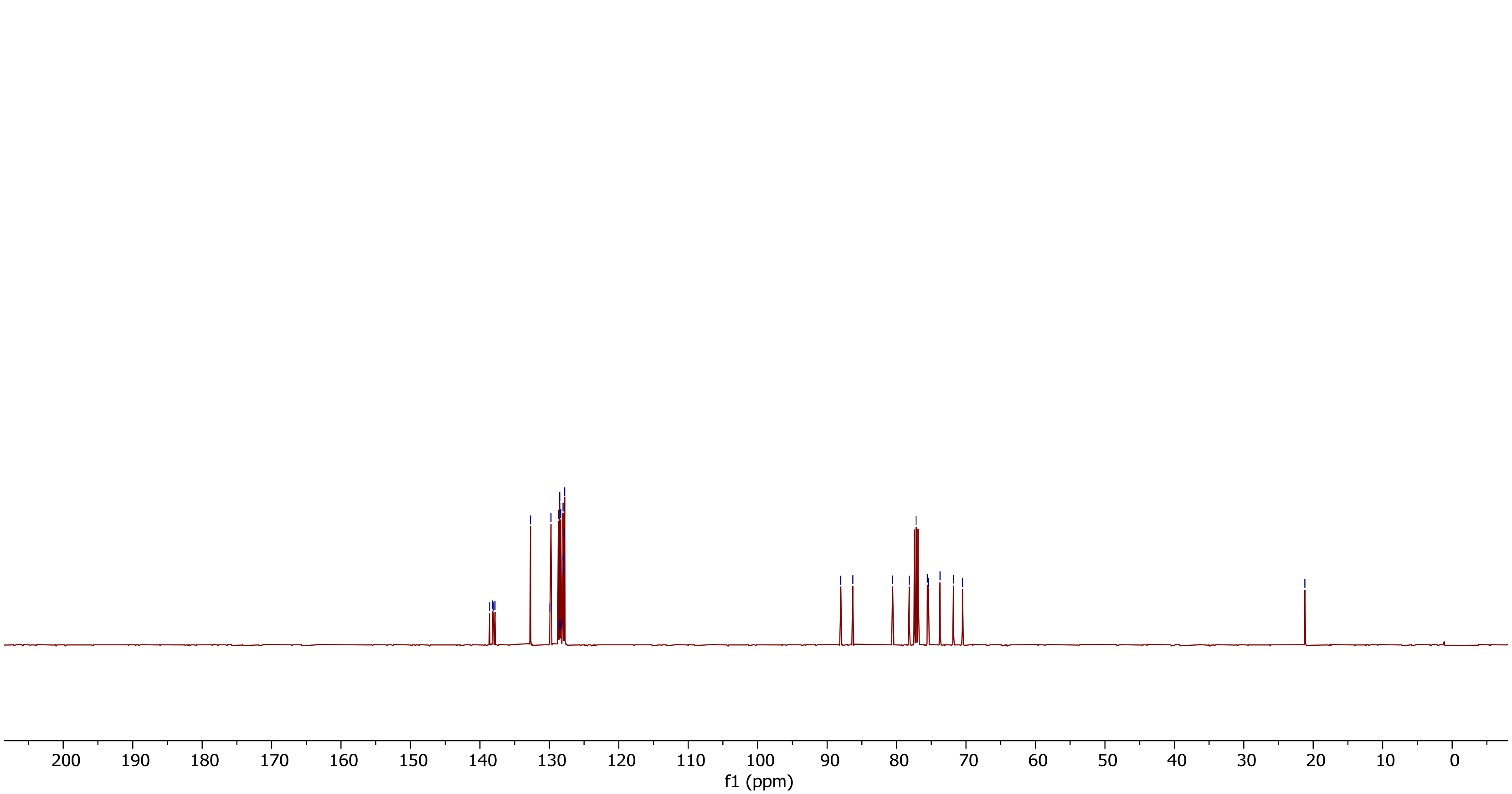

^{1}H NMR (500 MHz, $CDCl_3$) spectrum of *p*-methylphenyl 4-*O*-benzoyl-2,3,6-tri-*O*-benzyl-4-*C*-methyl-1-thio-*β*-D-galactopyranoside (3)

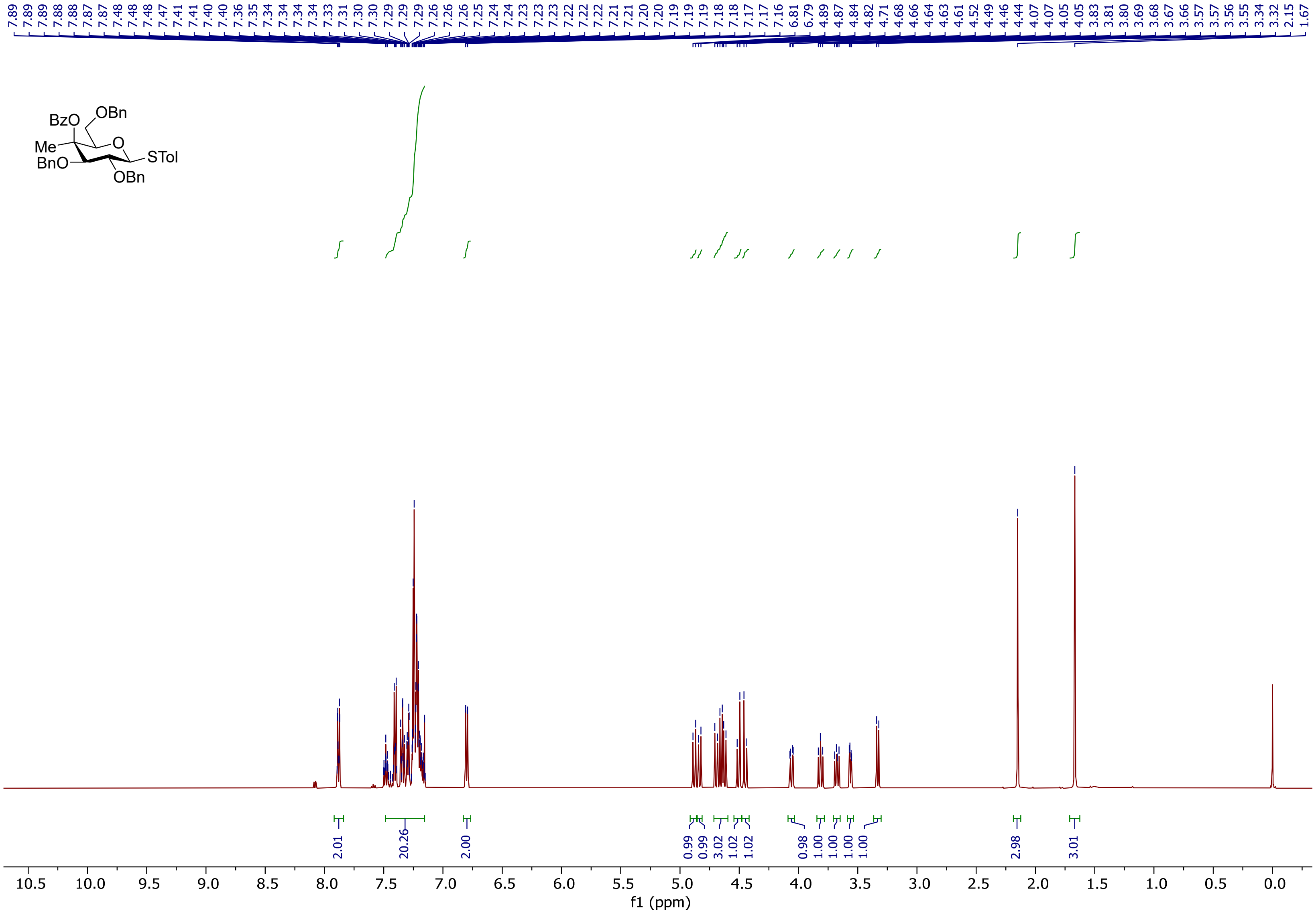

$^{13}C\{^{1}H\}$ NMR (126 MHz, $CDCl_3$) spectrum of *p*-methylphenyl 4-*O*-benzoyl-2,3,6-tri-*O*-benzyl-4-*C*-methyl-1-thio-*β*-D-galactopyranoside (3)

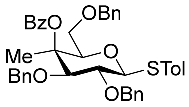

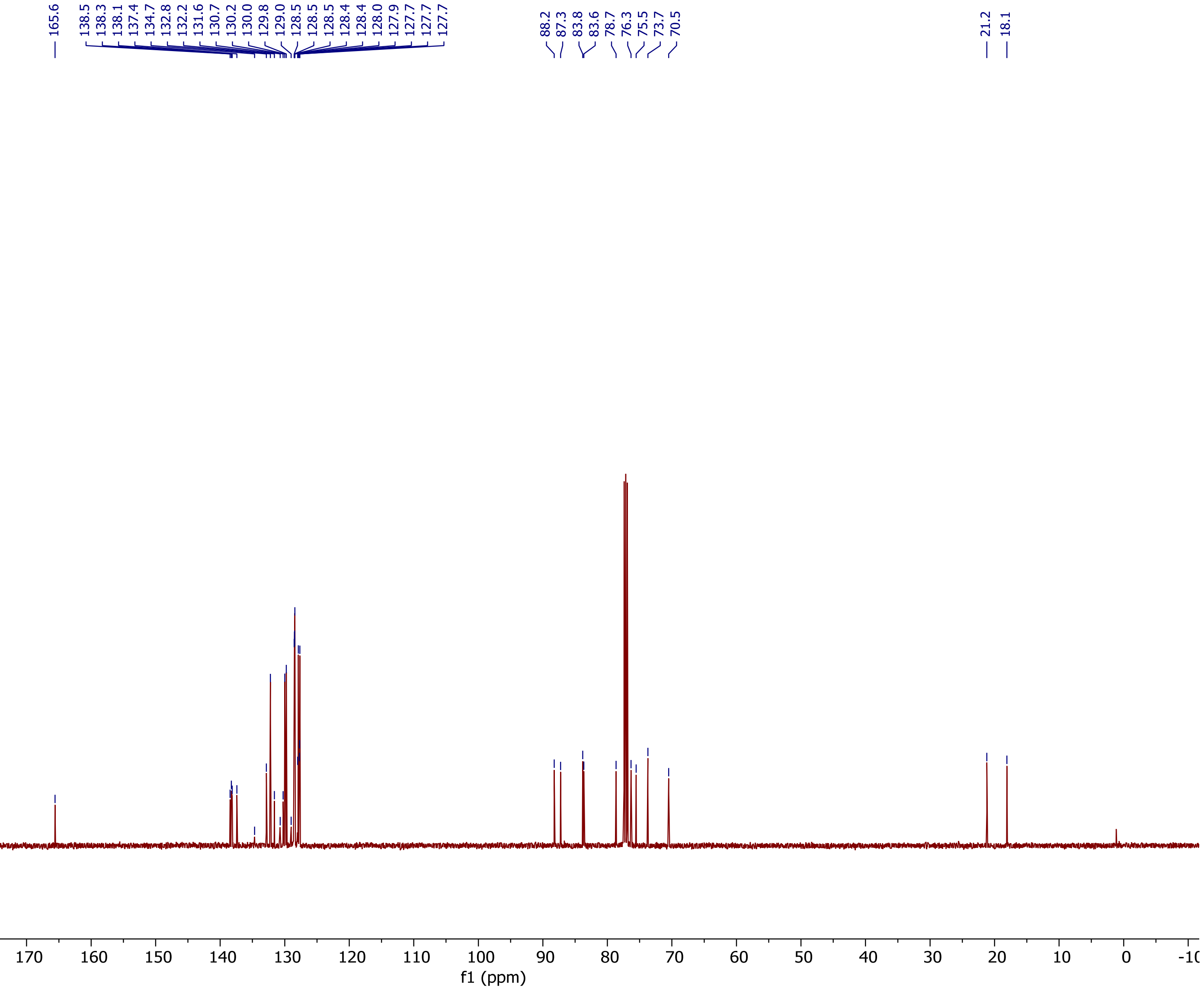

COSY NMR (500 MHz, CDCl$_3$) spectrum of *p*-methylphenyl 4-*O*-benzoyl-2,3,6-tri-*O*-benzyl-4-*C*-methyl-1-thio-*β*-D-galactopyranoside (3)

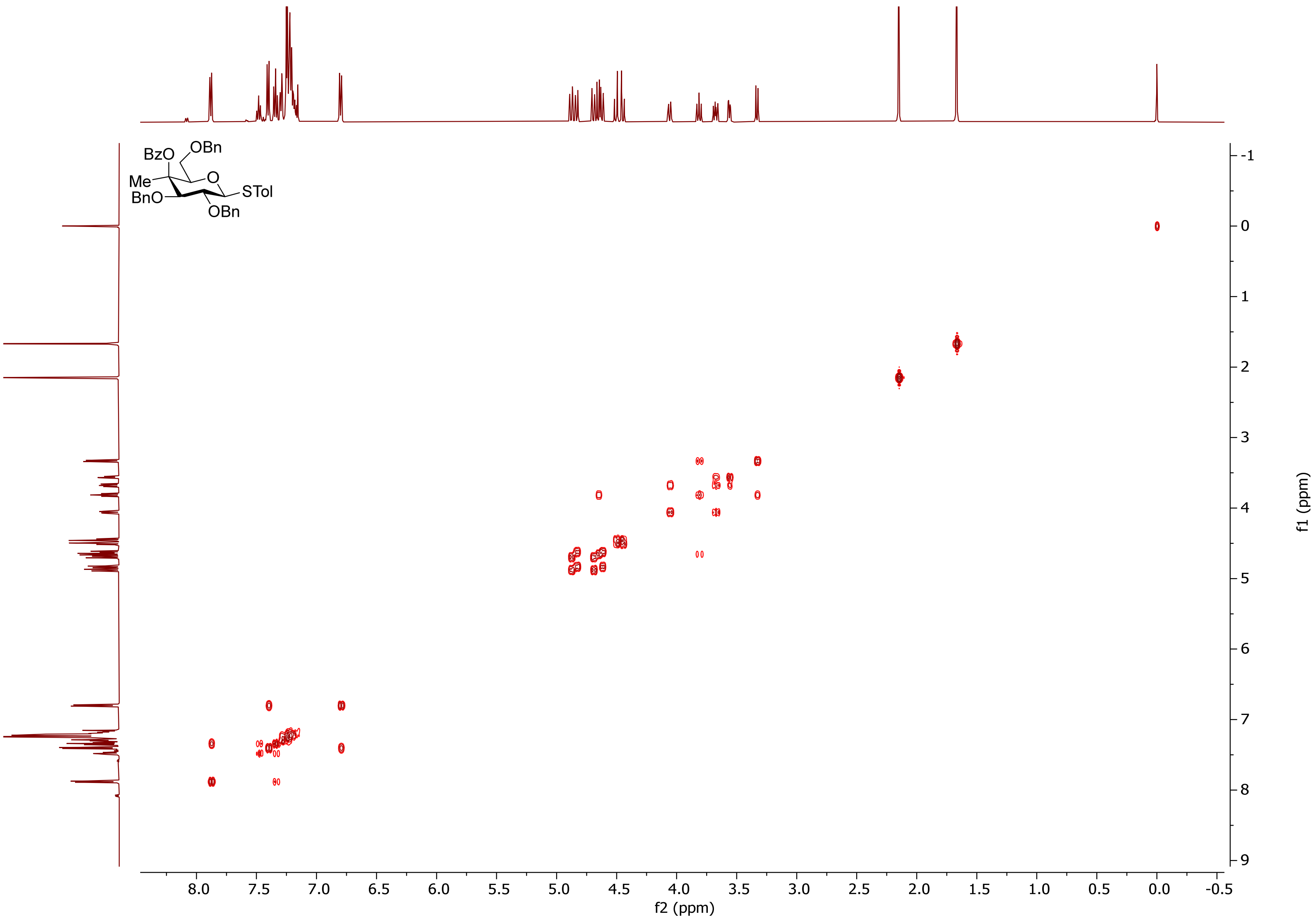

HSQC NMR (500 MHz, CDCl3) spectrum of *p*-methylphenyl 4-*O*-benzoyl-2,3,6-tri-*O*-benzyl-4-*C*-methyl-1-thio-*β*-D-galactopyranoside (3)

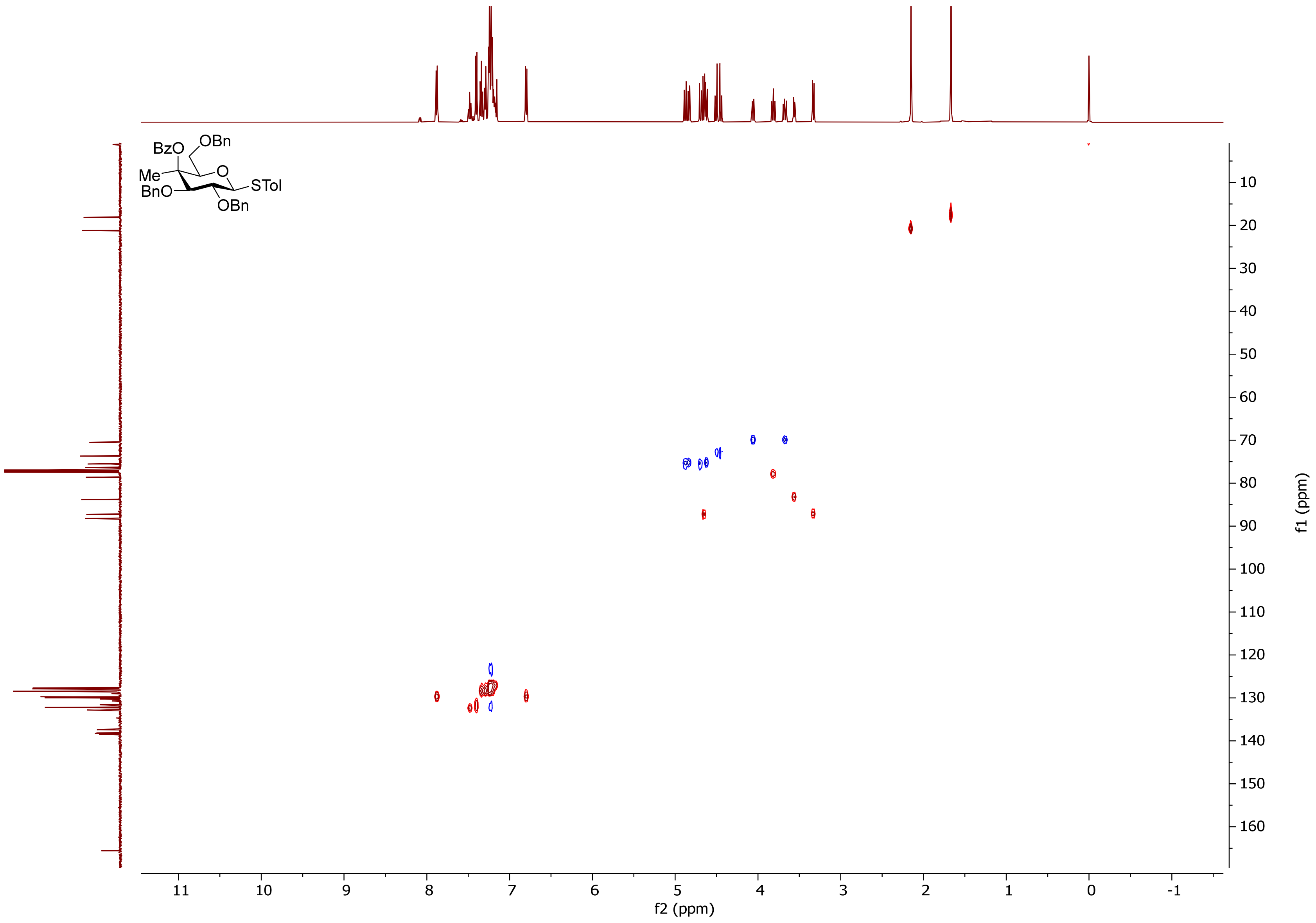

^{1}H NMR (500 MHz, $CDCl_3$) spectrum of *p*-methylphenyl 4-*O*-benzoyl-2,3,6-tri-*O*-benzyl-4-*C*-methyl-1-thio-*β*-D-glucopyranoside (28)

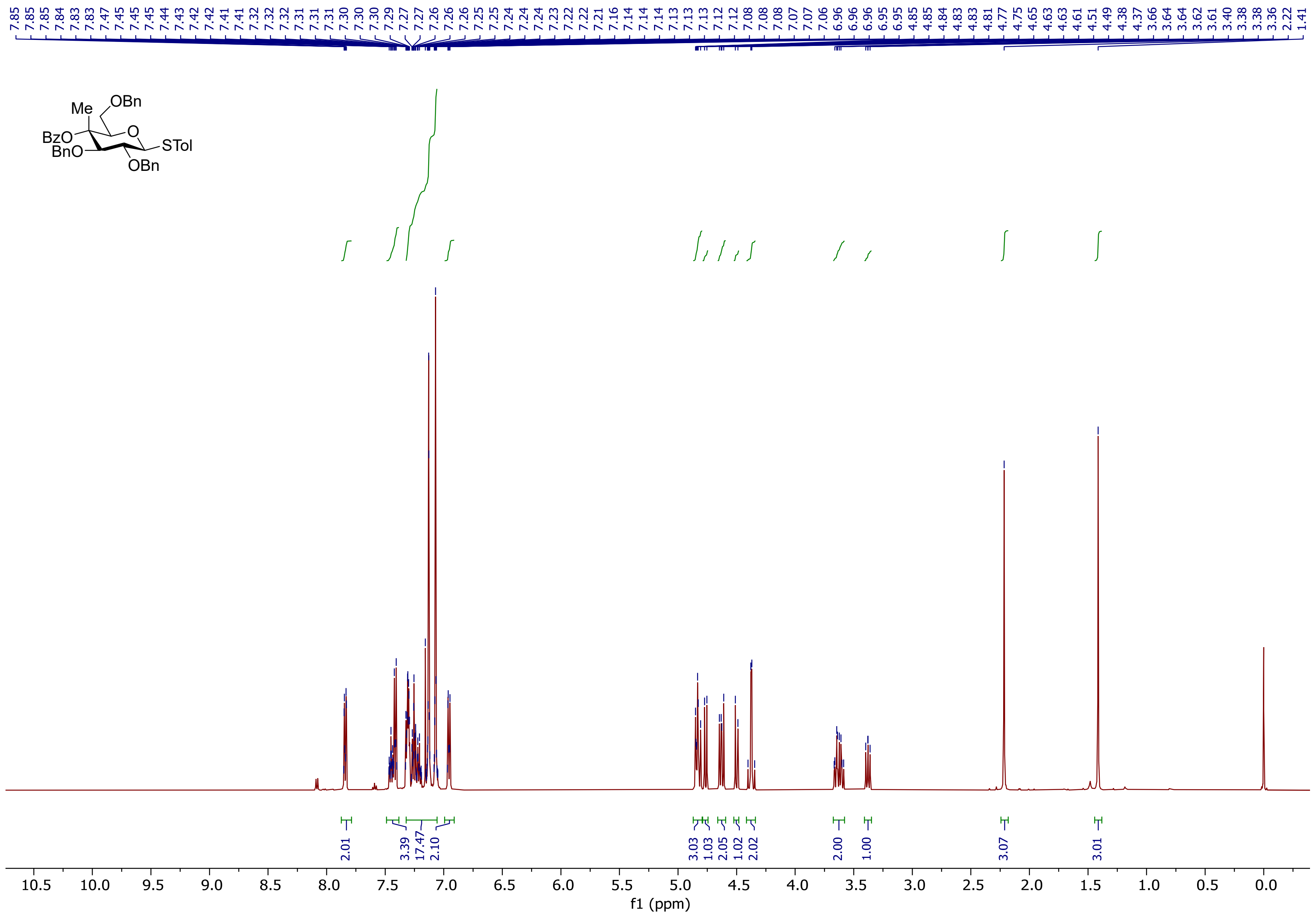

$^{13}C\{^{1}H\}$ NMR (126 MHz, $CDCl_3$) spectrum of *p*-Methylphenyl 4-*O*-benzoyl-2,3,6-tri-*O*-benzyl-4-*C*-methyl-1-thio-*β*-D-glucopyranoside (28)

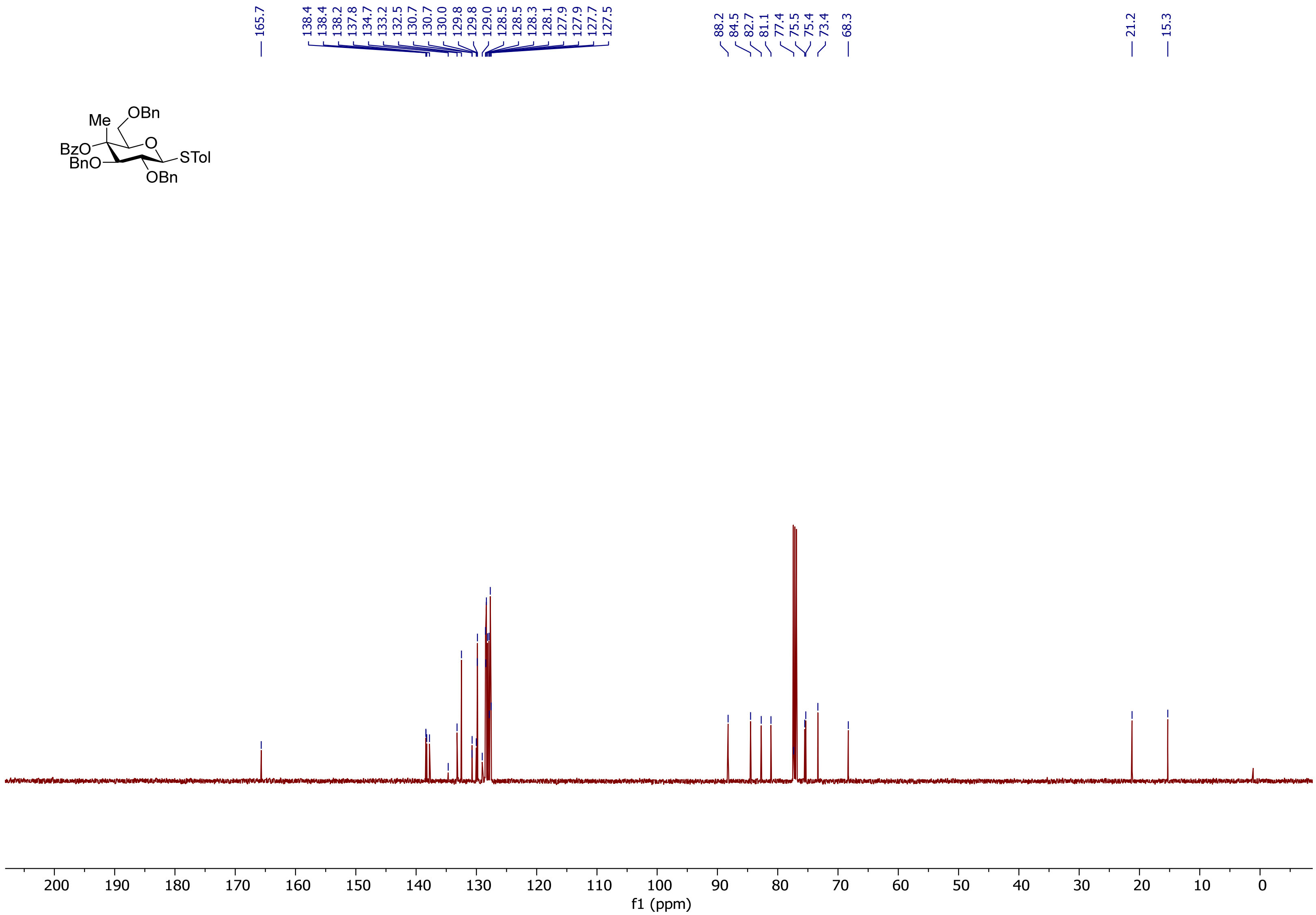

COSY NMR (500 MHz, CDCl$_3$) spectrum of *p*-Methylphenyl 4-*O*-benzoyl-2,3,6-tri-*O*-benzyl-4-*C*-methyl-1-thio-*β*-D-glucopyranoside (28)

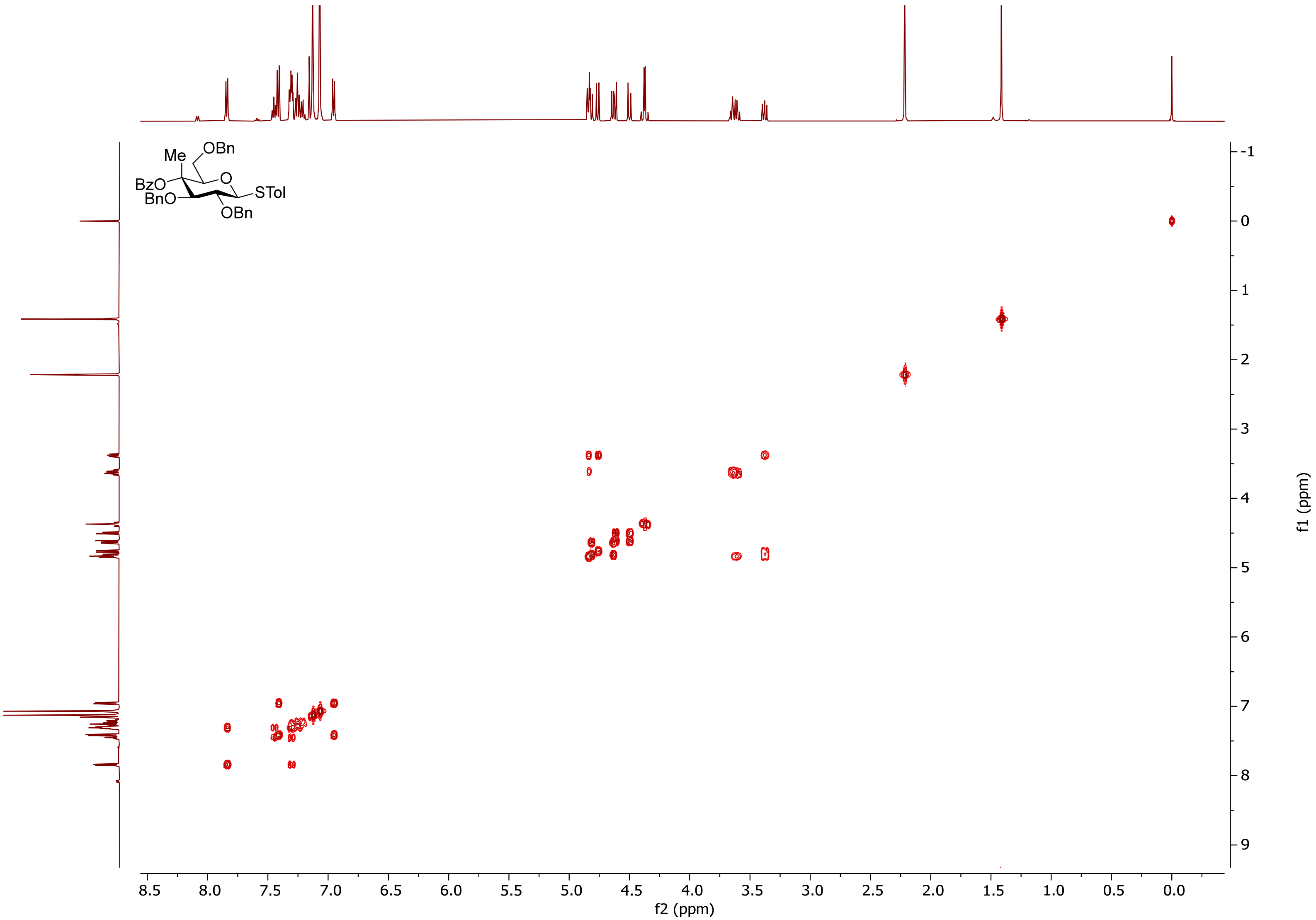

HSQC NMR (500 MHz, $CDCl_3$) spectrum of *p*-Methylphenyl 4-*O*-benzoyl-2,3,6-tri-*O*-benzyl-4-*C*-methyl-1-thio-*β*-D-glucopyranoside (28)

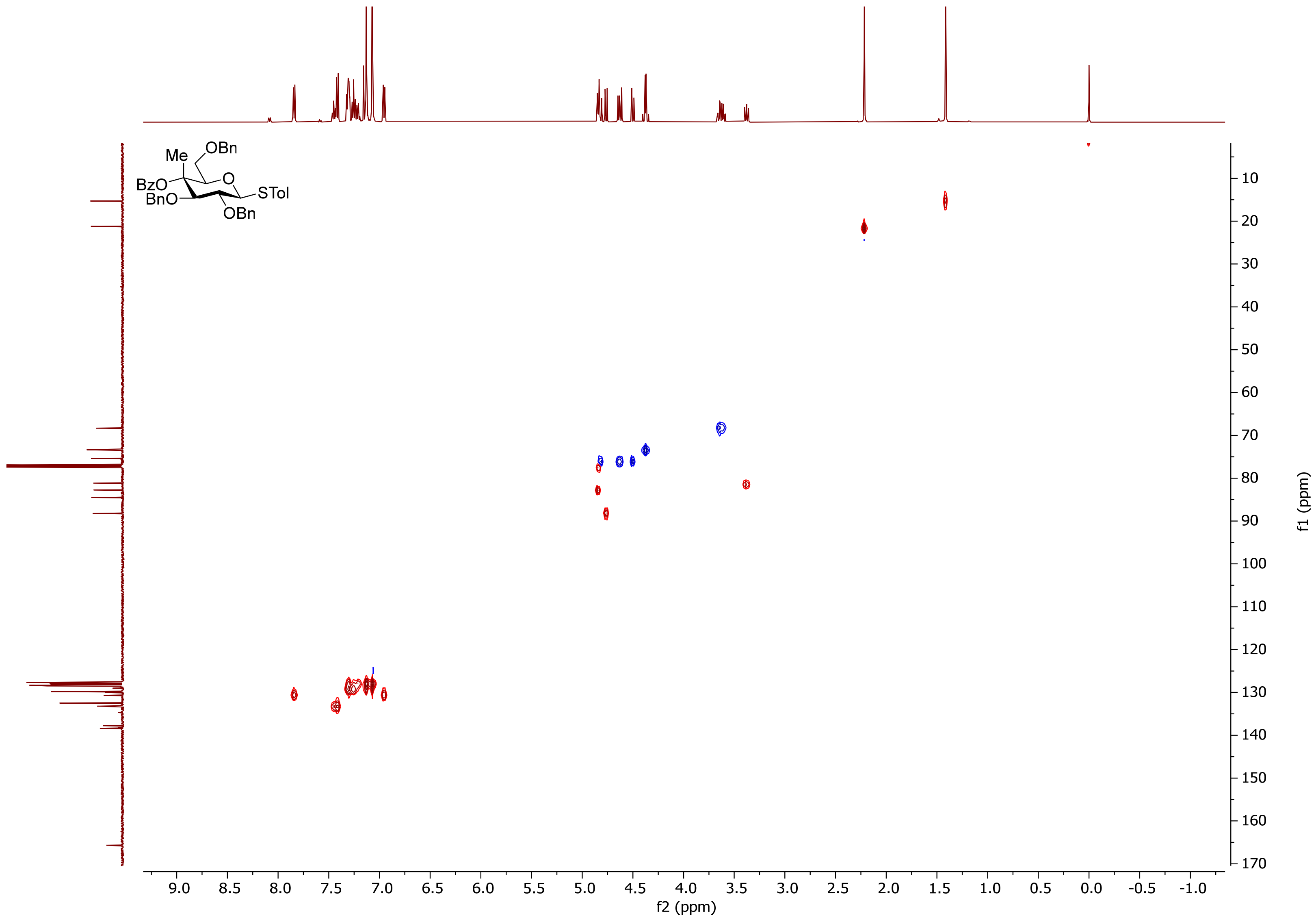

^{1}H NMR (500 MHz, CDCl$_3$) spectrum of *p*-methylphenyl 2,3,4,6-tetra-*O*-benzyl-4-*C*-methyl-1-thio-*β*-D-galactopyranoside (29)

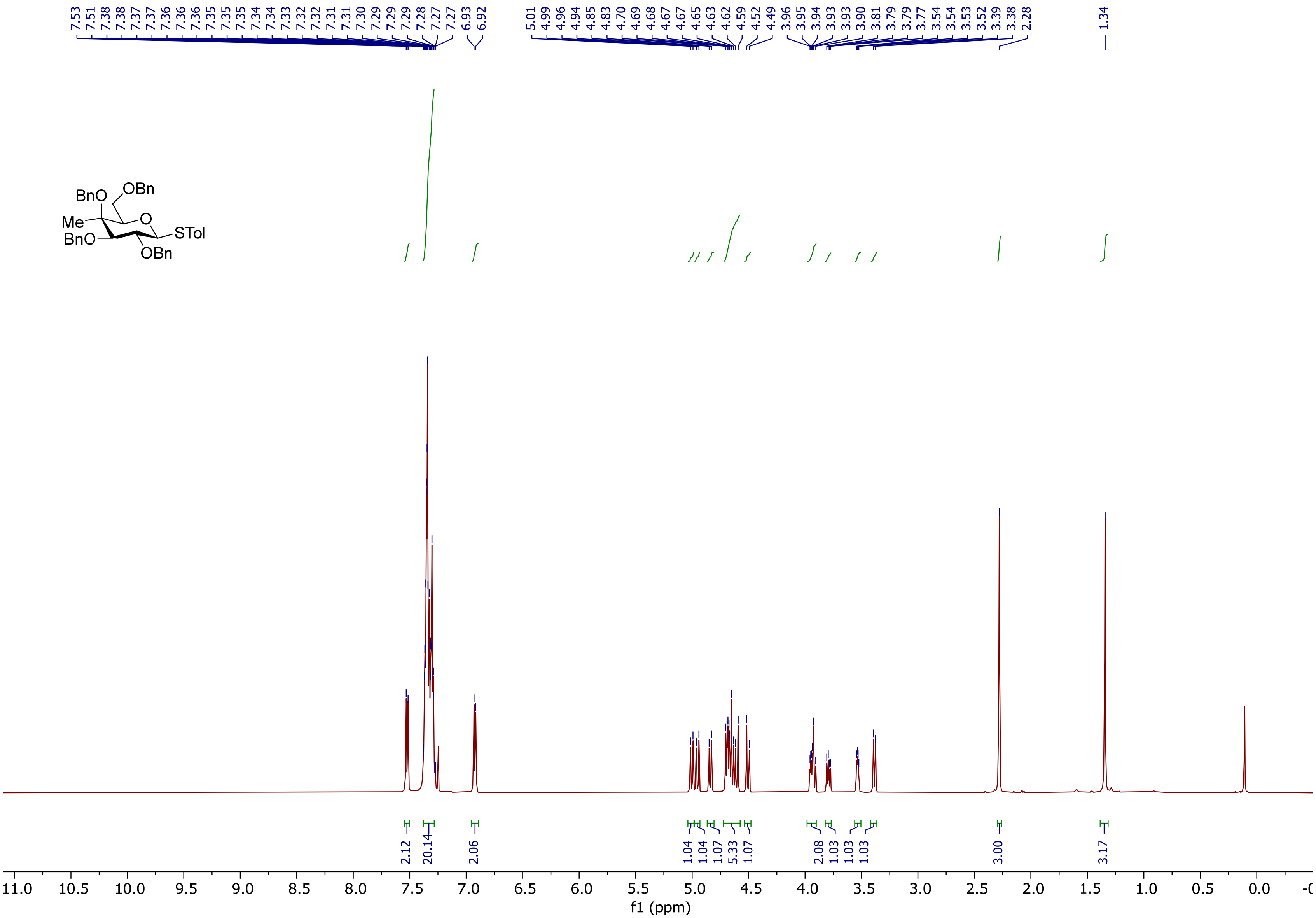

$^{13}C\{^1H\}$ NMR (126 MHz, $CDCl_3$) spectrum of *p*-methylphenyl 2,3,4,6-tetra-*O*-benzyl-4-*C*-methyl-1-thio-*β*-D-galactopyranoside (29)

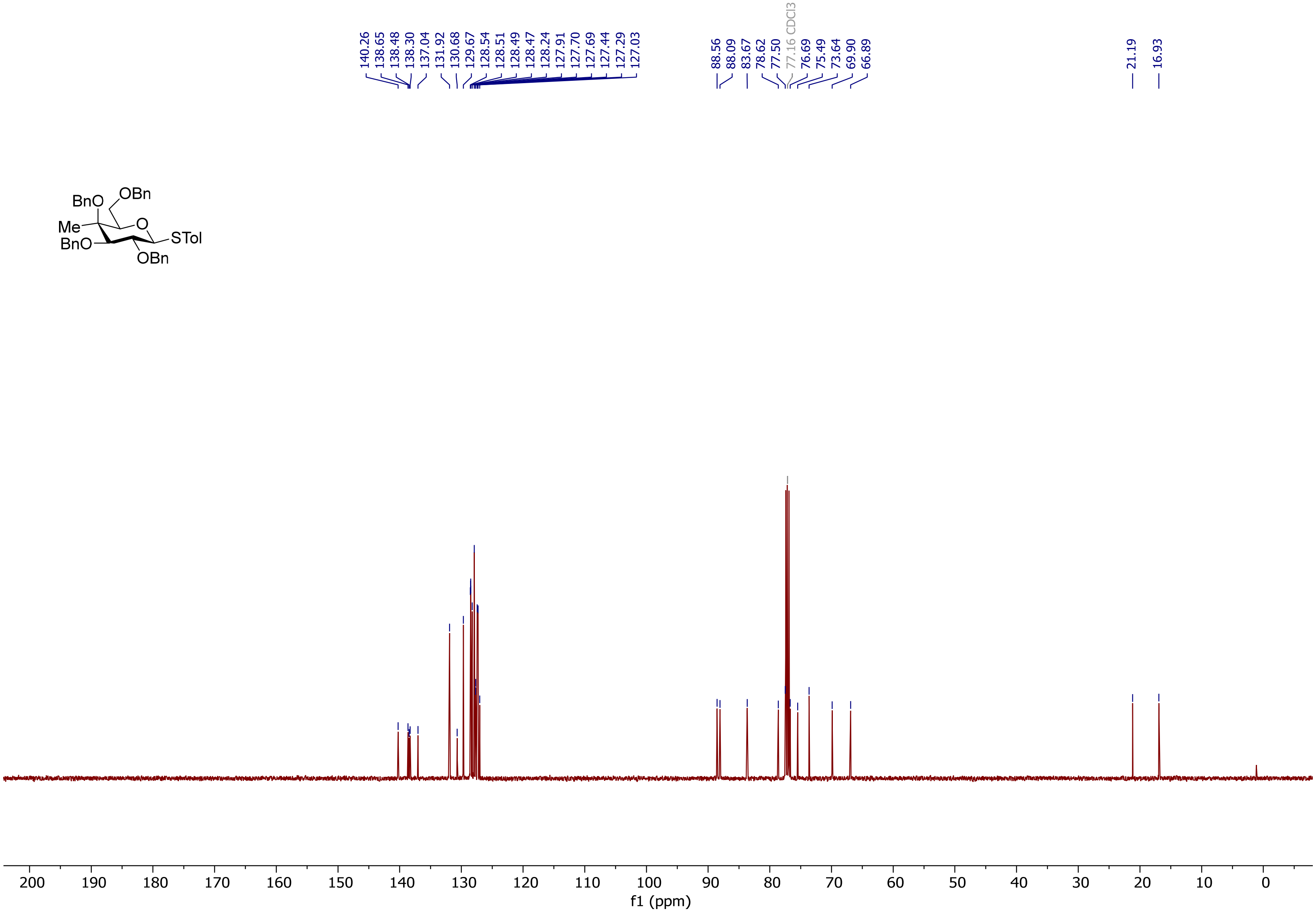

COSY (500 MHz, CDCl$_3$) spectrum of *p*-methylphenyl 2,3,4,6-tetra-*O*-benzyl-4-*C*-methyl-1-thio-*β*-D-galactopyranoside (29)

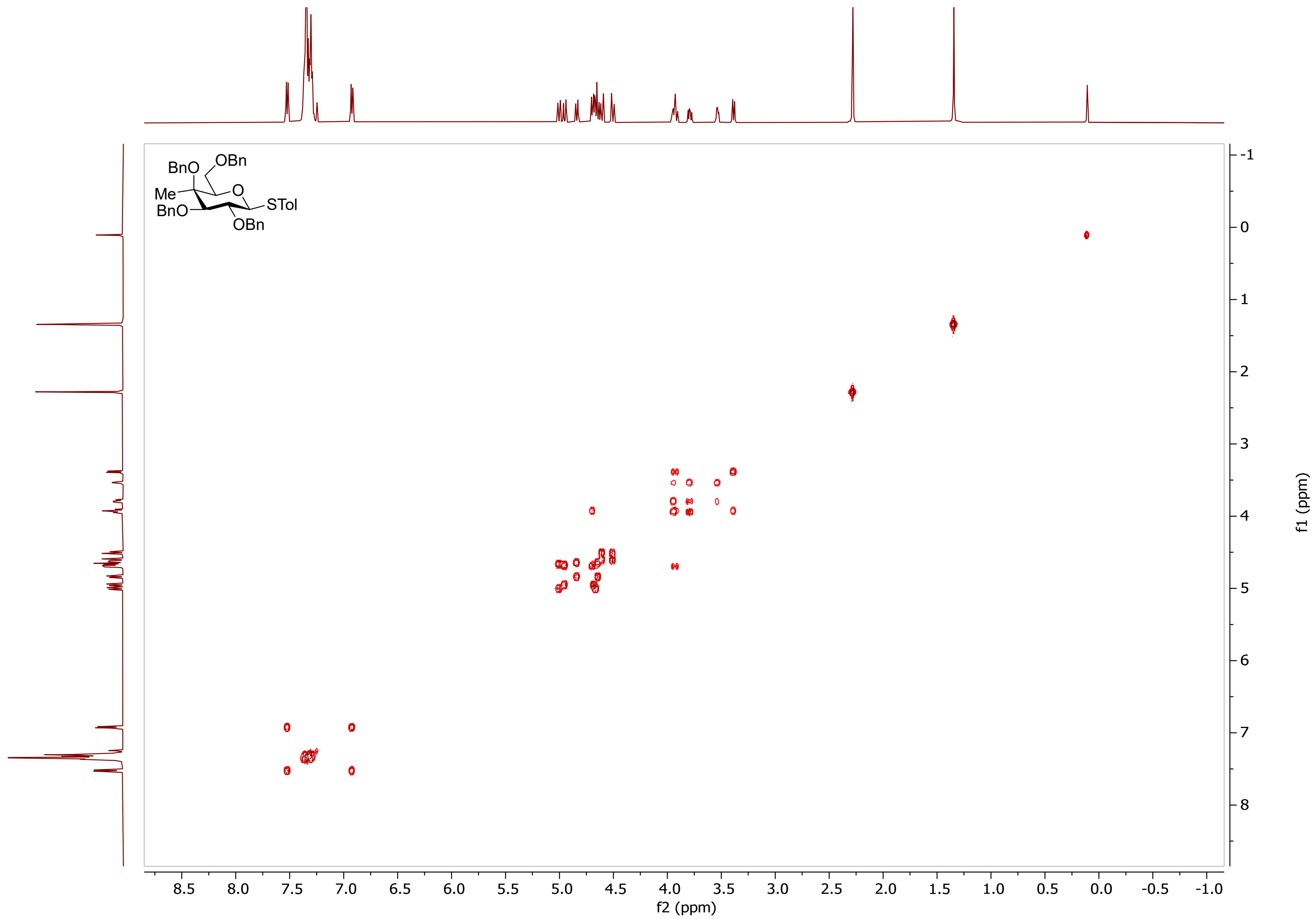

HSQC (500 MHz, CDCl$_3$) spectrum of *p*-methylphenyl 2,3,4,6-tetra-*O*-benzyl-4-*C*-methyl-1-thio-*β*-D-galactopyranoside (29)

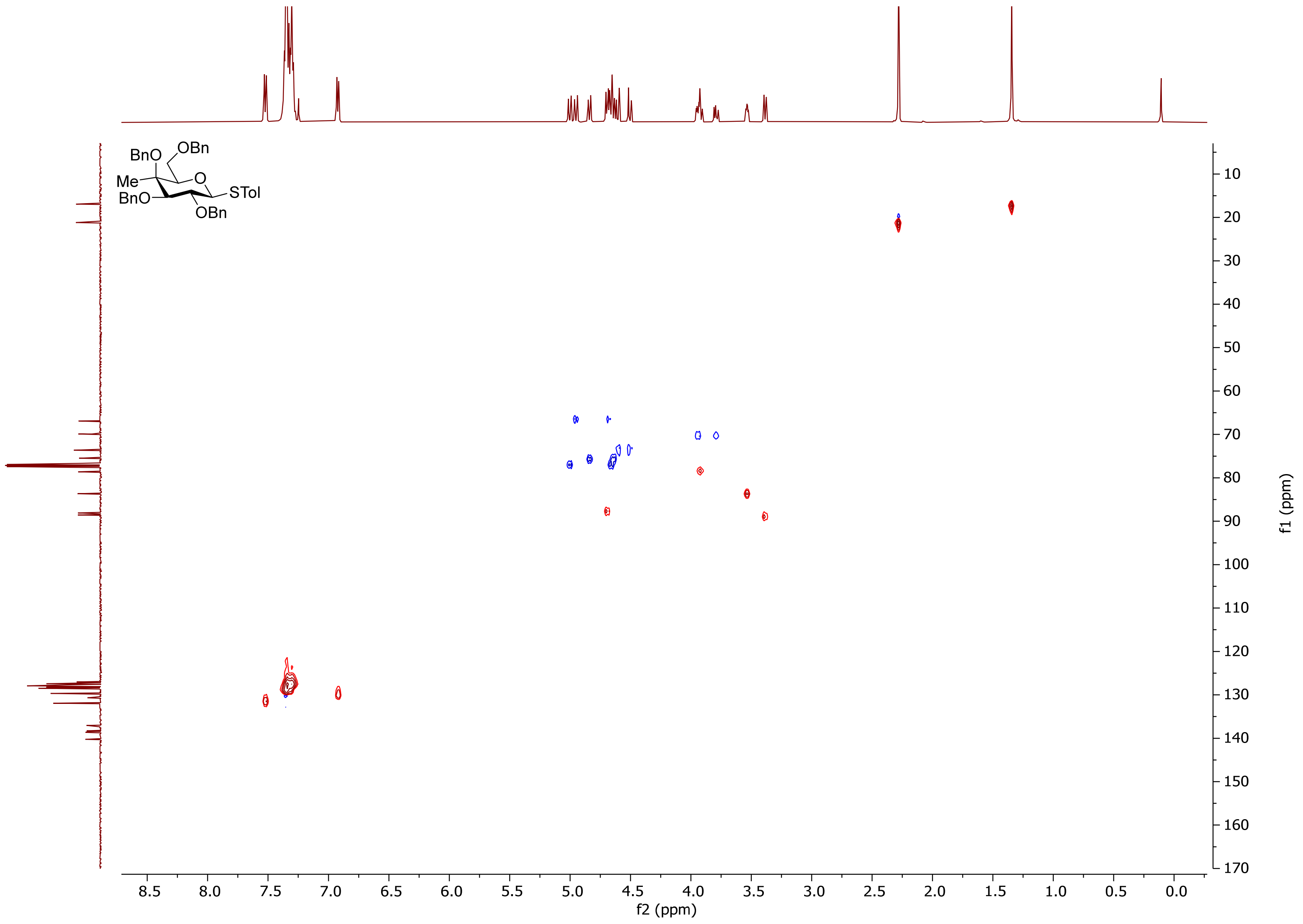

^{1}H NMR (500 MHz, CDCl$_3$) spectrum of *p*-methylphenyl 2,3,4,6-tetra-*O*-benzyl-4-*C*-methyl-1-thio-*β*-D-glucopyranoside (**30**)

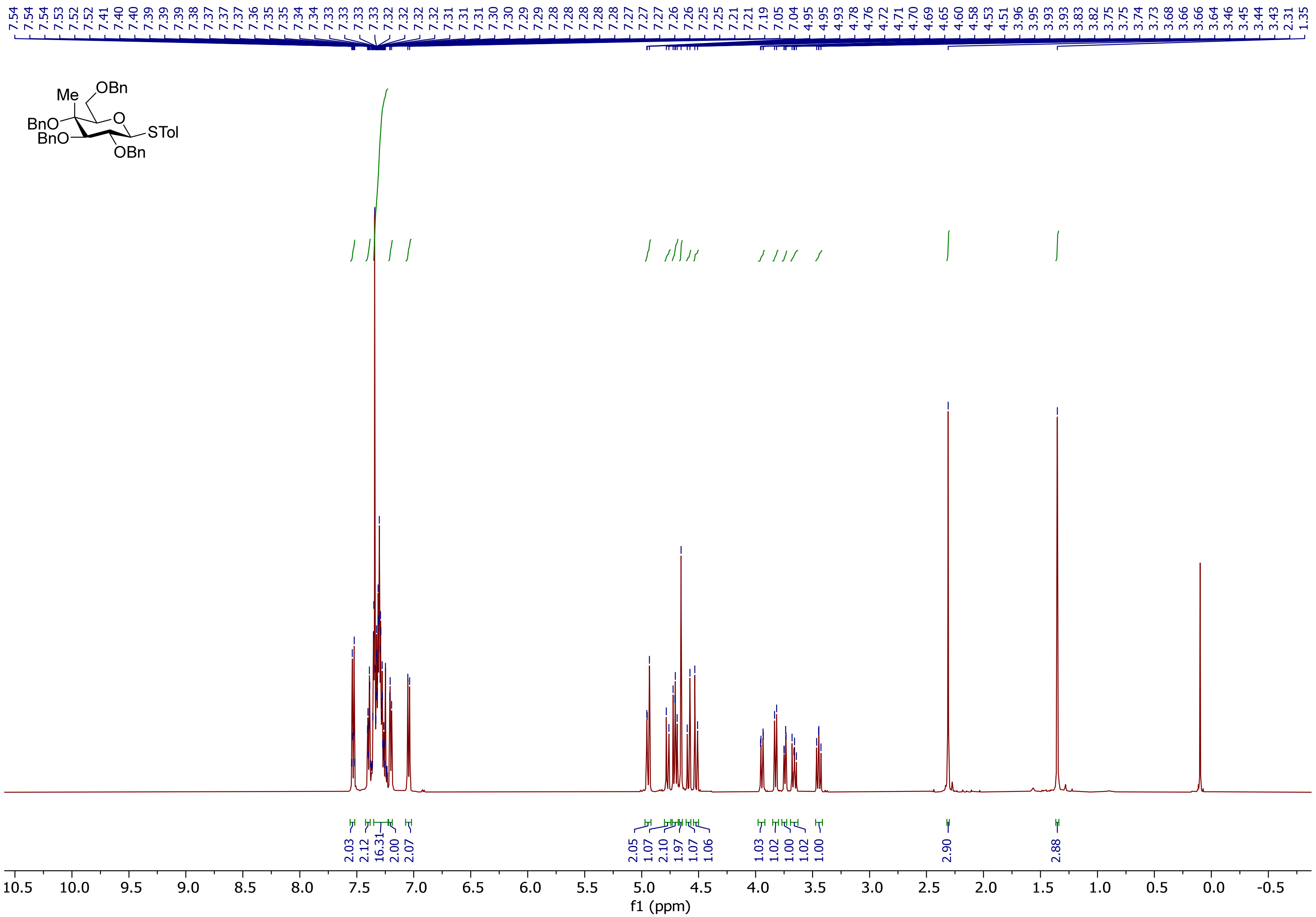

$^{13}C\{^1H\}$ NMR (126 MHz, $CDCl_3$) spectrum of *p*-methylphenyl 2,3,4,6-tetra-*O*-benzyl-4-*C*-methyl-1-thio-*β*-D-glucopyranoside (30)

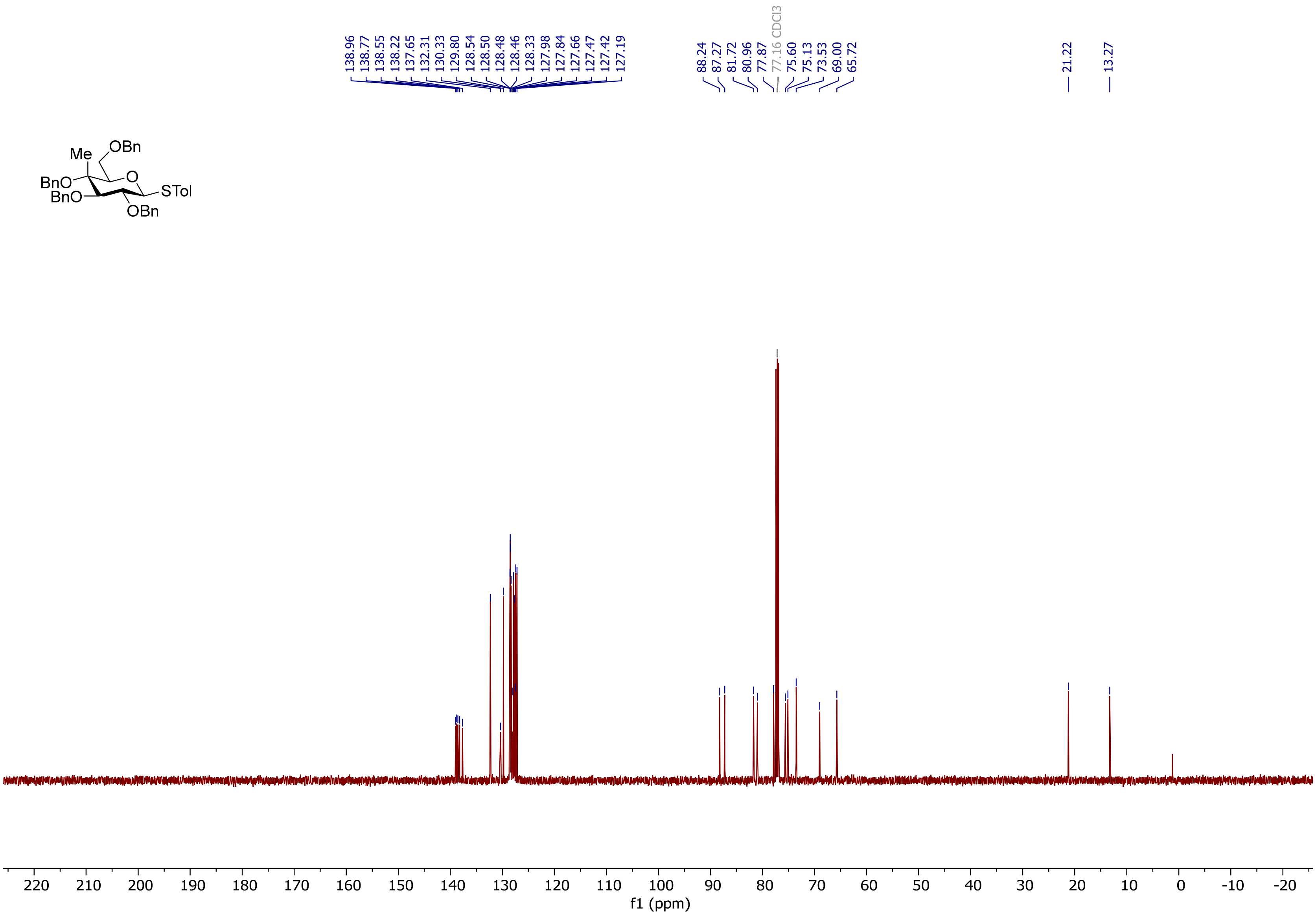

COSY NMR (500 MHz, CDCl$_3$) spectrum of *p*-methylphenyl 2,3,4,6-tetra-*O*-benzyl-4-*C*-methyl-1-thio-*β*-D-glucopyranoside (30)

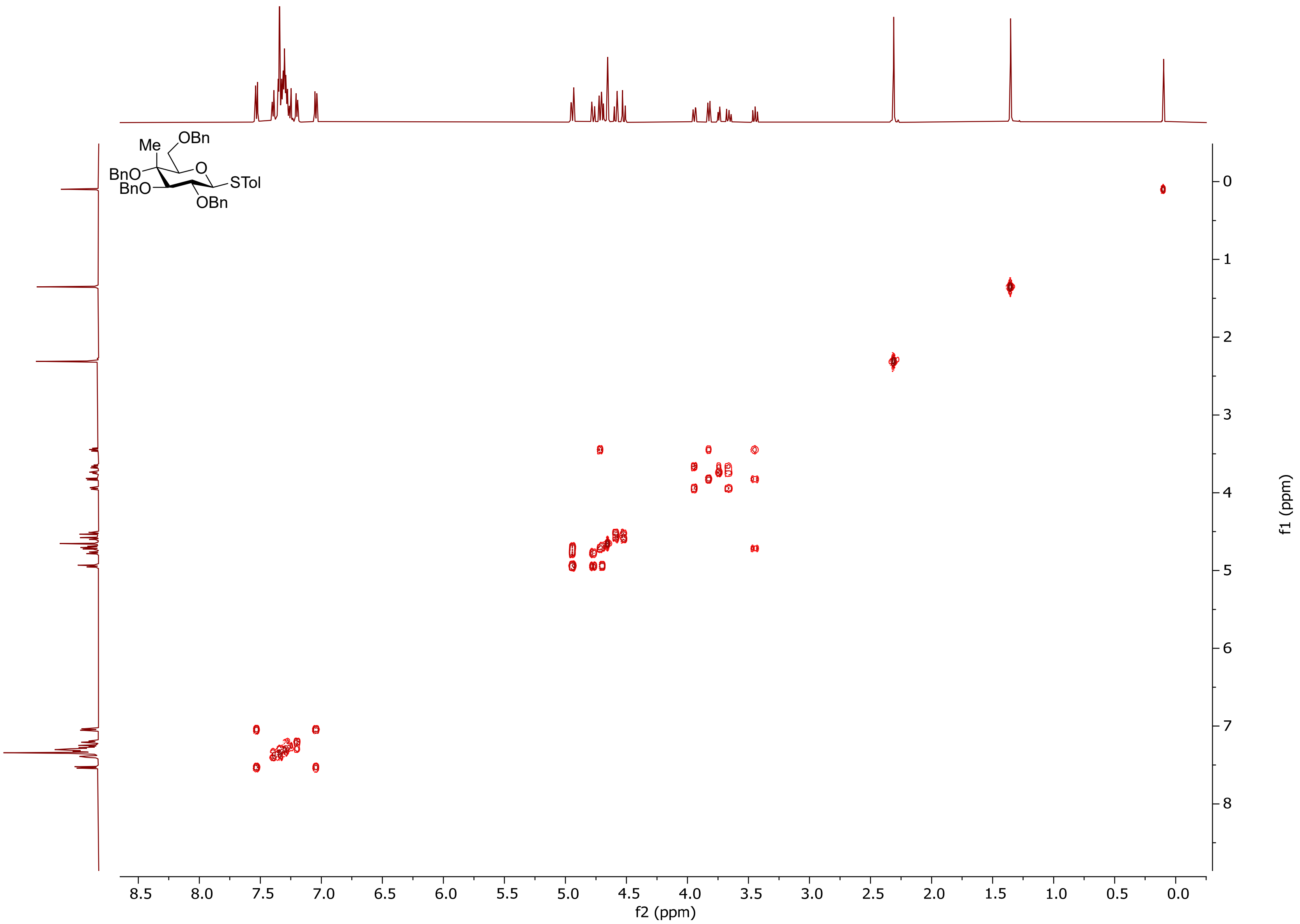

HSQC NMR (500 MHz, $CDCl_3$) spectrum of *p*-methylphenyl 2,3,4,6-tetra-*O*-benzyl-4-*C*-methyl-1-thio-*β*-D-glucopyranoside (30)

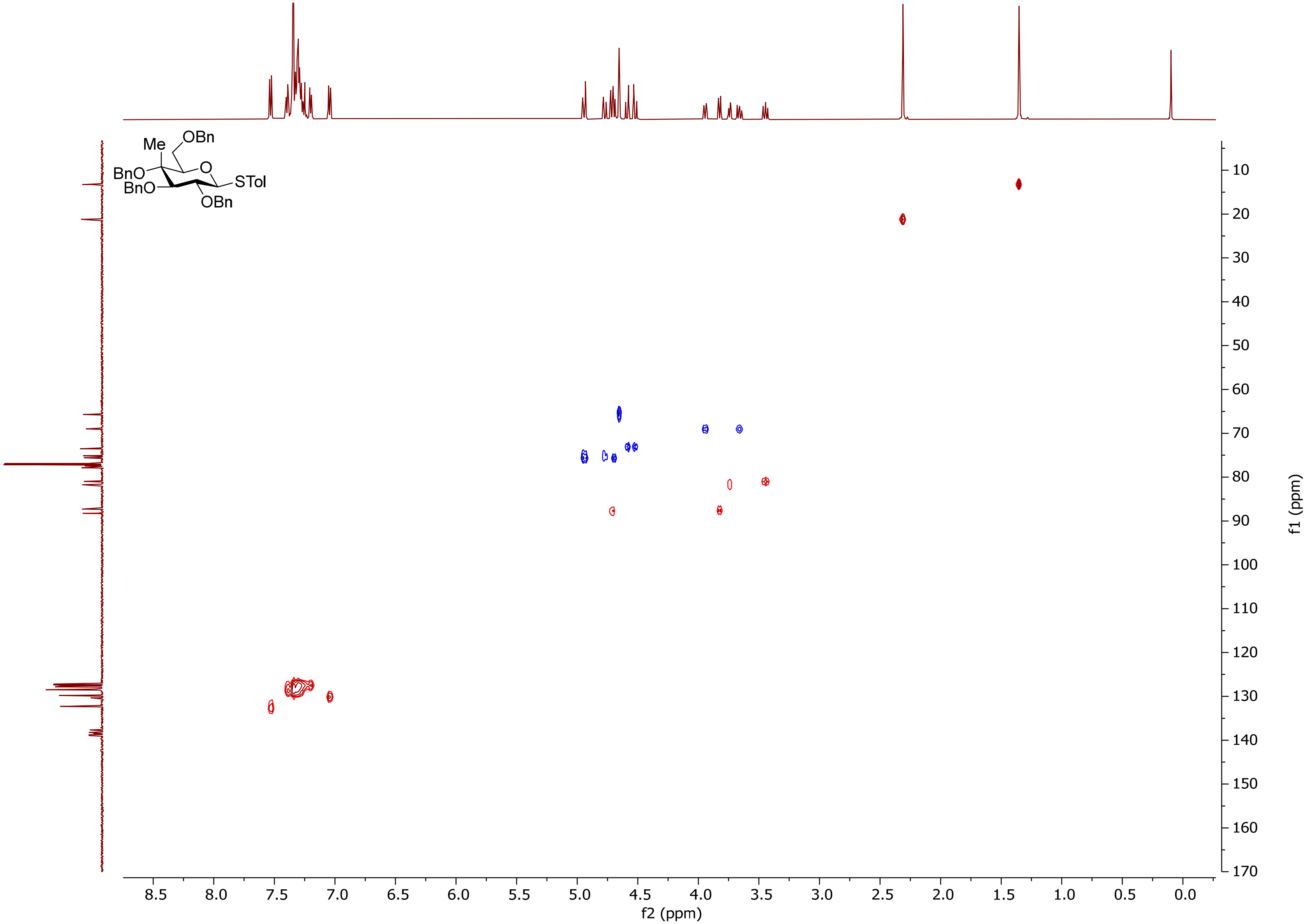

^{1}H NMR (500 MHz, $CDCl_3$) spectrum of 6-*O*-(2,3,4,6-tetra-*O*-benzyl-4-*C*-methyl-*α*-D-galactopyranosyl)-1,2:3,4-di-*O*-isopropylidene-*α*-D-galactopyranose (32α)

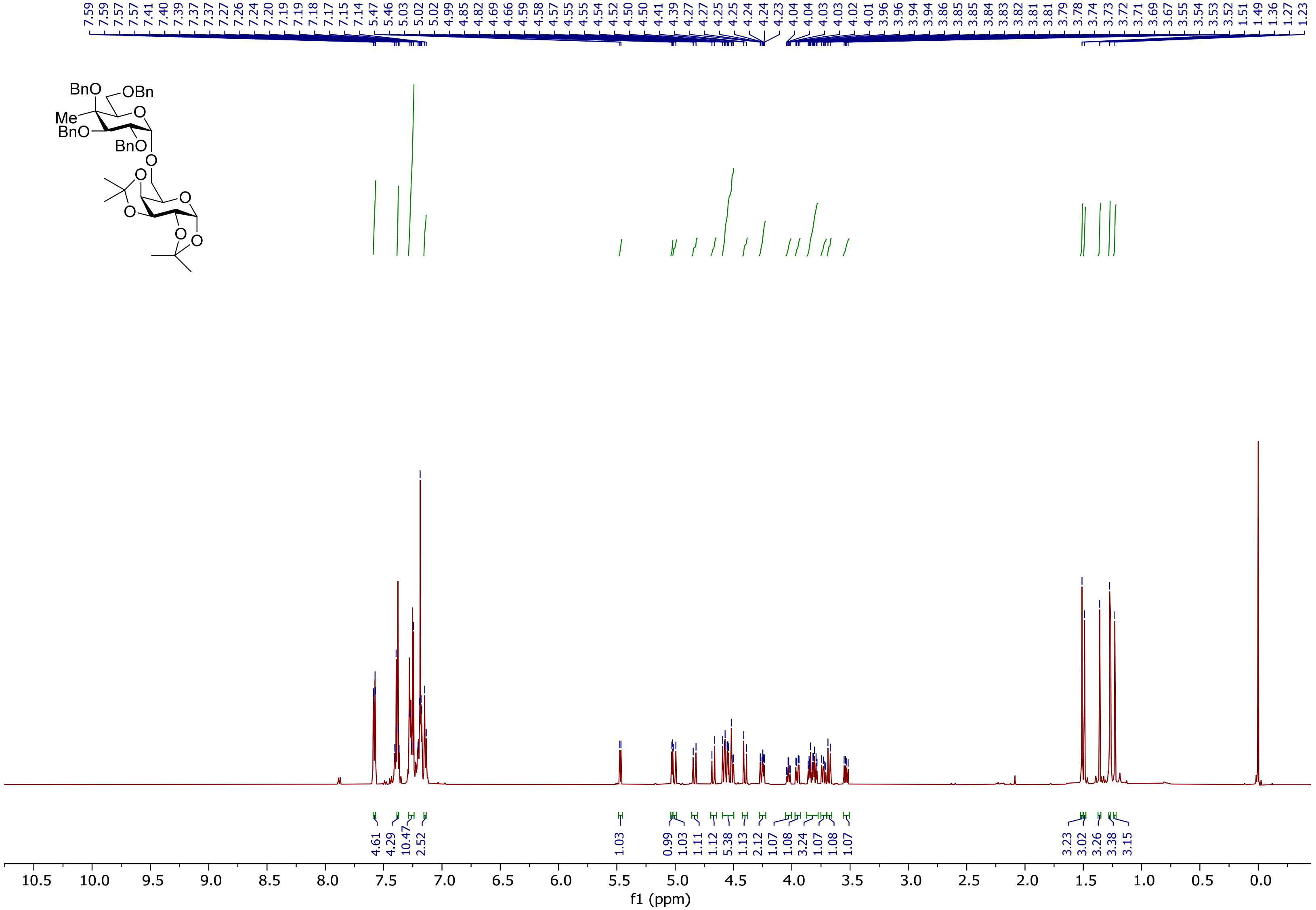

$^{13}C\{^1H\}$ NMR (126 MHz, $CDCl_3$) spectrum of 6-*O*-(2,3,4,6-tetra-*O*-benzyl-4-*C*-methyl-*α*-D-galactopyranosyl)-1,2:3,4-di-*O*-isopropylidene-*α*-D-galactopyranose (32α)

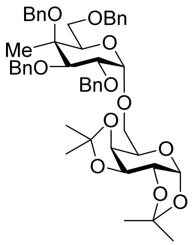

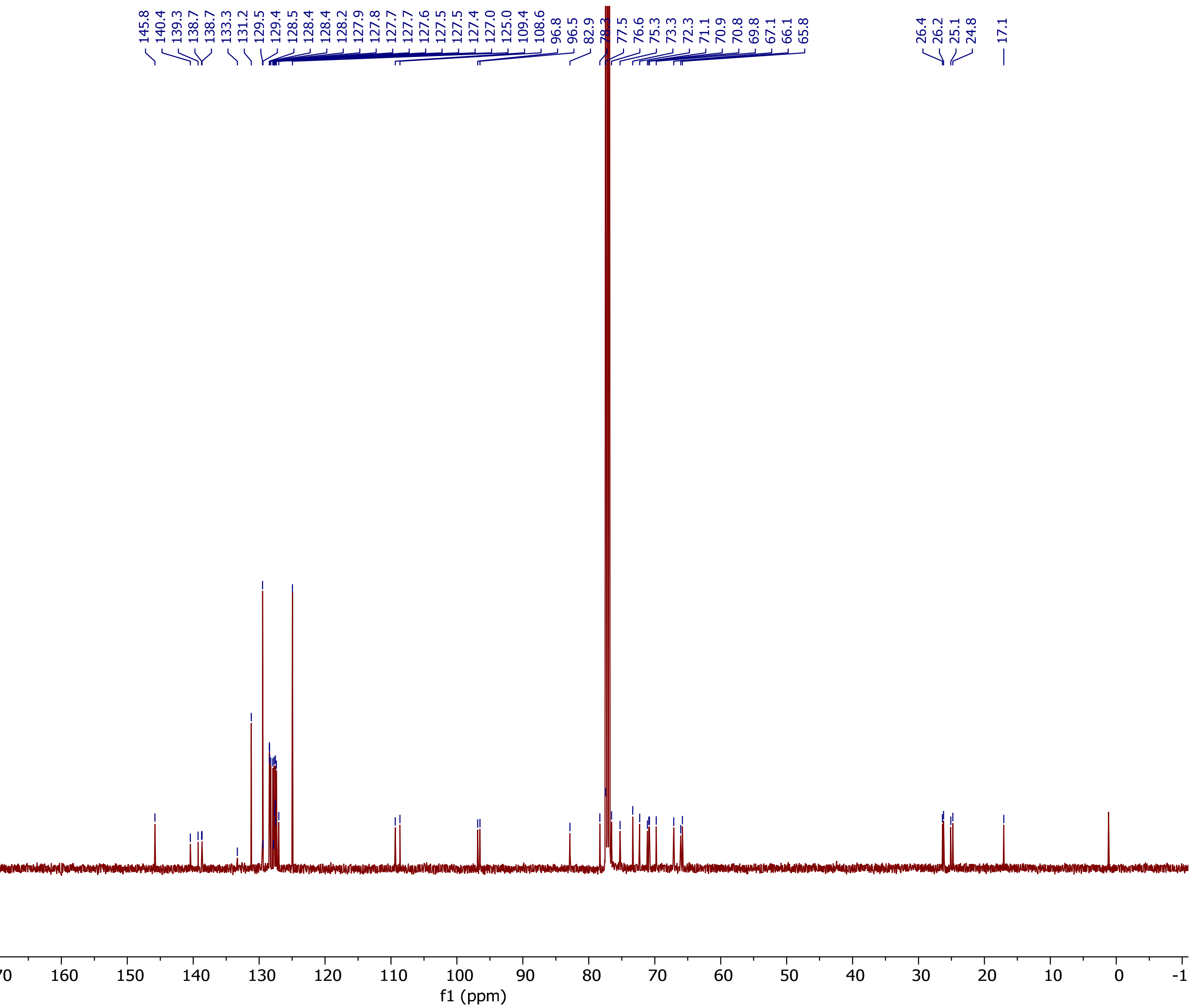

COSY NMR (500 MHz, CDCl$_3$) spectrum of 6-*O*-(2,3,4,6-tetra-*O*-benzyl-4-*C*-methyl-*α*-D-galactopyranosyl)-1,2:3,4-di-*O*-isopropylidene-*α*-D-galactopyranose (32α)

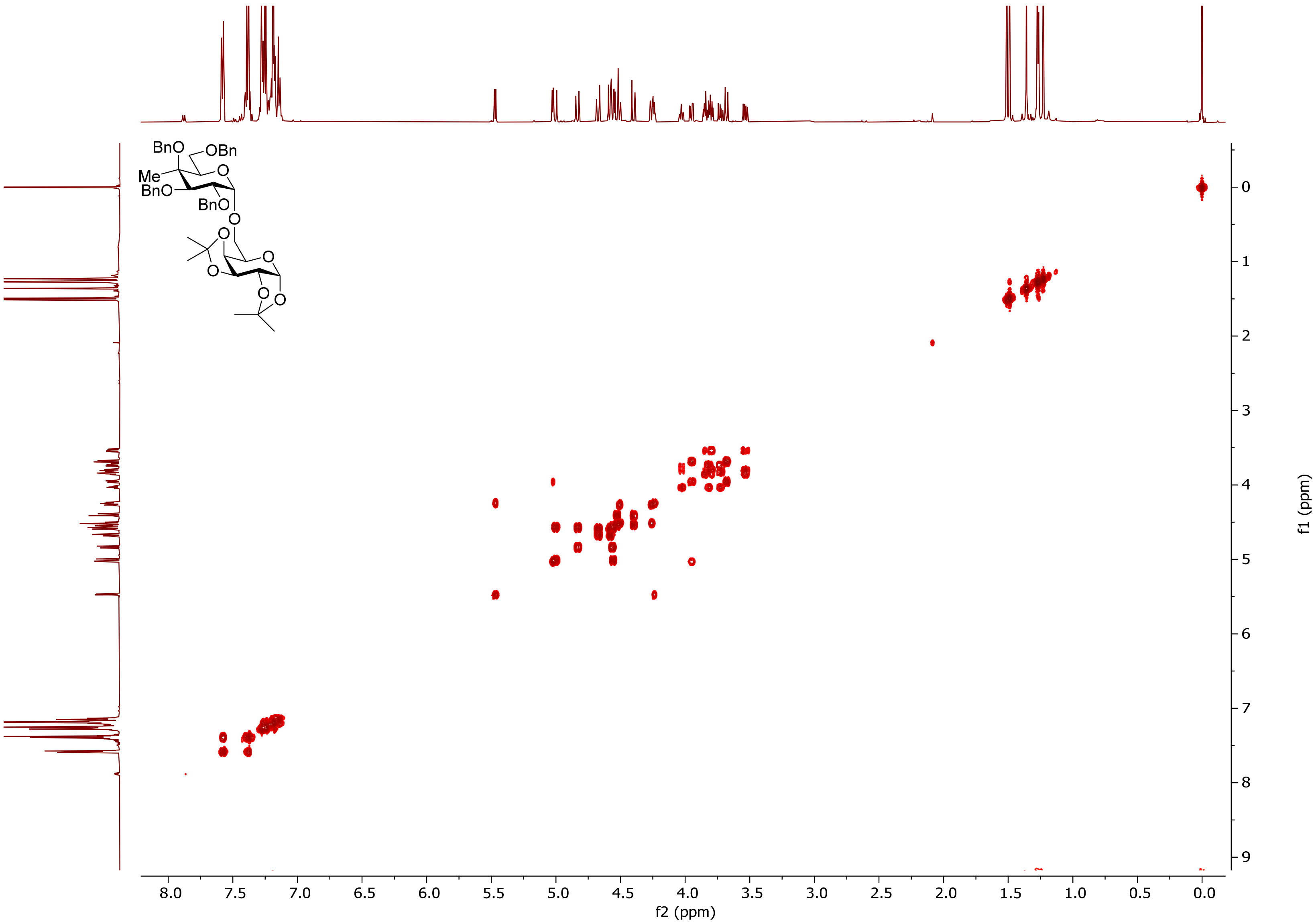

HSQC NMR (500 MHz, CDCl3) spectrum of 6-*O*-(2,3,4,6-tetra-*O*-benzyl-4-*C*-methyl-*α*-D-galactopyranosyl)-1,2:3,4-di-*O*-isopropylidene-*α*-D-galactopyranose (32α)

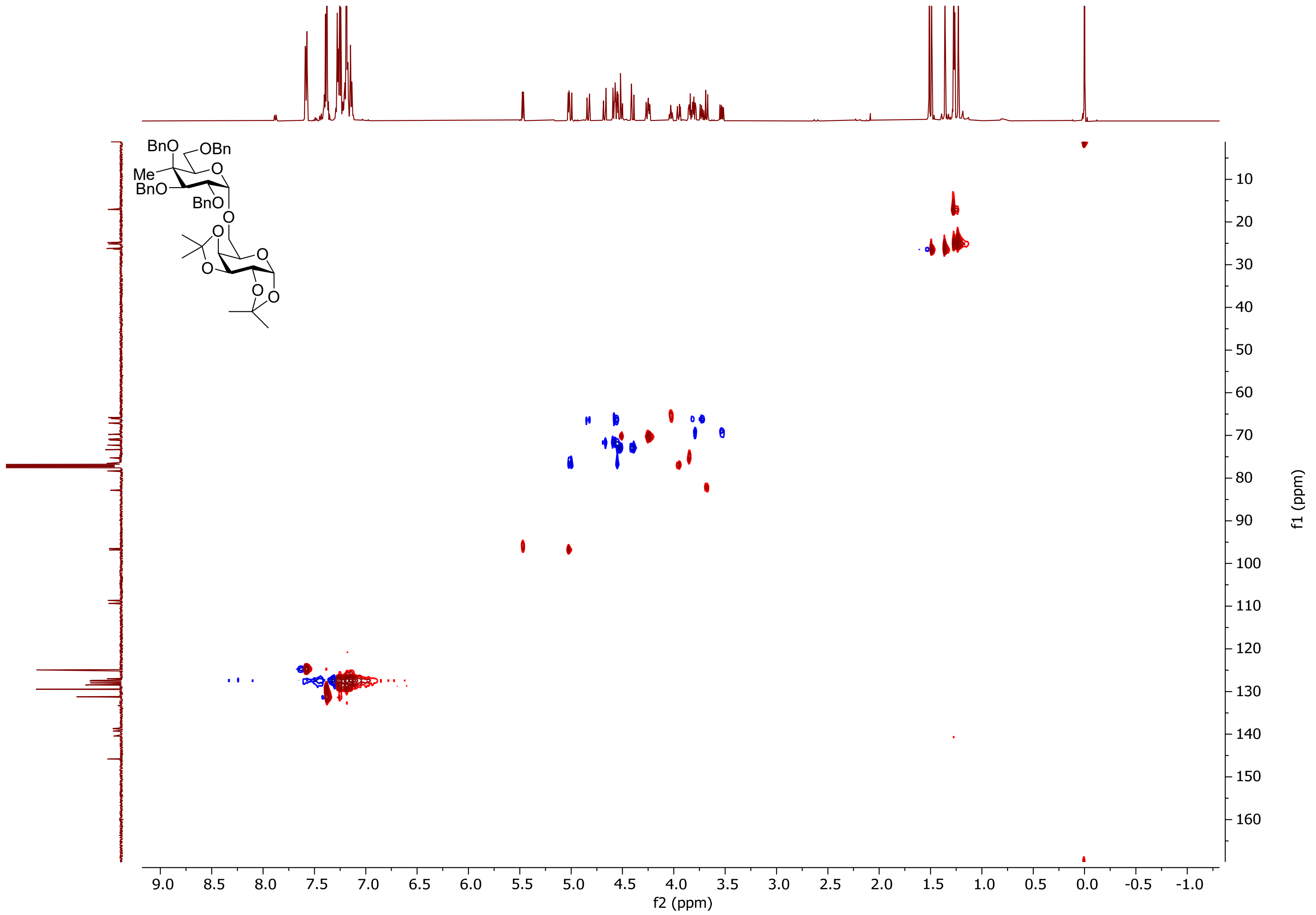

^{1}H NMR (500 MHz, $CDCl_3$) spectrum of 6-*O*-(2,3,4,6-tetra-*O*-benzyl-4-*C*-methyl-*β*-D-galactopyranosyl)-1,2:3,4-di-*O*-isopropylidene-*α*-D-galactopyranose (32β)

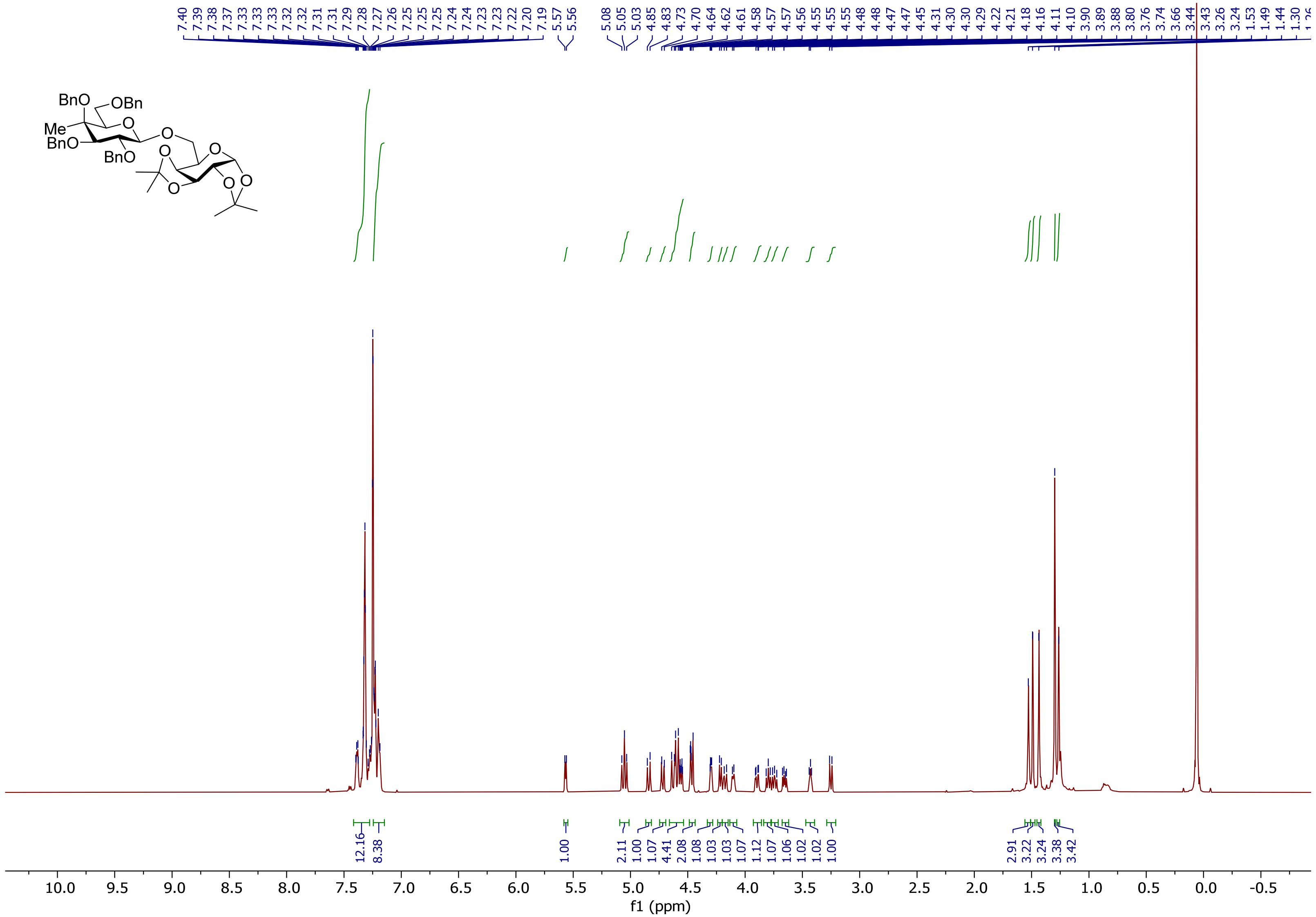

$^{13}C\{^{1}H\}$ NMR (126 MHz, $CDCl_3$) spectrum of 6-*O*-(2,3,4,6-tetra-*O*-benzyl-4-*C*-methyl-*β*-D-galactopyranosyl)-1,2:3,4-di-*O*-isopropylidene-*α*-D-galactopyranose (32β)

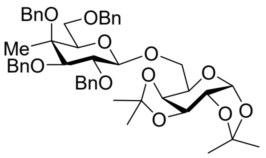

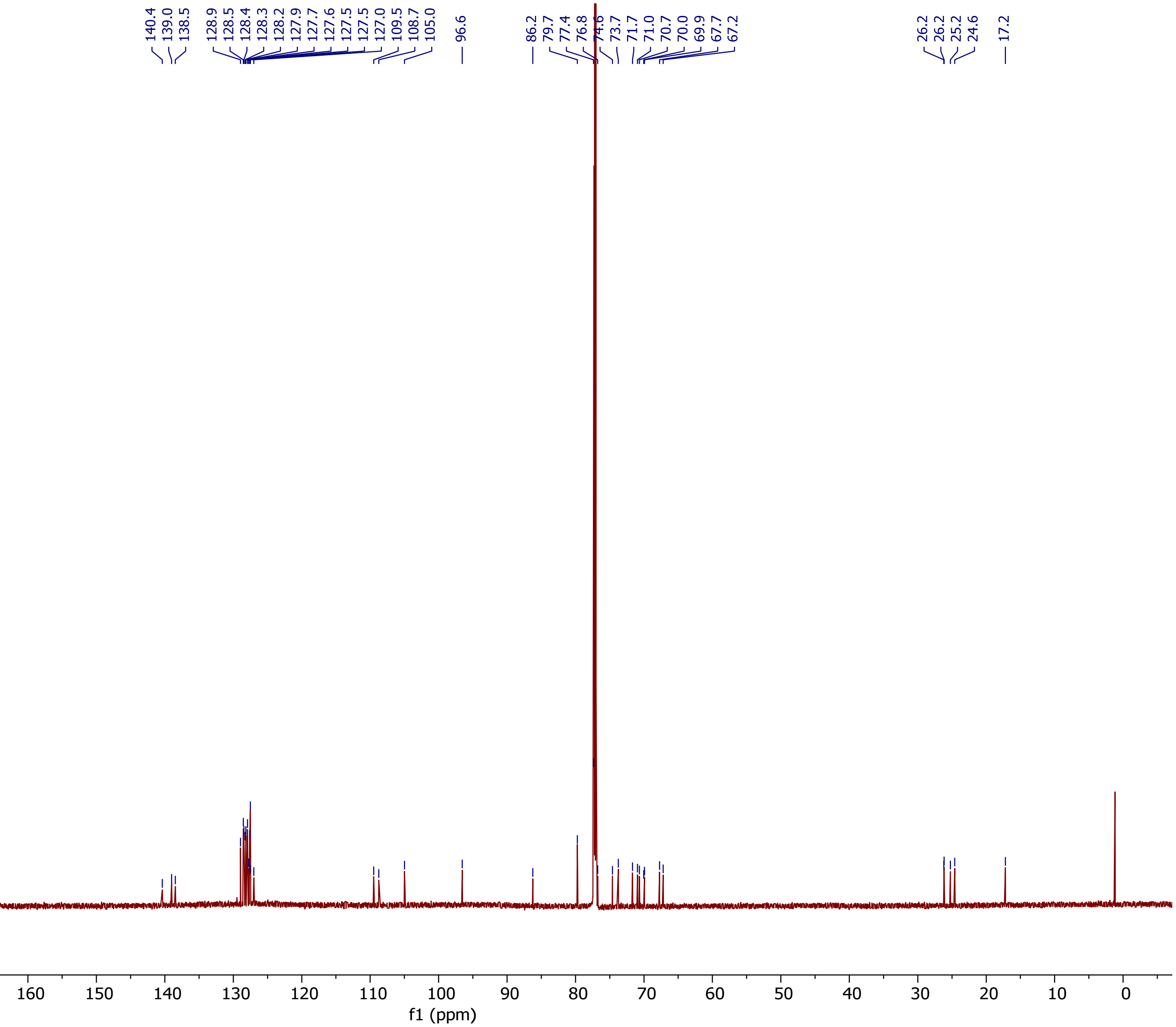

COSY NMR (500 MHz, CDCl₃) spectrum of 6-*O*-(2,3,4,6-tetra-*O*-benzyl-4-*C*-methyl-*β*-D-galactopyranosyl)-1,2:3,4-di-*O*-isopropylidene-*α*-D-galactopyranose (32β)

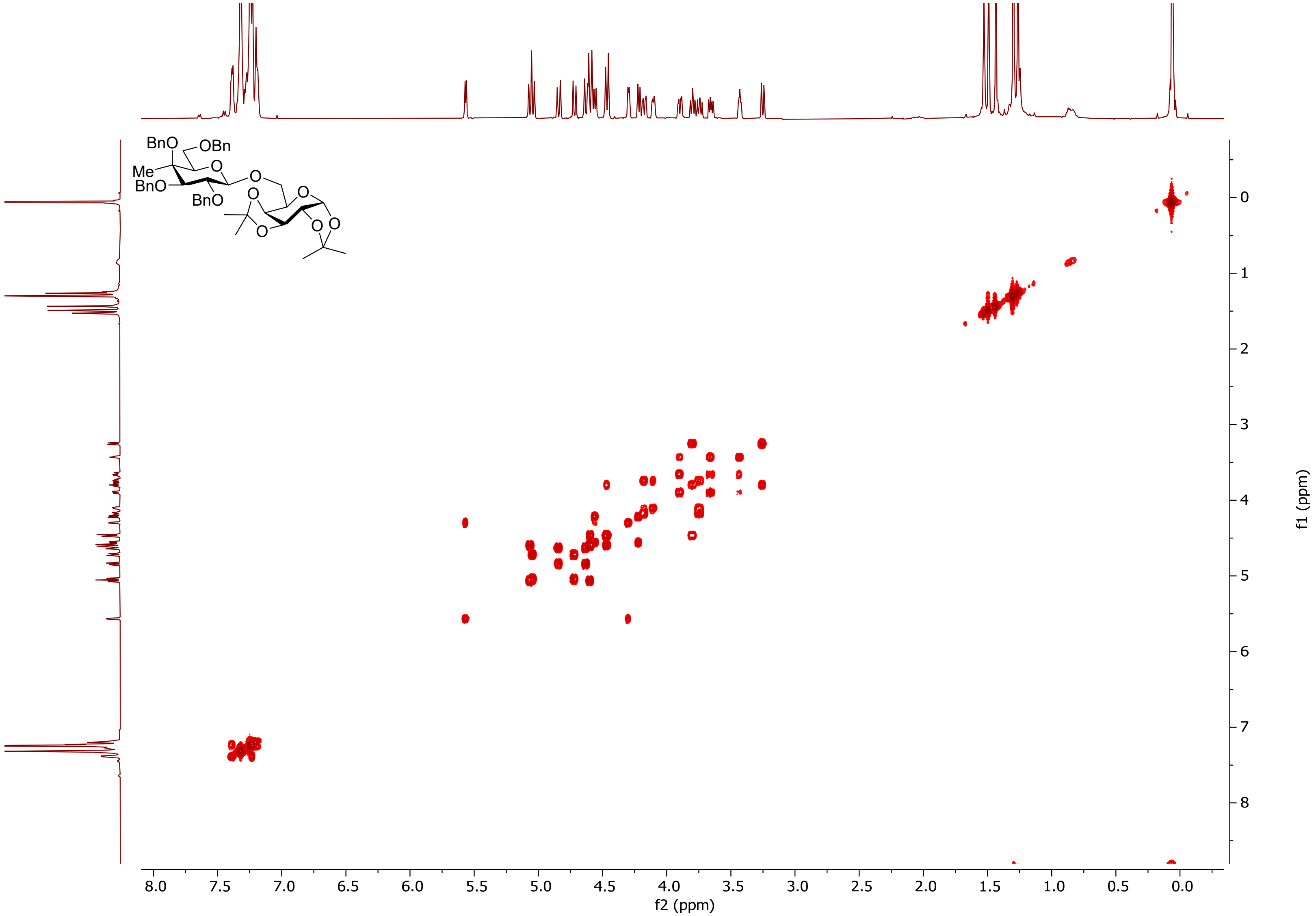

HSQC NMR (500 MHz, CDCl$_3$) spectrum of 6-*O*-(2,3,4,6-tetra-*O*-benzyl-4-*C*-methyl-*β*-D-galactopyranosyl)-1,2:3,4-di-*O*-isopropylidene-*α*-D-galactopyranose (32β)

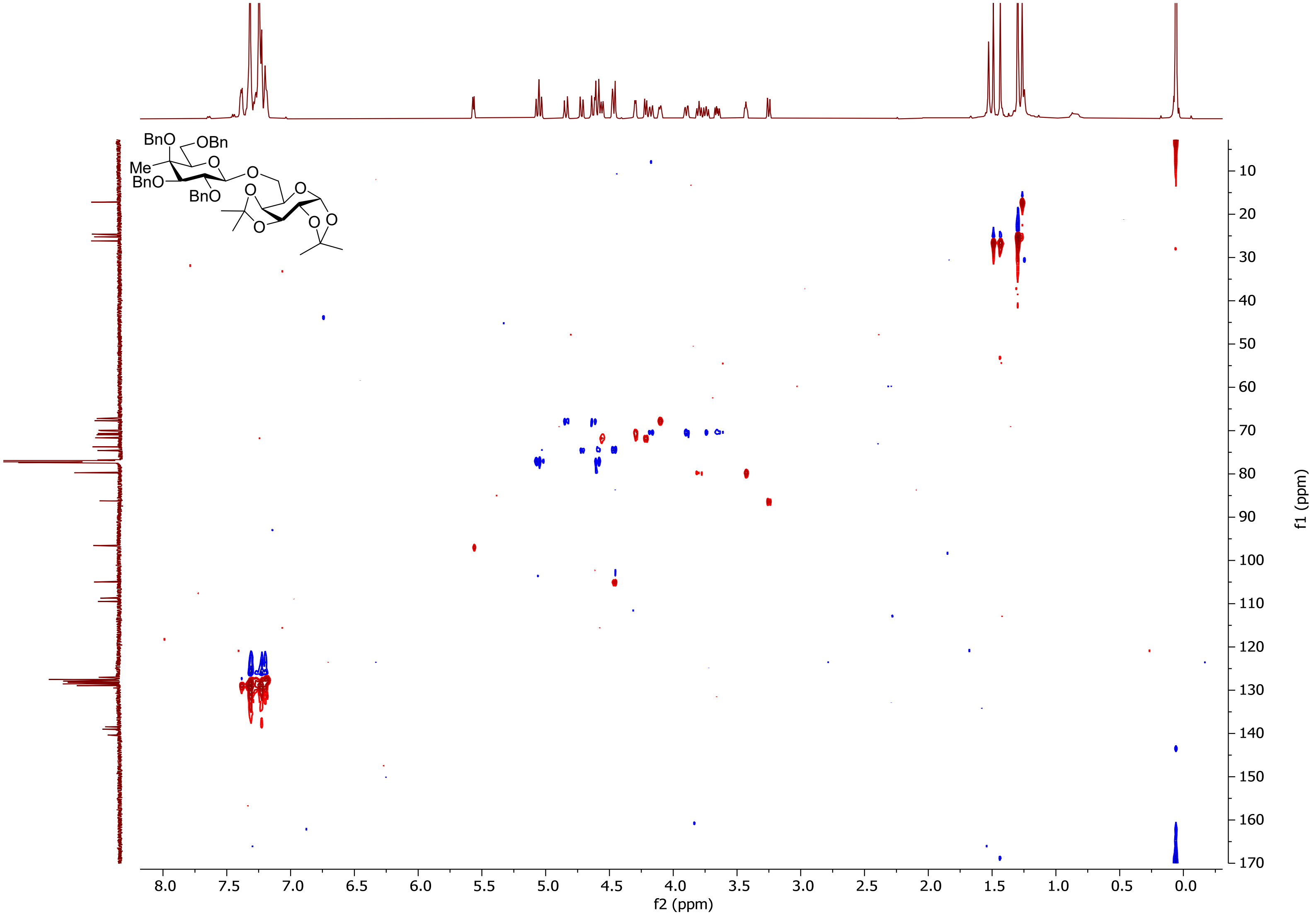

^{1}H NMR (500 MHz, CDCl$_3$) spectrum of 1,4-anhydro-2,3,6-tri-*O*-benzyl-4-*C*-methyl-*β*-D-galactopyranose (33)

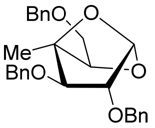

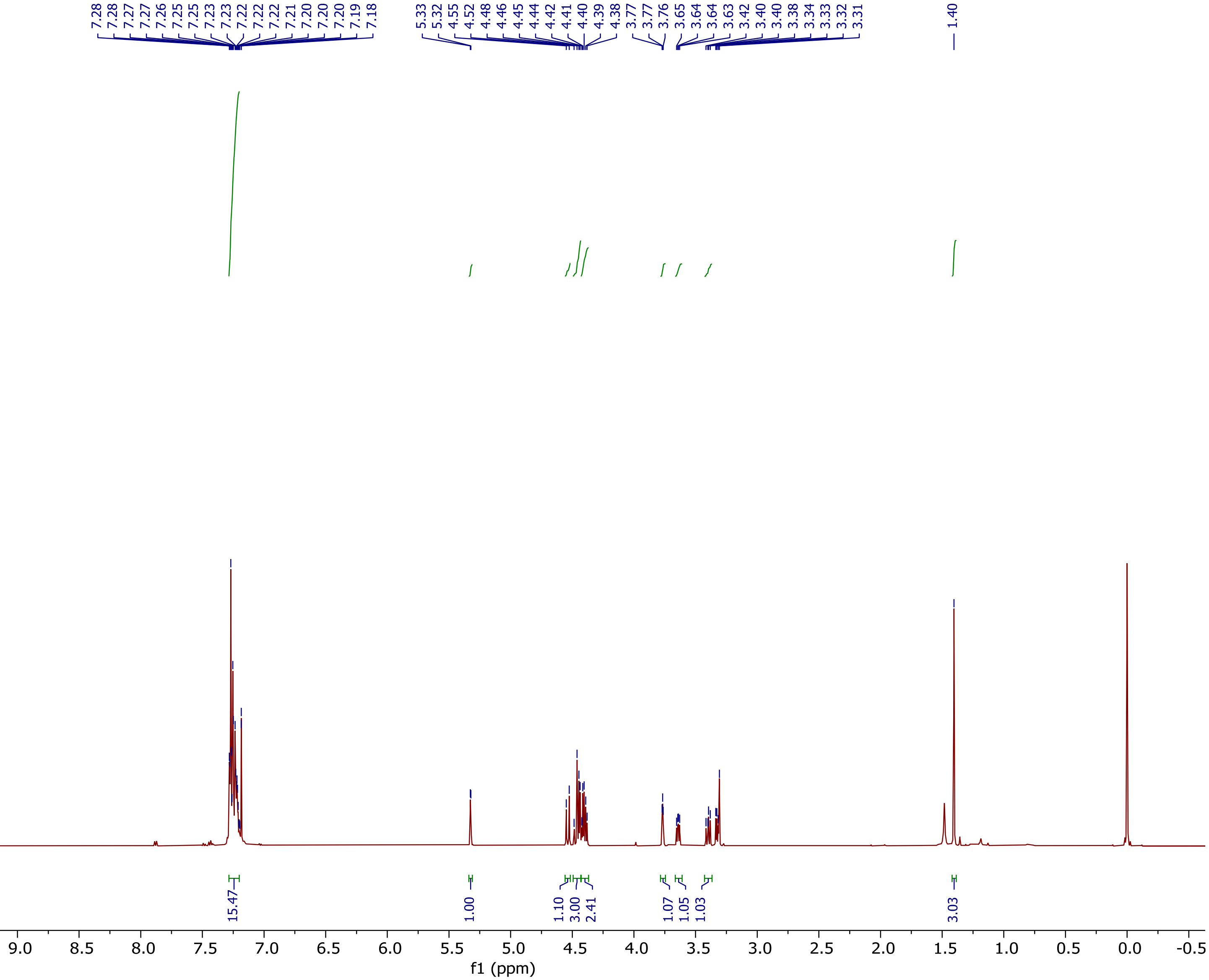

$^{13}C\{^1H\}$ NMR (126 MHz, $CDCl_3$) spectrum of 1,4-anhydro-2,3,6-tri-*O*-benzyl-4-*C*-methyl-*β*-D-galactopyranose (33)

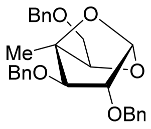

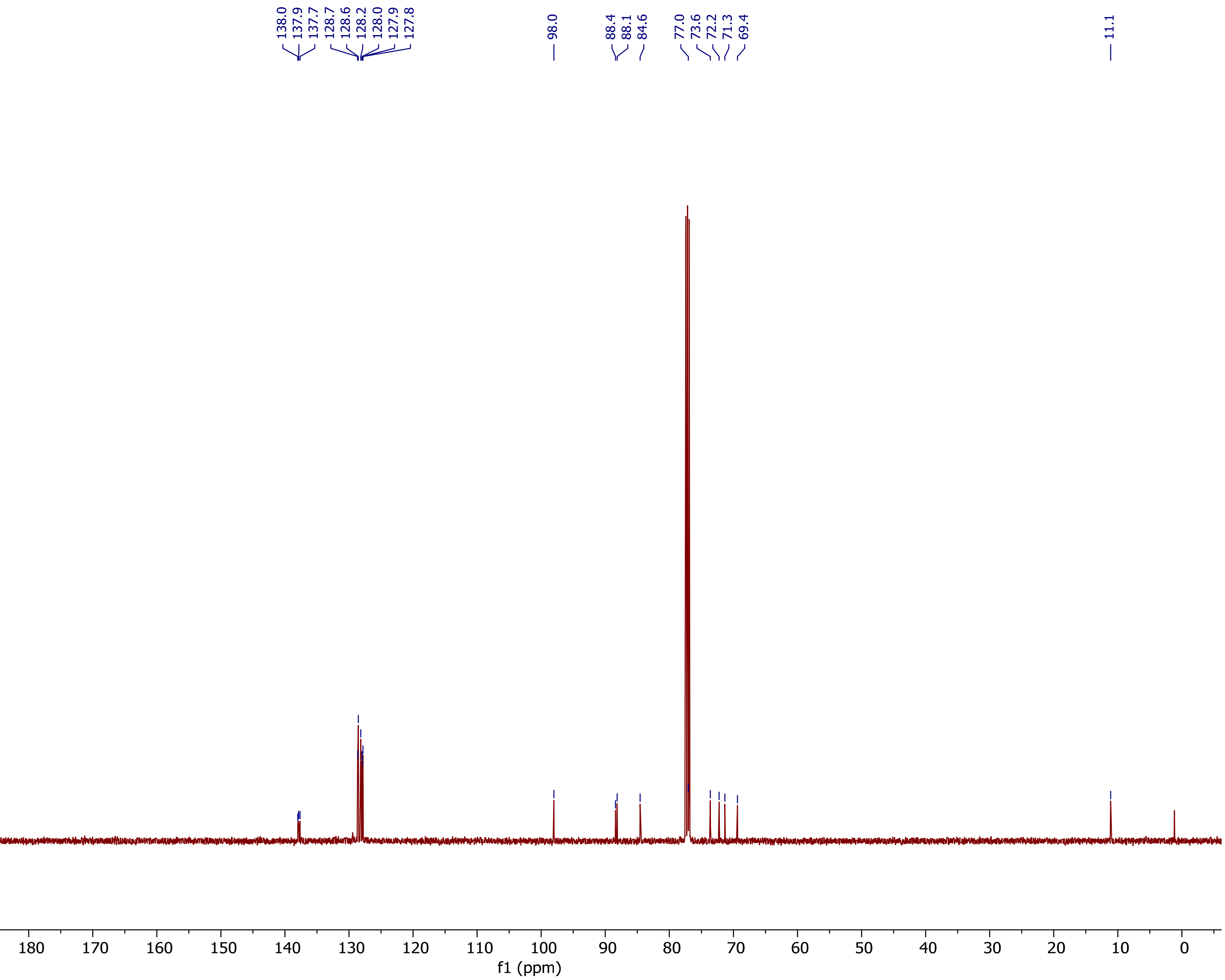

COSY NMR (500 MHz, $CDCl_3$) spectrum of 1,4-anhydro-2,3,6-tri-*O*-benzyl-4-*C*-methyl-*β*-D-galactopyranose (33)

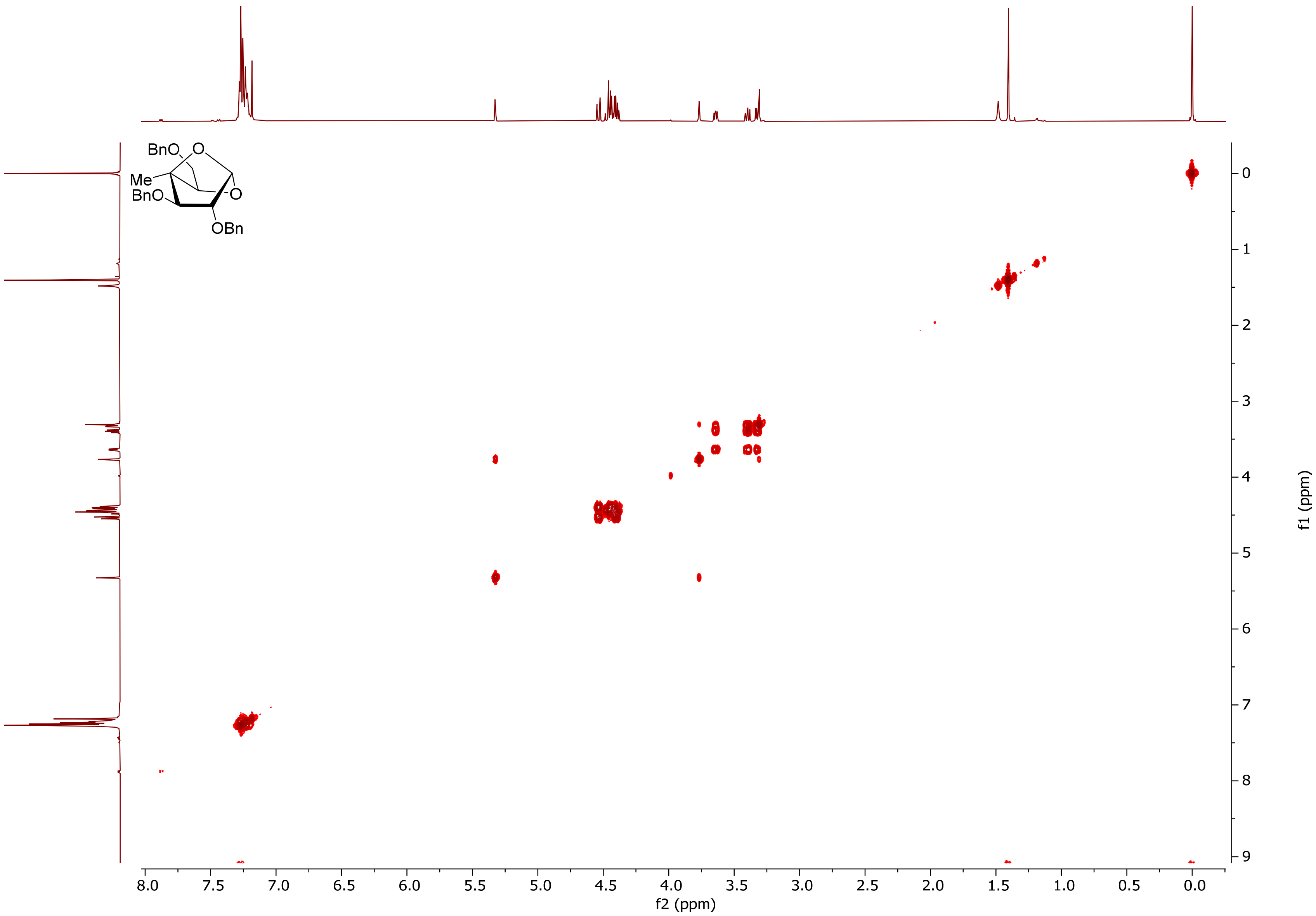

HSQC NMR (500 MHz, $CDCl_3$) spectrum of 1,4-anhydro-2,3,6-tri-*O*-benzyl-4-*C*-methyl-*β*-D-galactopyranose (33)

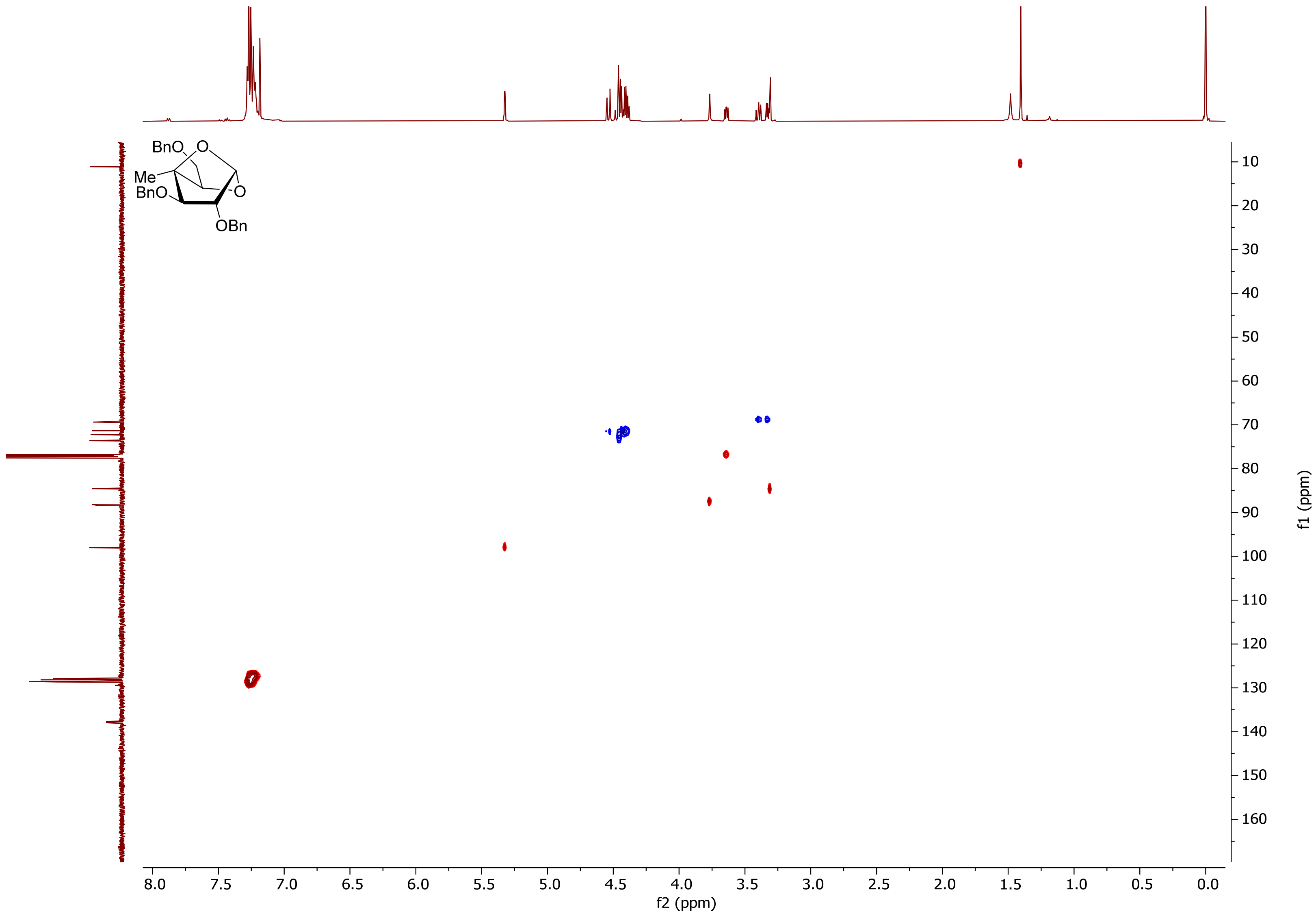

HMBC NMR (500 MHz, CDCl$_3$) spectrum of 1,4-anhydro-2,3,6-tri-*O*-benzyl-4-*C*-methyl-*β*-D-galactopyranose (33)

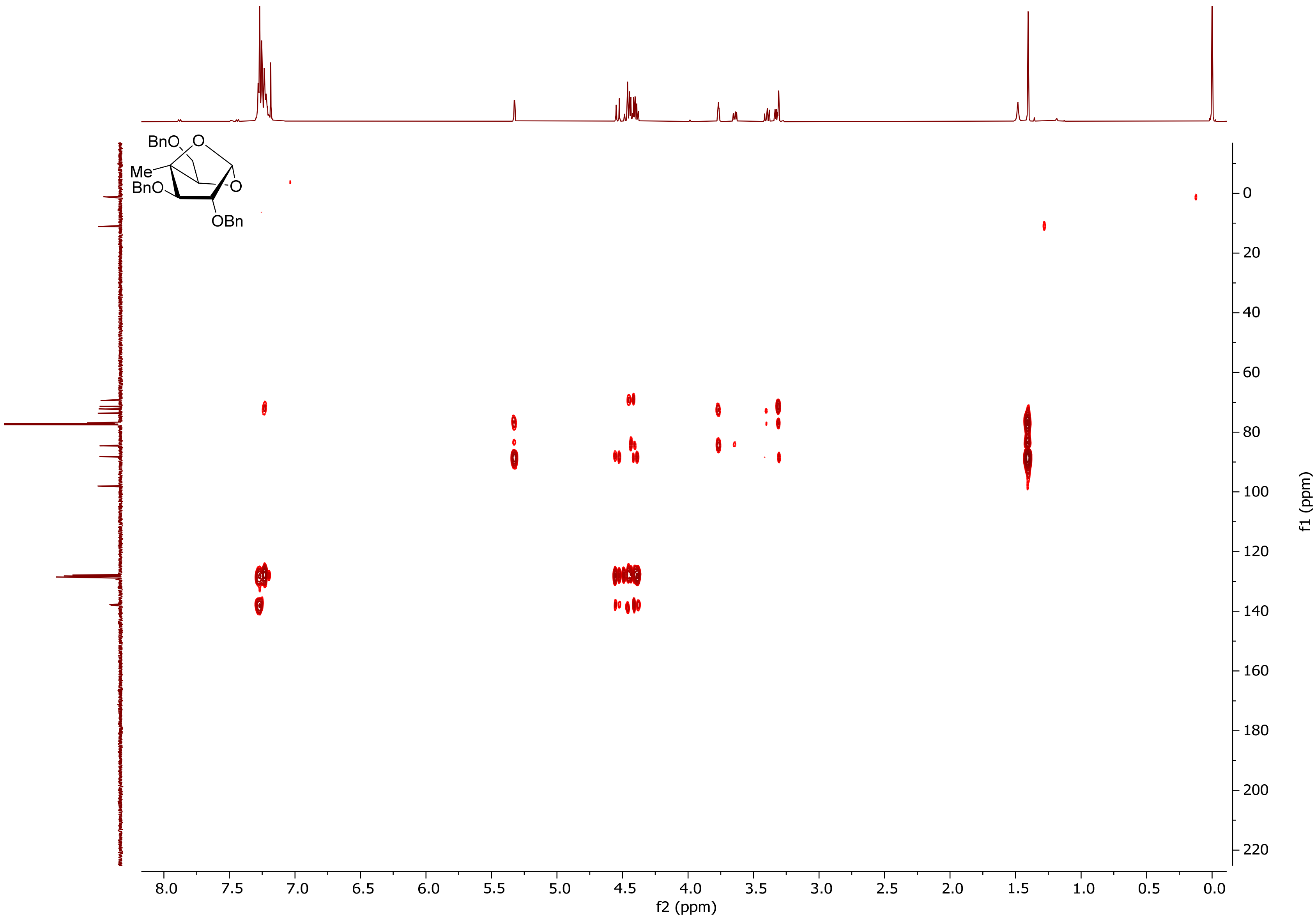

Band-selective HMBC (83-90 ppm) (800 MHz, CDCl3) spectrum of 1,4-anhydro-2,3,6-tri-*O*-benzyl-4-*C*-methyl-*β*-D-galactopyranose (33)

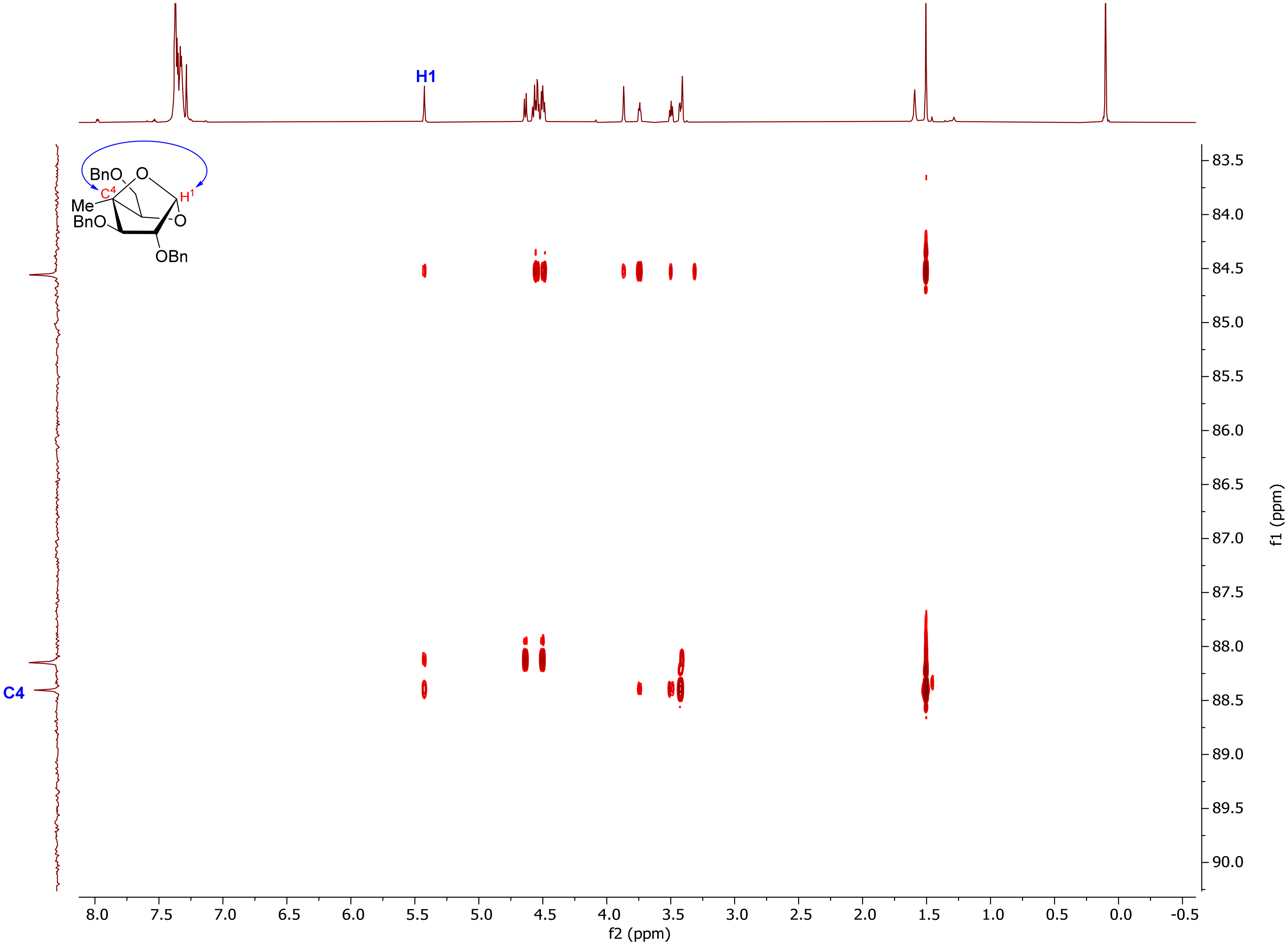

^{1}H NMR (600 MHz, CD_3CN) spectrum of 6-*O*-(2,3,4,6-tetra-*O*-benzyl-4-*C*-methyl-α-D-glucopyranosyl)-1,2:3,4-di-*O*-isopropylidene-*α*-D-galactopyranose (34α)

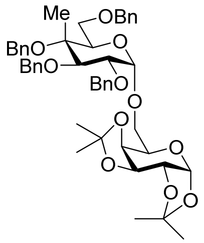

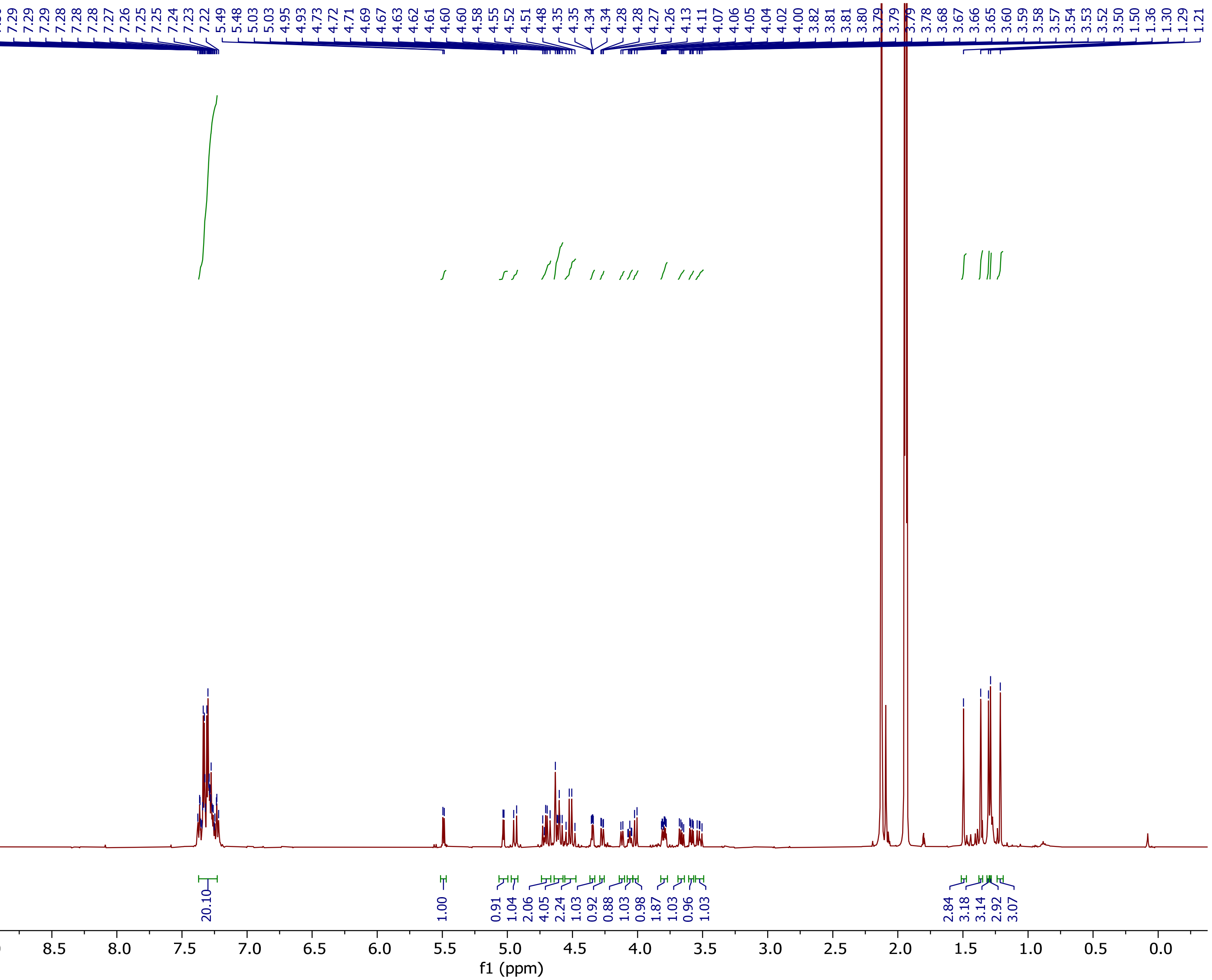

$^{13}C\{^{1}H\}$ NMR (151 MHz, CD_3CN) spectrum of 6-*O*-(2,3,4,6-tetra-*O*-benzyl-4-*C*-methyl-α-D-glucopyranosyl)-1,2:3,4-di-*O*-isopropylidene-α-D-galactopyranose (34α)

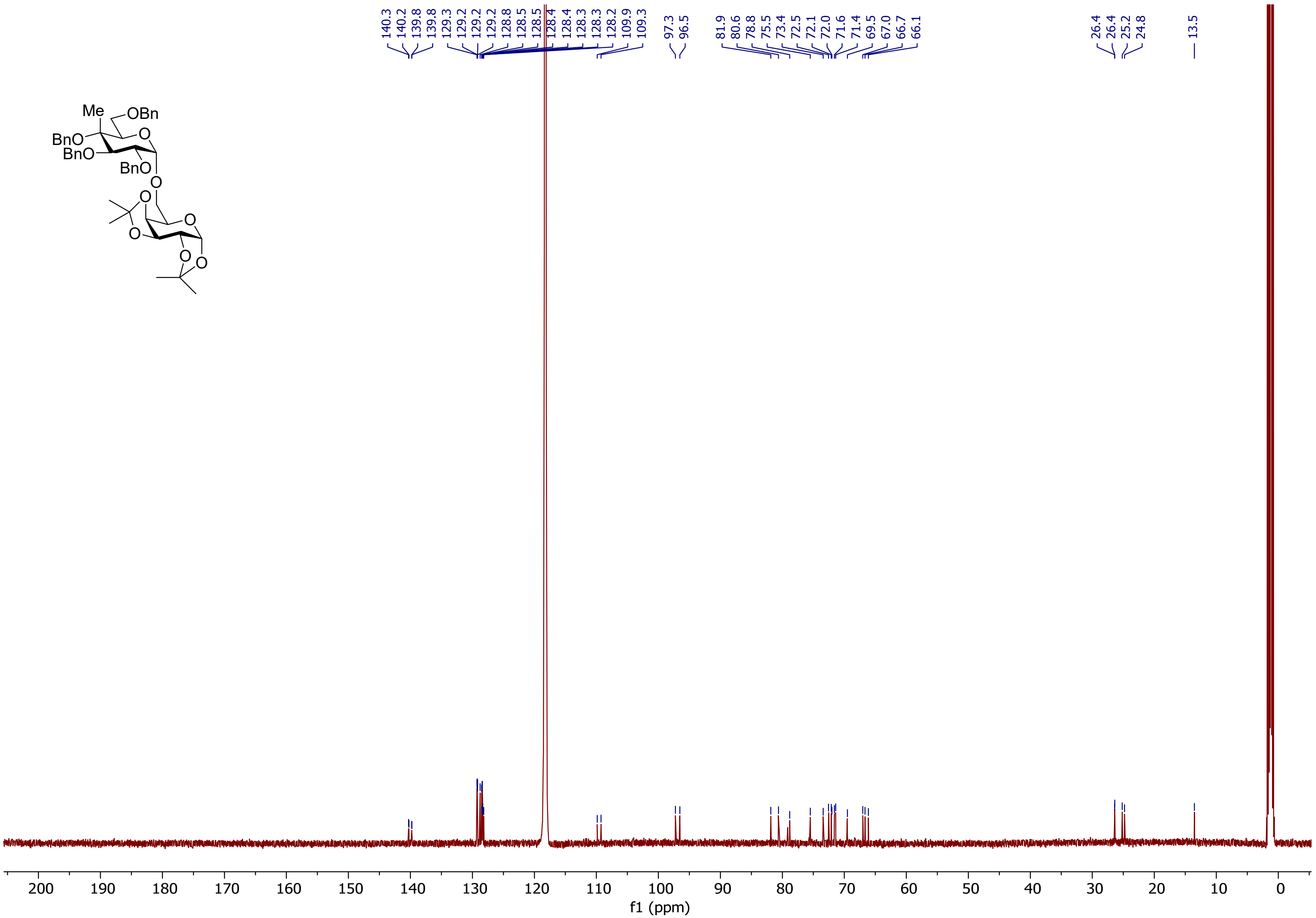

COSY NMR (600 MHz, CD_3CN) spectrum of 6-*O*-(2,3,4,6-tetra-*O*-benzyl-4-*C*-methyl-*α*-D-glucopyranosyl)-1,2:3,4-di-*O*-isopropylidene-*α*-D-galactopyranose (34α)

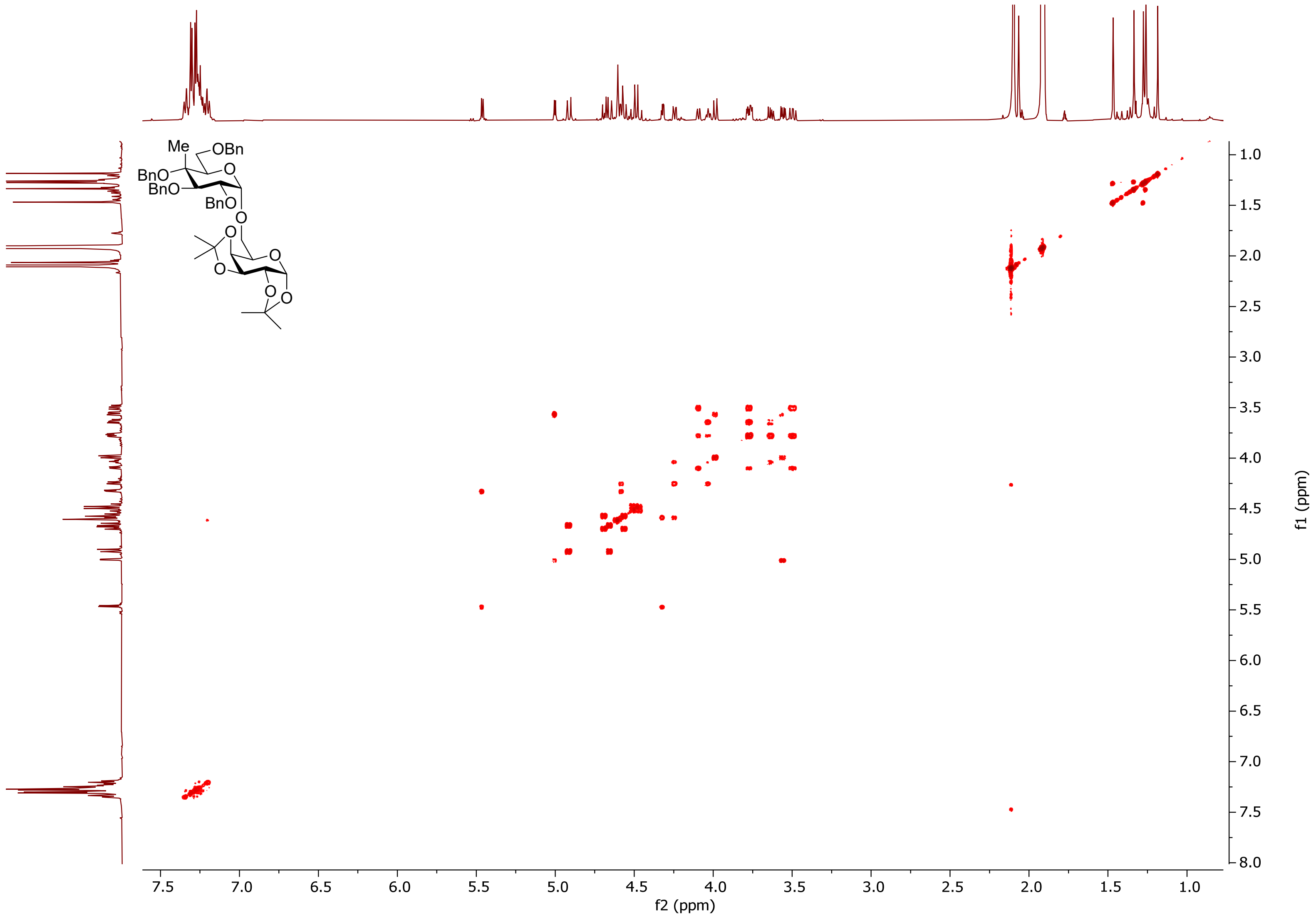

HSQC NMR (600 MHz, CD_3CN) spectrum of 6-*O*-(2,3,4,6-tetra-*O*-benzyl-4-*C*-methyl-*α*-D-glucopyranosyl)-1,2:3,4-di-*O*-isopropylidene-*α*-D-galactopyranose (34α)

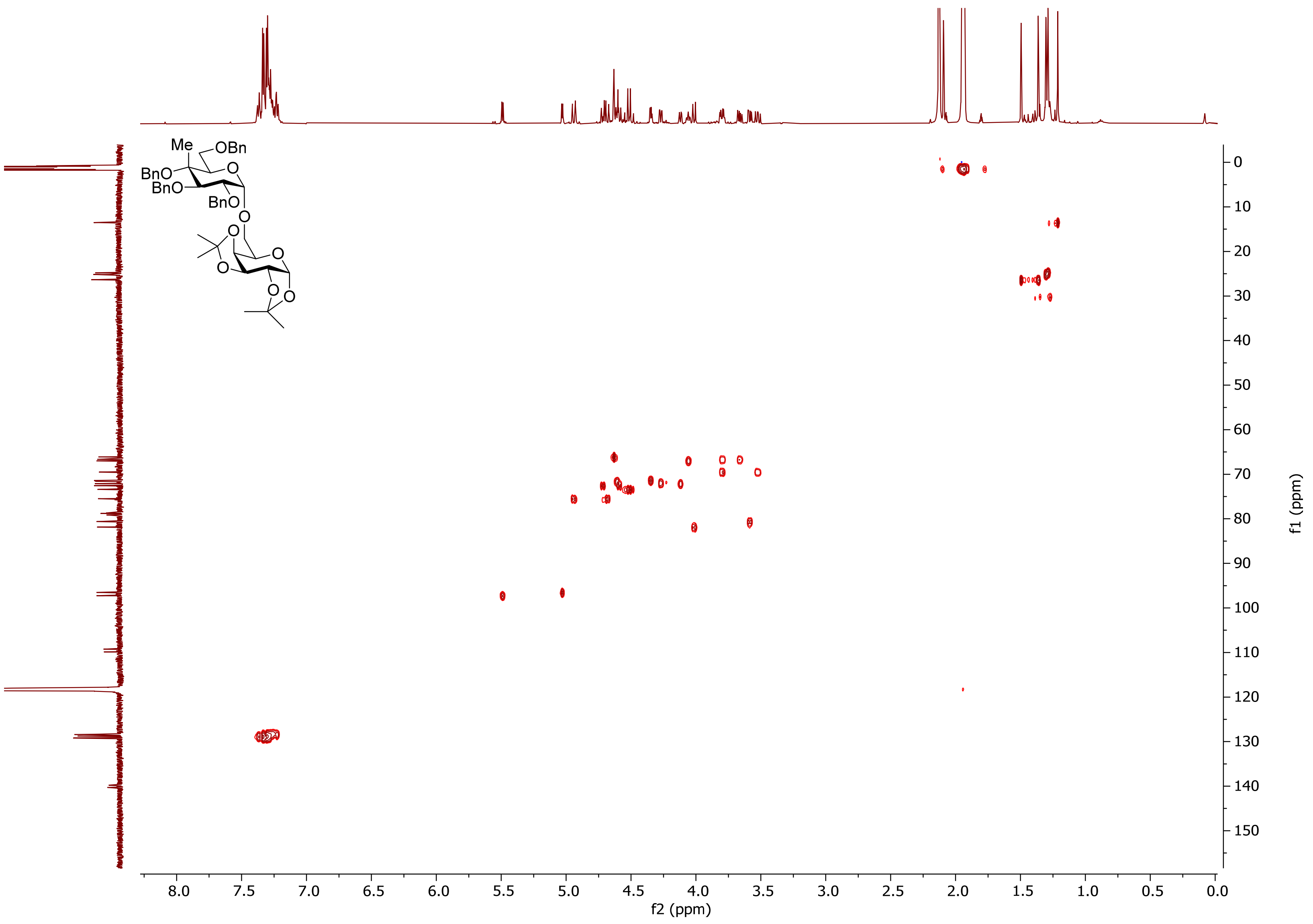

^{1}H NMR (500 MHz, CDCl$_3$) spectrum of 6-*O*-(2,3,4,6-tetra-*O*-benzyl-4-*C*-methyl-*β*-D-glucopyranosyl)-1,2:3,4-di-*O*-isopropylidene-*α*-D-galactopyranose (34β)

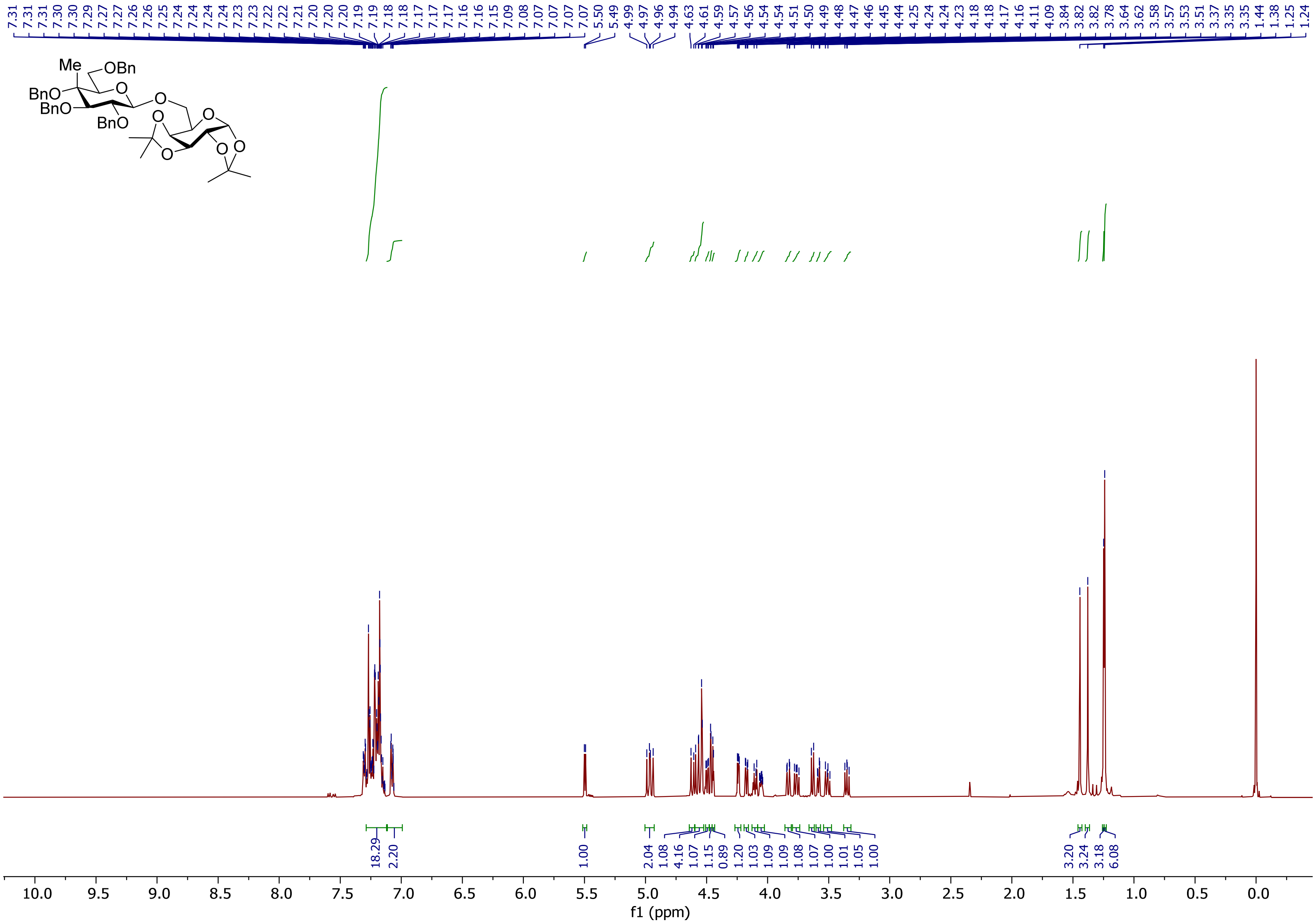

$^{13}C\{^{1}H\}$ NMR (126 MHz, $CDCl_3$) spectrum of 6-*O*-(2,3,4,6-tetra-*O*-benzyl-4-*C*-methyl-*β*-D-glucopyranosyl)-1,2:3,4-di-*O*-isopropylidene-*α*-D-galactopyranose (34β)

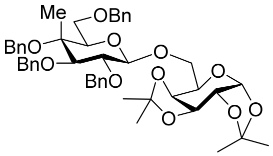

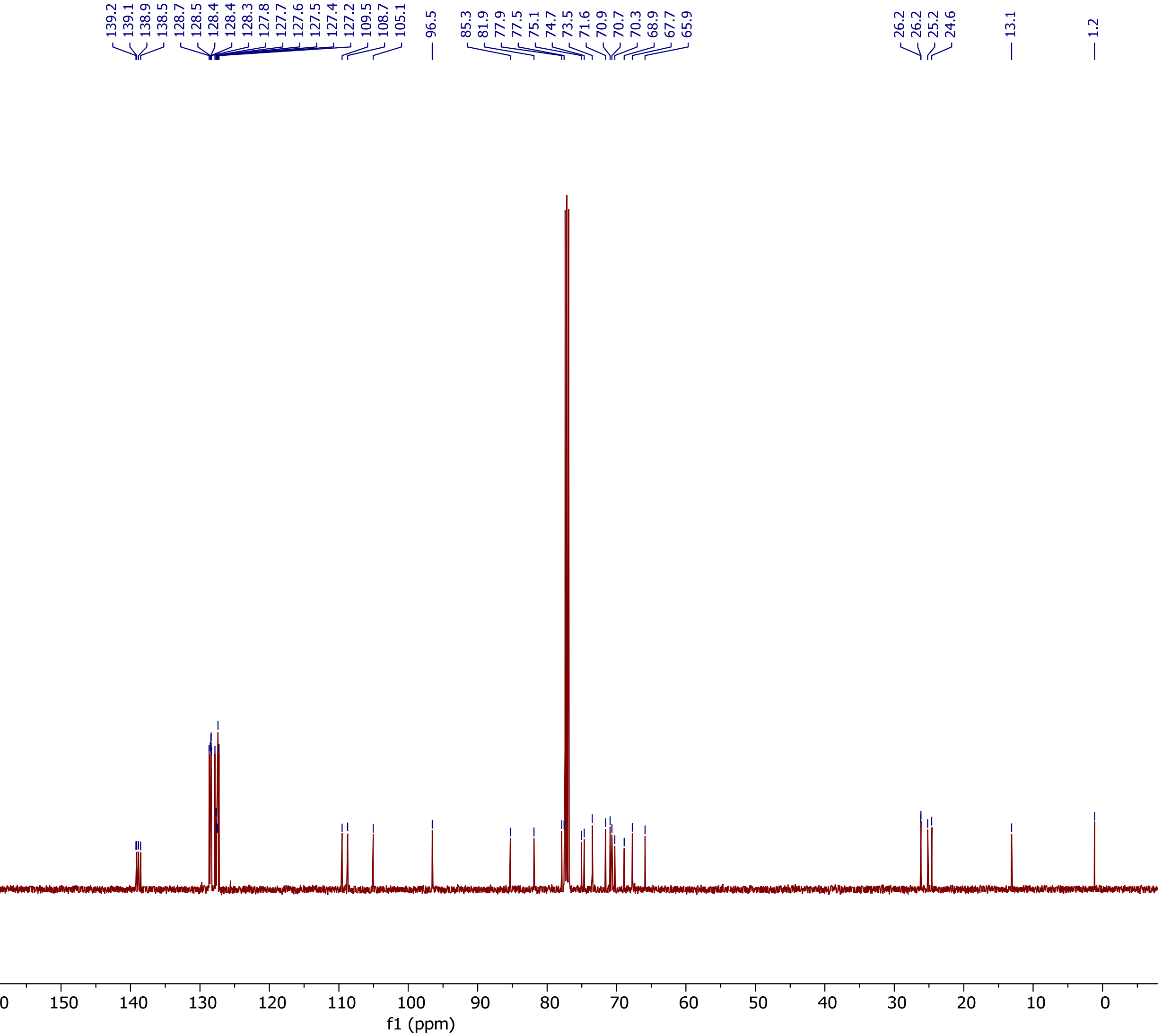

COSY NMR (500 MHz, $CDCl_3$) spectrum of 6-*O*-(2,3,4,6-tetra-*O*-benzyl-4-*C*-methyl-*β*-D-glucopyranosyl)-1,2:3,4-di-*O*-isopropylidene-*α*-D-galactopyranose (34β)

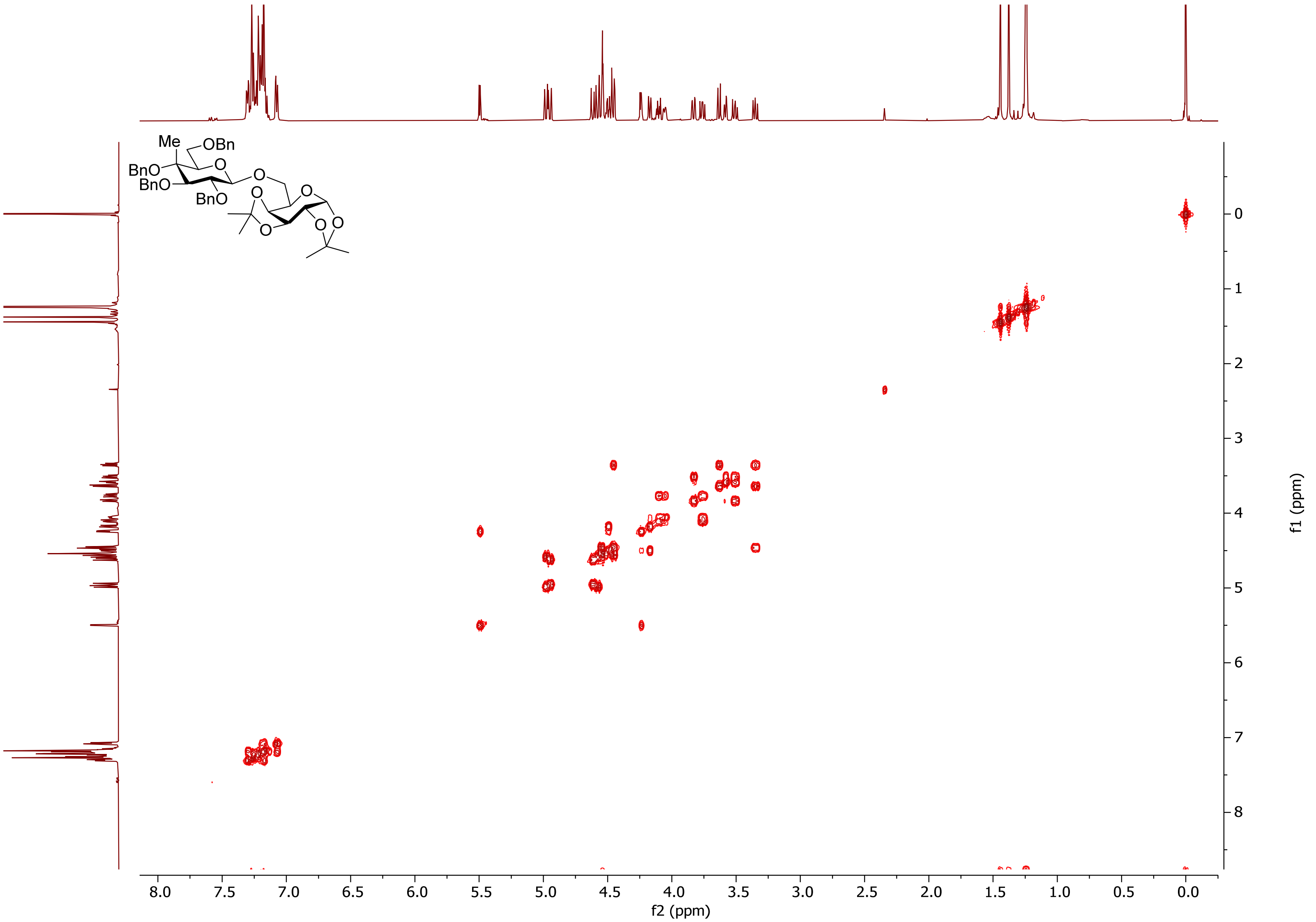

HSQC NMR (500 MHz, $CDCl_3$) spectrum of 6-*O*-(2,3,4,6-tetra-*O*-benzyl-4-*C*-methyl-*β*-D-glucopyranosyl)-1,2:3,4-di-*O*-isopropylidene-*α*-D-galactopyranose (34β)

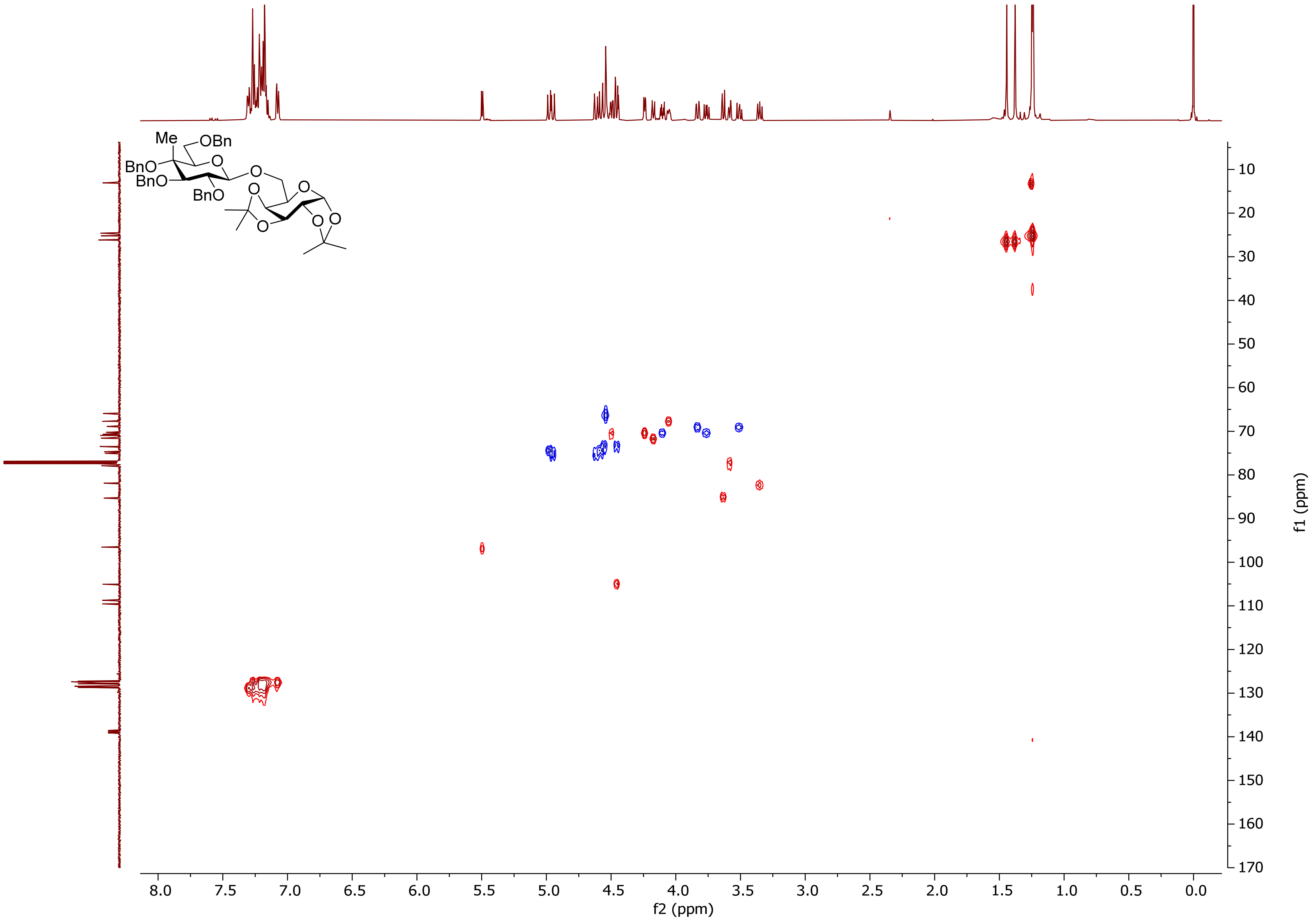

^{1}H NMR (500 MHz, $CDCl_3$) spectrum of 1,6-anhydro-2,3,4-tri-*O*-benzyl-4-*C*-methyl-*β*-D-glucopyranose (35)

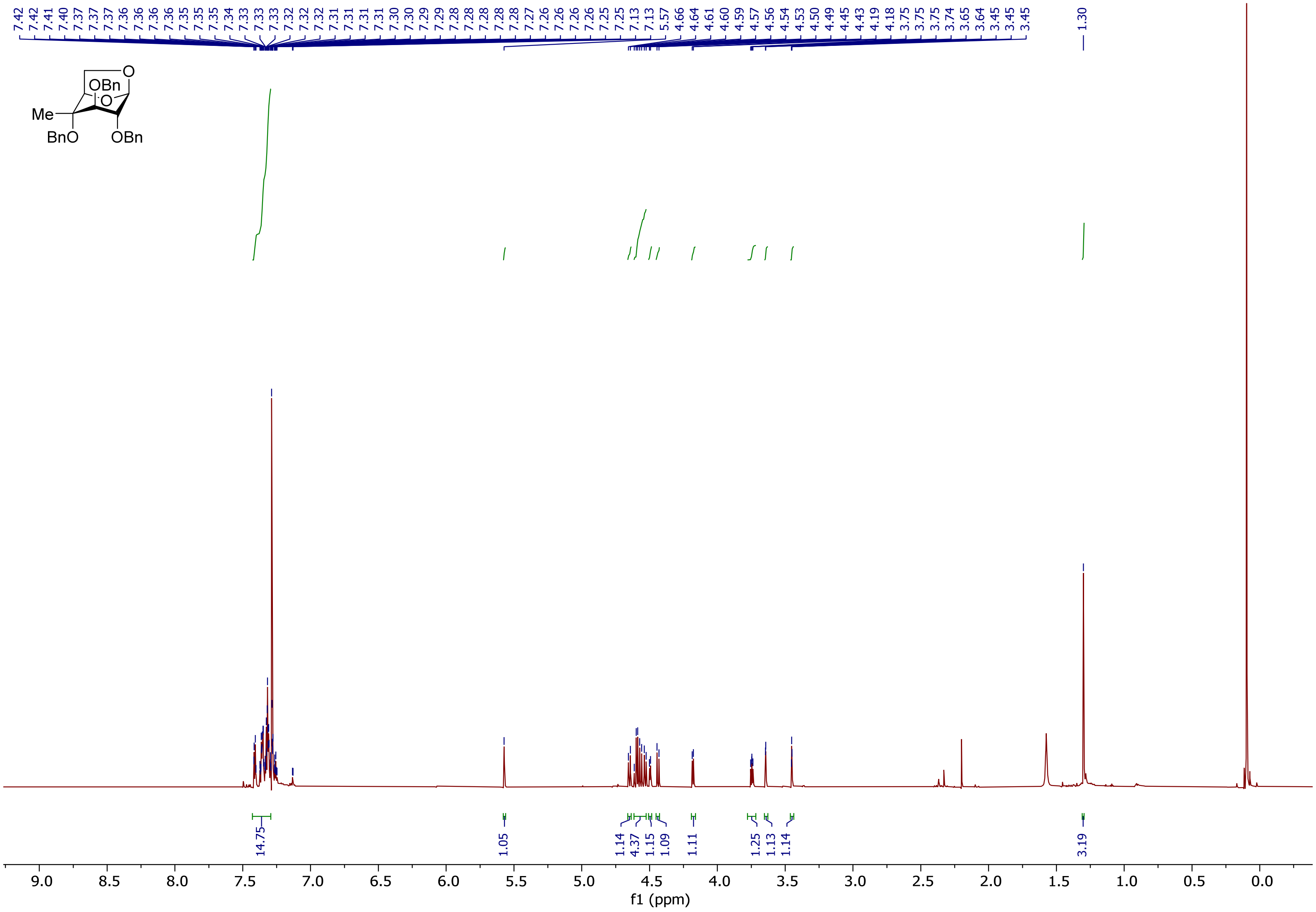

$^{13}C\{^{1}H\}$ NMR (126 MHz, $CDCl_3$) spectrum of 1,6-anhydro-2,3,4-tri-*O*-benzyl-4-*C*-methyl-*β*-D-glucopyranose (35)

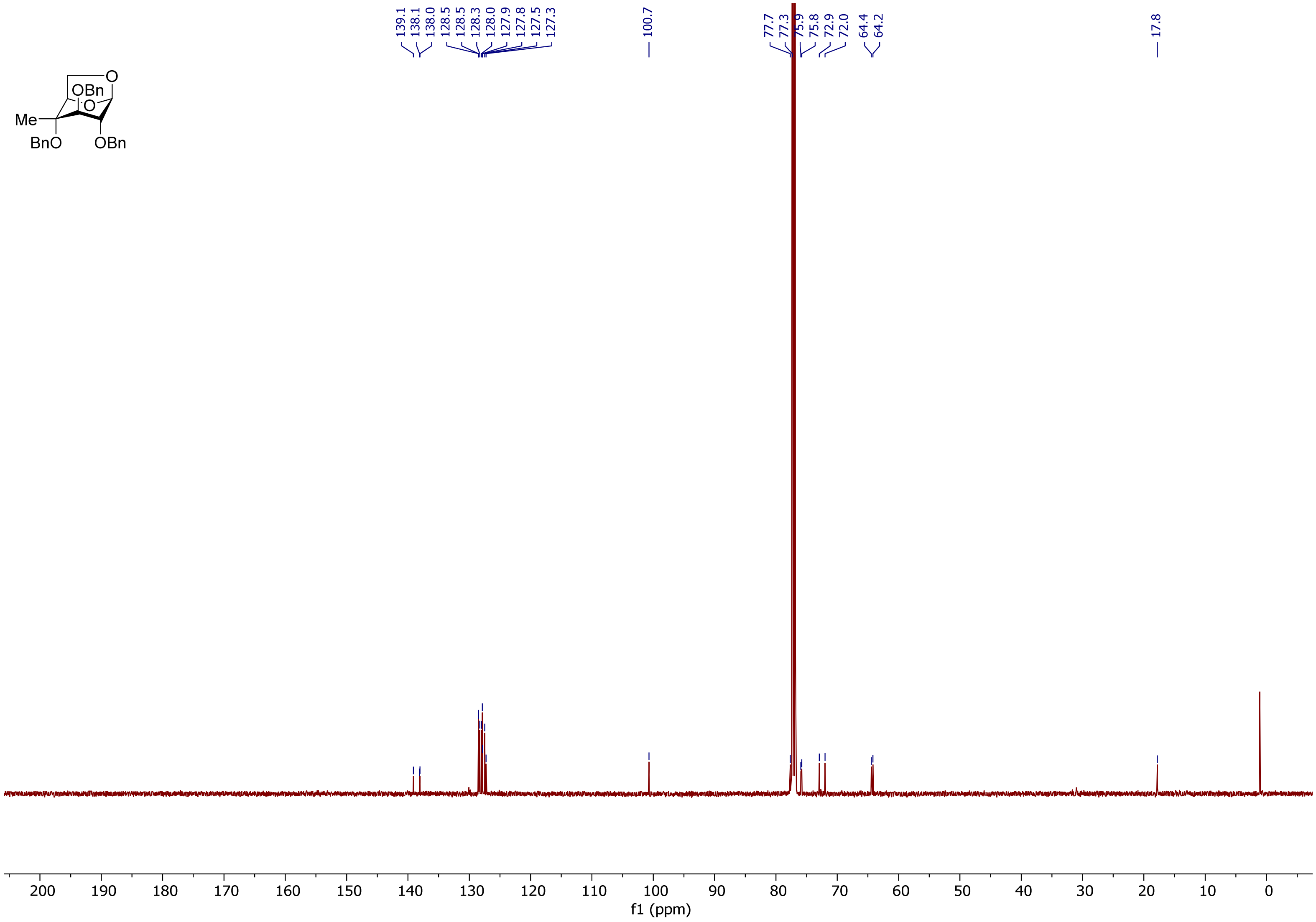

COSY NMR (500 MHz, $CDCl_3$) spectrum of 1,6-anhydro-2,3,4-tri-*O*-benzyl-4-*C*-methyl-*β*-D-glucopyranose (35)

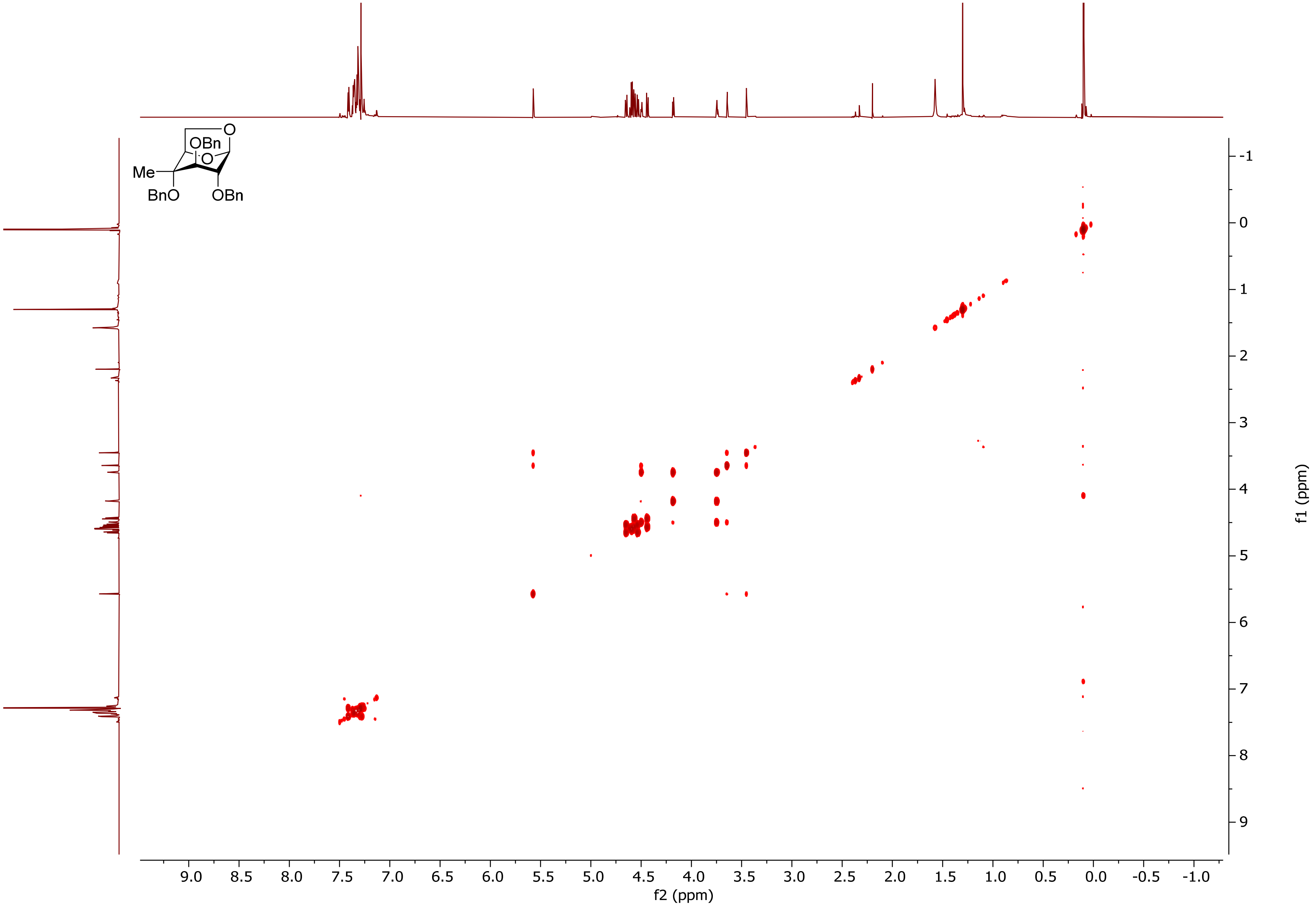

HSQC NMR (500 MHz, $CDCl_3$) spectrum of 1,6-anhydro-2,3,4-tri-*O*-benzyl-4-*C*-methyl-*β*-D-glucopyranose (35)

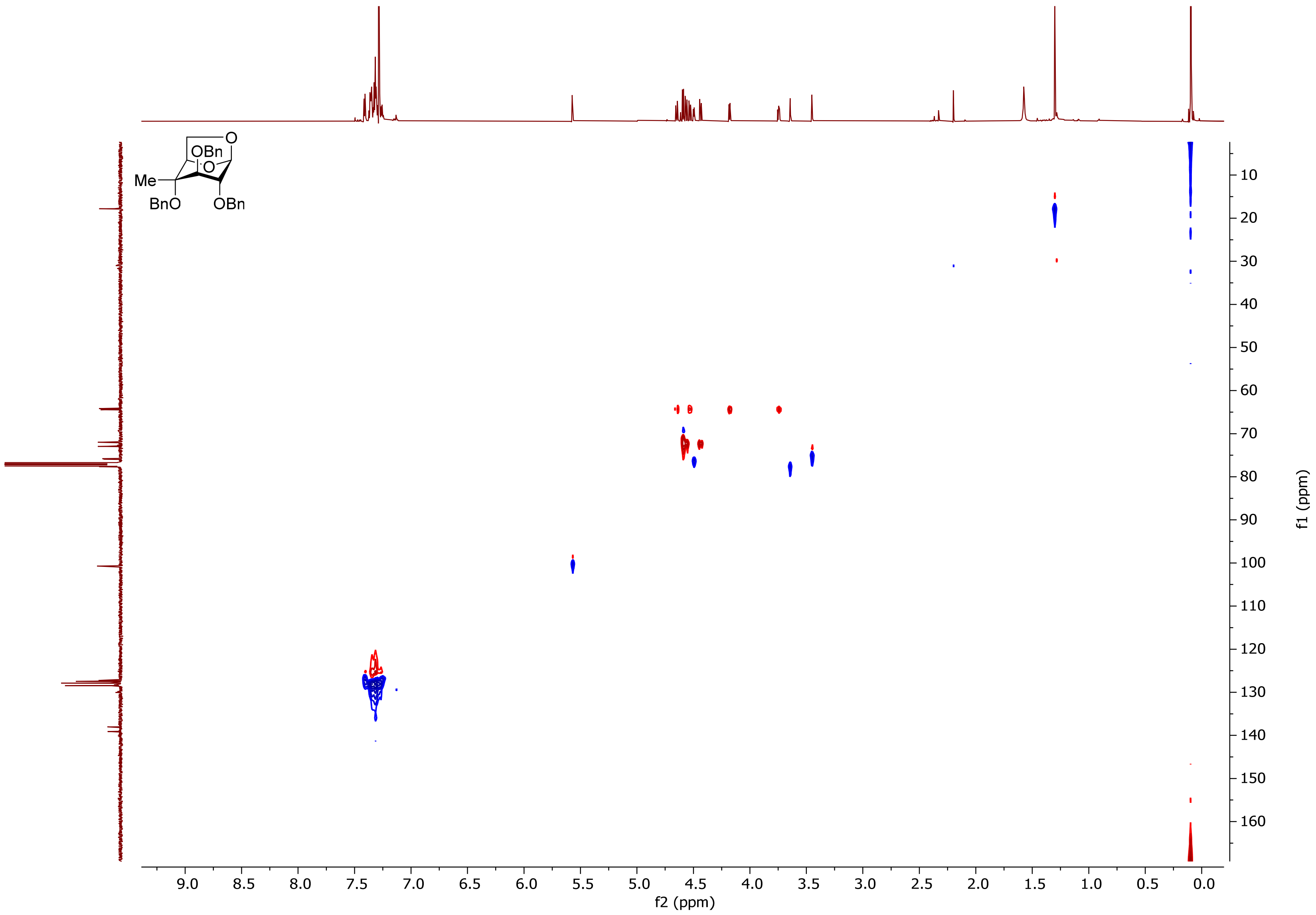

HMBC NMR (500 MHz, $CDCl_3$) spectrum of 1,6-anhydro-2,3,4-tri-*O*-benzyl-4-*C*-methyl-*β*-D-glucopyranose (35)

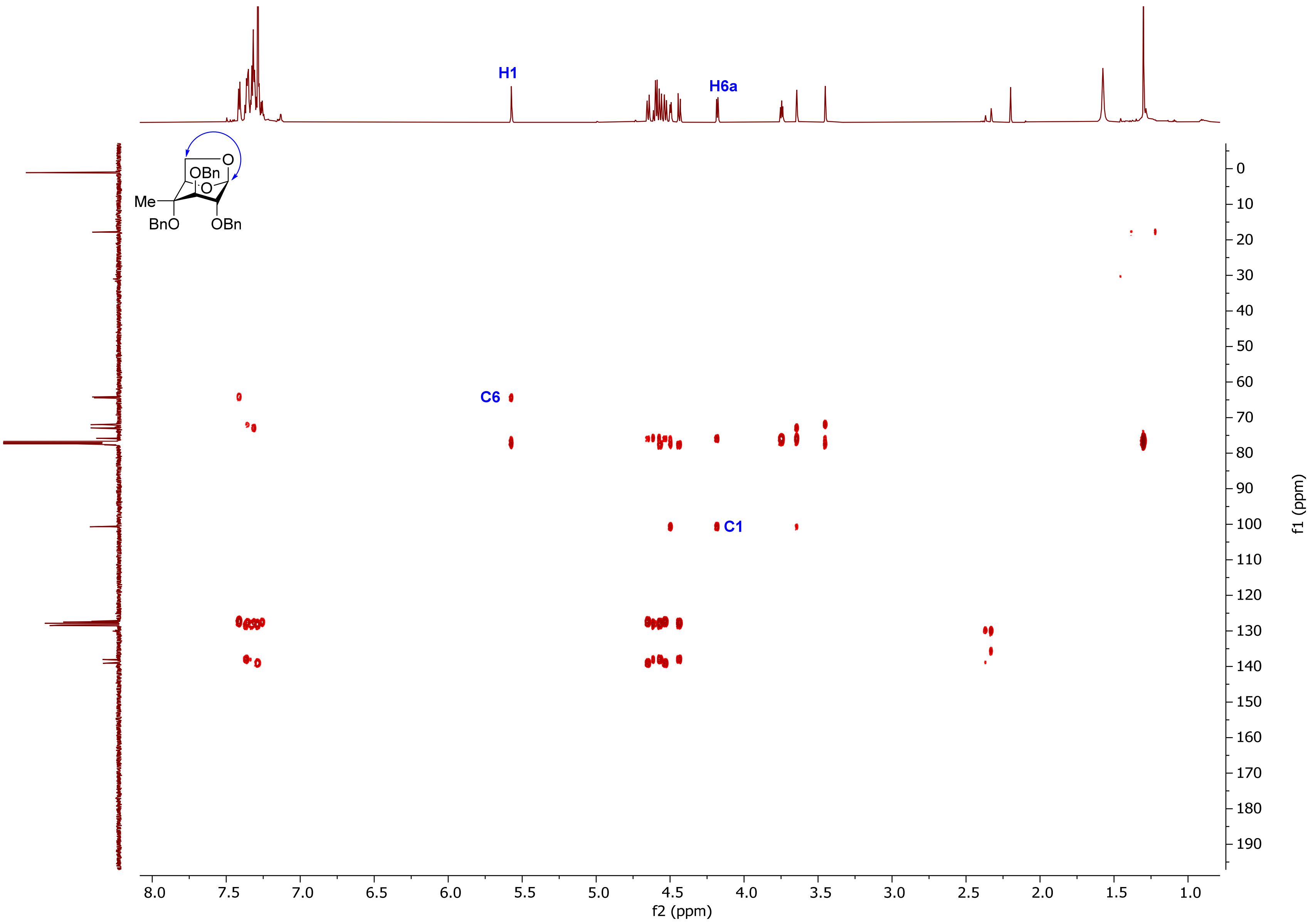

^{1}H NMR (500 MHz, $CDCl_3$) spectrum of 6-*O*-(4-*O*-benzoyl-2,3,6-tri-*O*-benzyl-4-*C*-methyl-*α*-D-galactopyranosyl)-1,2:3,4-di-*O*-isopropylidene-*α*-D-galactopyranose (36α)

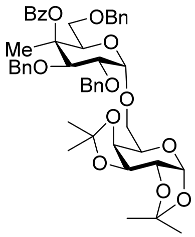

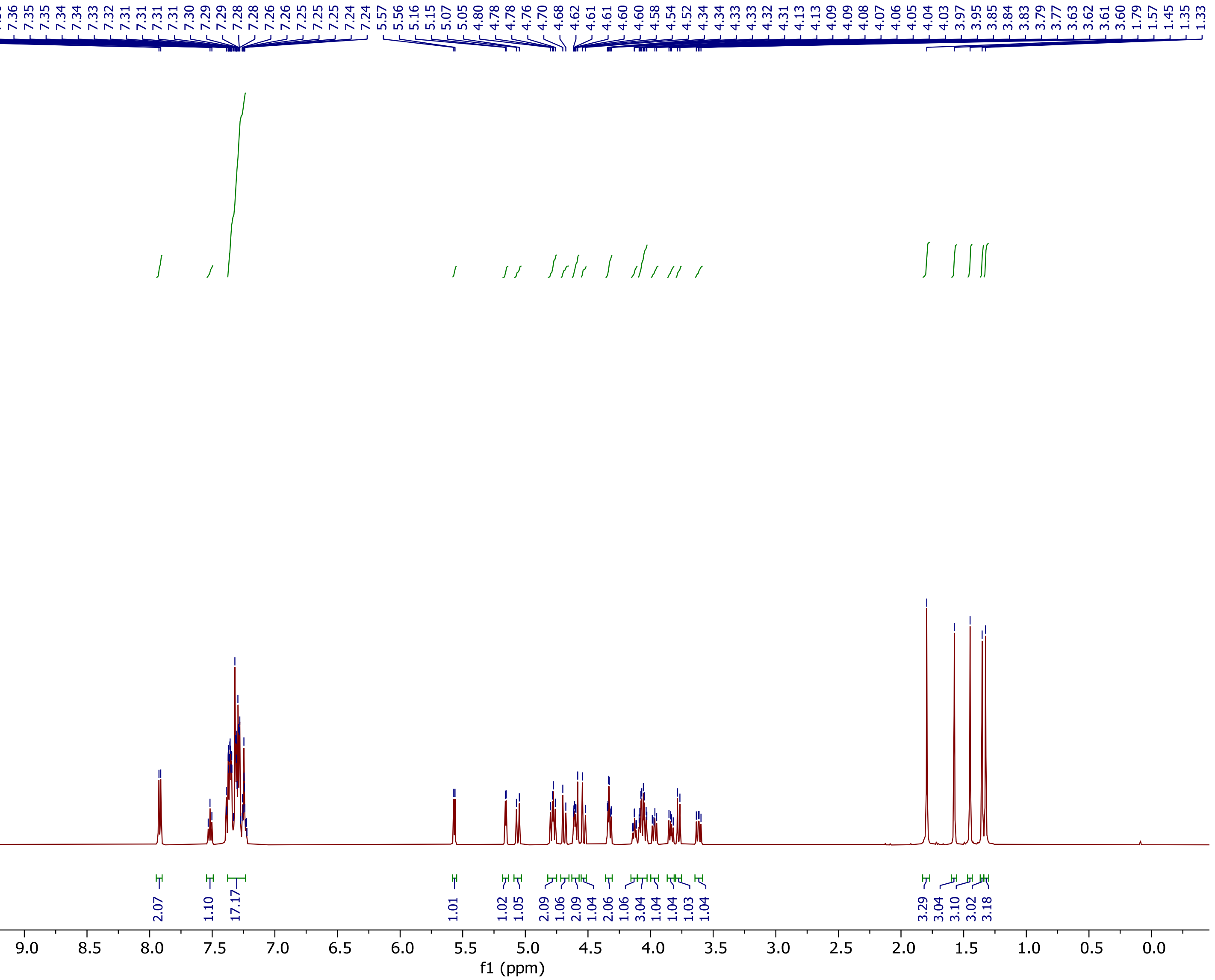

$^{13}C\{^{1}H\}$ NMR (126 MHz, $CDCl_3$) spectrum of 6-*O*-(4-*O*-benzoyl-2,3,6-tri-*O*-benzyl-4-*C*-methyl-*α*-D-galactopyranosyl)-1,2:3,4-di-*O*-isopropylidene-*α*-D-galactopyranose (36α)

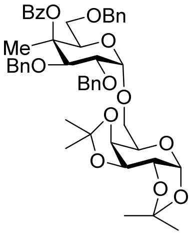

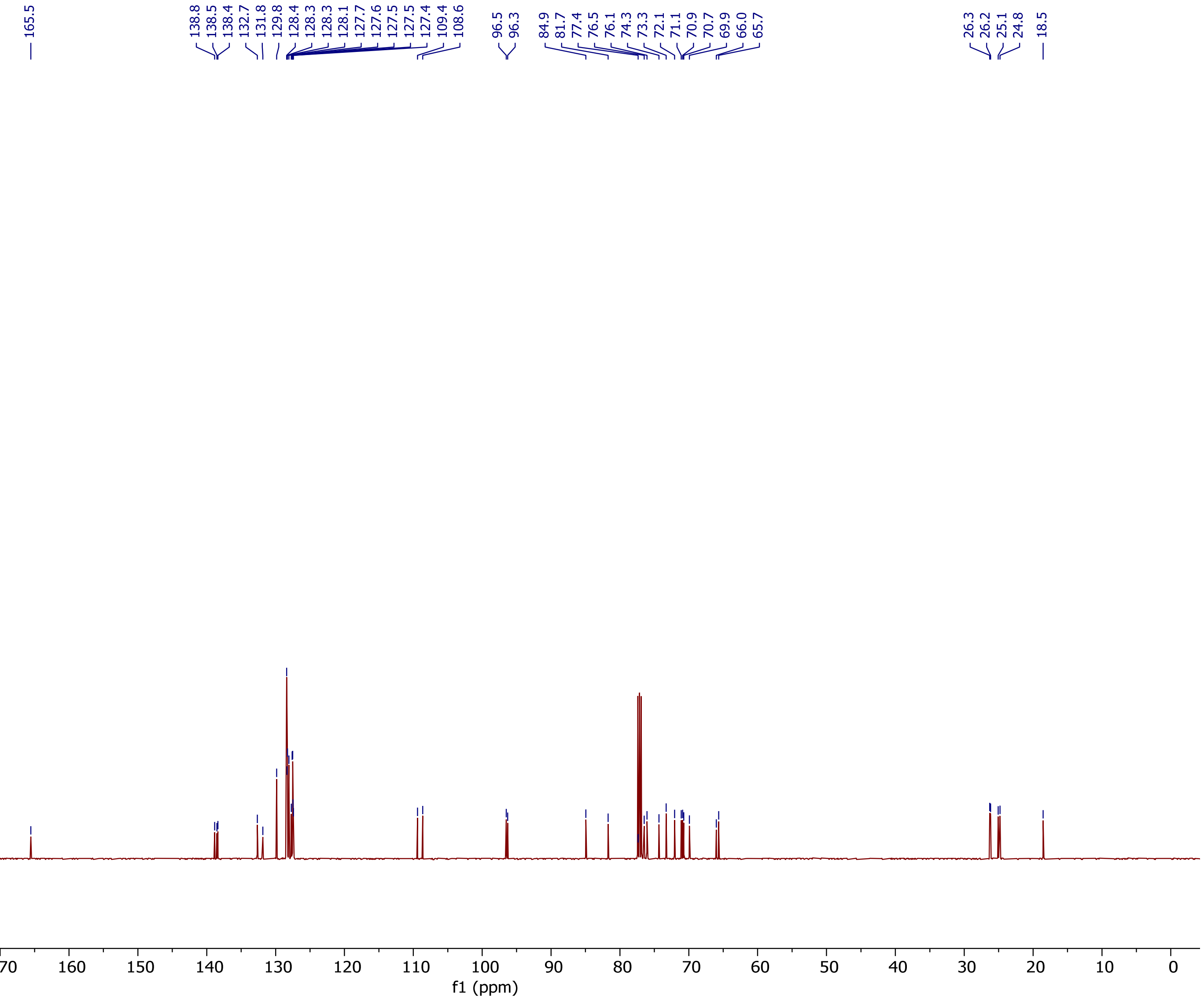

COSY NMR (500 MHz, CDCl₃) spectrum of 6-*O*-(4-*O*-benzoyl-2,3,6-tri-*O*-benzyl-4-*C*-methyl-*α*-D-galactopyranosyl)-1,2:3,4-di-*O*-isopropylidene-*α*-D-galactopyranose (36α)

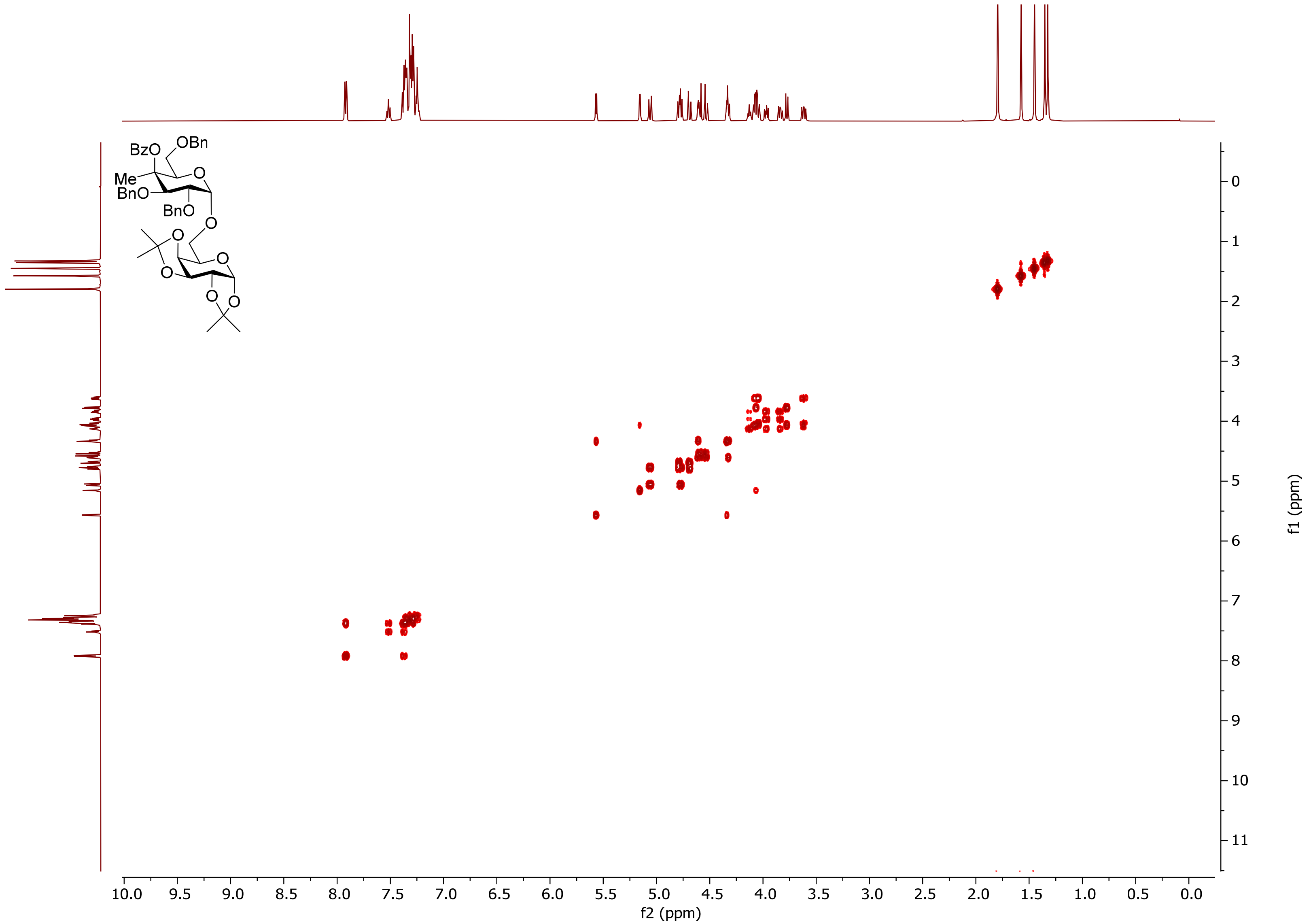

HSQC NMR (500 MHz, $CDCl_3$) spectrum of 6-*O*-(4-*O*-benzoyl-2,3,6-tri-*O*-benzyl-4-*C*-methyl-*α*-D-galactopyranosyl)-1,2:3,4-di-*O*-isopropylidene-*α*-D-galactopyranose (36α)

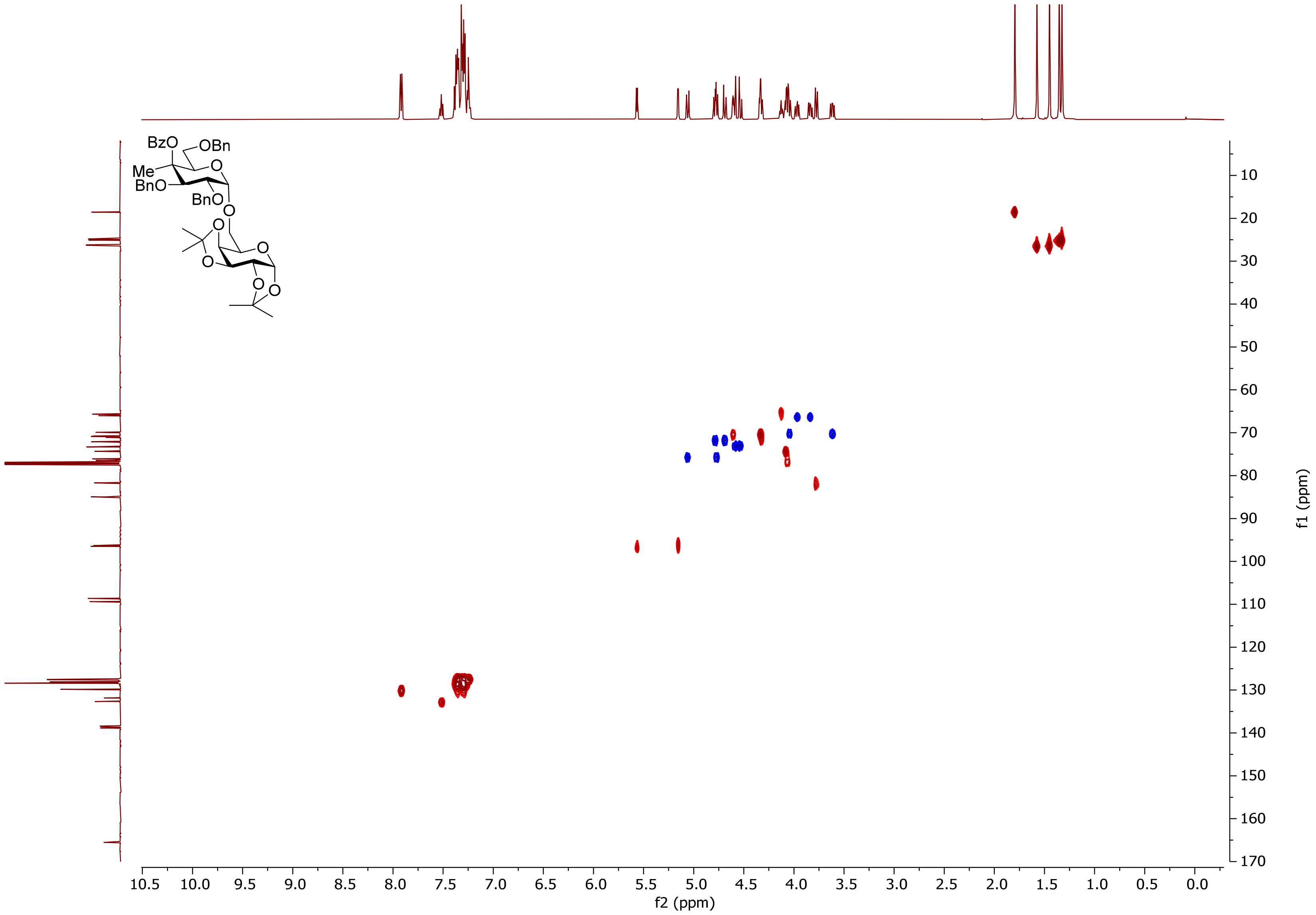

^{1}H NMR (500 MHz, $CDCl_3$) spectrum of 6-*O*-(4-*O*-benzoyl-2,3,6-tri-*O*-benzyl-4-*C*-methyl-*β*-D-glucopyranosyl)-1,2:3,4-di-*O*-isopropylidene-*α*-D-galactopyranose (37β)

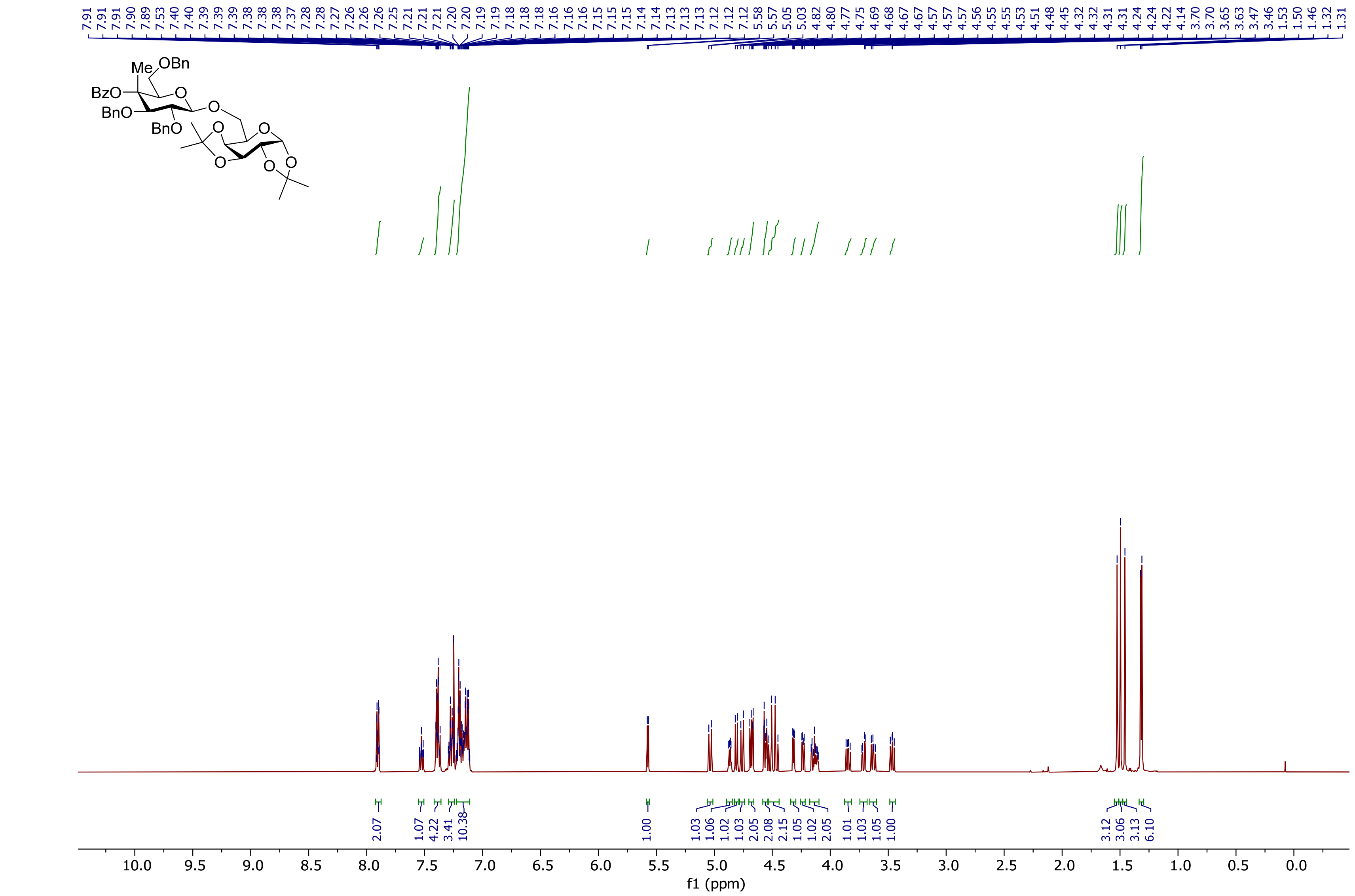

$^{13}C\{^{1}H\}$ NMR (126 MHz, $CDCl_3$) spectrum of 6-*O*-(4-*O*-benzoyl-2,3,6-tri-*O*-benzyl-4-*C*-methyl-*β*-D-glucopyranosyl)-1,2:3,4-di-*O*-isopropylidene-*α*-D-galactopyranose (37β)

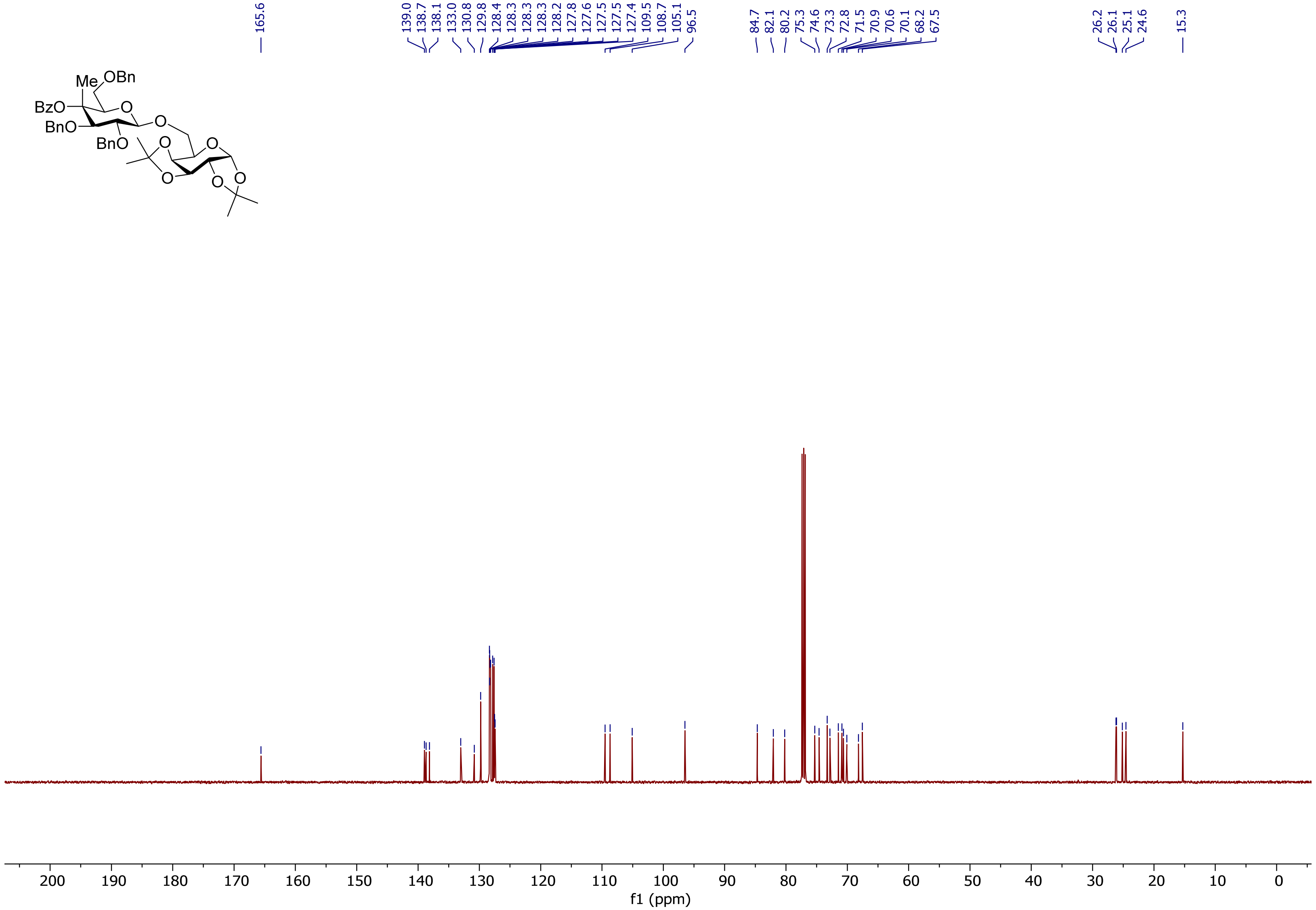

COSY NMR (500 MHz, $CDCl_3$) spectrum of 6-*O*-(4-*O*-benzoyl-2,3,6-tri-*O*-benzyl-4-*C*-methyl-*β*-D-glucopyranosyl)-1,2:3,4-di-*O*-isopropylidene-*α*-D-galactopyranose (37β)

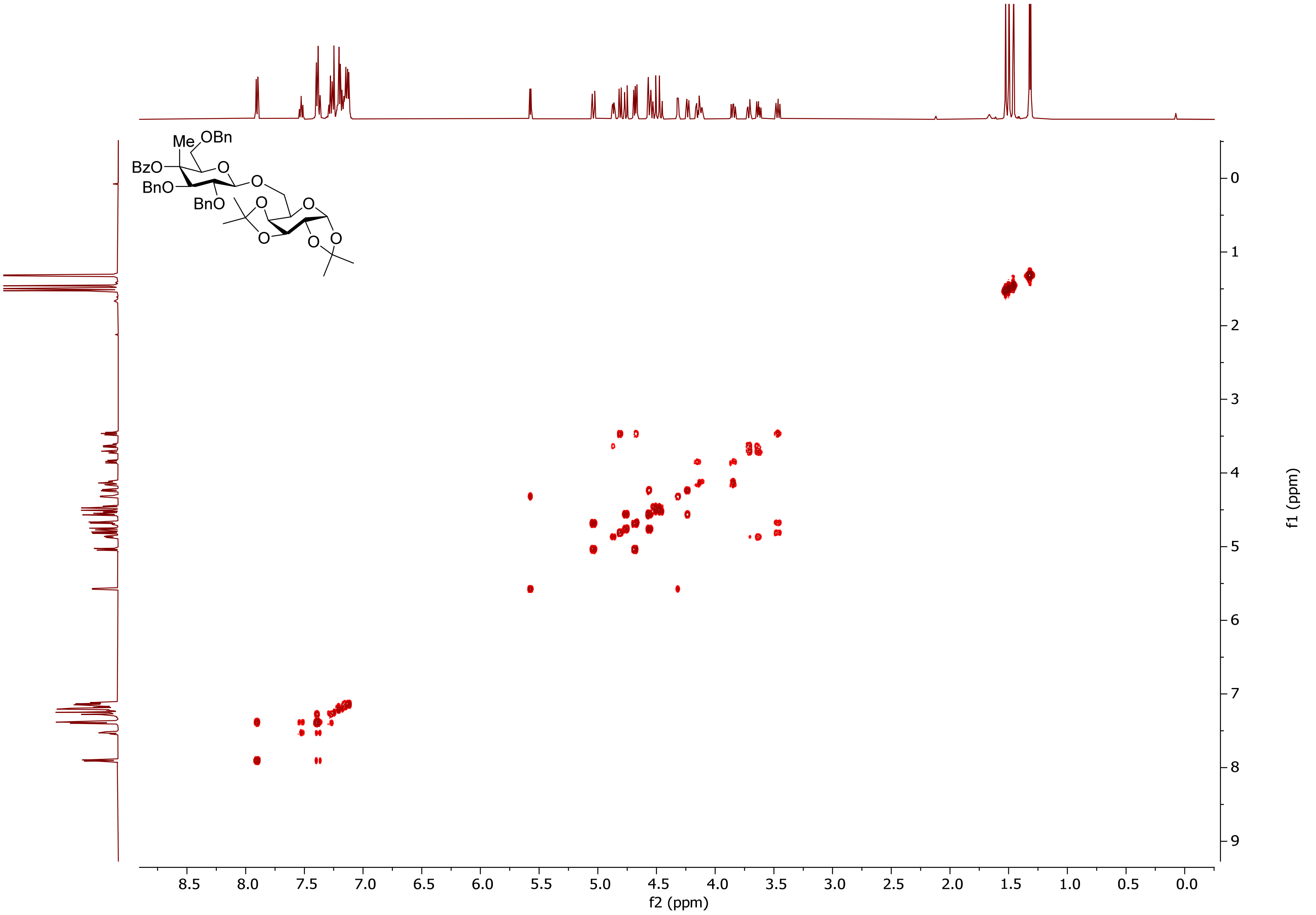

HSQC NMR (500 MHz, $CDCl_3$) spectrum of 6-*O*-(4-*O*-benzoyl-2,3,6-tri-*O*-benzyl-4-*C*-methyl-*β*-D-glucopyranosyl)-1,2:3,4-di-*O*-isopropylidene-*α*-D-galactopyranose (37β)

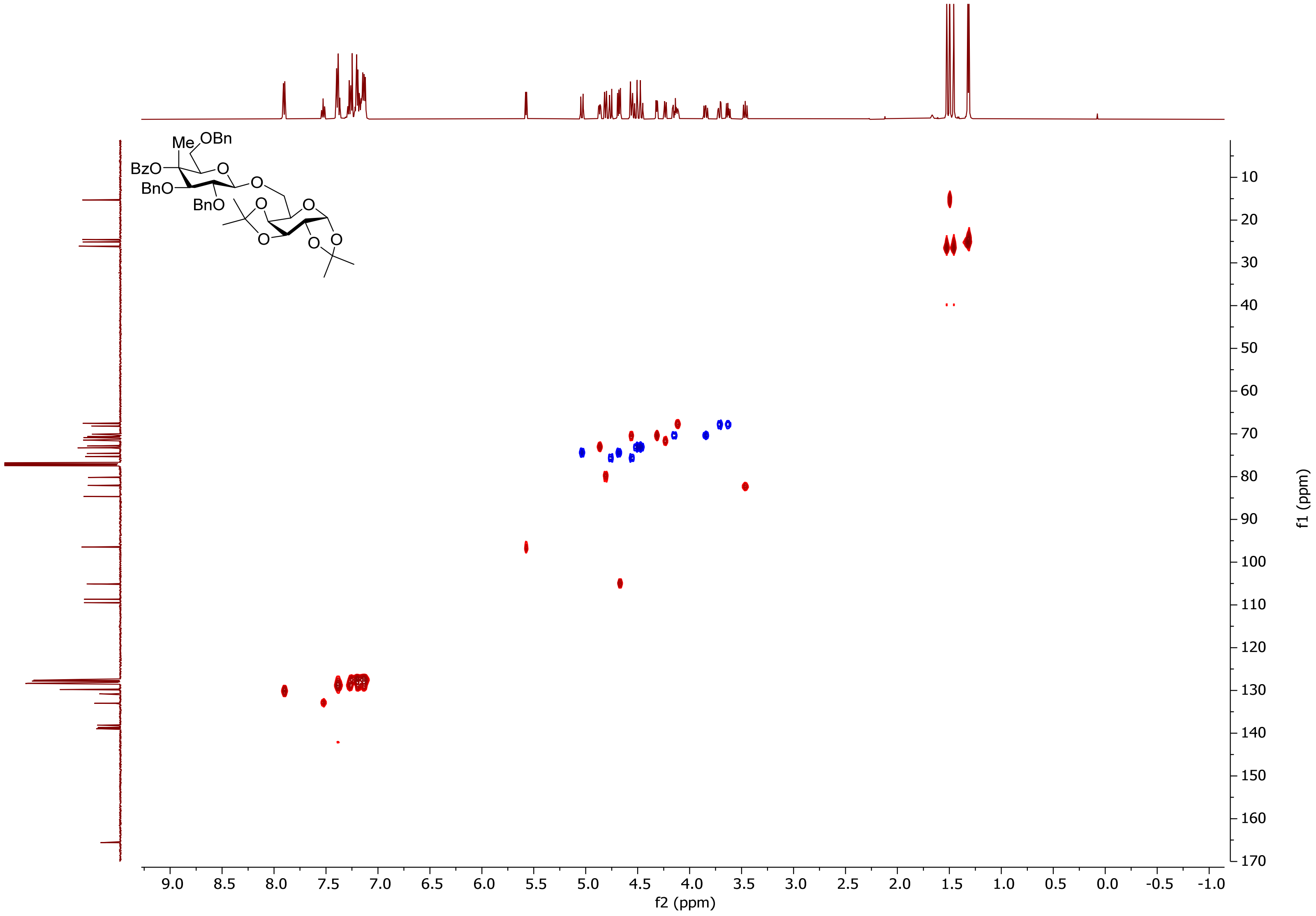

^{1}H NMR (500 MHz, CDCl$_3$) spectrum of 1,6-anhydro-4-*O*-benzoyl-2,3-di-*O*-benzyl-4-*C*-methyl-*β*-D-glucopyranose (38)

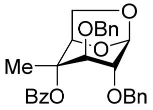

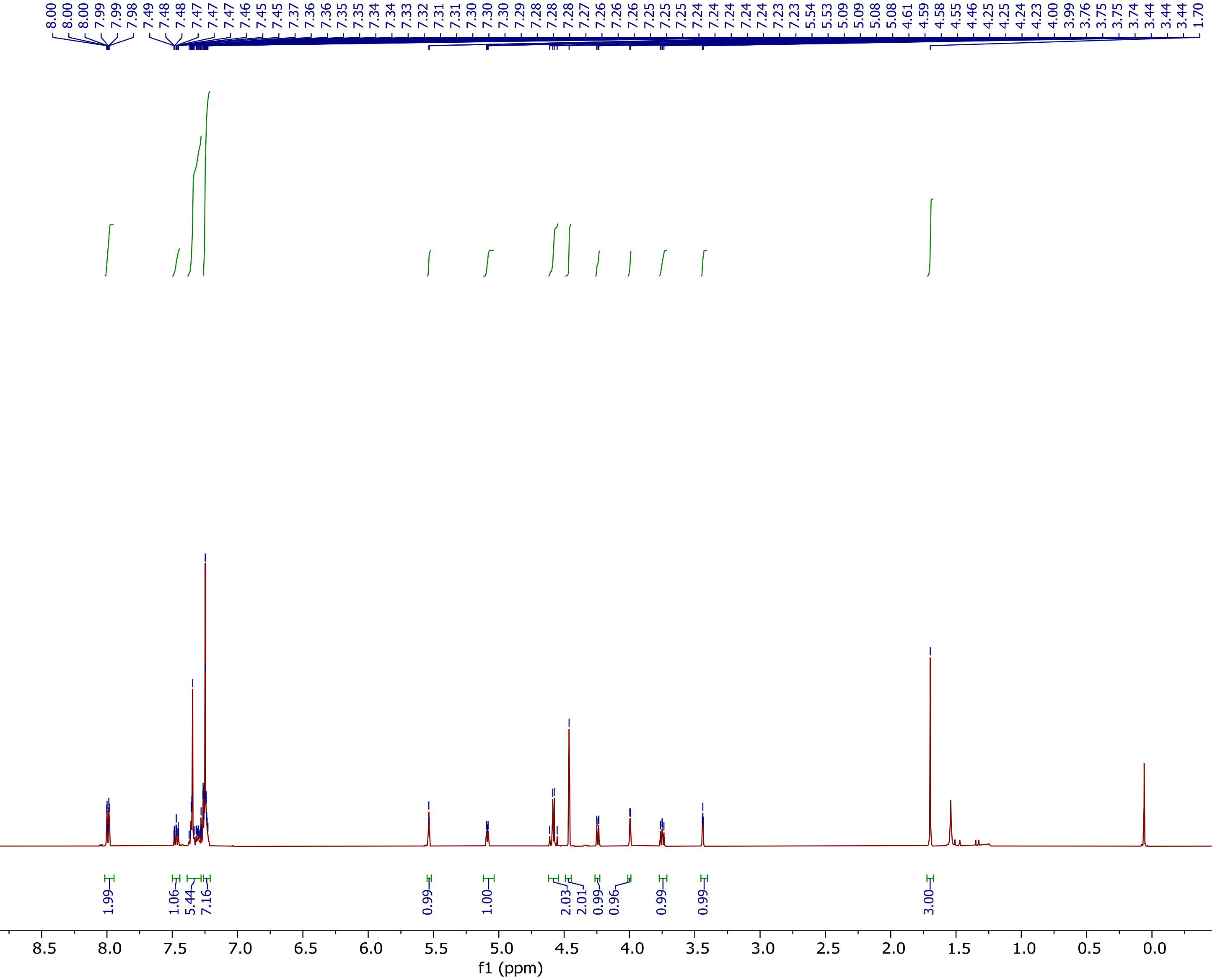

$^{13}C\{^1H\}$ **NMR (126 MHz, CDCl$_3$) spectrum of 1,6-anhydro-4-*O*-benzoyl-2,3-di-*O*-benzyl-4-*C*-methyl-*β*-D-glucopyranose (38)**

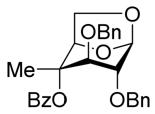

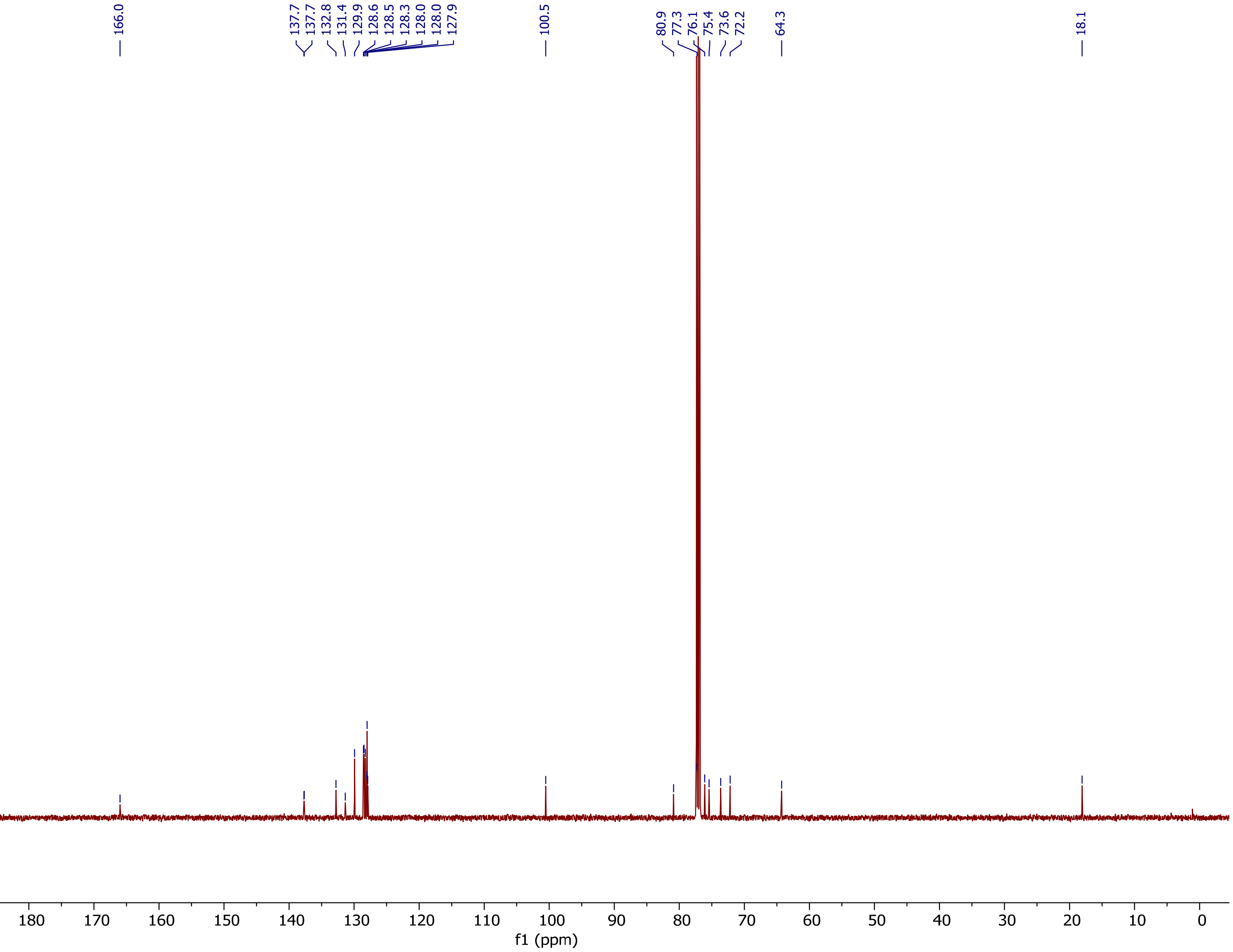

COSY NMR (500 MHz, $CDCl_3$) spectrum of 1,6-anhydro-4-*O*-benzoyl-2,3-di-*O*-benzyl-4-*C*-methyl-*β*-D-glucopyranose (38)

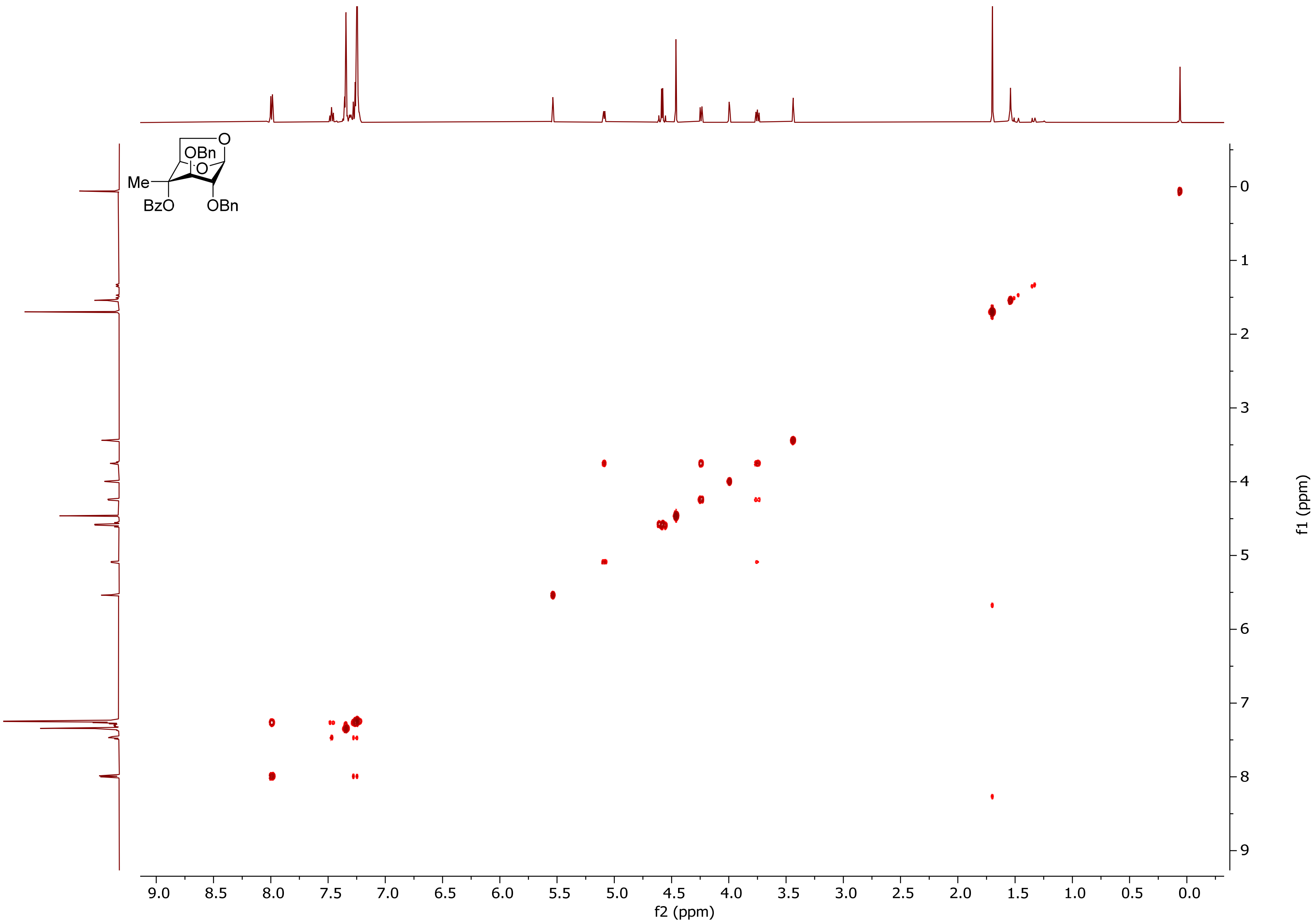

HSQC NMR (500 MHz, $CDCl_3$) spectrum of 1,6-anhydro-4-*O*-benzoyl-2,3-di-*O*-benzyl-4-*C*-methyl-*β*-D-glucopyranose (38)

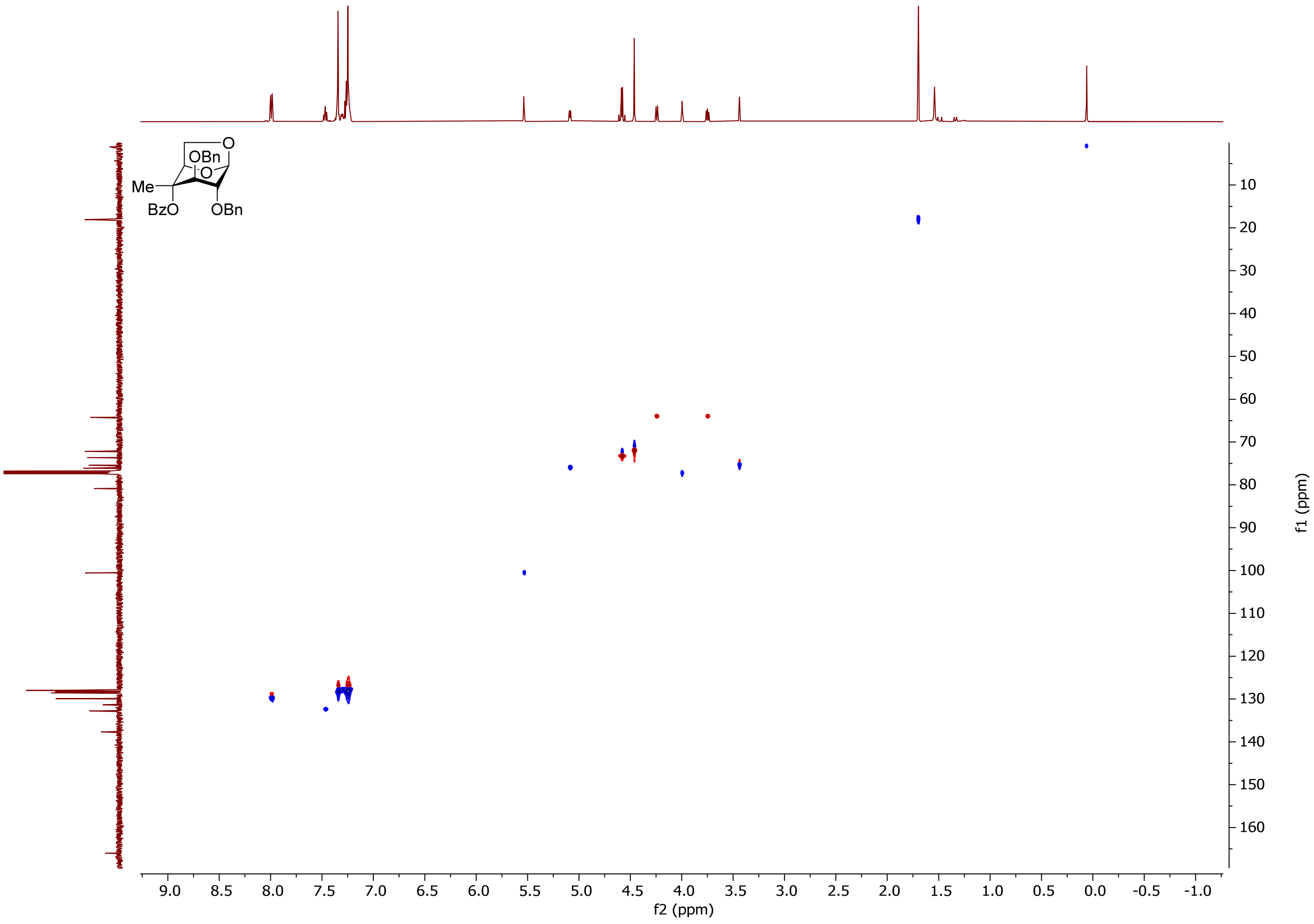

HMBC NMR (500 MHz, $CDCl_3$) spectrum of 1,6-anhydro-4-*O*-benzoyl-2,3-di-*O*-benzyl-4-*C*-methyl-*β*-D-glucopyranose (38)

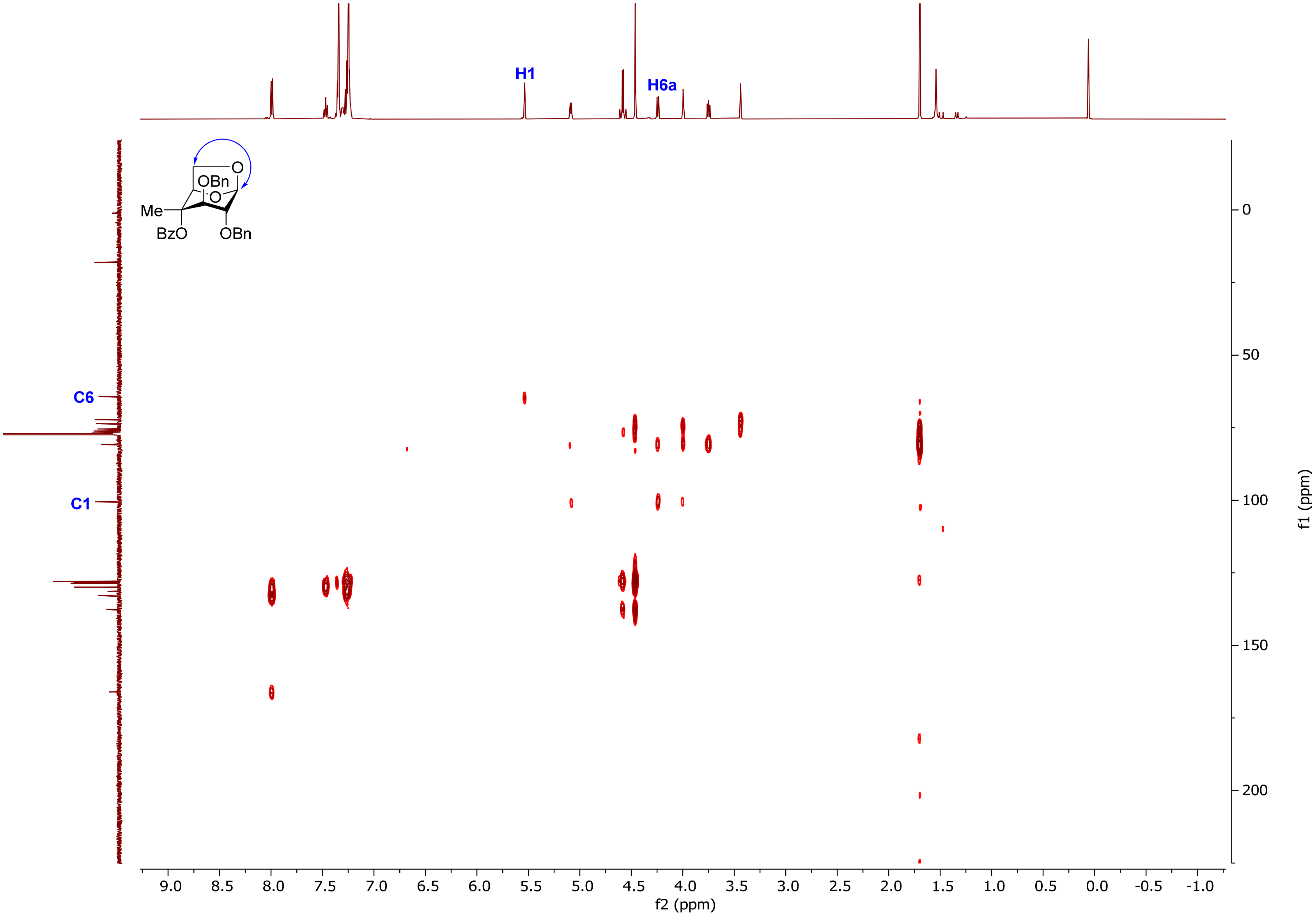

^{1}H NMR (500 MHz, CDCl$_3$) spectrum of 4-*O*-benzoyl-2,3,6-tri-*O*-benzyl-4-*C*-methyl-*α*/*β*-D-glucopyranose (39)

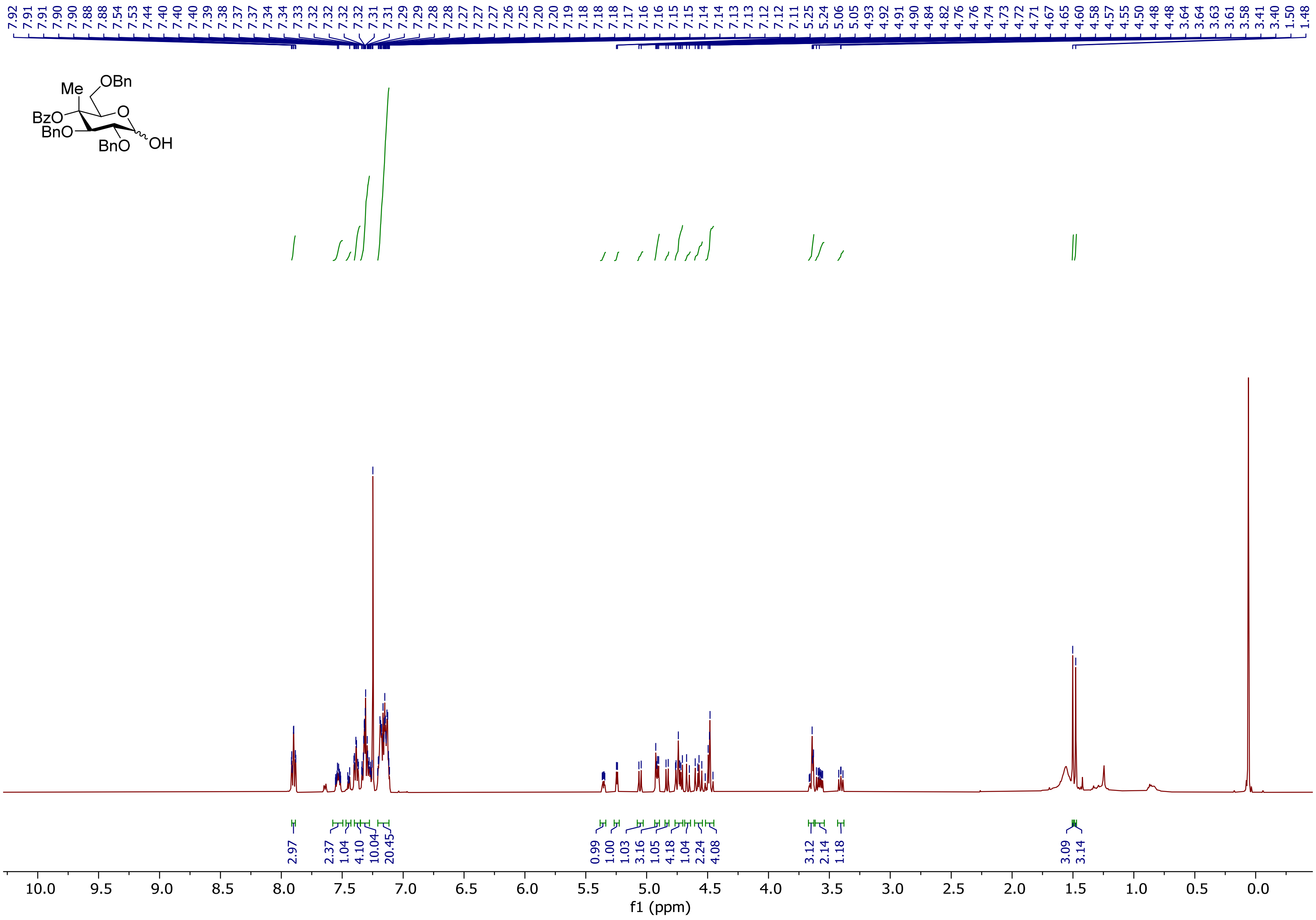

$^{13}C\{^1H\}$ NMR (126 MHz, $CDCl_3$) spectrum of 4-*O*-benzoyl-2,3,6-tri-*O*-benzyl-4-*C*-methyl-α/β-D-glucopyranose (39)

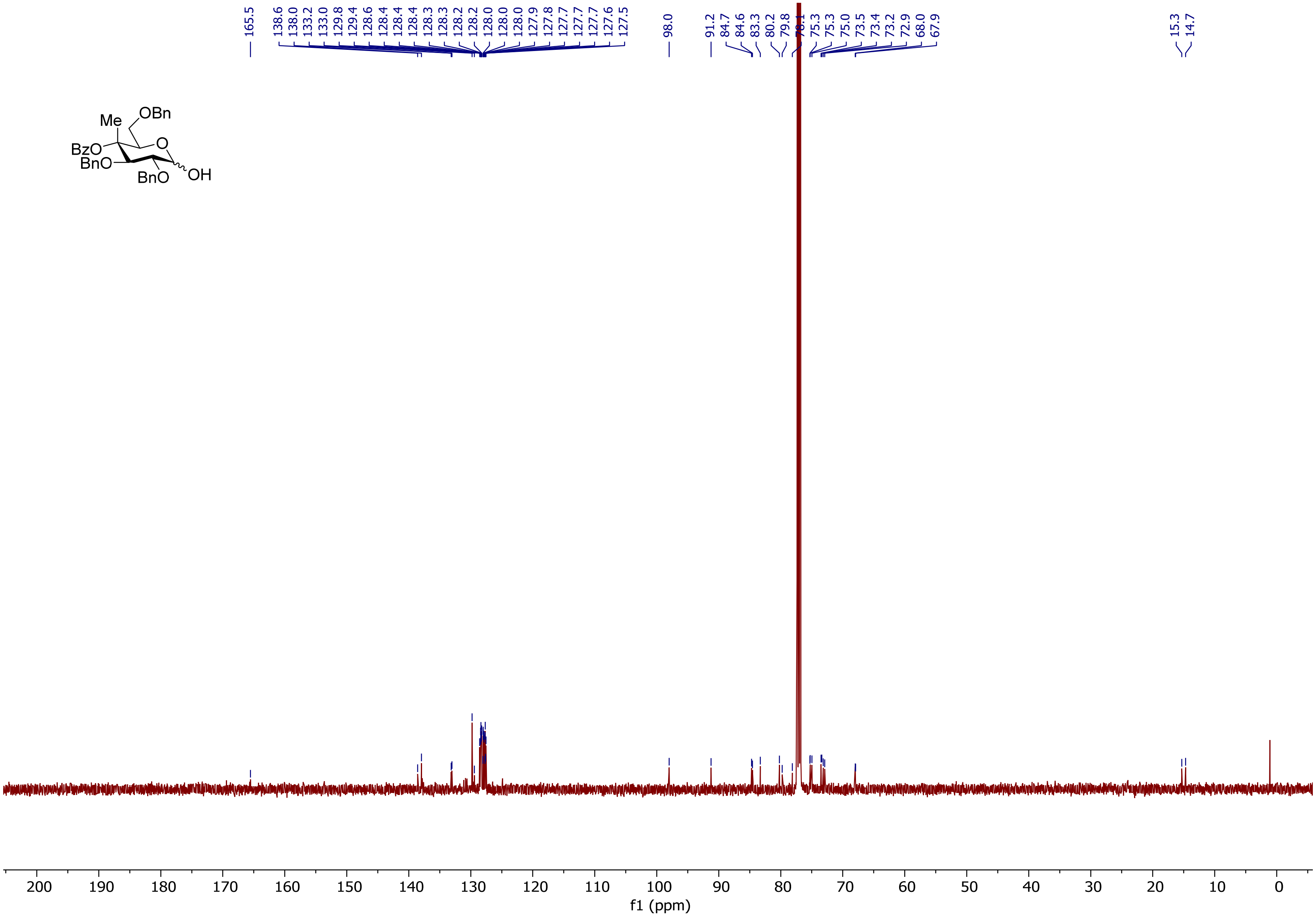

COSY NMR (500 MHz, $CDCl_3$) spectrum of 4-*O*-benzoyl-2,3,6-tri-*O*-benzyl-4-*C*-methyl-α/β-D-glucopyranose (39)

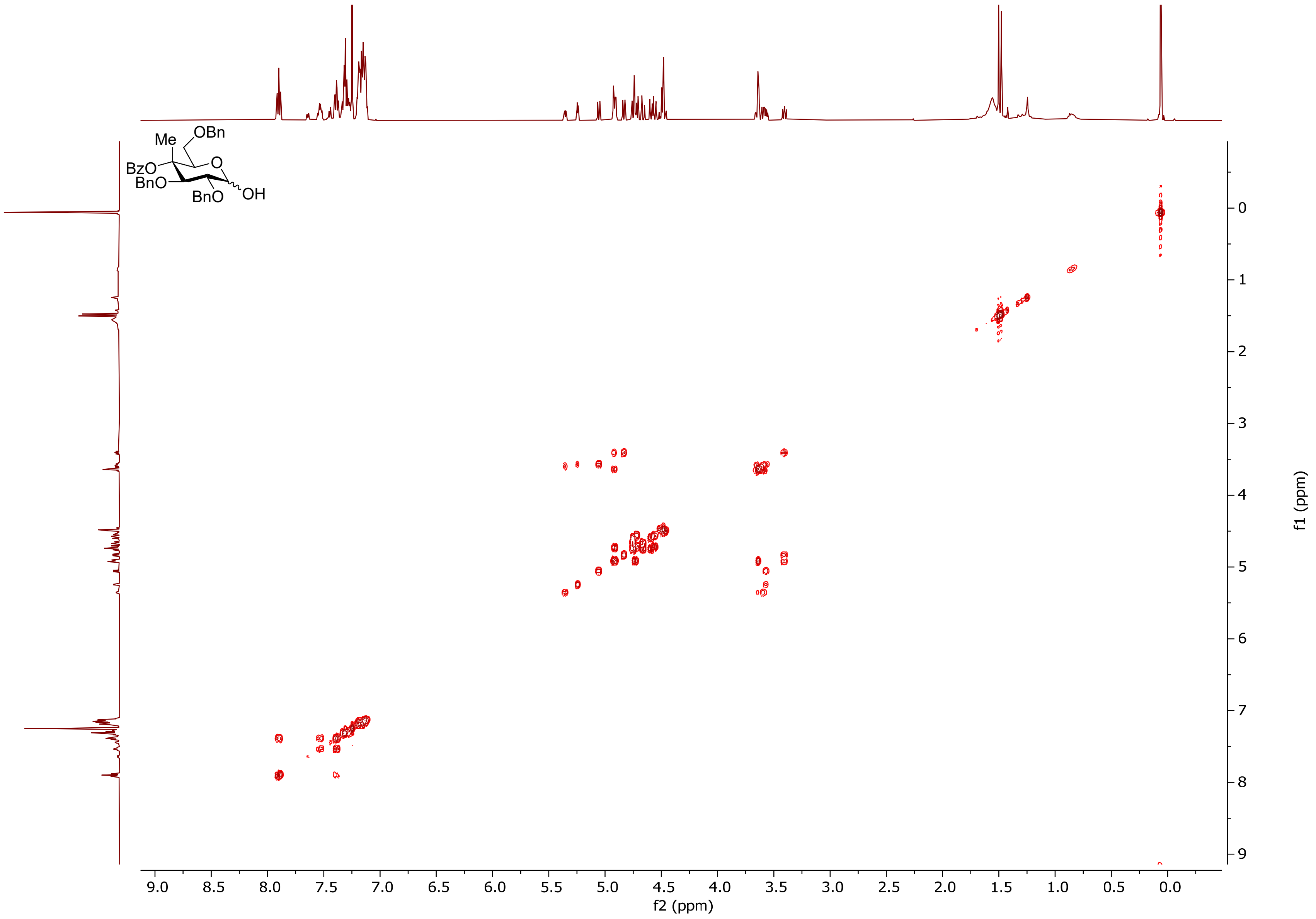

HSQC NMR (500 MHz, CDCl$_3$) spectrum of 4-*O*-benzoyl-2,3,6-tri-*O*-benzyl-4-*C*-methyl-*α*/*β*-D-glucopyranose (39)

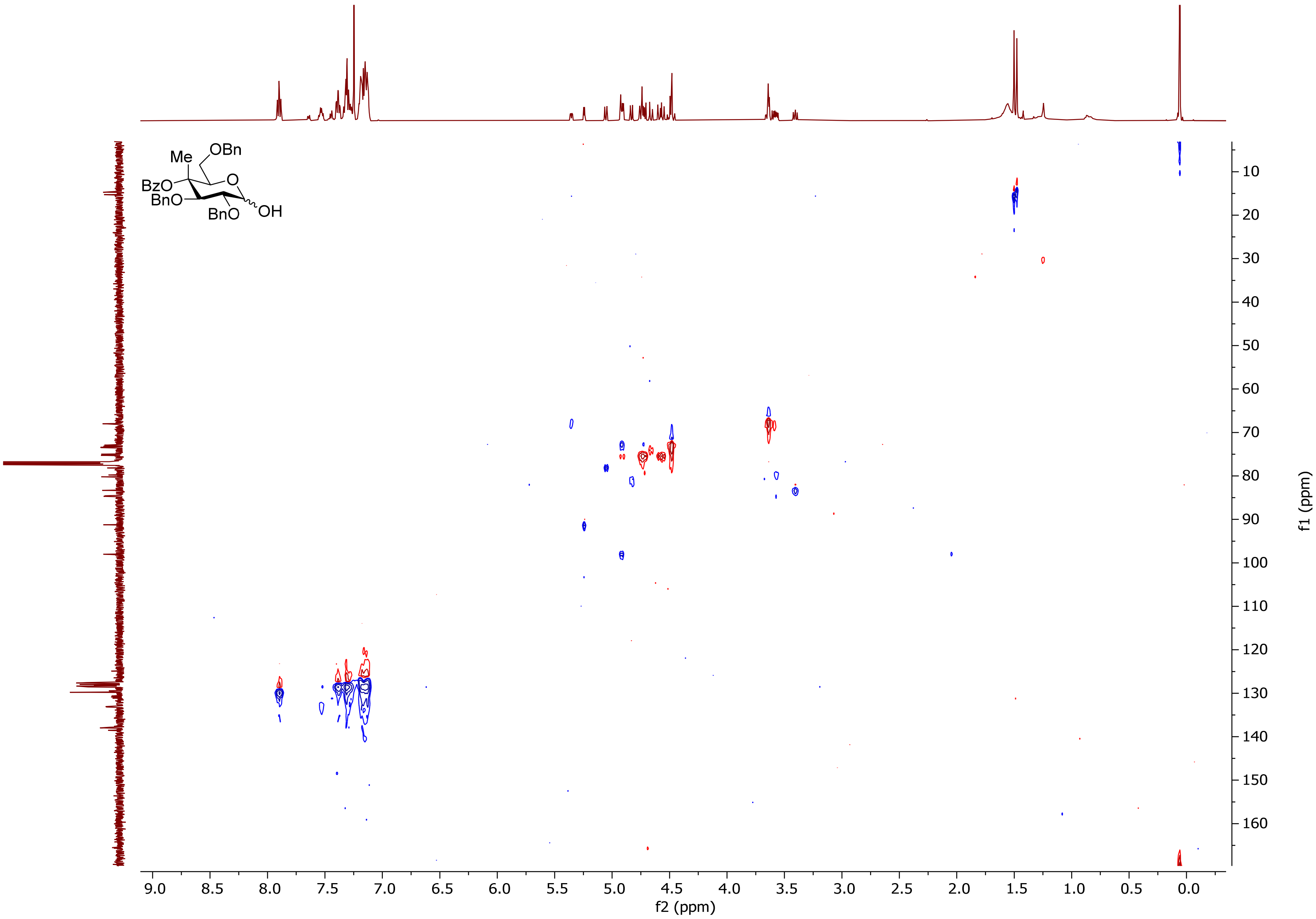

^{1}H NMR (500 MHz, CDCl$_3$) spectrum of 1,2:3,4-di-*O*-isopropylidene-6-*O*-trifluoromethanesulfonyl-*α*-D-galactopyranose (40)

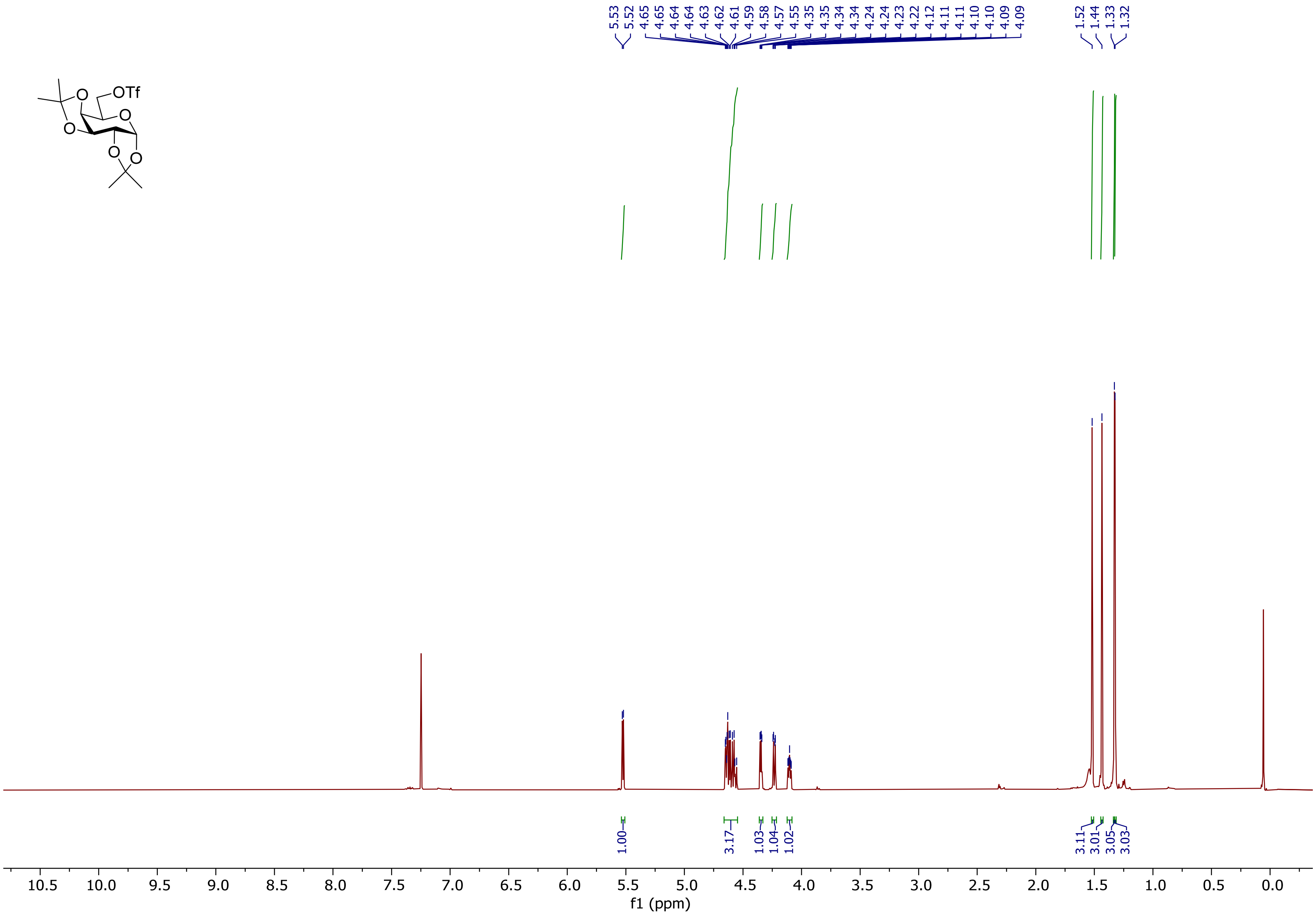

$^{13}C\{^{1}H\}$ NMR (126 MHz, $CDCl_3$) spectrum of 1,2:3,4-di-*O*-isopropylidene-6-*O*-trifluoromethanesulfonyl-α-D-galactopyranose (**40**)

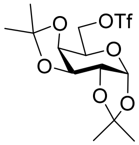

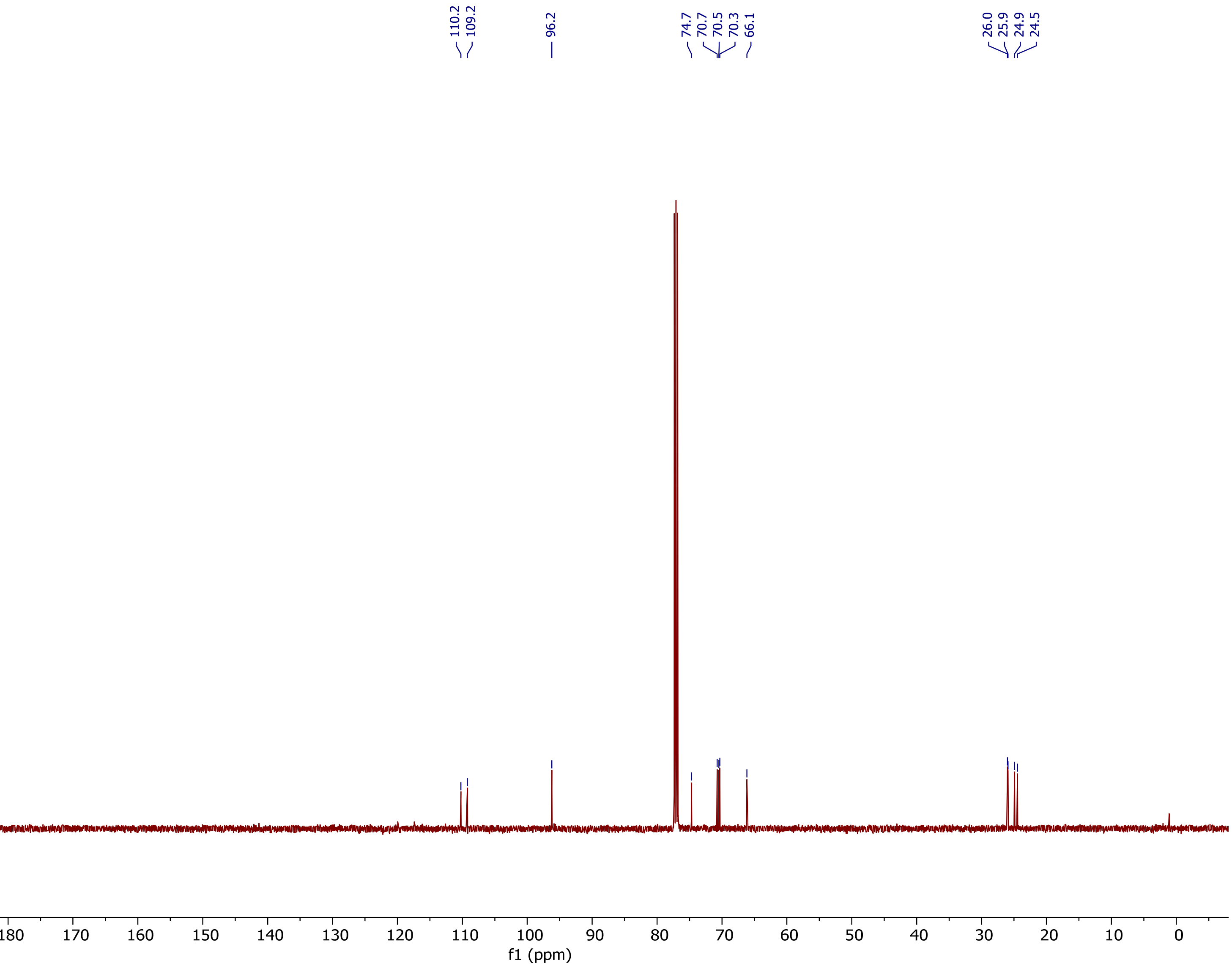

COSY NMR (500 MHz, $CDCl_3$) spectrum of 1,2:3,4-di-*O*-isopropylidene-6-*O*-trifluoromethanesulfonyl-*α*-D-galactopyranose (40)

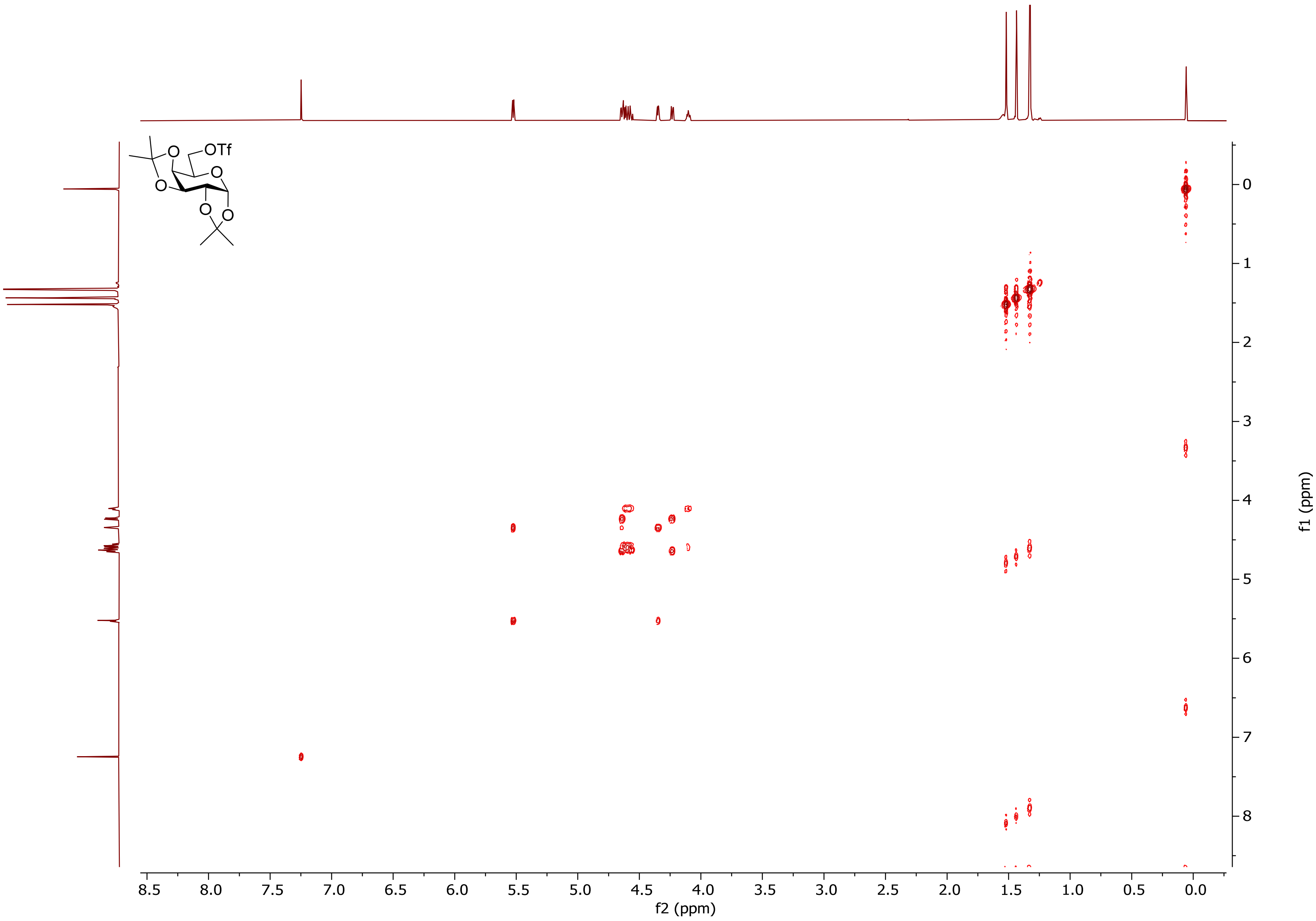

HSQC NMR (500 MHz, $CDCl_3$) spectrum of 1,2:3,4-di-*O*-isopropylidene-6-*O*-trifluoromethanesulfonyl-*α*-D-galactopyranose (40)

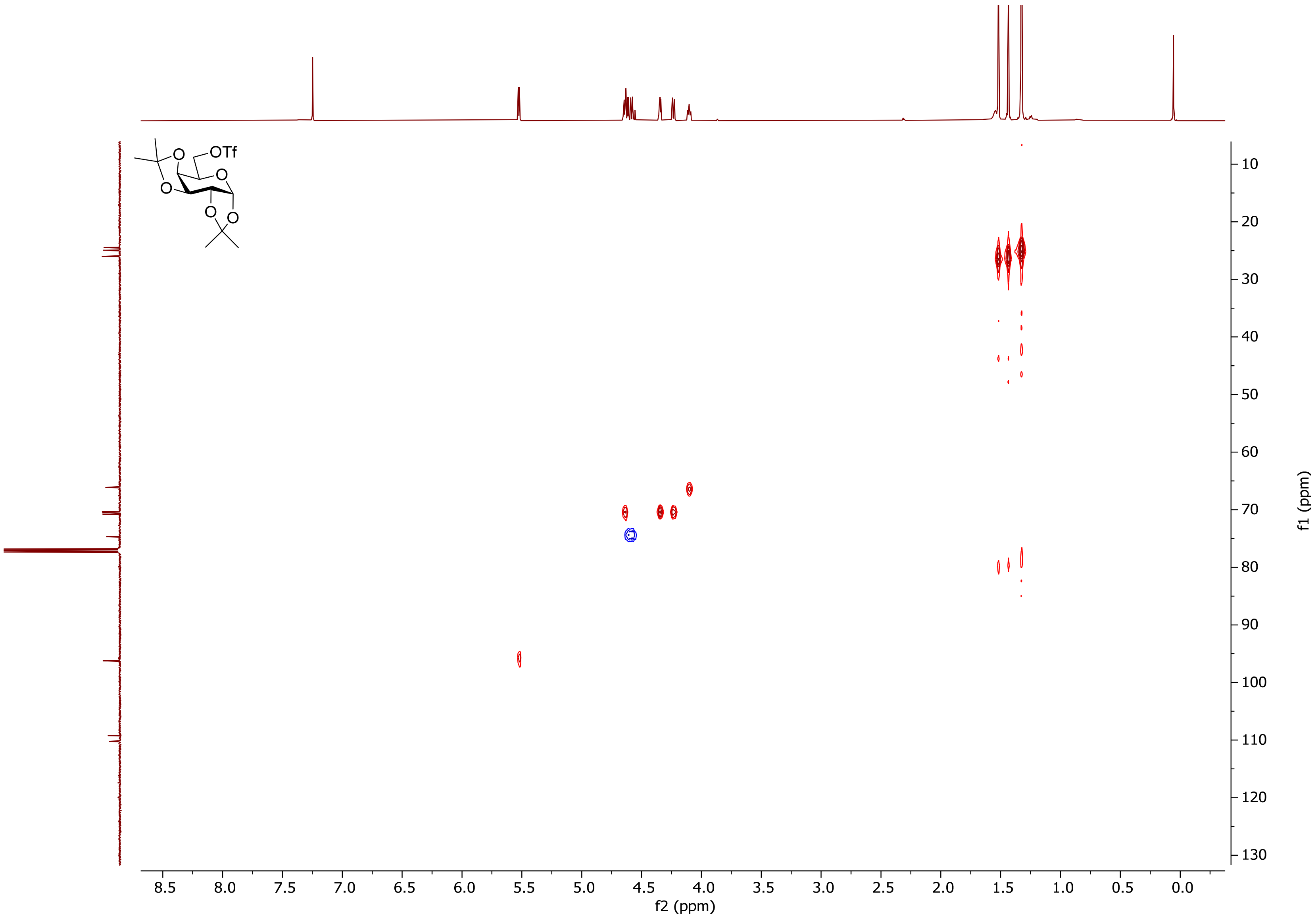

^{19}F NMR (470 MHz, $CDCl_3$) spectrum of 1,2:3,4-di-*O*-isopropylidene-6-*O*-trifluoromethanesulfonyl-α-D-galactopyranose (40)

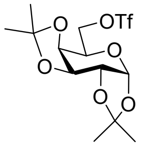

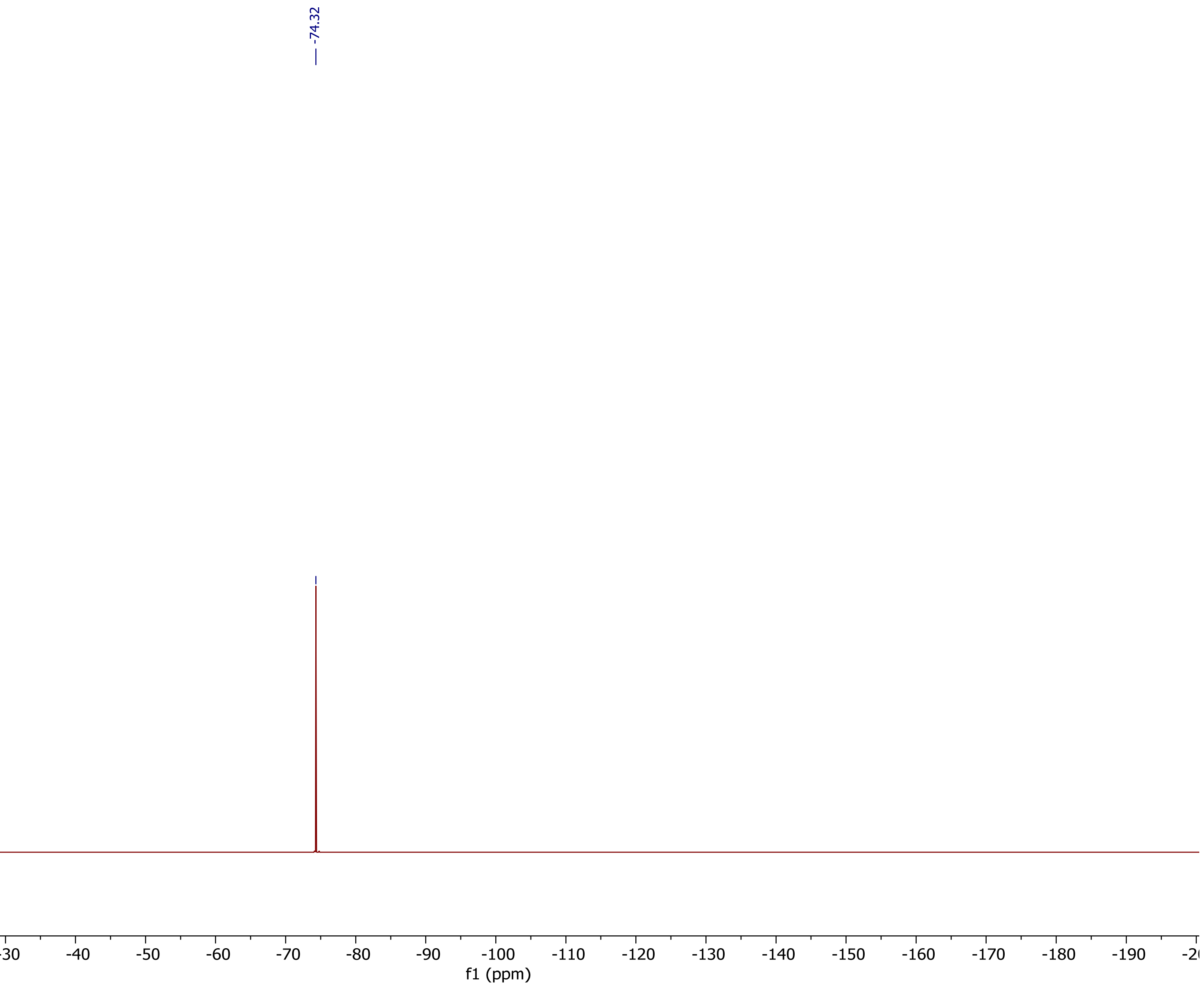

^{1}H NMR (500 MHz, $CDCl_3$) spectrum of 6-*O*-(4-*O*-benzoyl-2,3,6-tri-*O*-benzyl-*α*-D-galactopyranosyl)-1,2:3,4-di-*O*-isopropylidene-*α*-D-galactopyranose (41α)

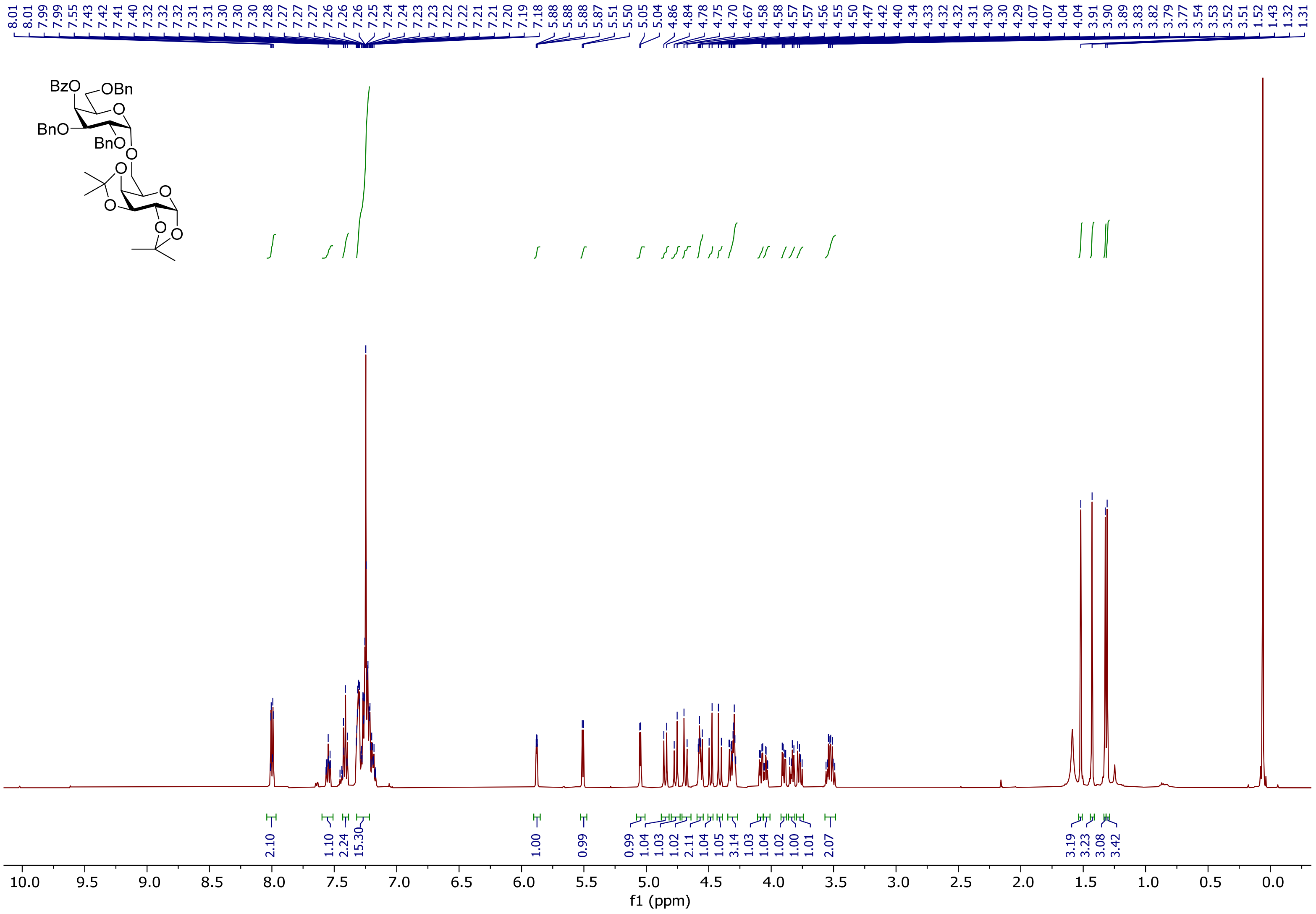

$^{13}C\{^{1}H\}$ NMR (126 MHz, $CDCl_3$) spectrum of 6-*O*-(4-*O*-benzoyl-2,3,6-tri-*O*-benzyl-*α*-D-galactopyranosyl)-1,2:3,4-di-*O*-isopropylidene-*α*-D-galactopyranose (41α)

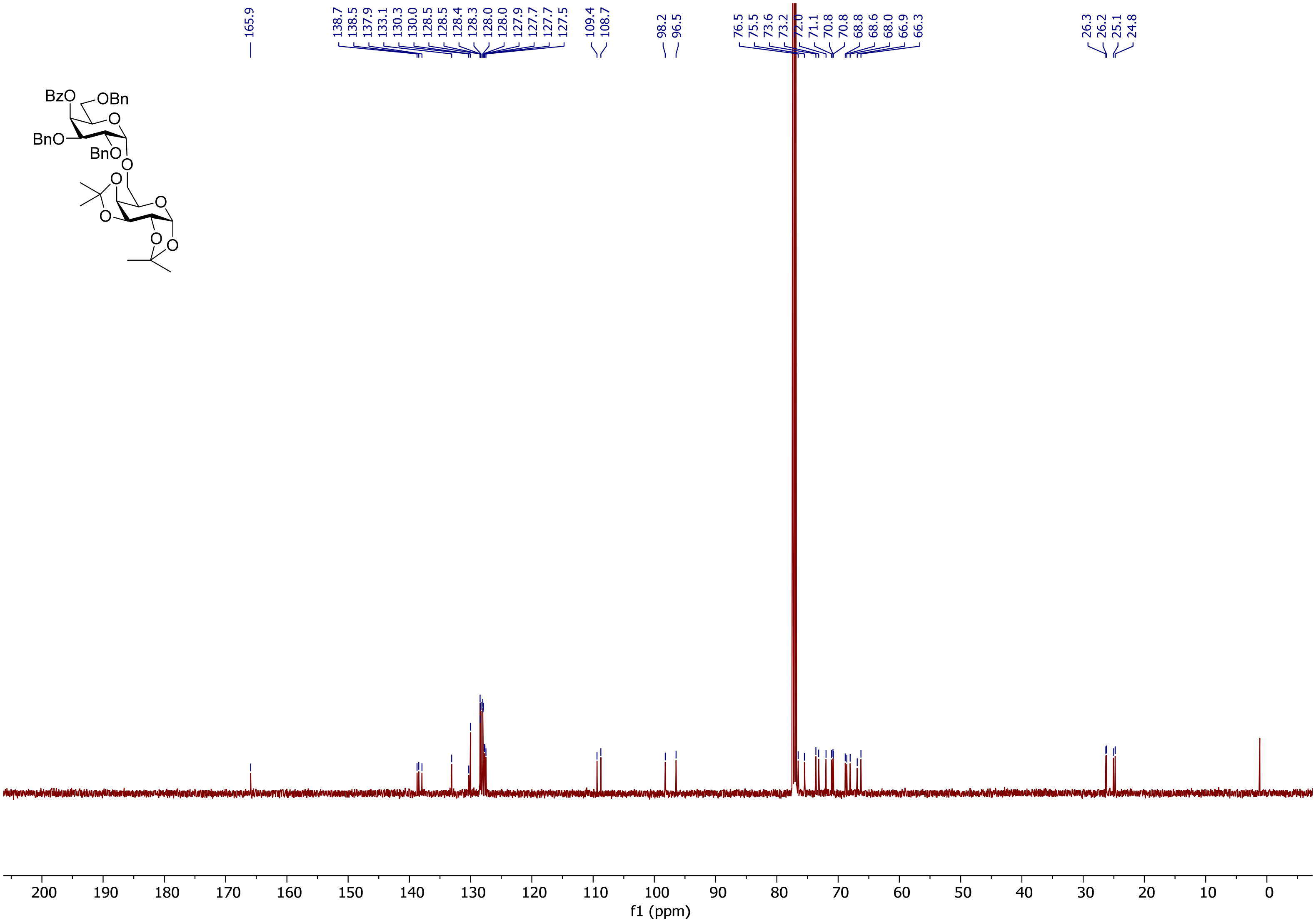

COSY NMR (500 MHz, CDCl$_3$) spectrum of 6-*O*-(4-*O*-benzoyl-2,3,6-tri-*O*-benzyl-*α*-D-galactopyranosyl)-1,2:3,4-di-*O*-isopropylidene-*α*-D-galactopyranose (41α)

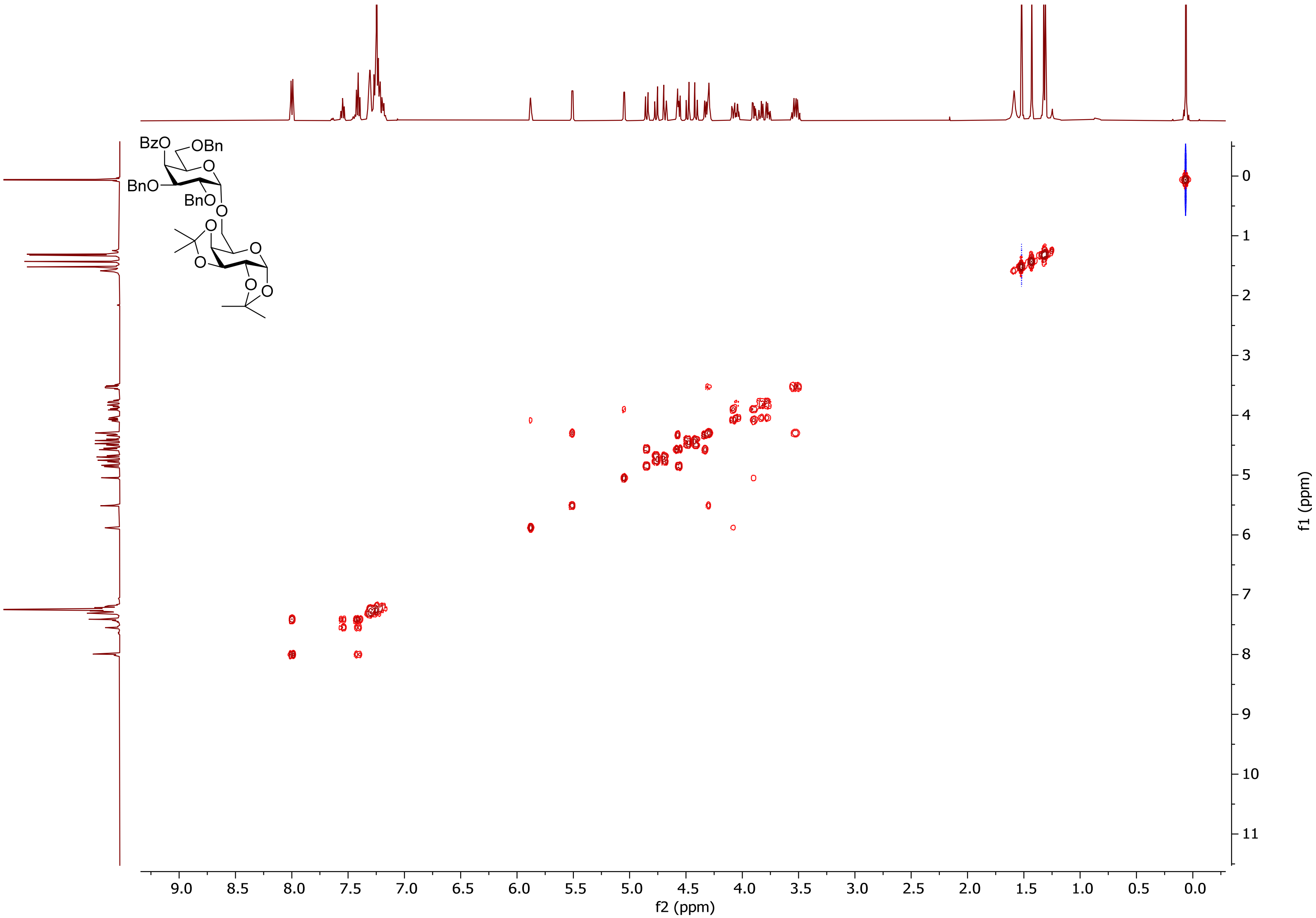

HSQC NMR (500 MHz, CDCl$_3$) spectrum of 6-*O*-(4-*O*-benzoyl-2,3,6-tri-*O*-benzyl-*α*-D-galactopyranosyl)-1,2:3,4-di-*O*-isopropylidene-*α*-D-galactopyranose (41α)

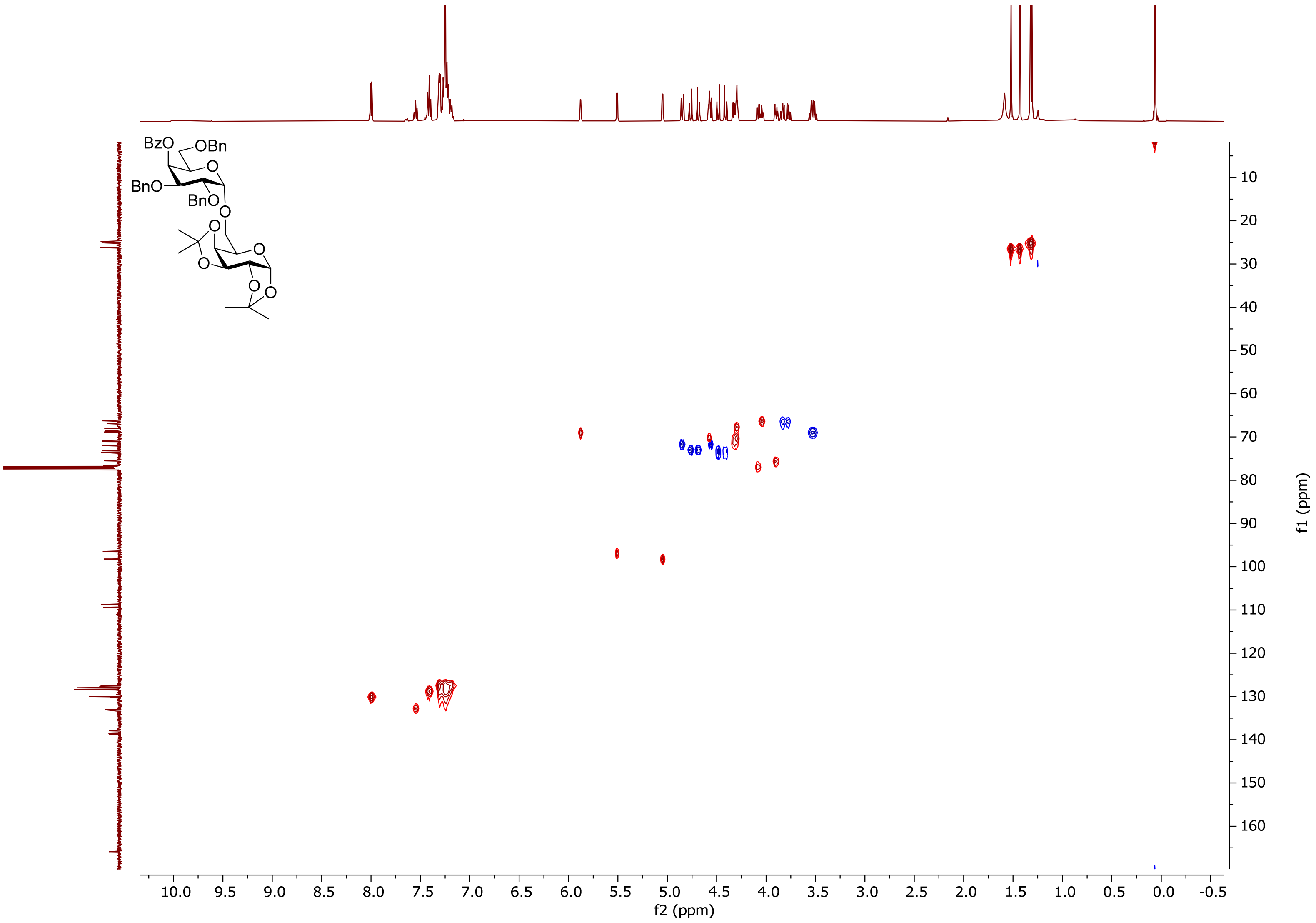

^{1}H NMR (500 MHz, CDCl$_3$) spectrum of 6-*O*-(4-*O*-benzoyl-2,3,6-tri-*O*-benzyl-*β*-D-galactopyranosyl)-1,2:3,4-di-*O*-isopropylidene-*α*-D-galactopyranose (41β)

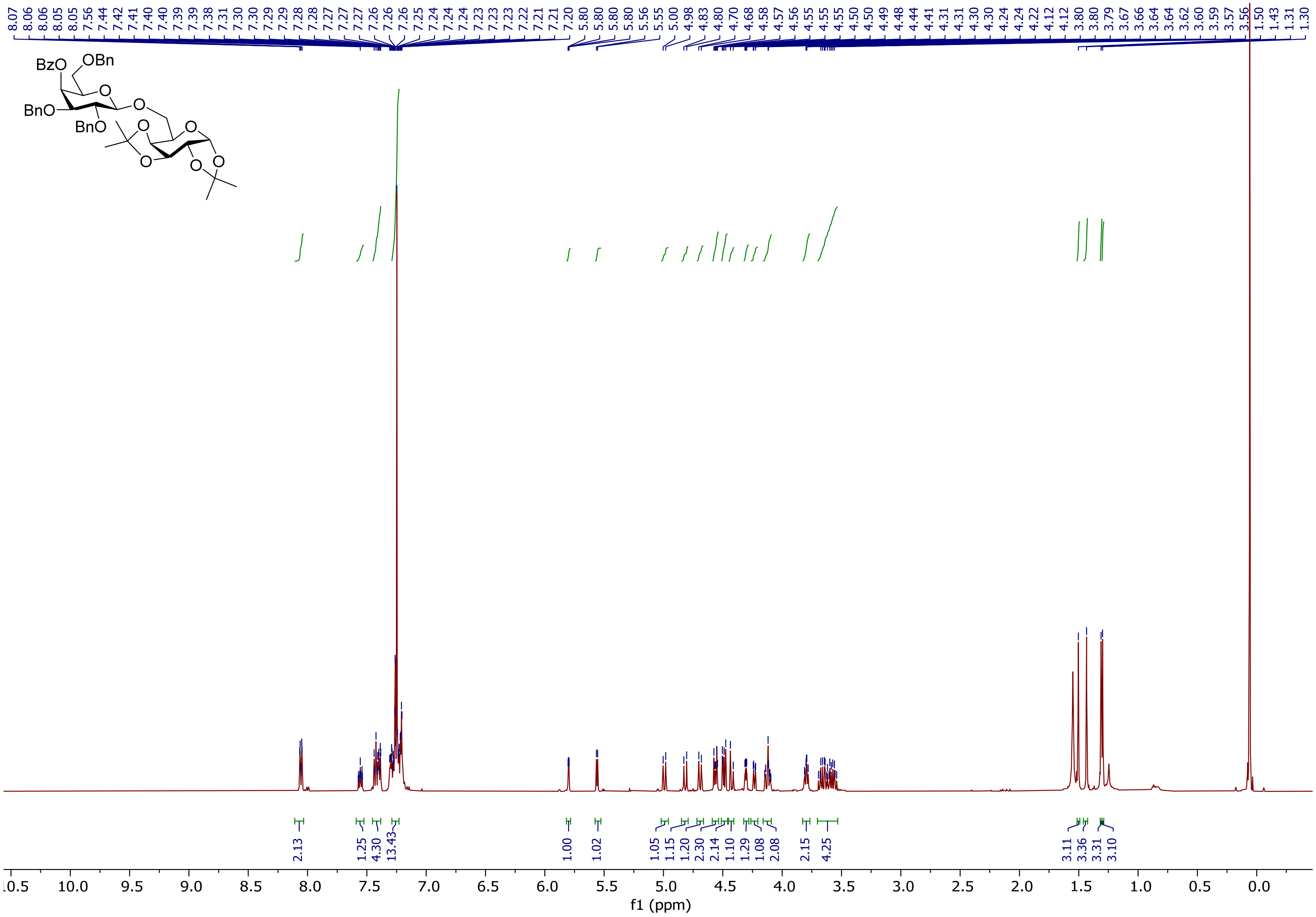

$^{13}C\{^{1}H\}$ NMR (126 MHz, $CDCl_3$) spectrum of 6-*O*-(4-*O*-benzoyl-2,3,6-tri-*O*-benzyl-*β*-D-galactopyranosyl)-1,2:3,4-di-*O*-isopropylidene-*α*-D-galactopyranose (41β)

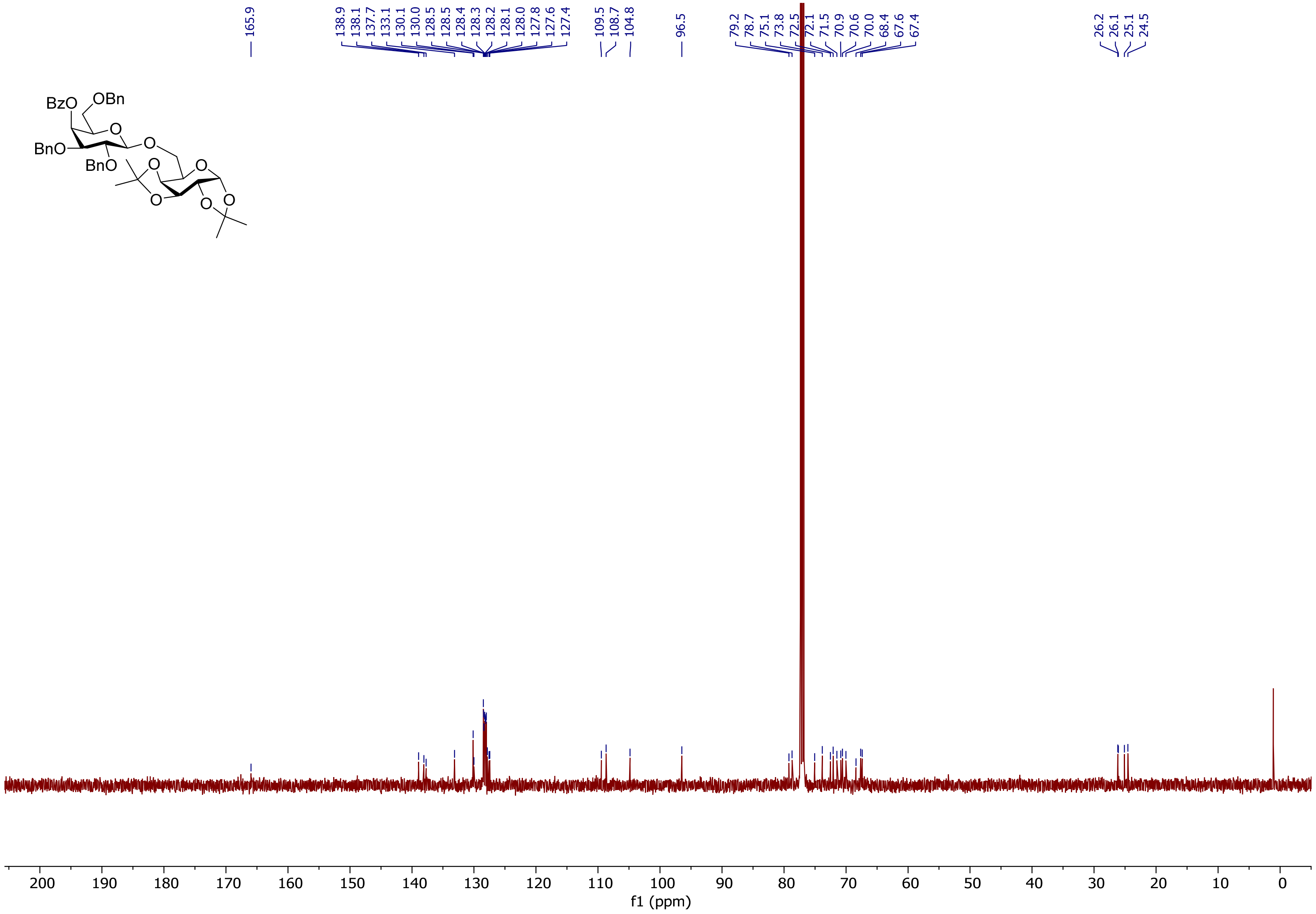

COSY NMR (500 MHz, $CDCl_3$) spectrum of 6-*O*-(4-*O*-benzoyl-2,3,6-tri-*O*-benzyl-*β*-D-galactopyranosyl)-1,2:3,4-di-*O*-isopropylidene-*α*-D-galactopyranose (41β)

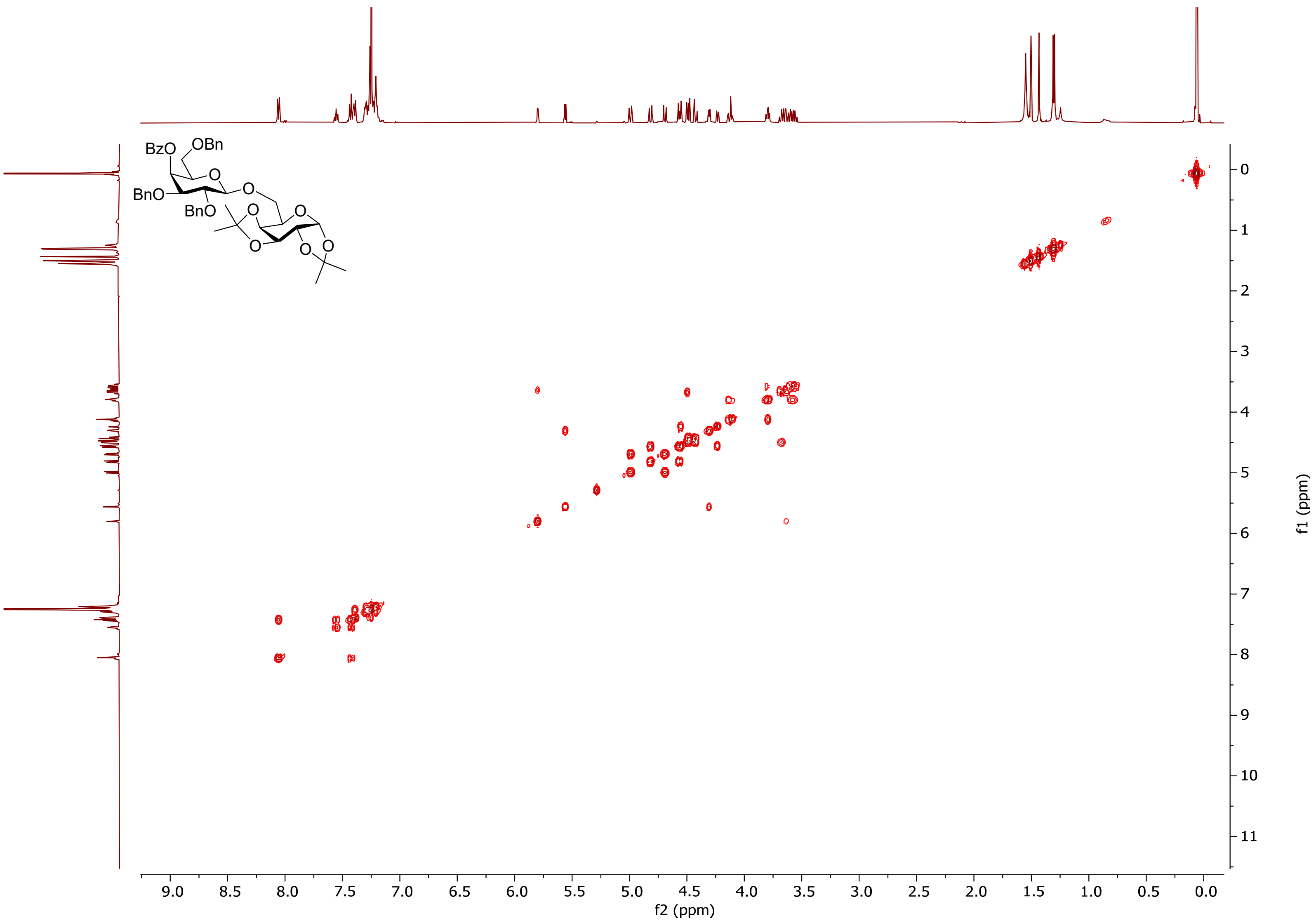

HSQC NMR (500 MHz, CDCl$_3$) spectrum of 6-*O*-(4-*O*-benzoyl-2,3,6-tri-*O*-benzyl-*β*-D-galactopyranosyl)-1,2:3,4-di-*O*-isopropylidene-*α*-D-galactopyranose (41β)

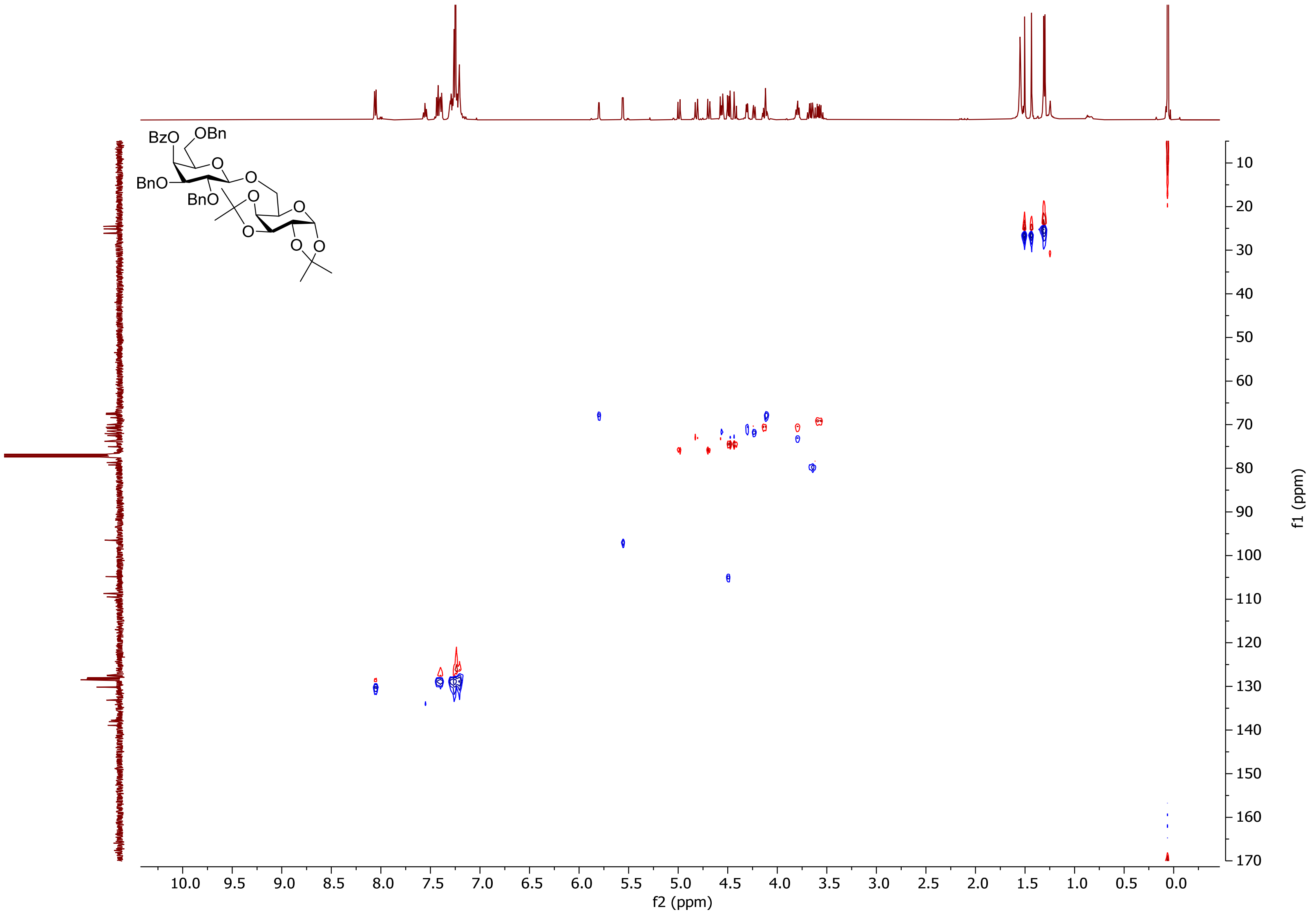

^{1}H NMR (500 MHz, CDCl$_3$) spectrum of *p*-methylphenyl 4-*O*-benzoyl-2,3,6-tri-*O*-benzyl-4-*C*-methyl-1-thio-*β*-D-galactopyranoside-^{13}*C* (3-^{13}*C*)

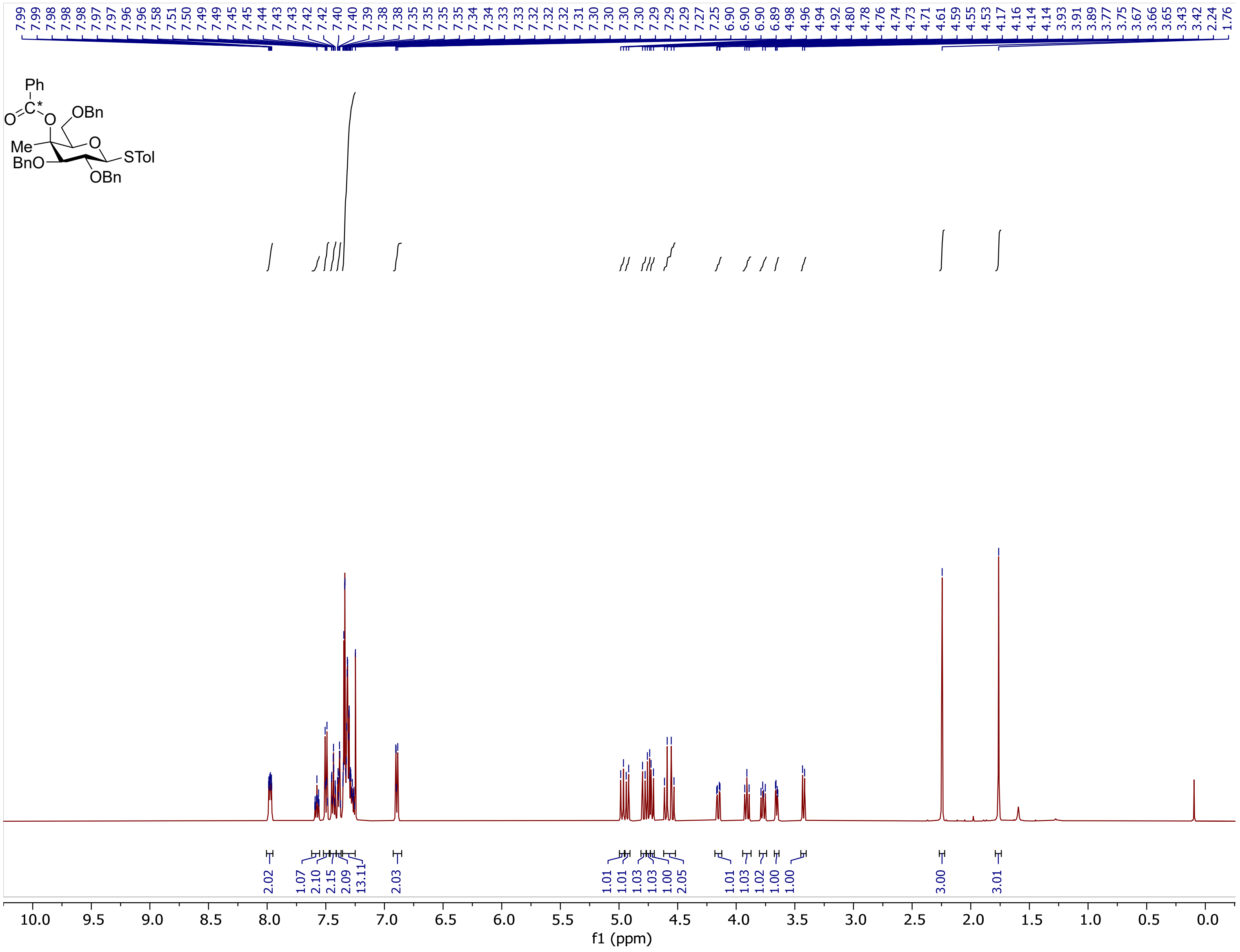

$^{13}C\{^1H\}$ NMR (126 MHz, $CDCl_3$) spectrum of *p*-methylphenyl 4-*O*-benzoyl-2,3,6-tri-*O*-benzyl-4-*C*-methyl-1-thio-*β*-D-galactopyranoside-*^{13}C* (3-*^{13}C*)

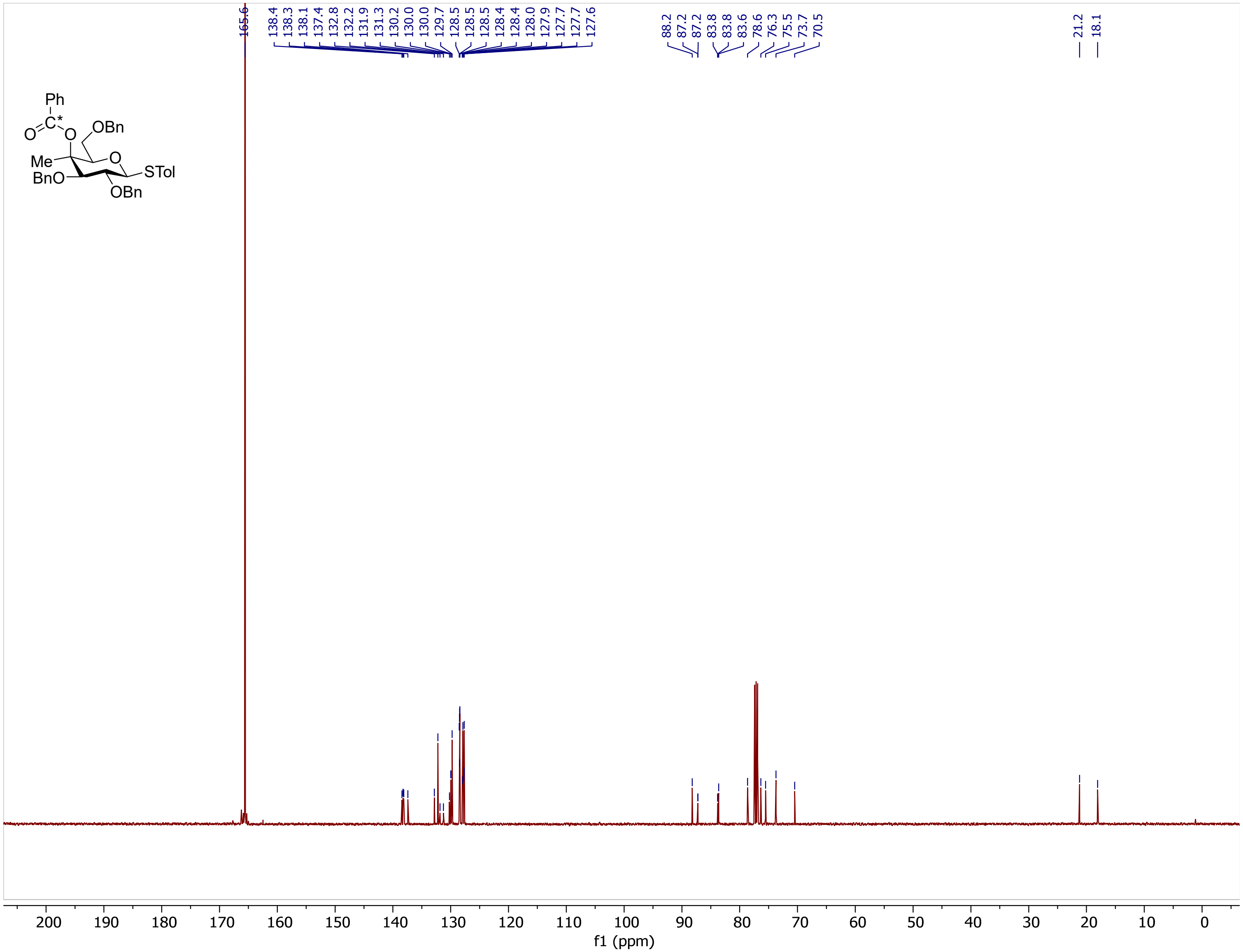

COSY NMR (500 MHz, $CDCl_3$) spectrum of *p*-methylphenyl 4-*O*-benzoyl-2,3,6-tri-*O*-benzyl-4-*C*-methyl-1-thio-*β*-D-galactopyranoside-^{13}C (3-^{13}C)

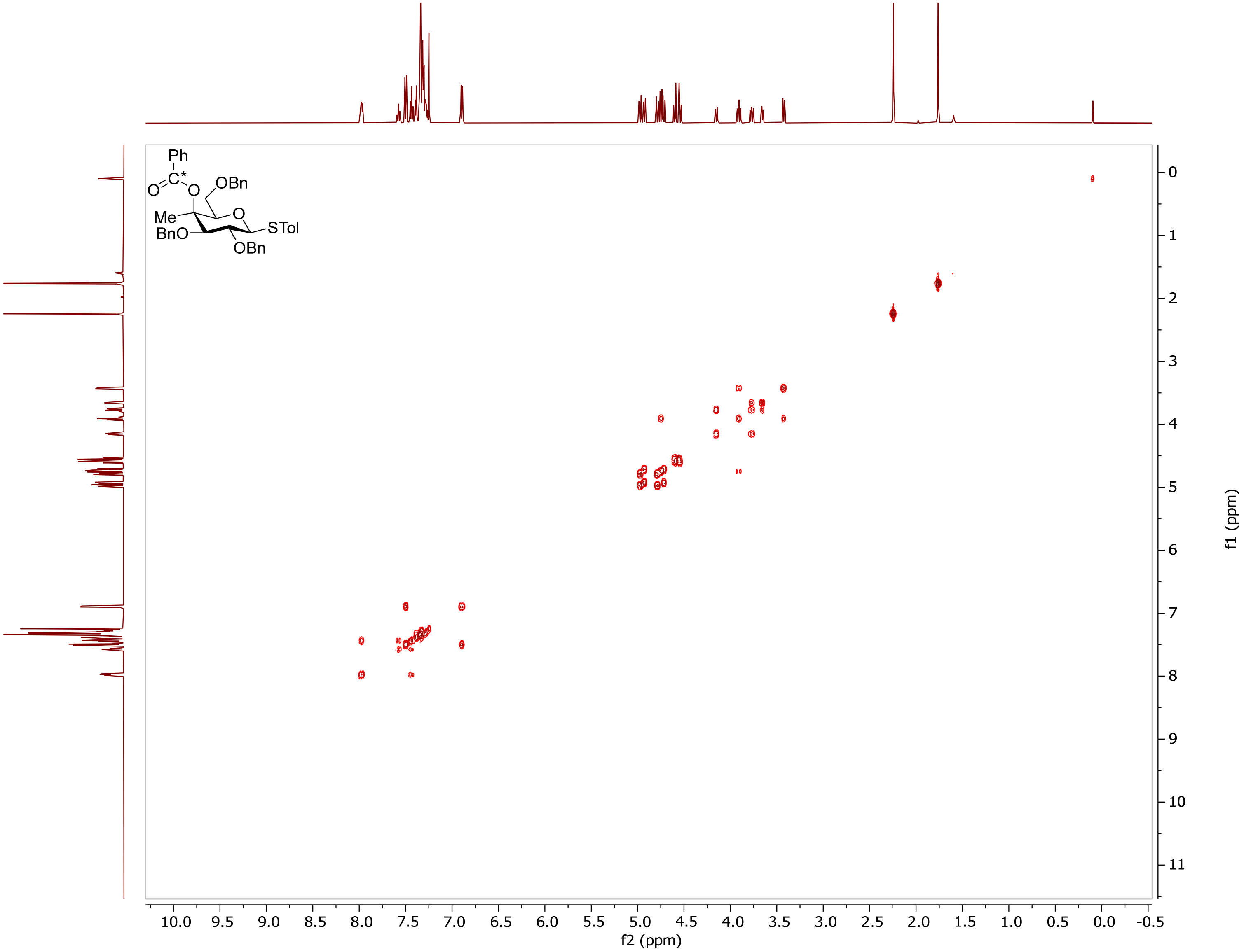

HSQC NMR (500 MHz, $CDCl_3$) spectrum of *p*-methylphenyl 4-*O*-benzoyl-2,3,6-tri-*O*-benzyl-4-*C*-methyl-1-thio-*β*-D-galactopyranoside-^{13}C (3-^{13}C)

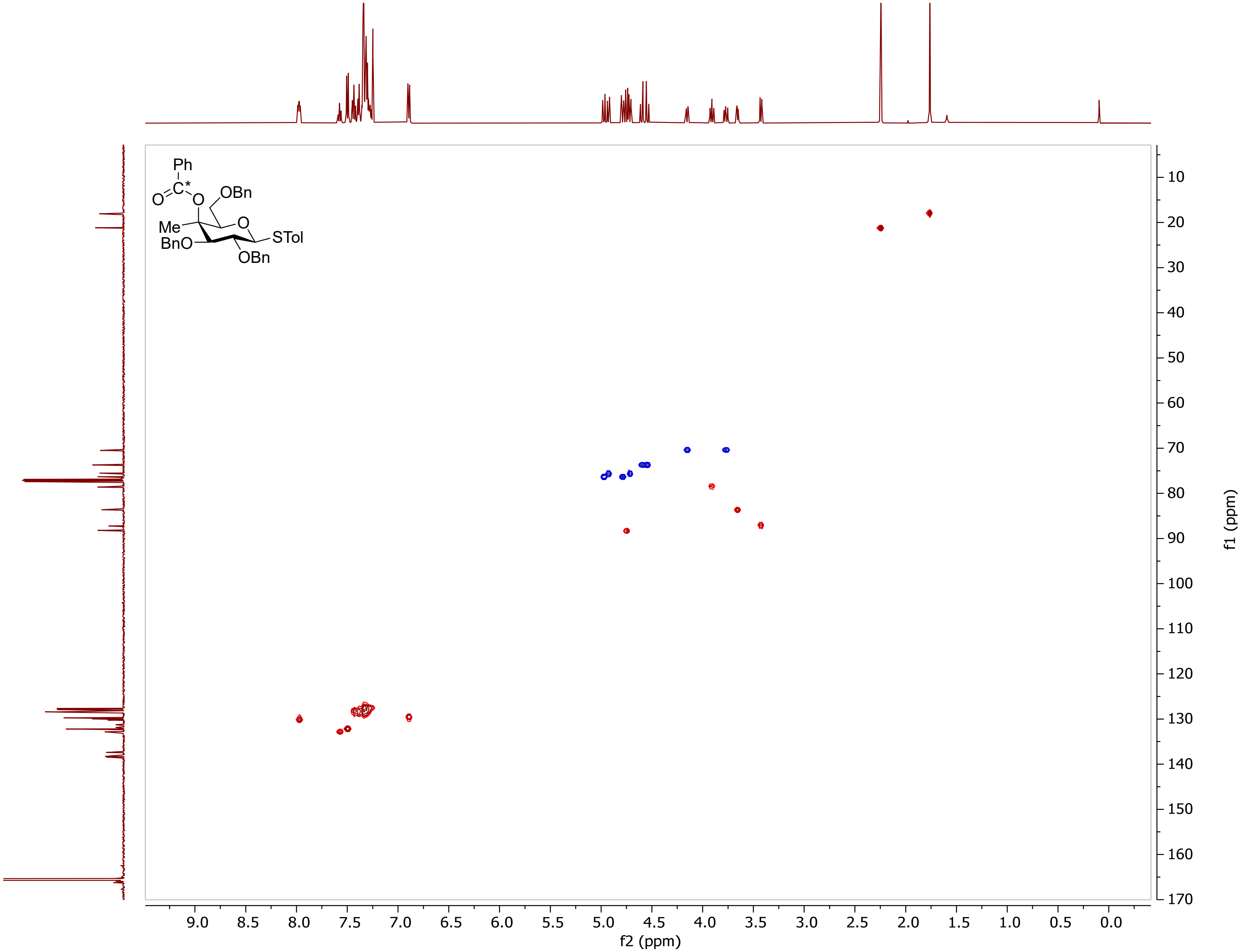

^{1}H NMR (500 MHz, CD_2Cl_2) spectrum of *p*-methylphenyl 4-*O*-benzoyl-2,3,6-tri-*O*-benzyl-4-*C*-methyl-1-thio-*β*-D-glucopyranoside-*^{13}C* (44)

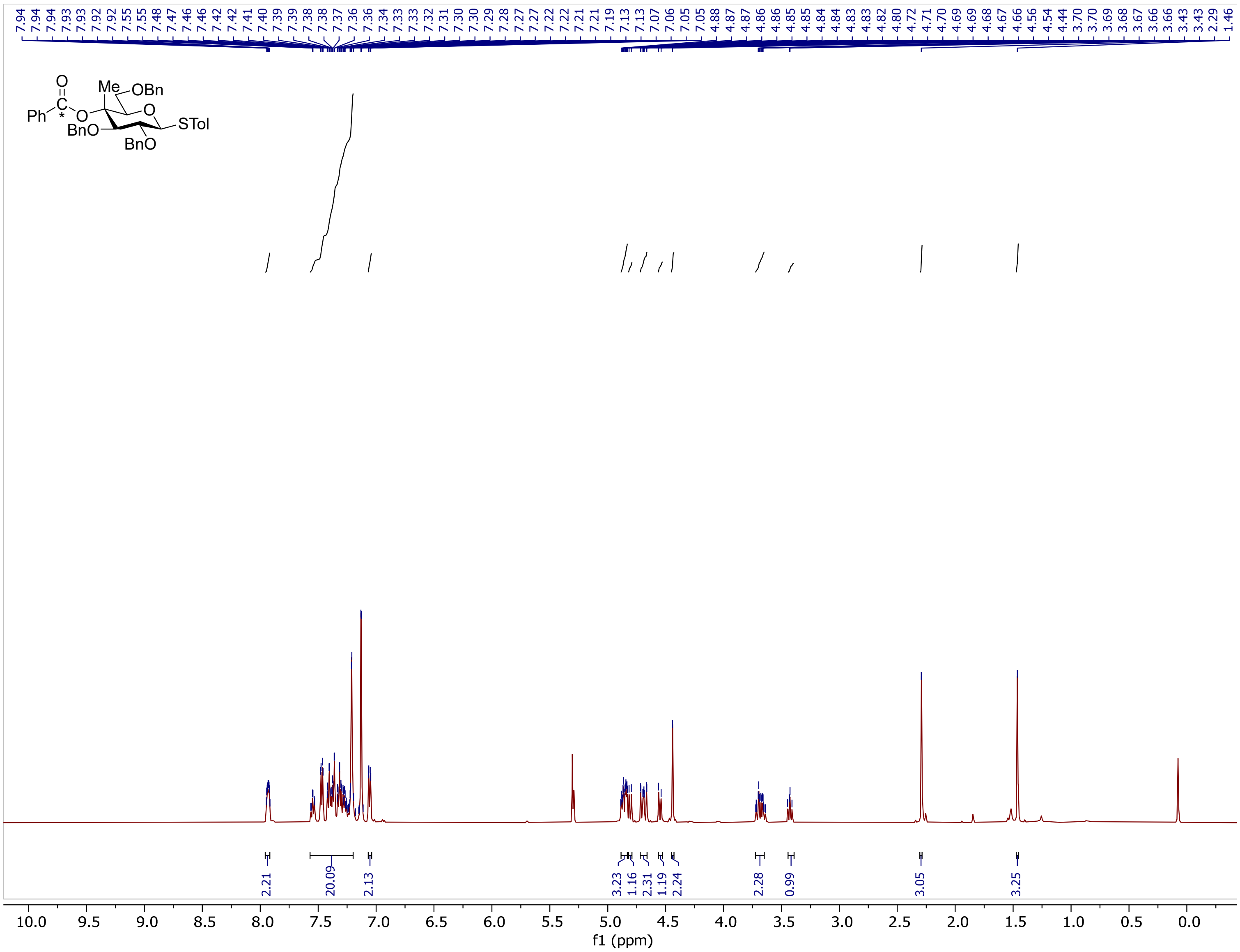

$^{13}C\{^{1}H\}$ NMR (500 MHz, CD_2Cl_2) spectrum of *p*-methylphenyl 4-*O*-benzoyl-2,3,6-tri-*O*-benzyl-4-*C*-methyl-1-thio-*β*-D-glucopyranoside-^{13}*C* (44)

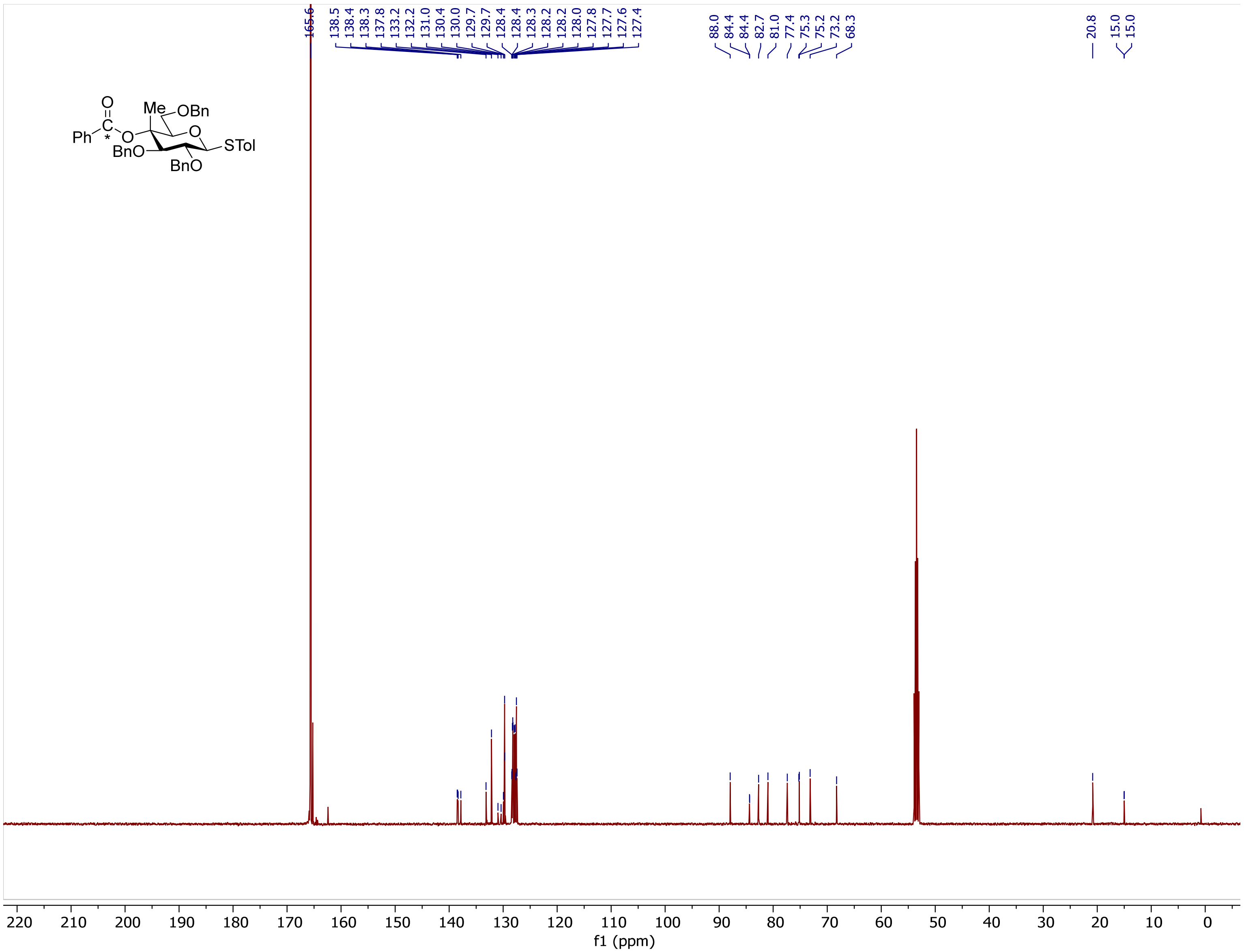

COSY NMR (500 MHz, CD_2Cl_2) spectrum of *p*-methylphenyl 4-*O*-benzoyl-2,3,6-tri-*O*-benzyl-4-*C*-methyl-1-thio-*β*-D-glucopyranoside-^{13}C (44)

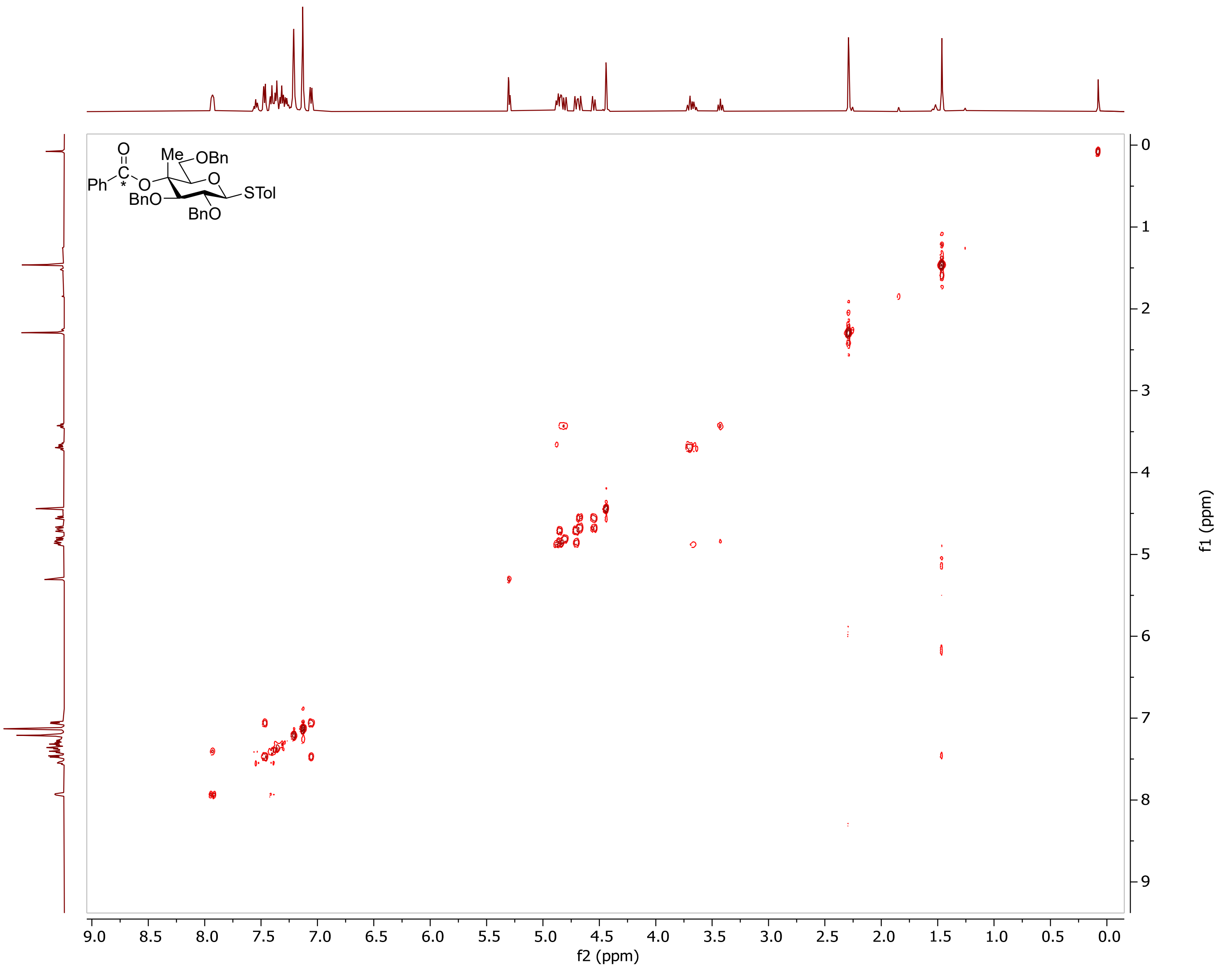

HSQC NMR (500 MHz, CD_2Cl_2) spectrum of *p*-methylphenyl 4-*O*-benzoyl-2,3,6-tri-*O*-benzyl-4-*C*-methyl-1-thio-*β*-D-glucopyranoside-^{13}C (44)

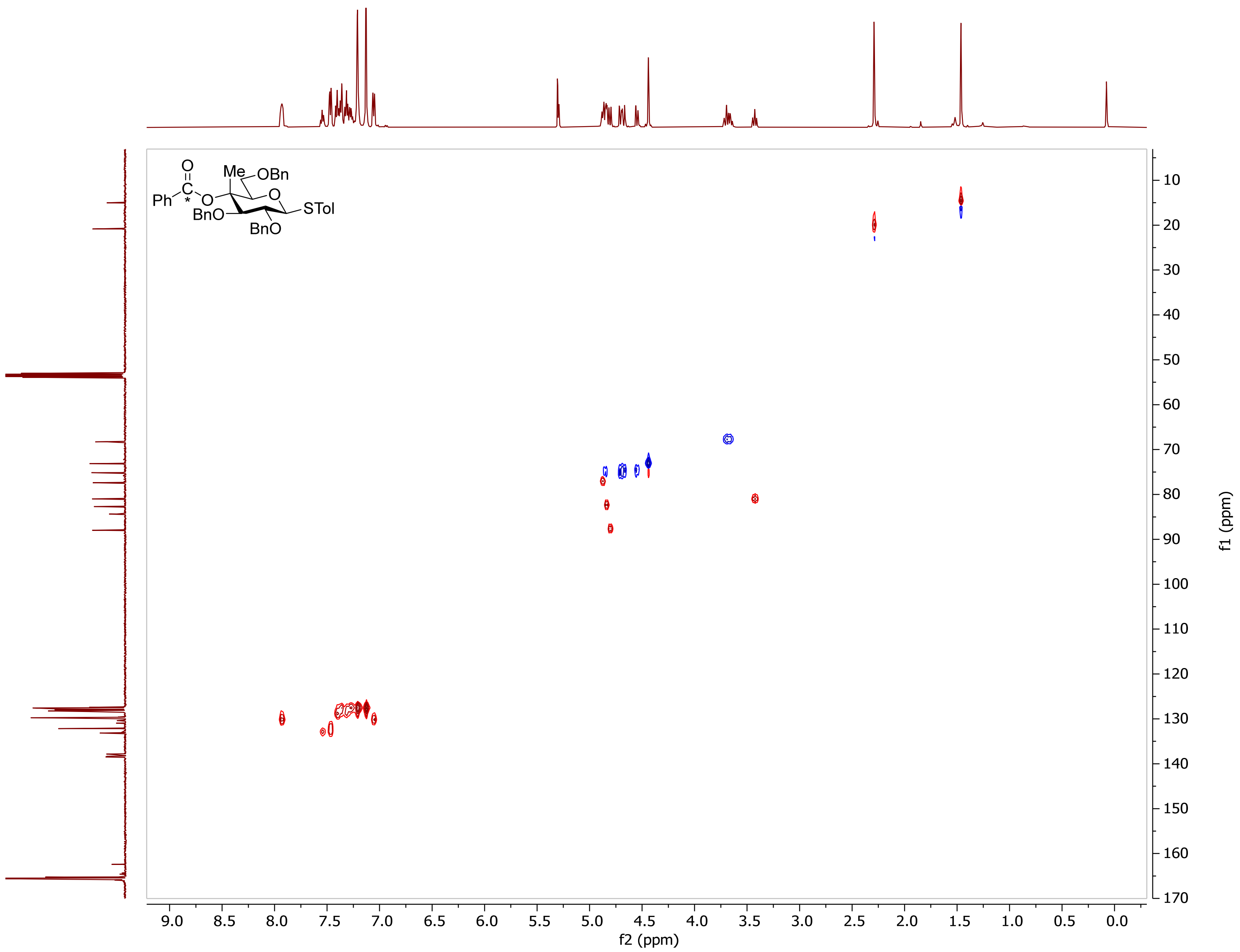

^{1}H NMR (500 MHz, CD_2Cl_2) spectrum of *p*-methylphenyl 4-*O*-benzoyl-2,3,6-tri-*O*-benzyl-4-*C*-methyl-1-thio-*β*-D-glucopyranosylsulfoxide-*^{13}C* (45)

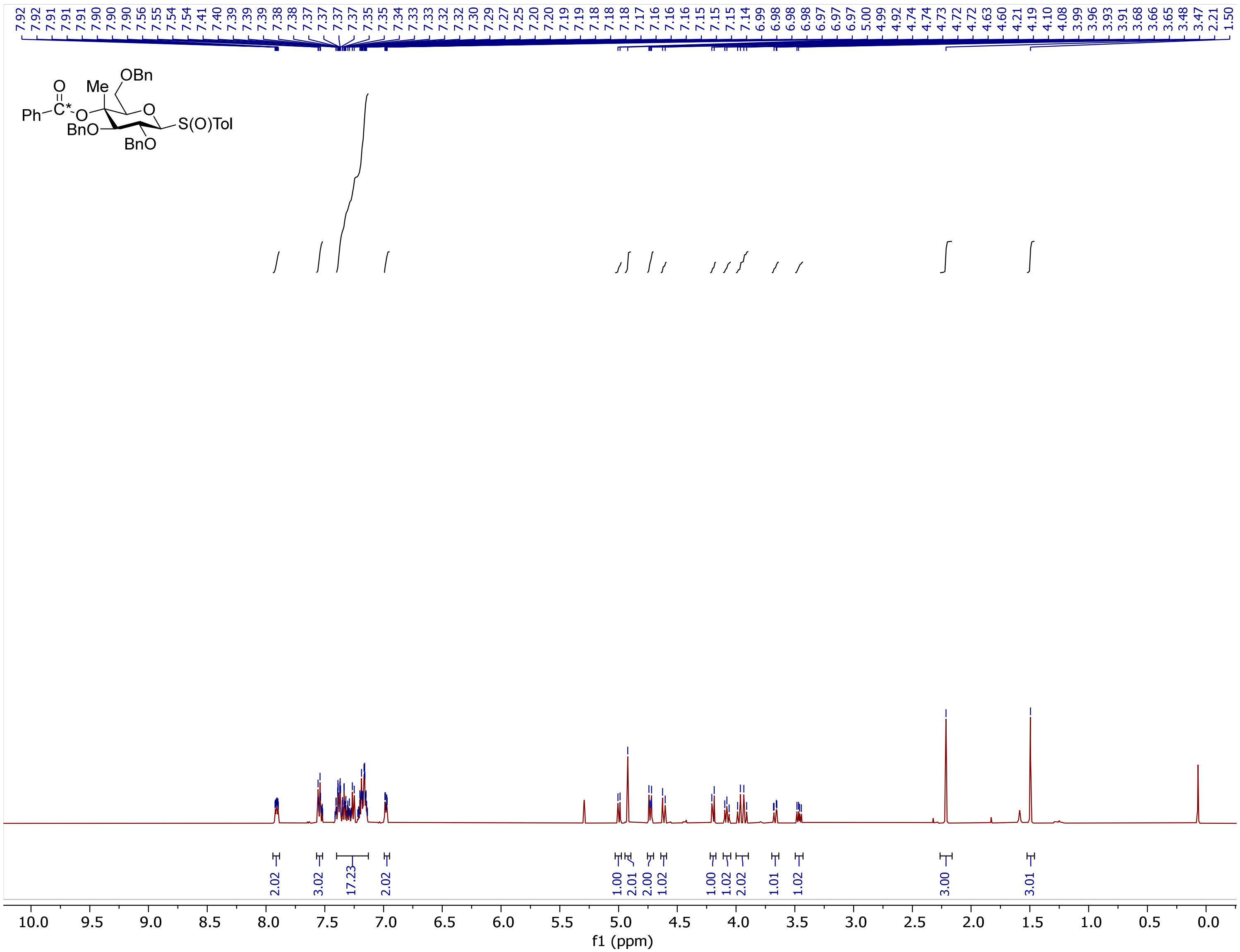

$^{13}C\{^{1}H\}$ NMR (126 MHz, CD_2Cl_2) spectrum of *p*-methylphenyl 4-*O*-benzoyl-2,3,6-tri-*O*-benzyl-4-*C*-methyl-1-thio-*β*-D-glucopyranosylsulfoxide-*^{13}C* (45)

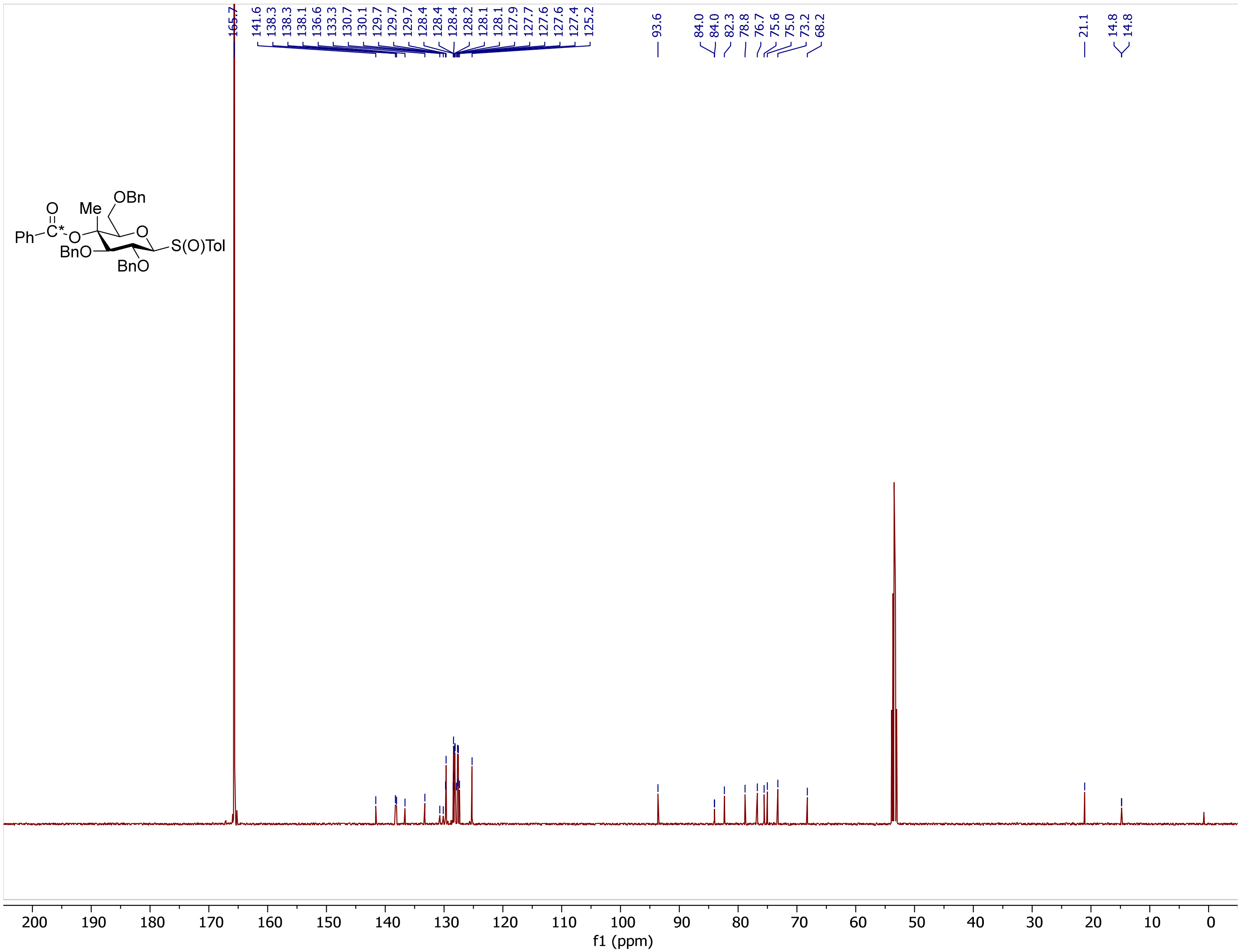

COSY NMR (500 MHz, CD_2Cl_2) spectrum of *p*-methylphenyl 4-*O*-benzoyl-2,3,6-tri-*O*-benzyl-4-*C*-methyl-1-thio-*β*-D-glucopyranosylsulfoxide-^{13}C (45)

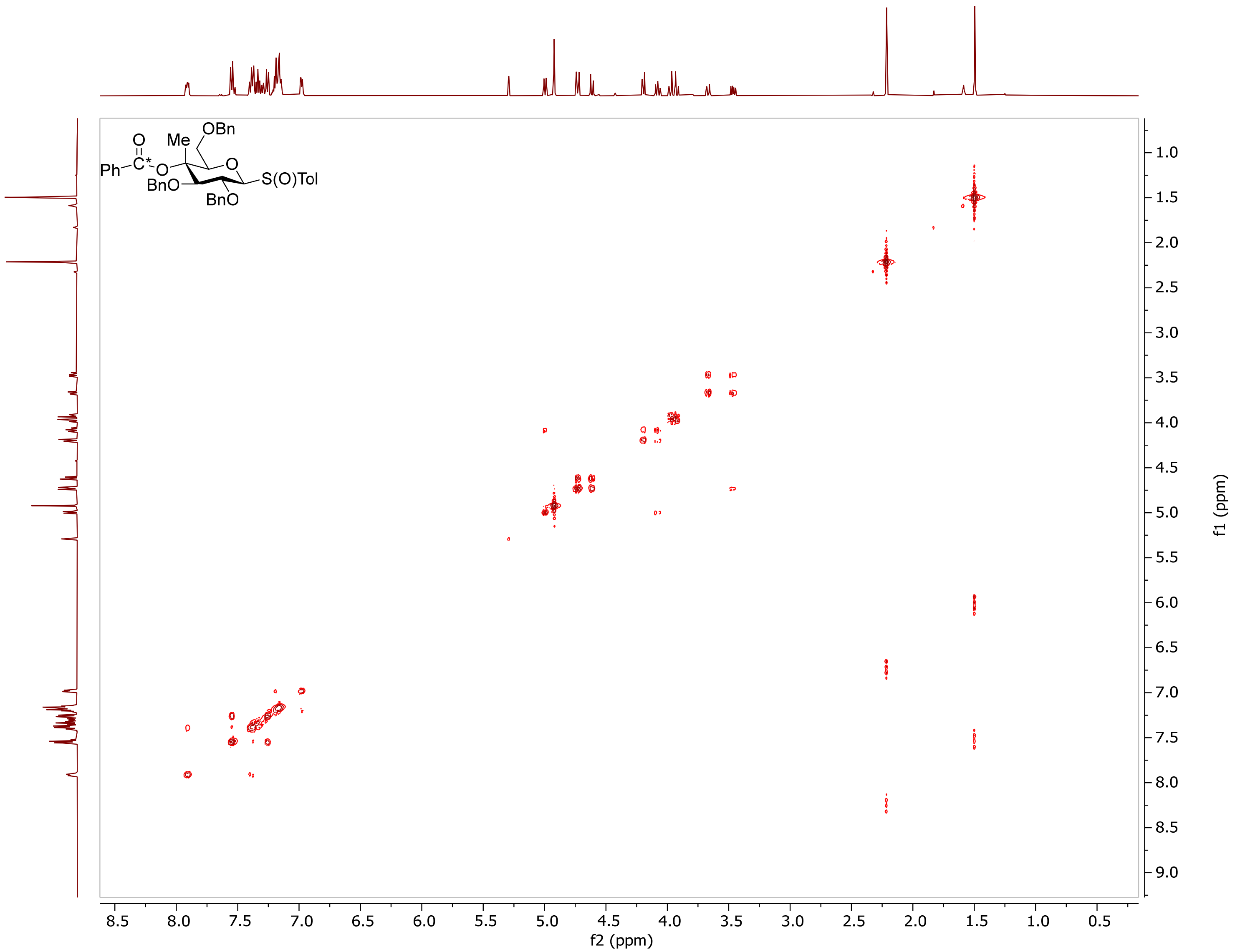

HSQC NMR (500 MHz, CD_2Cl_2) spectrum of *p*-methylphenyl 4-*O*-benzoyl-2,3,6-tri-*O*-benzyl-4-*C*-methyl-1-thio-*β*-D-glucopyranosylsulfoxide-^{13}C (45)

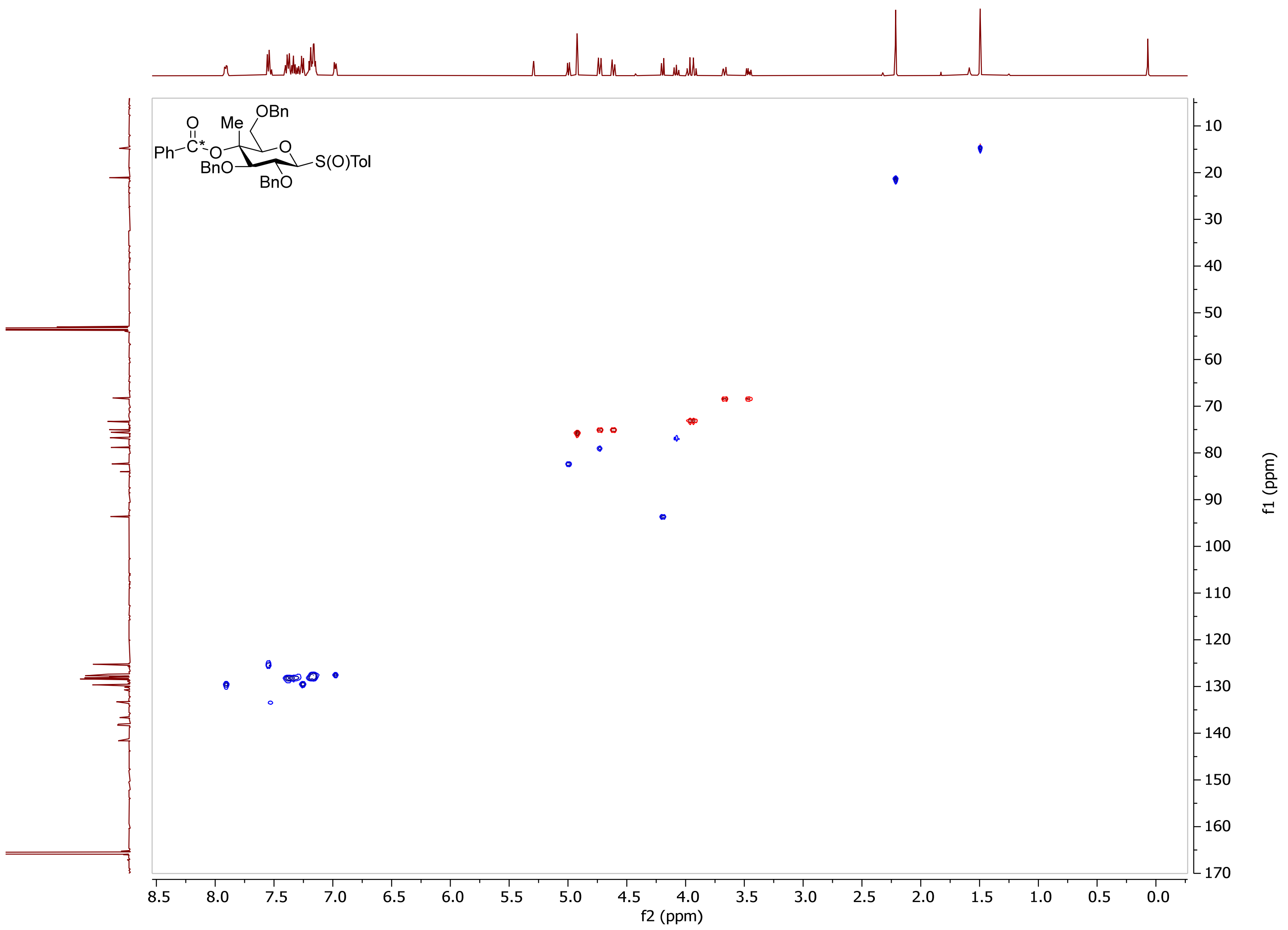

Variable temperature ^{1}H NMR (500 MHz, CD_2Cl_2) study of *p*-methylphenyl 4-*O*-benzoyl-2,3,6-tri-*O*-benzyl-4-*C*-methyl-1-thio-*β*-D-glucopyranosylsulfoxide-*^{13}C* (45)

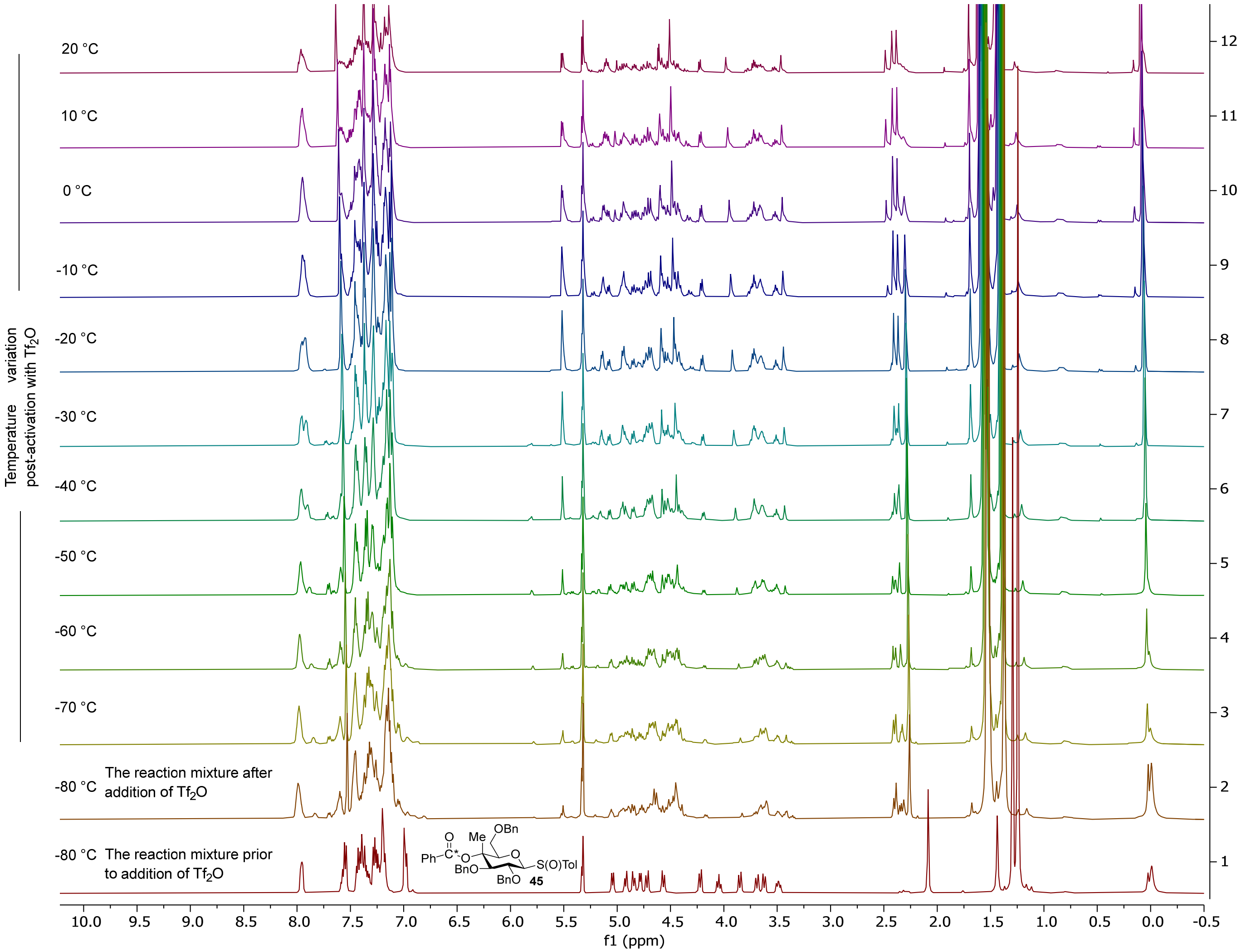

^{1}H NMR spectrum of sulfoxide **45**: after the addition of Tf_2O, temperature increased by 10 °C increments every 10 minutes and ^{1}H NMR spectra acquired at each temperature.

Variable temperature ^{13}C NMR (126 MHz, CD_2Cl_2) study of *p*-methylphenyl 4-*O*-benzoyl-2,3,6-tri-*O*-benzyl-4-*C*-methyl-1-thio-*β*-D-glucopyranosylsulfoxide-*^{13}C* (45)

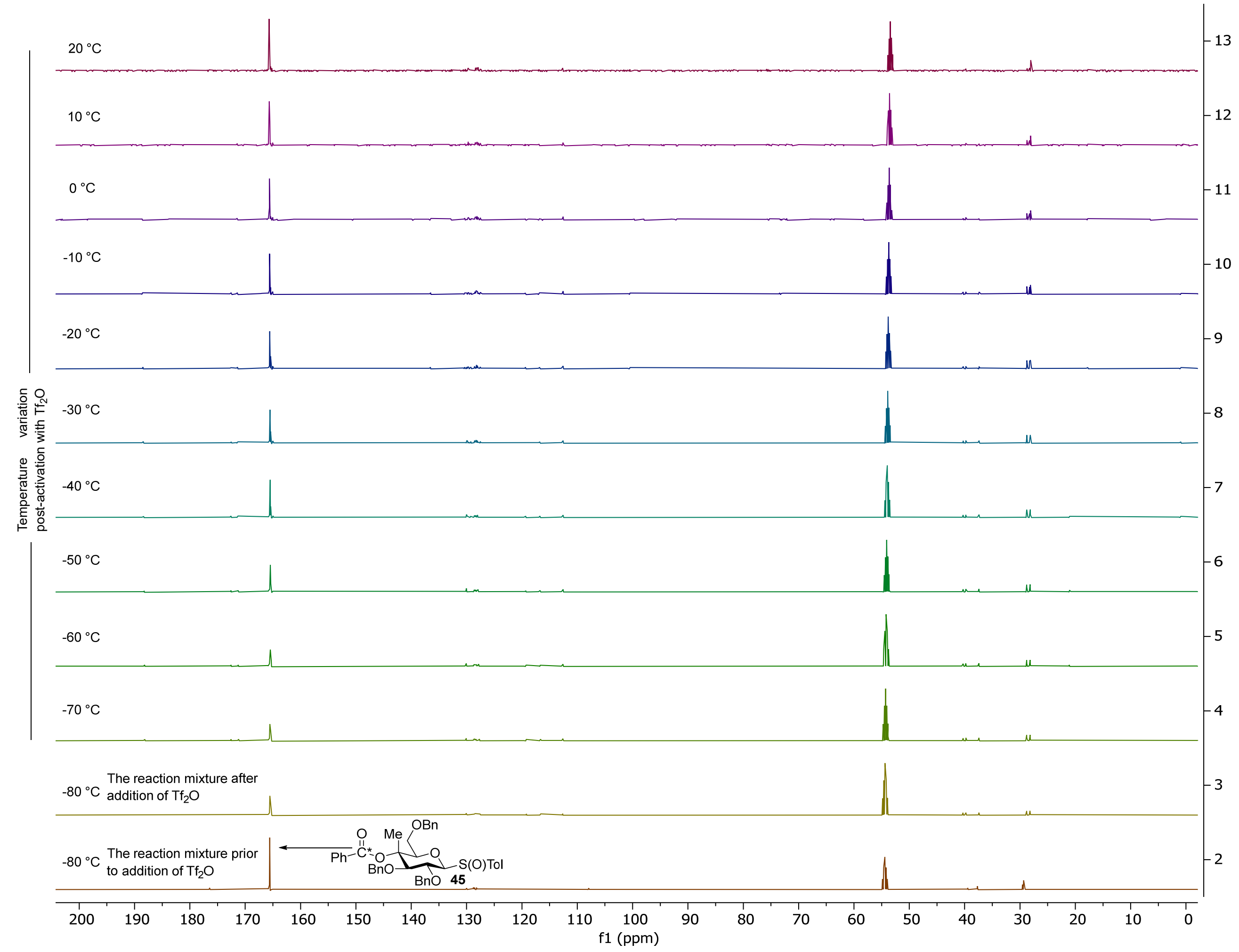

^{13}C NMR spectrum of sulfoxide **45**: after the addition of Tf_2O, temperature increased by 10 °C increments every 10 minutes and ^{13}C NMR spectra acquired at each temperature. ^{13}C NMR analysis revealed that the carbonyl carbon resonance of sulfoxide **45** remaining constant at $\delta \approx 166.0$-165.7 throughout the course of the experiment. Indicating no evidence for the distal group participation in gluco-sulfoxide **45**.

HSQC (500 MHz, CD_2Cl_2) spectrum of *p*-methylphenyl 4-*O*-benzoyl-2,3,6-tri-*O*-benzyl-4-*C*-methyl-1-thio-*β*-D-glucopyranosylsulfoxide-*^{13}C* (45) after Tf_2O addition at -80°C.

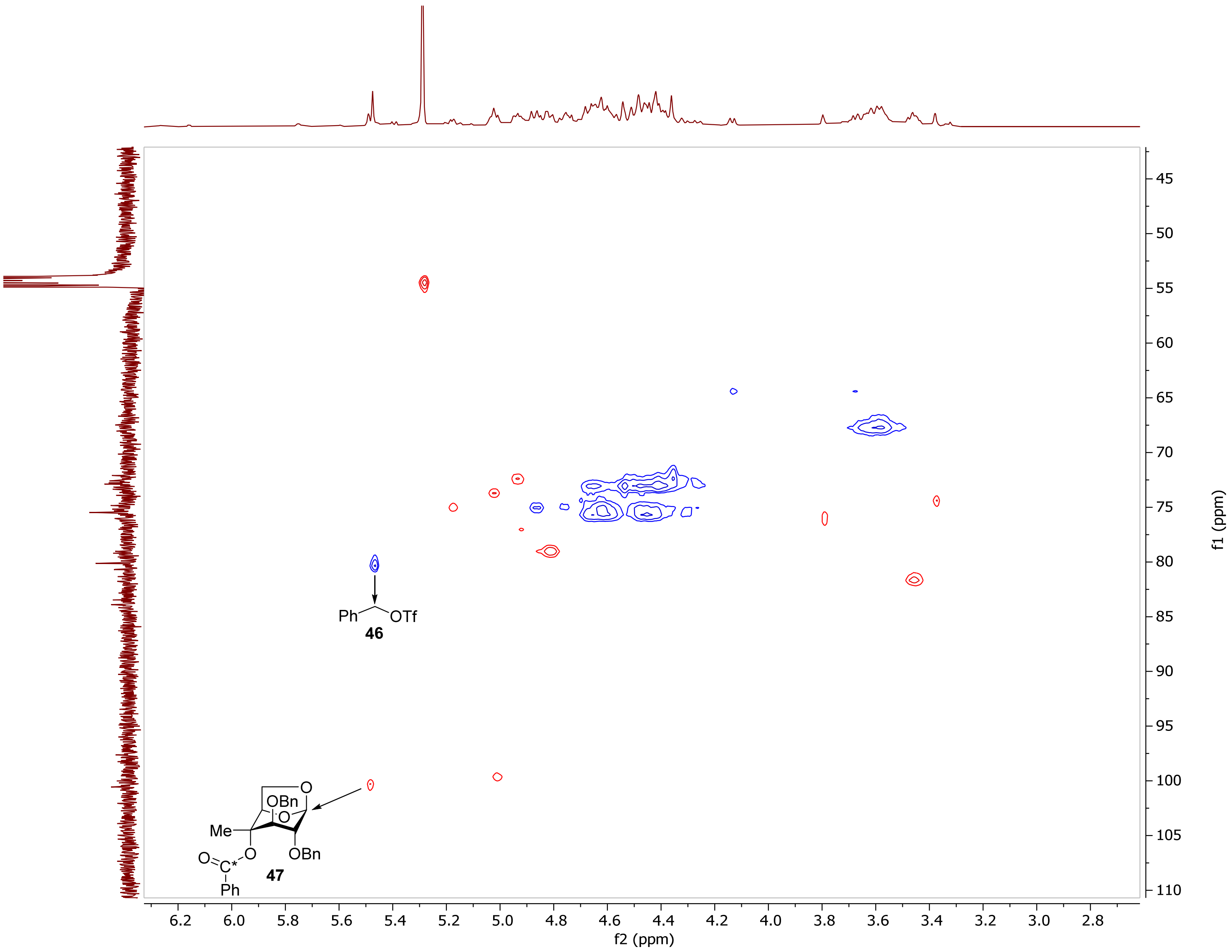

^{1}H NMR (500 MHz, $CDCl_3$) spectrum of 1,6-anhydro-4-*O*-benzoyl-2,3-di-*O*-benzyl-4-*C*-methyl-*β*-D-glucopyranose-^{13}C (47)

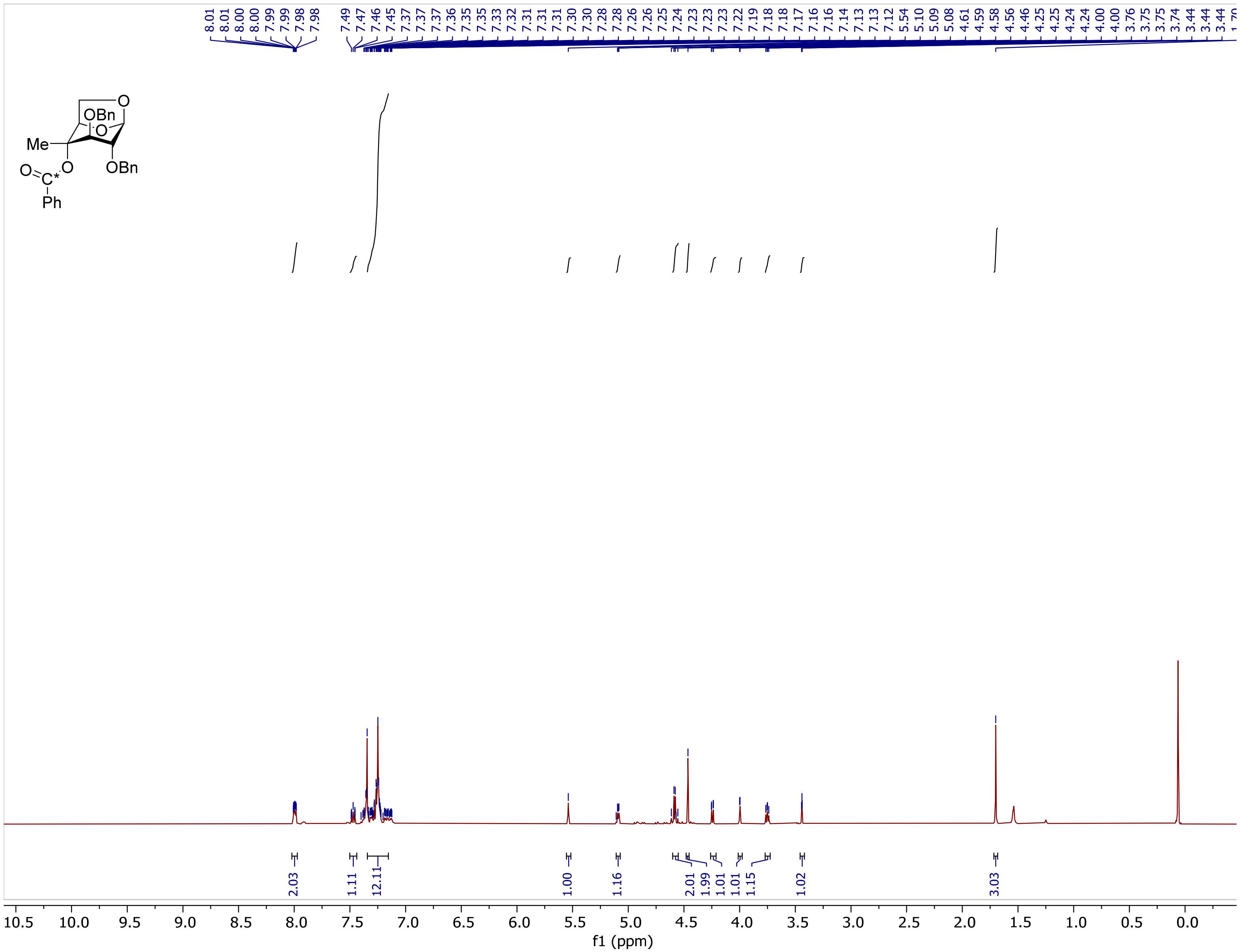

$^{13}C\{^{1}H\}$ NMR (126 MHz, $CDCl_3$) spectrum of 1,6-anhydro-4-*O*-benzoyl-2,3-di-*O*-benzyl-4-*C*-methyl-*β*-D-glucopyranose-*^{13}C* (47)

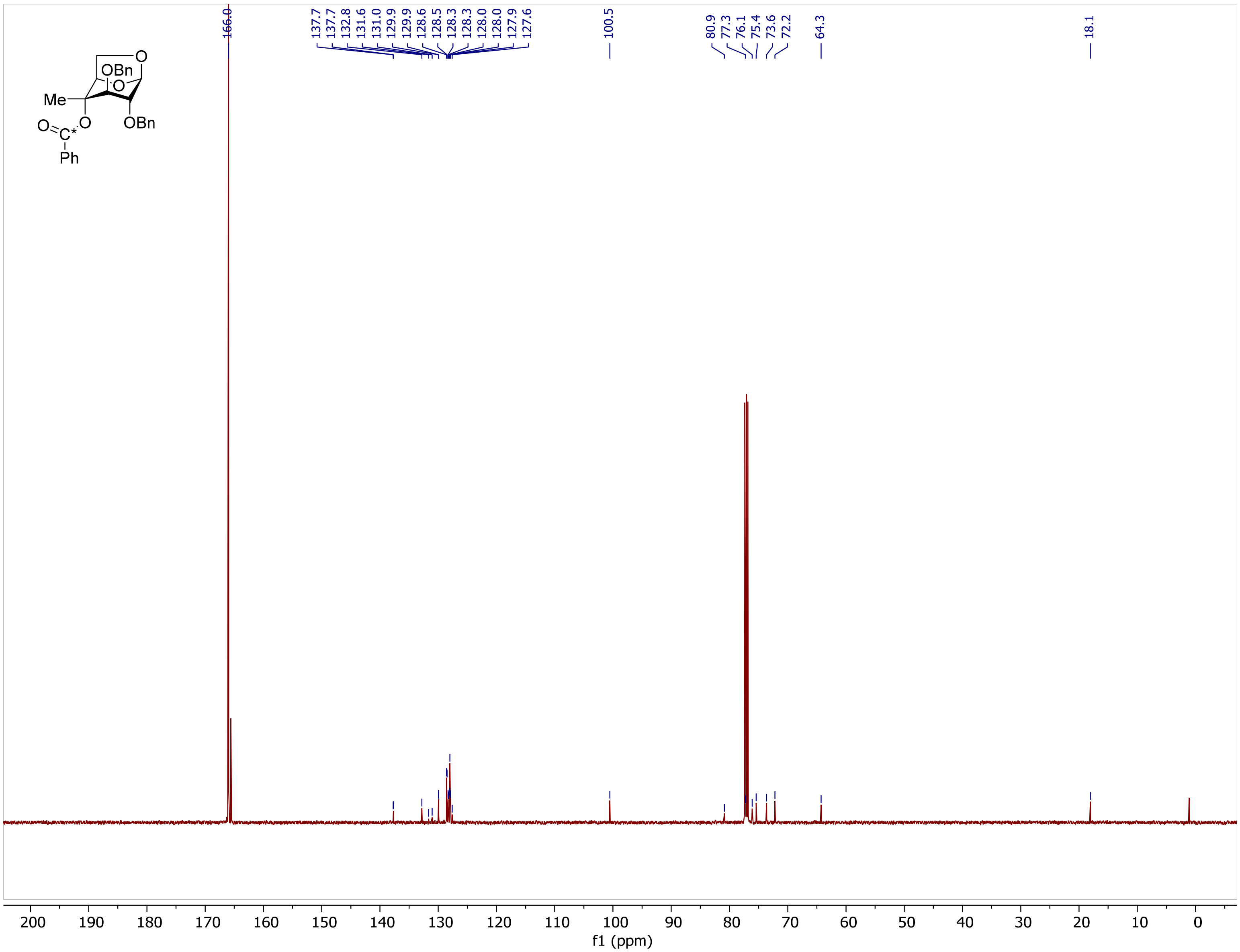

COSY NMR (500 MHz, CDCl$_3$) spectrum of 1,6-anhydro-4-*O*-benzoyl-2,3-di-*O*-benzyl-4-*C*-methyl-*β*-D-glucopyranose-^{13}*C* (47)

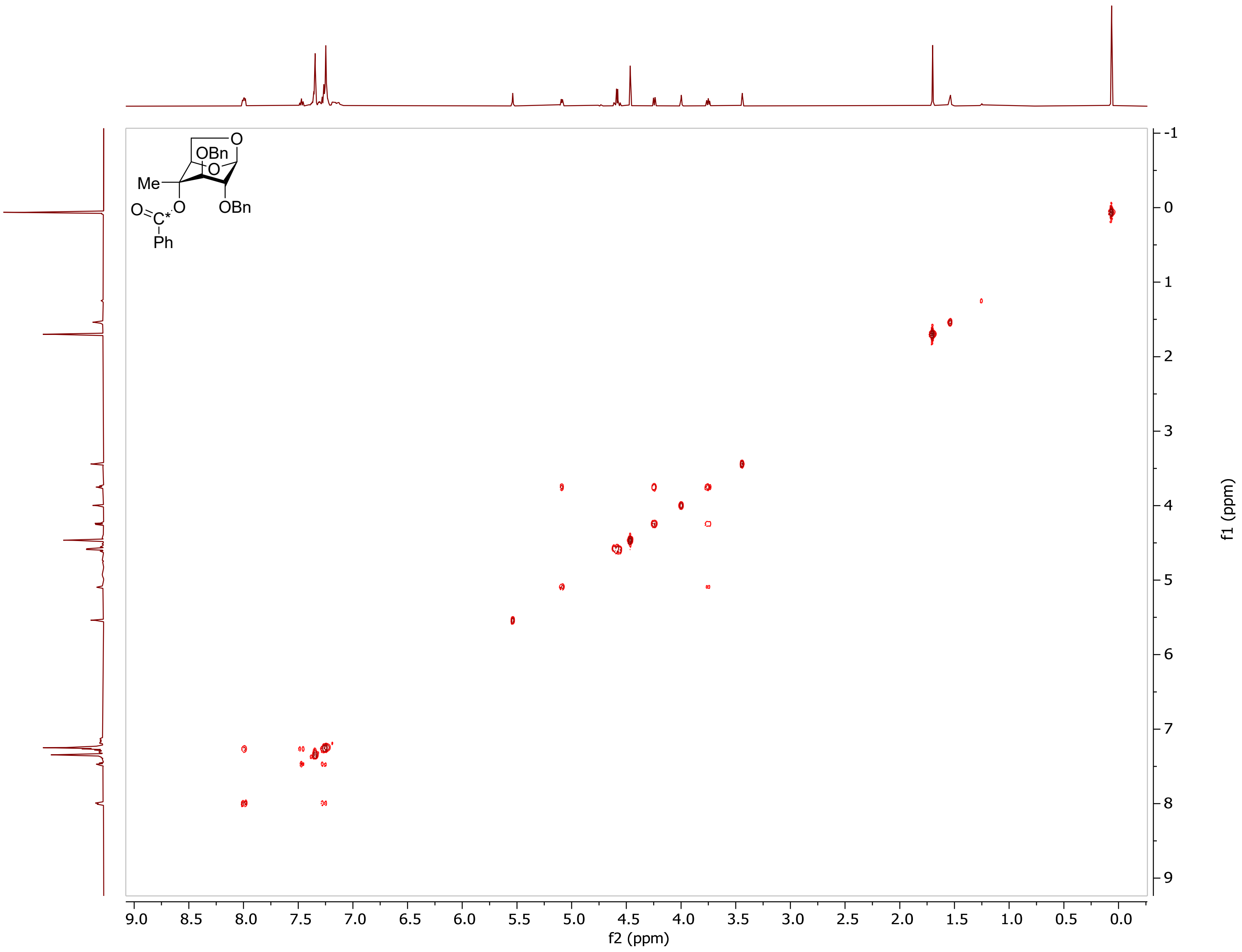

HSQC NMR (500 MHz, $CDCl_3$) spectrum of 1,6-anhydro-4-*O*-benzoyl-2,3-di-*O*-benzyl-4-*C*-methyl-*β*-D-glucopyranose-^{13}*C* (47)

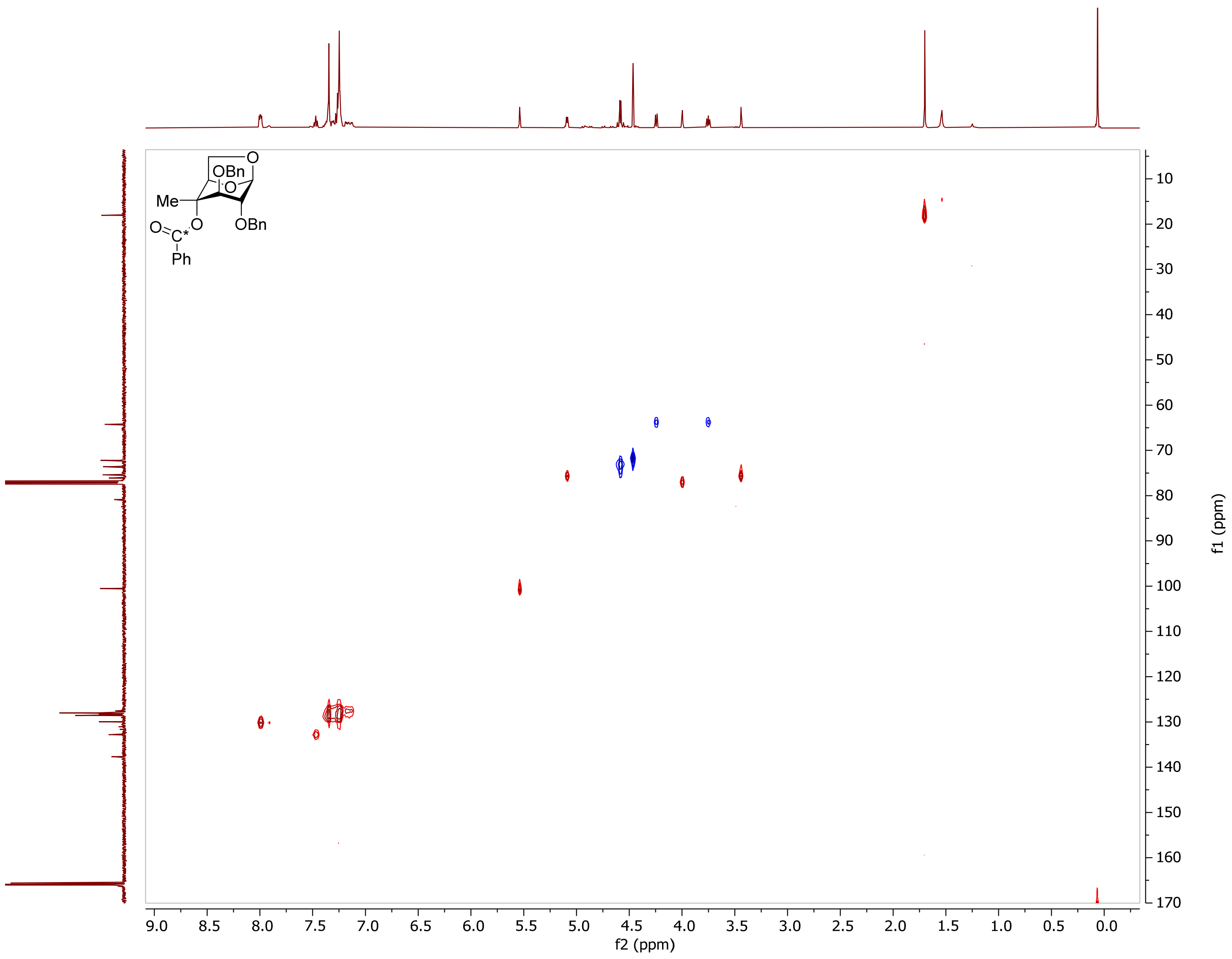

UHPLC-MS chromatogram of the crude reaction of competitive glycosylation of donors **29** and **30** (conversion was determined by ratio of height)

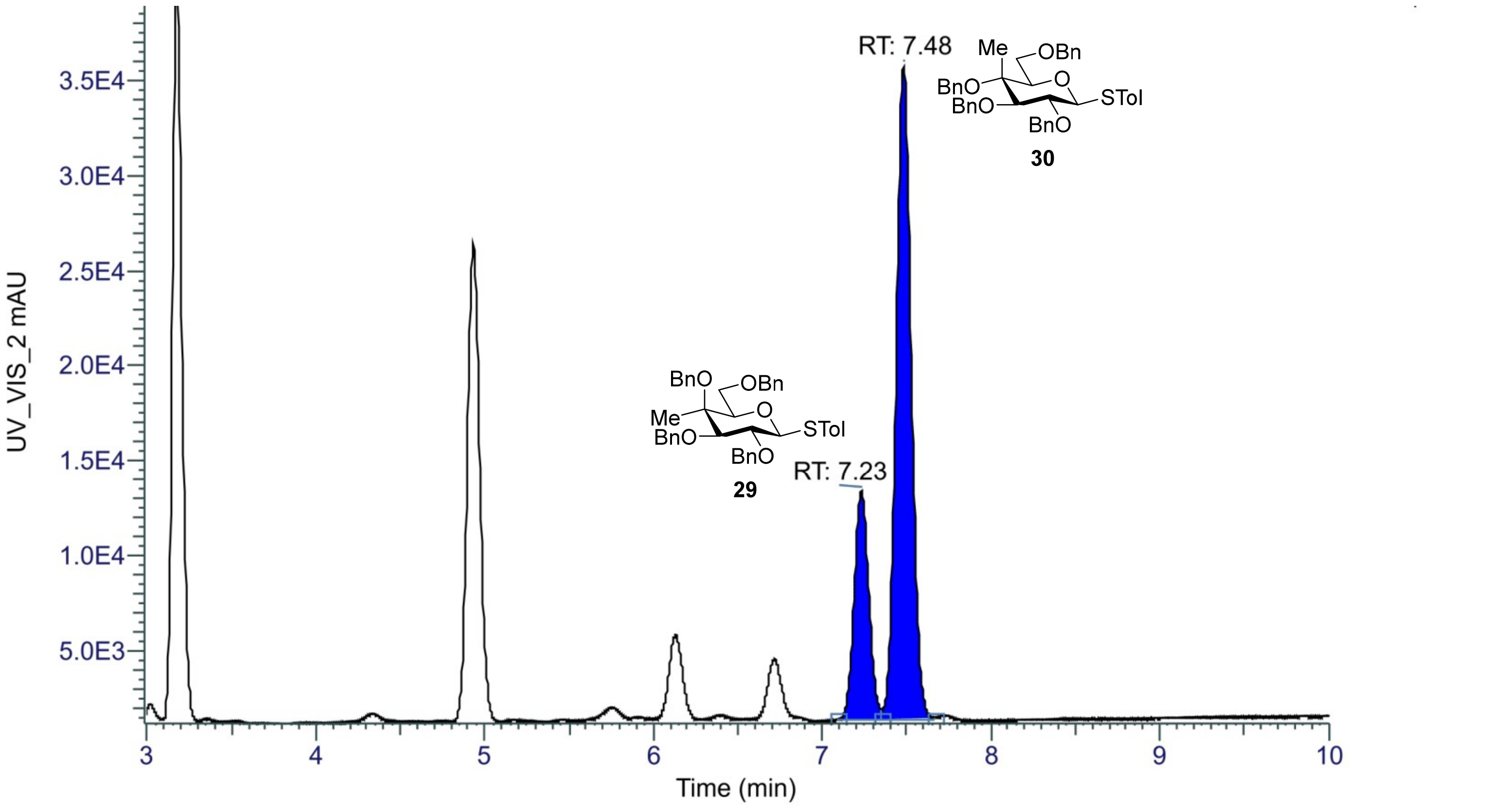

Index	RT (Min)	RT (Sec)	Start RT	End RT	Peak Area	Peak Height	Baseline W...	TraceId	% Area	% Height
1	7.23	433.8	7.08	7.35	7.264E+4	1.202E+4	0.27	Trace1	25.25	25.94
2	7.48	448.8	7.35	7.66	2.15E+5	3.431E+4	0.31	Trace1	74.75	74.06

Table S3. Flow rate: 1.00 (ml/min), injection volume 1 uL. Mobile phase A: CH_3CN, Mobile Phase B 0.1% (v/v) formic acid in water

Time (min	Mobile Phase A (%)	Mobile Phase B (%)
0	5	95
5	0	100
9.5	50	50

UHPLC-MS chromatogram of the crude reaction of competitive glycosylation of donors **3** and **28** (conversion was determined by ratio of area/height)

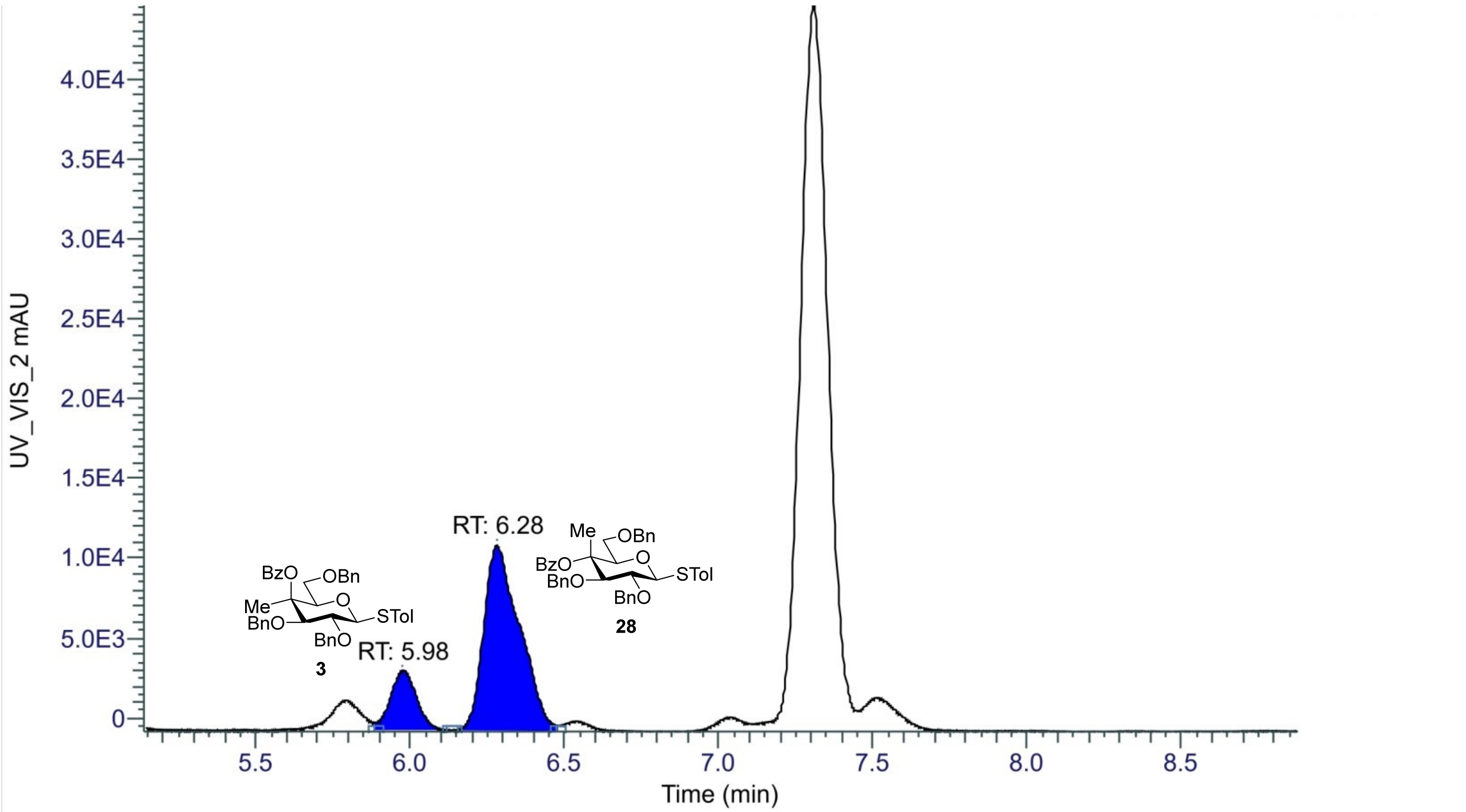

Index	RT (Min)	RT (Sec)	Start RT	End RT	Peak Area	Peak Height	Baseline W...	TraceId	% Area	% Height
1	5.98	358.6	5.88	6.12	2.218E+4	3.73E+3	0.24	Trace1	19.15	24.38
2	6.28	376.8	6.14	6.47	9.362E+4	1.157E+4	0.34	Trace1	80.85	75.62

Table S4. Flow rate: 1.00 (ml/min), injection volume 2 uL. Mobile phase A: CH_3CN, Mobile Phase B 0.1% (v/v) formic acid in water

Time (min	Mobile Phase A (%)	Mobile Phase B (%)
0	5	95
5	0	100
9.5	50	50

UHPLC-MS chromatogram of the crude reaction of competitive glycosylation of donors **3** and **29** (conversion was determined by ratio of area/height)

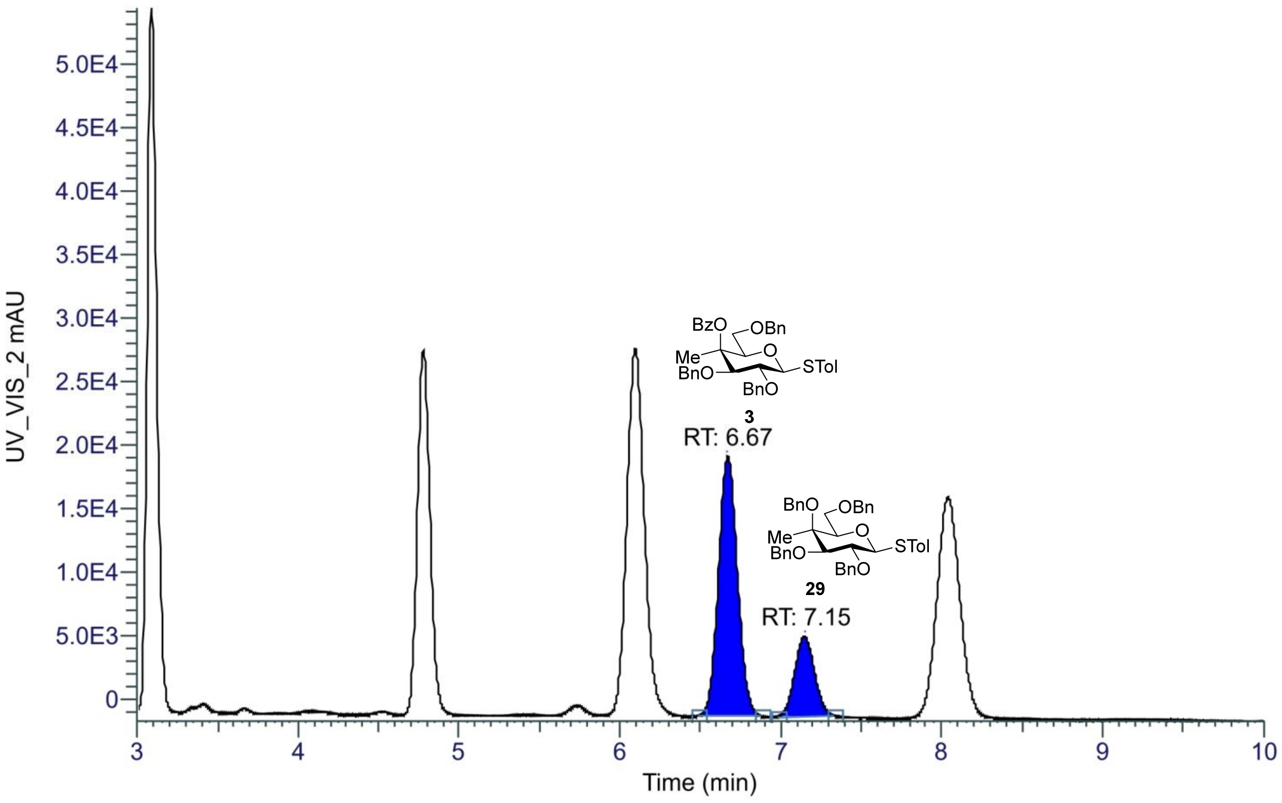

Index	RT (Min)	RT (Sec)	Start RT	End RT	Peak Area	Peak Height	Baseline W...	TraceId	% Area	% Height
1	6.67	400.2	6.49	6.88	1.661E+5	2.036E+4	0.39	Trace1	75.71	76.56
2	7.15	428.8	6.98	7.33	5.328E+4	6.233E+3	0.35	Trace1	24.29	23.44

Table S5. Flow rate: 1.00 (ml/min), injection volume 2 uL. Mobile phase A: CH_3CN, Mobile Phase B 0.1% (v/v) formic acid in water

Time (min	Mobile Phase A (%)	Mobile Phase B (%)
0	5	95
5	5	95
9.5	50	50

^{1}H NMR (500 MHz, $CDCl_3$) spectrum of 6-*O*-(4-*O*-benzoyl-2,3,6-tri-*O*-benzyl-4-*C*-methyl-*β*-D-galactopyranosyl)-1,2:3,4-di-*O*-isopropylidene-*α*-D-galactopyranose (36β)

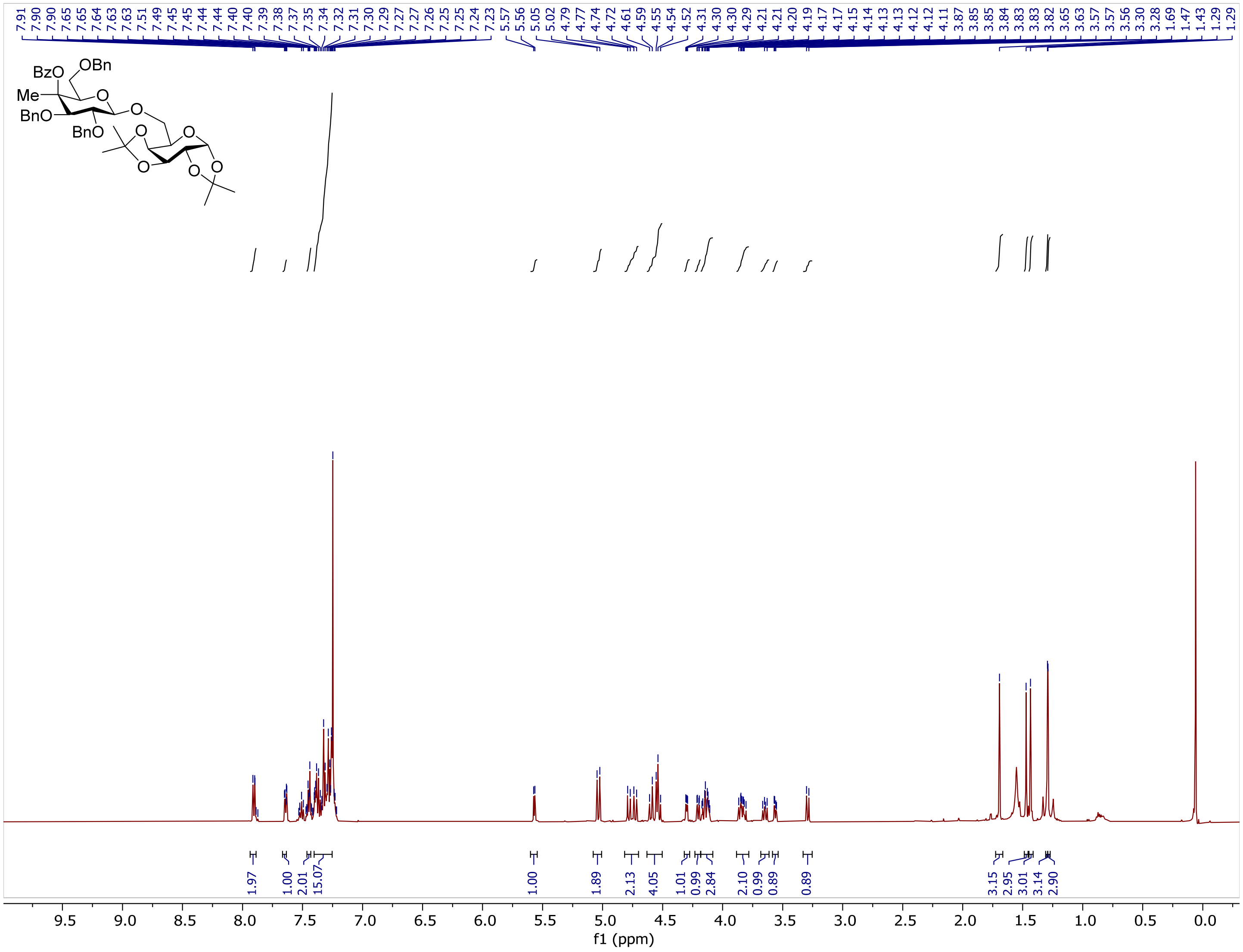

$^{13}C\{^{1}H\}$ NMR (126 MHz, $CDCl_3$) spectrum of 6-*O*-(4-*O*-benzoyl-2,3,6-tri-*O*-benzyl-4-*C*-methyl-*β*-D-galactopyranosyl)-1,2:3,4-di-*O*-isopropylidene-*α*-D-galactopyranose (36β)

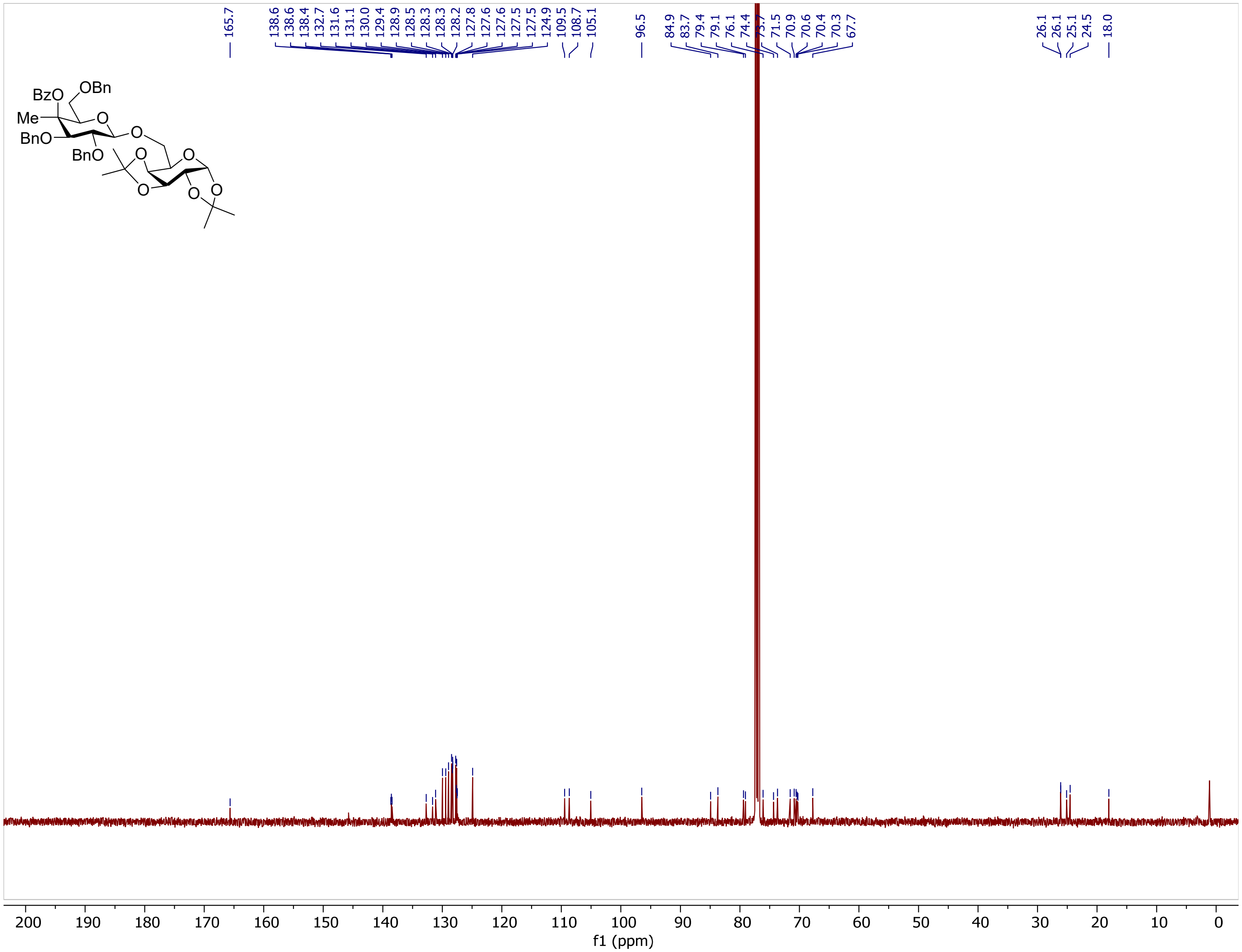

COSY NMR (500 MHz, CDCl₃) spectrum of 6-*O*-(4-*O*-benzoyl-2,3,6-tri-*O*-benzyl-4-*C*-methyl-*β*-D-galactopyranosyl)-1,2:3,4-di-*O*-isopropylidene-*α*-D-galactopyranose (36β)

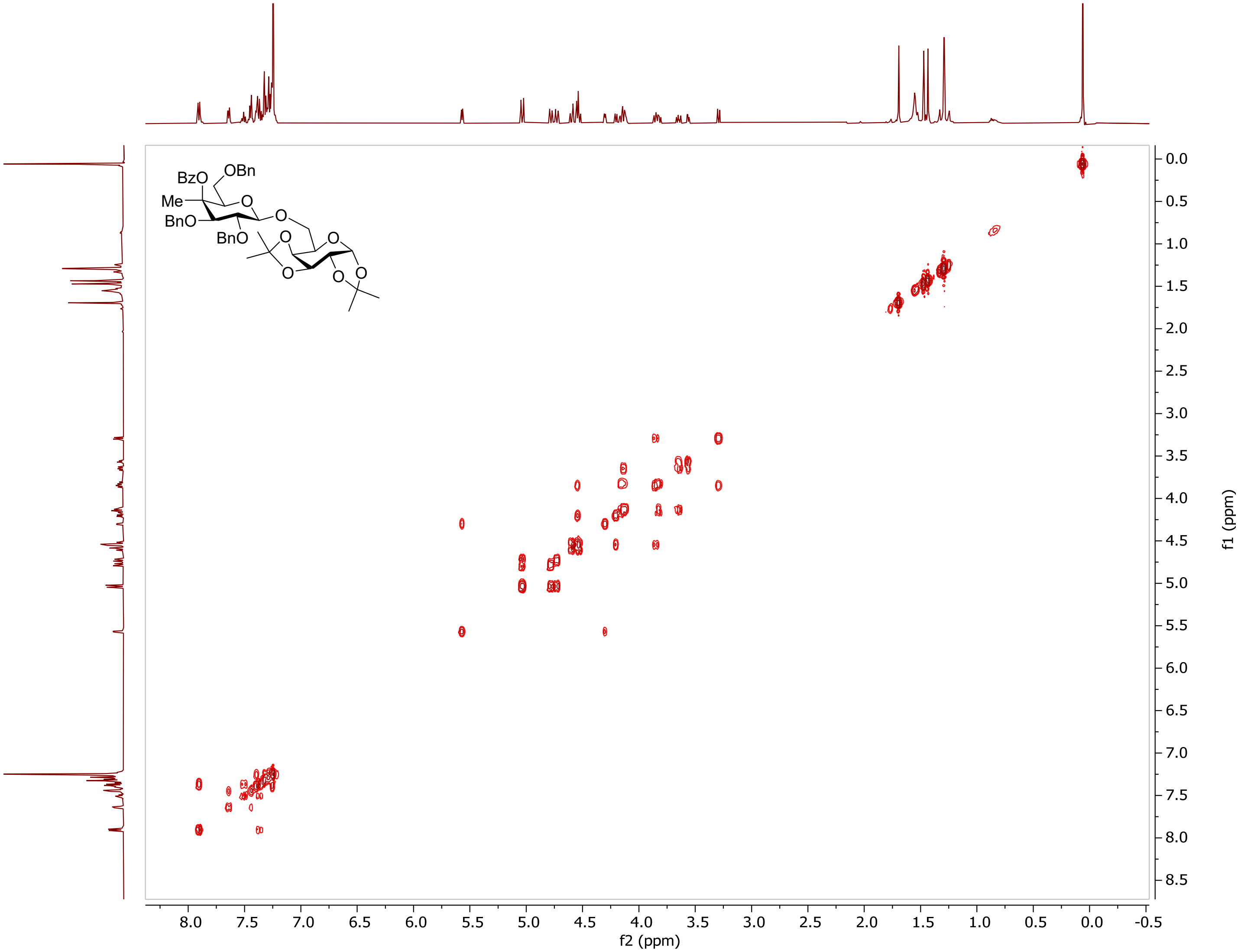

HSQC NMR (500 MHz, $CDCl_3$) spectrum of 6-*O*-(4-*O*-benzoyl-2,3,6-tri-*O*-benzyl-4-*C*-methyl-*β*-D-galactopyranosyl)-1,2:3,4-di-*O*-isopropylidene-*α*D-galactopyranose (36β)

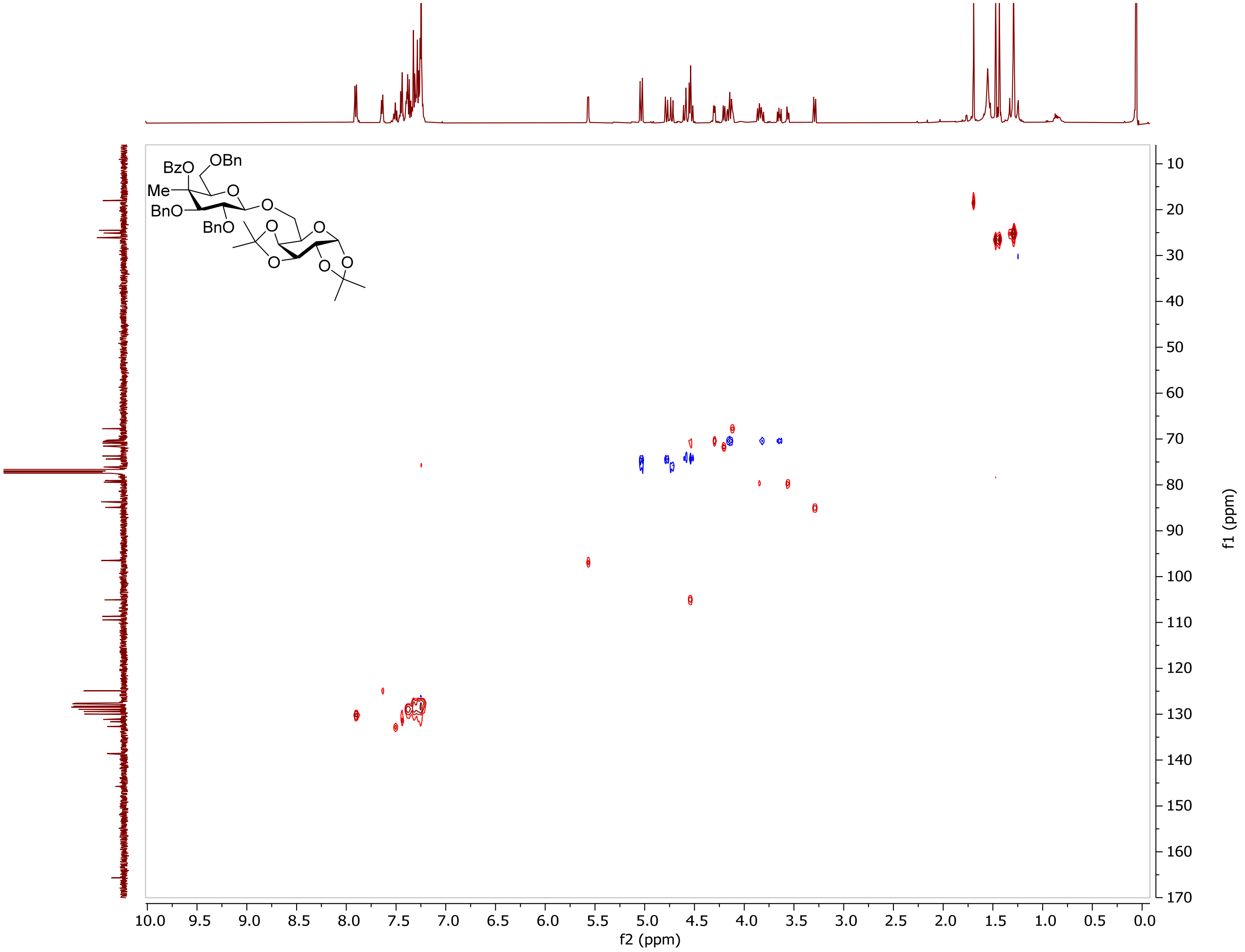

^{1}H NMR (500 MHz, CD_3CN) spectrum of ethyl 2,3-bis-*O*-benzyl-4,6-bis(2,2-dimethylpropanoate)-1-thio-*β*-D-galactopyranoside (51)

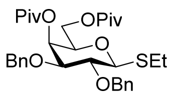

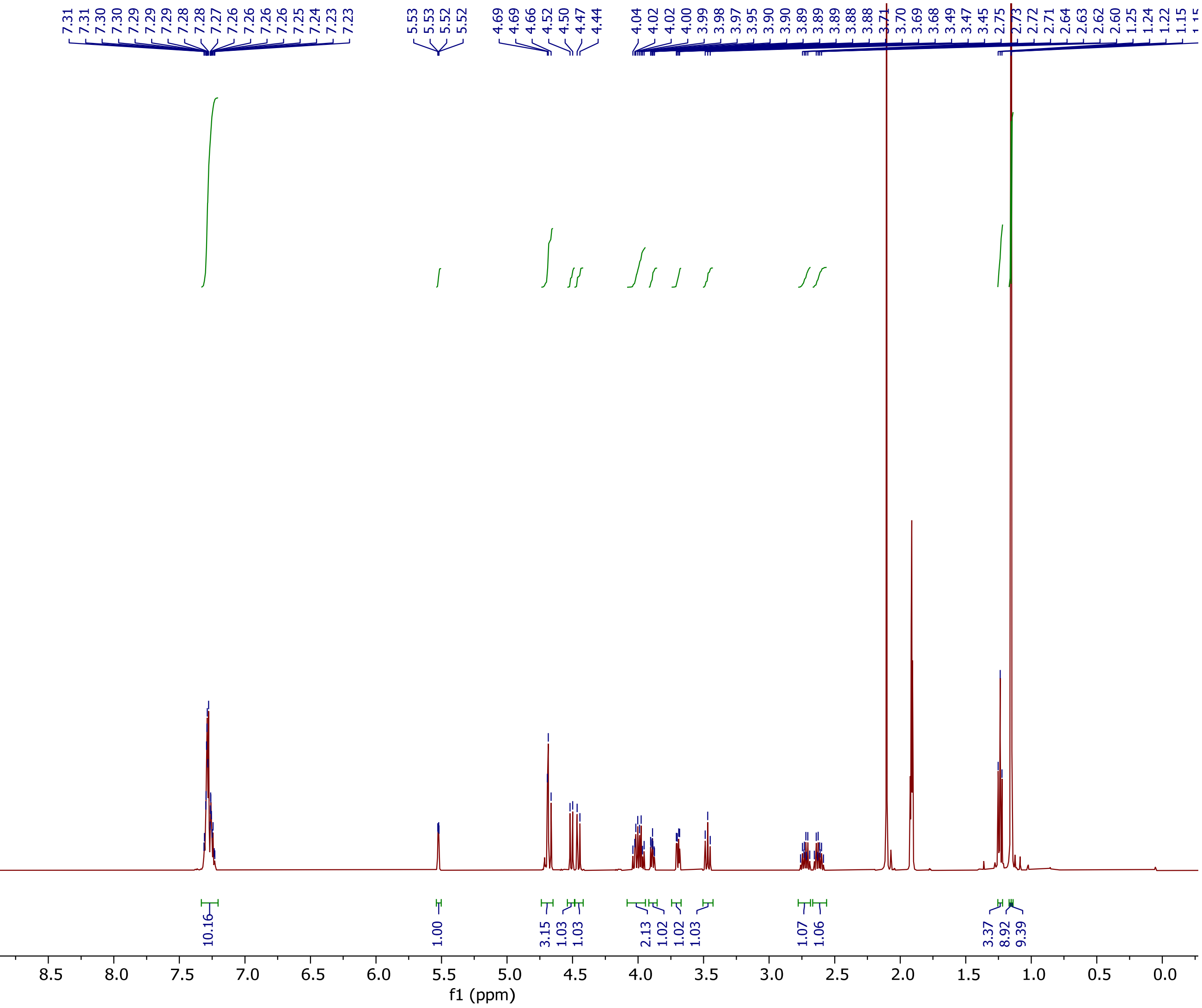

$^{13}C\{^1H\}$ NMR (126 MHz, CD_3CN) spectrum of ethyl 2,3-bis-*O*-benzyl-4,6-bis(2,2-dimethylpropanoate)-1-thio-*β*-D-galactopyranoside (51)

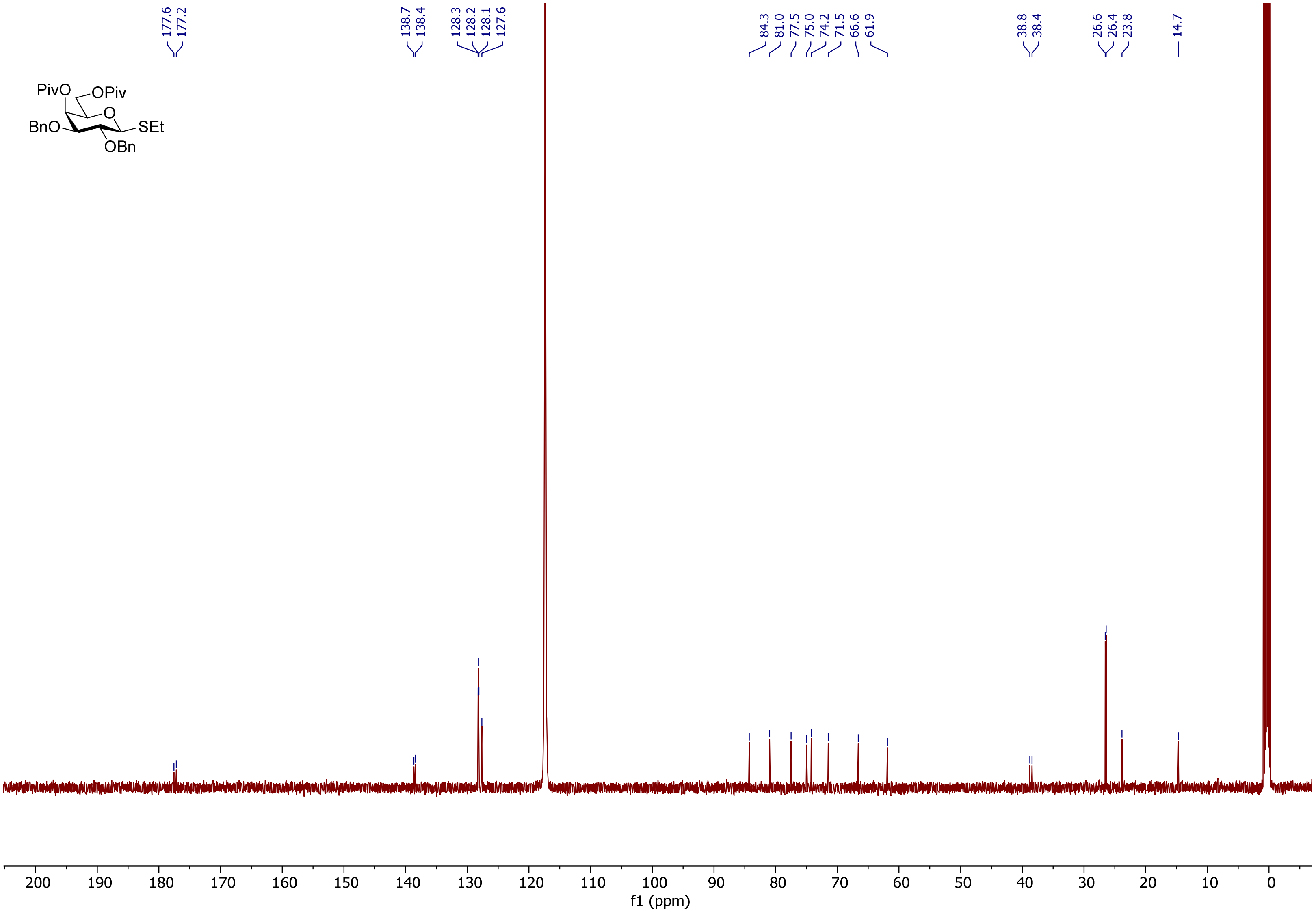

COSY (500 MHz, CD_3CN) spectrum of ethyl 2,3-bis-*O*-benzyl-4,6-bis(2,2-dimethylpropanoate)-1-thio-*β*-D-galactopyranoside (51)

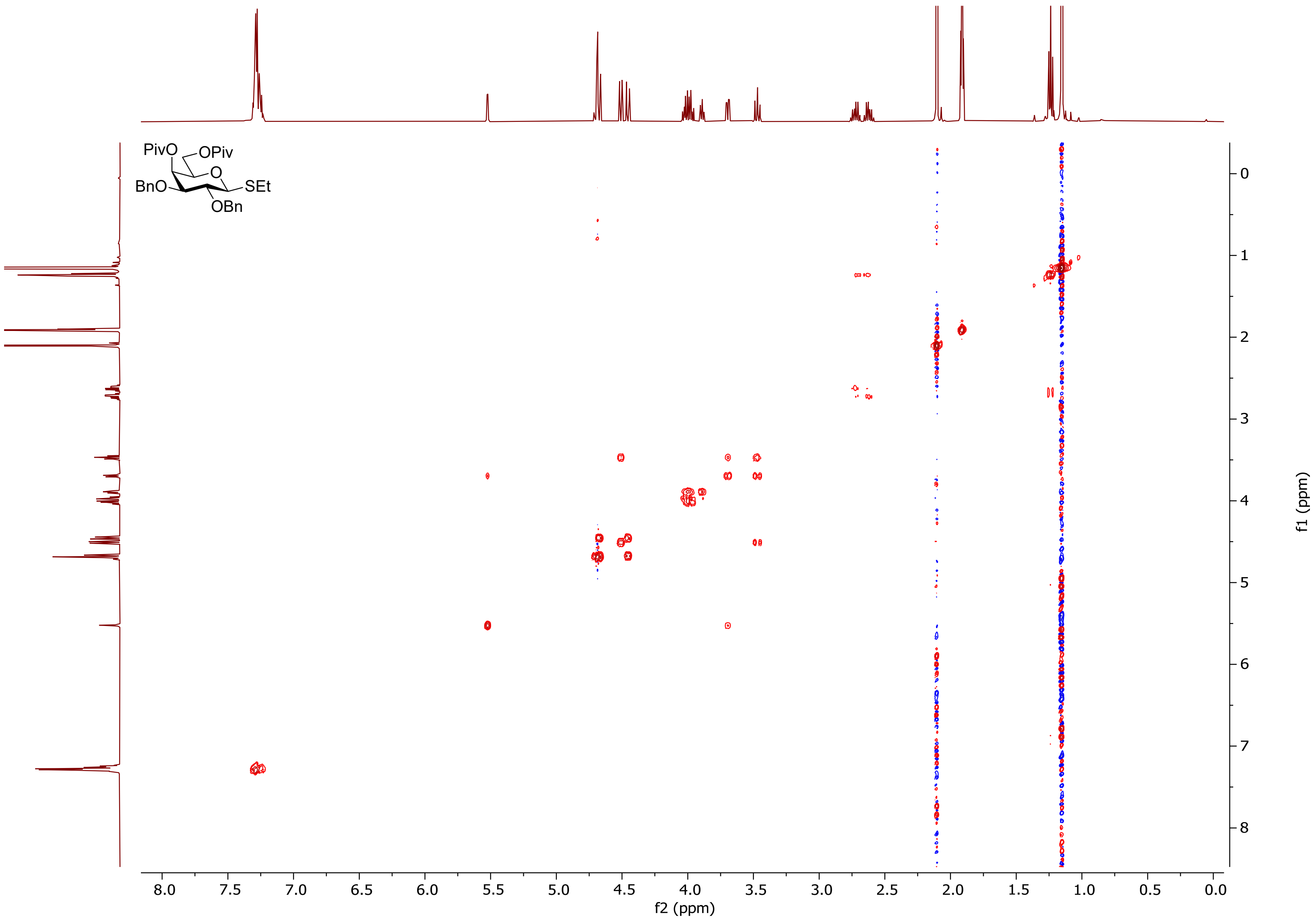

HSQC (500 MHz, CD_3CN) spectrum of ethyl 2,3-bis-*O*-benzyl-4,6-bis(2,2-dimethylpropanoate)-1-thio-*β*-D-galactopyranoside (**51**)

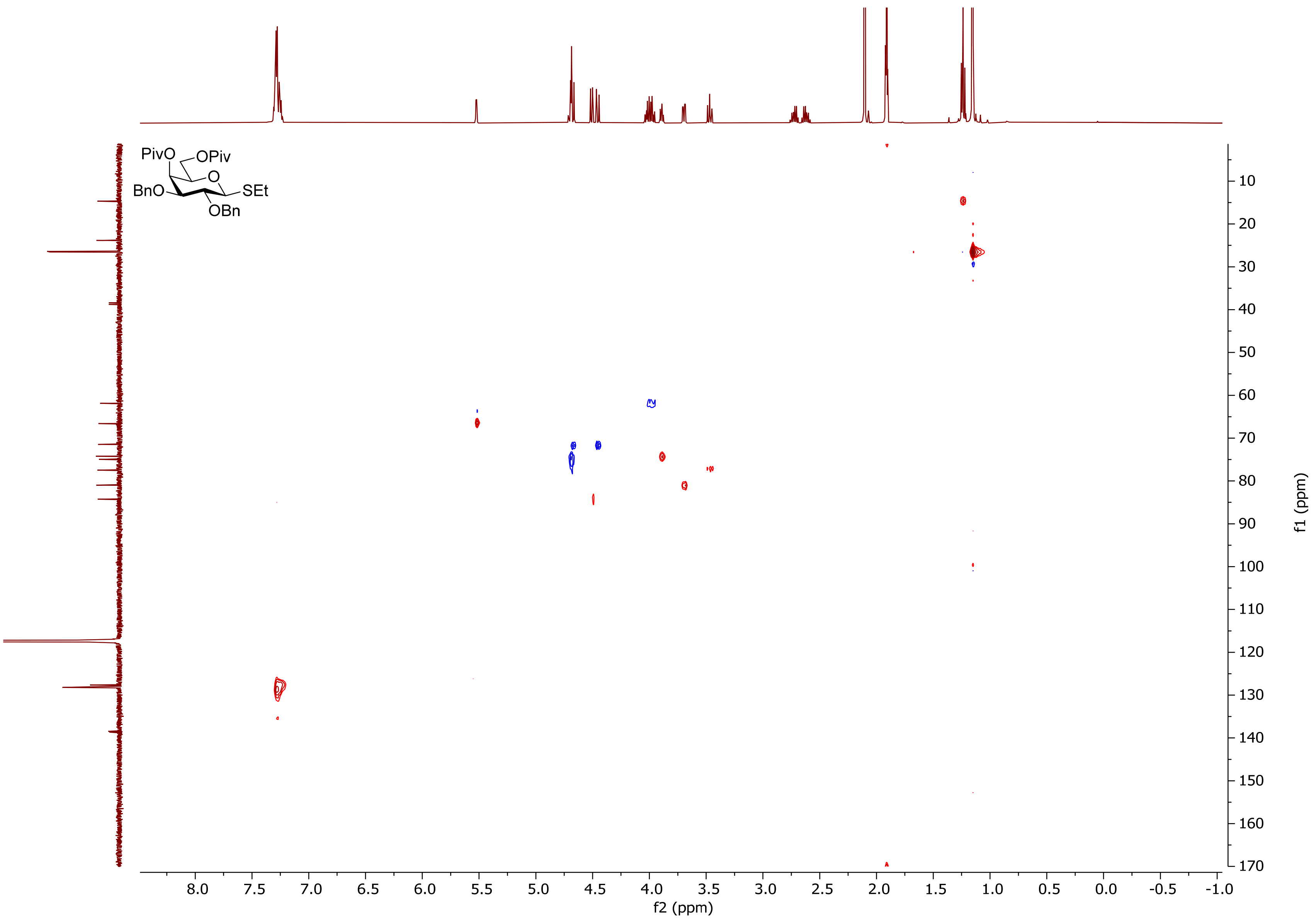

Supplement: Supplementary file 1 — jo3c01496_si_001.pdf [file jo3c01496_si_001.pdf]
